# Supplementary material for: Scoping review of assessment tools for, magnitudes of and factors associated with problem drinking in population-based studies
Source: BMJ Open. 2024 Mar 8;14(3):e080657. doi: 10.1136/bmjopen-2023-080657 (PMC10928735; doi:10.1136/bmjopen-2023-080657)
Supplement: Supplementary data [file bmjopen-2023-080657supp001.pdf]

Supplementary File 1

Search Strategy used for a study "A scoping review of assessment tools for, magnitudes of, and factors associated with problem drinking in population-based studies," 2023.

A) PubMed/MEDLINE:

((((Prevalence [Title/Abstract]) OR "Prevalence" [Mesh])) AND (((alcohol* [Title/Abstract] OR "alcohol abuse" [Title/Abstract] OR "alcohol use" [Title/Abstract] OR "alcohol use disorder" [Title/Abstract] OR "alcohol dependence" [Title/Abstract] OR "alcohol consumption" [Title/Abstract] OR "heavy drinking" [Title/Abstract] OR "risk drinking" [Title/Abstract] OR "harmful drinking" [Title/Abstract] OR "hazardous drinking" [Title/Abstract] OR "binge drinking" [Title/Abstract])) OR ("Alcohol Drinking" [Mesh] OR "Alcoholism" [Mesh] OR "Binge Drinking" [Mesh]))) AND (((Ethiopia [Title/Abstract] OR community-based [Title/Abstract] OR "community based" [Title/Abstract] OR population-based [Title/Abstract] OR "population based" [Title/Abstract])) OR ("Ethiopia"[Mesh] OR "Health Surveys/epidemiology" [Mesh] OR "Population Health/epidemiology" [Mesh]))

B) EMBASE:

1. exp prevalence/
2. prevalence.ti. or prevalence.ab.
3. **1 or 2**
4. exp alcohol consumption/ or exp alcohol/ or exp alcohol abuse/
5. exp alcoholism/ or exp drinking behavior/ or exp binge drinking/
6. (alcohol$ or "alcohol abuse" or "alcohol use" or "alcohol consumption" or "binge drinking").ti. or (alcohol$ or "alcohol abuse" or "alcohol use" or "alcohol consumption" or "binge drinking").ab.
7. **4 or 5 or 6**
8. exp Ethiopia/
9. "community based".mp.
10. "population based".mp.
11. exp primary health care/

12. (Ethiopia or 'community based' or 'population based' or 'primary health care').ti. or (Ethiopia or 'community based' or 'population based' or 'primary health care').ab.

13. 8 or 9 or 10 or 11 or 12

14. 3 and 7 and 13

15. limit 14 to dd=20190826-20220722

16. limit 14 to rd=20190826-20220722

17. 15 or 16

18. limit 14 to dd=20220722-20231125

19. limit 14 to rd=20220722-20231125

20. 18 or 19

C) PsycINFO:

1. prevalence.mp.
2. prevalence.ti. or prevalence.ab.
3. exp "Alcohol Use Disorder"/ or exp Alcohol Abuse/ or exp Alcohol Drinking Patterns/
4. exp Binge Drinking/ or exp Drinking Behavior/ or exp Alcoholism/
5. (alcohol$ or "alcohol abuse" or "alcohol use" or "alcohol consumption" or "binge drinking").ti. or (alcohol$ or "alcohol abuse" or "alcohol use" or "alcohol consumption" or "binge drinking").ab.

6. 1 or 2

7. 3 or 4 or 5

8. ethiopia.mp.
9. "community based".mp.
10. "population based".mp.
11. exp Primary Health Care/
12. (Ethiopia or 'community based' or 'population based' or 'primary health care').ti. or (Ethiopia or 'community based' or 'population based' or 'primary health care').ab.

13. 8 or 9 or 10 or 11 or 12

14. 6 and 7 and 13

15. limit 14 to up=20190826-20220722

16. limit 14 to ch=20190826-20220722

17. 15 or 16

18. limit 14 to up=20220722-20231125

19. limit 14 to ch=20220722-20231125

20. 18 or 19

D) Global Index Medicus (GIM):

(tw:(prevalence)) AND (tw:(alcohol$ OR "alcohol abuse" OR "alcohol use" OR "alcohol consumption" OR "binge drinking")) AND (tw:(Ethiopia OR "community based" OR "population based" OR "primary health care"))

Abbreviations

Date Delivered (dd): the date a citation XML file was produced for distribution to Ovid with the state = "new." The Date Delivered is removed when a record is revised.

Revised Date (rd): the date the citation XML file was produced for distribution to Ovid with the state="update".This date can change if an updated record is delivered to Ovid.

Update Date/Code (up): The date a record was added to the database since the yearly reload completion.

Correction Date (ch): CH field appears in corrected records and contains the date the record was revised.
