# Supplementary material for: Scoping review of assessment tools for, magnitudes of and factors associated with problem drinking in population-based studies
Source: BMJ Open. 2024 Mar 8;14(3):e080657. doi: 10.1136/bmjopen-2023-080657 (PMC10928735; doi:10.1136/bmjopen-2023-080657)
Supplement: Supplementary data [file bmjopen-2023-080657supp002.pdf]

Supplementary File 2

Newcastle-Ottawa Scale (NOS) adapted for quality assessment of cross-sectional studies for the study "A scoping review of assessment tools for, magnitudes of, and factors associated with problem drinking in population-based studies," 2023.

**Selection: (Maximum 5 points/scores/stars)**
**1. Representativeness of the sample:**
a. Truly representative of the average in the target population. * (all subjects/consecutive or random sampling)
b. Somewhat representative of the average in the target group. * (non-random sampling)
c. Selected group of users/convenience sample.
d. No description of the derivation of the included subjects (sampling strategy).
**2. Sample size:**
a. Justified and satisfactory (including sample size calculation). * (1 score)
b. Not justified
c. No information provided
**3. Non-respondents:**
a. Proportion of target sample recruited attains pre-specified target or basic summary of non-respondent characteristics in sampling frame recorded. *
b. Unsatisfactory recruitment rate, no summary data on non-respondents.
c. No information provided
**4. Ascertainment of the exposure (risk factor/disease) or screening/surveillance (measurement) tool:**
a. Secure record (medical charts) or validated measurement (screening/surveillance) tool. **
b. Non-validated measurement tool, but the tool is available or described or Self-report. *
c. No description of the measurement tool.
**Comparability: (Maximum 2 stars)**
**1. Comparability of subjects in different outcome groups on the basis of design or analysis. Confounding factors controlled.**
a. Data/results adjusted for relevant predictors/risk factors/confounders e.g., age, sex, marital status, job etc. **

b. Data/results not adjusted for all relevant confounders/risk factors/information not provided.
**Outcome: (Maximum 3 stars)**
**1. Assessment of outcome:**
a. Independent blind (structured) assessment. **
b. Record linkage. **
c. Self report. *
d. No description.
**2. Statistical test:**
a.Statistical test used to analyse the data clearly described, appropriate, and measures of the association presented including confidence intervals and probability level (p-value). *
b.Statistical test not appropriate, not described, or incomplete.

Scoring for cross-sectional Studies:

Very Good Studies: 9-10 points

Good Studies: 7-8 points

Satisfactory Studies: 5-6 points

Unsatisfactory Studies: 0 to 4 points

References

1. Ribeiro CM, Beserra BTS, Silva NG, Lima CL, Rocha PRS, Coelho MS, et al. Exposure to endocrine-disrupting chemicals and anthropometric measures of obesity: A systematic review and meta-analysis. BMJ open. 2020;10(6):e033509.

2. Zhao C, Xing F, Yeo YH, Jin M, Le R, Le M, et al. Only one-third of hepatocellular carcinoma cases are diagnosed via screening or surveillance: a systematic review and meta-analysis. European journal of gastroenterology & hepatology. 2020;32(3):406-19.

3. Patra J, Bhatia M, Suraweera W, Morris SK, Patra C, Gupta PC, et al. Exposure to second-hand smoke and the risk of tuberculosis in children and adults: a systematic review and meta-analysis of 18 observational studies. PLoS medicine. 2015;12(6):e1001835.

4. Modesti P, Reboldi G, Cappuccio F. Newcastle-Ottawa Quality Assessment Scale (adapted for cross sectional studies). PloS one. 2016;11(1):e0147601.

5. Dubey VP, Kievišienė J, Rauckiene-Michealsson A, Norkiene S, Razbadauskas A, Agostinis-Sobrinho C. Bullying and Health Related Quality of Life among Adolescents—A Systematic Review. Children. 2022;9(6):766.

6. Naafs JC, Vendrig LM, Limpens J, Van Der Lee H, Duijnhoven RG, Marchal J, et al. Cognitive outcome in congenital central hypothyroidism: a systematic review with meta-analysis of individual patient data. European journal of endocrinology. 2020;182(3):351-61.
