# Supplementary material for: Scoping review of assessment tools for, magnitudes of and factors associated with problem drinking in population-based studies
Source: BMJ Open. 2024 Mar 8;14(3):e080657. doi: 10.1136/bmjopen-2023-080657 (PMC10928735; doi:10.1136/bmjopen-2023-080657)
Supplement: Supplementary data [file bmjopen-2023-080657supp003.pdf]

Supplementary File 3

Table: Prevalence, associated factors, and pattern of problem drinking in high-income countries (HICs), 2023.

**Author, Year**<br><br>**Country/Location**	**Study Design & Study Setting (population)**	**Participants:** **Sample size** (Male subjects, %) Mean age (range) in years	**Tools (measures) or questions used**	**Outcomes:** **(Definition/nature of use)**	**Results & statistical methods used.**
**Aalto et al., 1999**<br><br>**Finland (town of Lahti)**	**Cross-sectional** PHC outpatients & General population (Urban residents)	PHC,2370 (40.3%) OHC,3268 (29.3%) GNP,544 (51.7%) 38-41(20-60) years	Quantity or frequency questionnaires **(QFQs)** (last 2 month) **CAGE**	**Heavy drinking:** **Male**: ≥ 280g of absolute ethanol /24 drinks/week/ &/or ≥ 3 in CAGE. **Women**: ≥ 190g/16 drinks per wk &/or ≥ 2 in CAGE. **Abstinence:** no self-reported drinking at all & no answers to CAGE	**t-test & Chi-square analysis:** **Men:** heavy drinking in PHC, OHC & GNP were 19.5%, 17.3% & 16.4%, respectively. **Women:** corresponding figures were 8.6%, 6.2% & 12.9%.
**Aira et al., 2005**<br><br>**Finland (City of Kuopio)**	**Cross-sectional** home-dwelling elderly persons, **Community-based** (Urban residents)	700 persons (27.4% men) 81 (75-95.7) years	**QFQs** (1 year) & **CAGE**	**Four categories:** Abstainers, < 1 unit/week, 1–7 units/week, & > 7 units/week.	**Chi-square & t-test** (frequencies vs means): 44% had used alcohol during past year (65% of men & 36% of women). ≥ 3 units/occasion used by 2.9% of women & by 11.7% of men.
**Andrews-Chavez et al., 2015**<br><br>**United States (Greater Boston area, MA)**	**Cross-sectional** (Puerto Rican adults, Hispanics). (Urban residents)	1472 adults (29.6% men) ? (45–75) years	**QFQs** **NIAAA** definitions **(NIAAA guidelines)**	**Lifetime abstainer (LA):** (**< 12 drinks** in lifetime) **Former drinker (FD):** **(> 12 drinks** in lifetime, but not currently drinking) **Moderate drinker (MD):** (**Man/women**: ≤14/7drinks per week & ≤ 4/3 drinks/d) **Heavy drinker (HD)**: (**Man/women**:>14/7drinks per week & > 4/3drinks/d)	**A multinomial logistic regression model:** 8% men & 39% women were **LAs;** 40% of men & 25% women (**FDs**); **&** 21 % men & 8 % of women **(HDs).** **Young men**: likely than older to be MDs. **Women**: higher BMI, age, lower income & psychological acculturation (associated with abstention); age, lower perceived emotional support associated with increased FD; & women without v. with diabetes were more likely to be heavy drinkers.
**Bataille et al., 2003**<br><br>**France (Lille, Strasbourg & Toulouse)**	**Cross-sectional** (3rd MONICA) **Population survey** (Urban/Semi-urban & rural)	3508 subjects (51.0% men) 50.3 (35–64) years	Self-reported **QFQs** French alcohol consumption habits	**Heavy drinkers:** **Men:** ≥ 60g ethanol/day, (6 glasses/d-any drink) & **Women:** ≥ 30g/day (3 glasses/day)	**Multivariate analyses:** 14% men & 40.8% women (non-drinkers) 9.0% women & 14.4% of men were HDs. Low educational level, smoking, apoprotein B, HDL, MCV), GGT & CAGE score for men, & living area, age, MCV, GGT & the CAGE score for women were significantly associated with **heavy drinking (HD).**

				**Reference class (RC):** non-drinkers & moderate drinkers together.	
**Coulson et al., 2010**<br><br>**Australia (south Eastern)**	**Cohort study** (Geelong Osteoporosis Study, GOS)<br>**Community-based cohort** (secondary data)	1420 men (100%)<br>56 (20 – 93) years	Validated self-report **FFQ**<br>**Mean daily alcohol intake** (Australian National Health & MRC 2009 guidelines)	**Consumption/12 months**: (never, < 1/month, 1–3 days/month, 1–6 days/week & every day<br>**Mean daily alcohol intake** non-drinkers/nil, > 0 but ≤ 2 drinks/ day, > 2 drinks/day<br>**(with in past 12 months)**	**ANOVA & Multivariate analyses:**<br>Age-standardized proportion of non-drinkers was 8.7%, 51.5% consumed ≤ 2 drinks/day (≤ 20g/day), & 39.9% > 2 standard drinks per day (> 20g ethanol/day).<br>**Alcohol use (> 20g/day)** was positively associated with cigarette smoking, weight, higher SES & inversely with age & physical activity.
**Foulds et al., 2012**<br><br>**New Zealand**	**Cross-sectional** (Permanent private dwellers)<br>**Population survey**	12,488 adults (42.2% male)<br>? (≥ 15 years)	**AUDIT**	**Harmful/hazardous drinking (HHD):**<br>Score of ≥ 8 on AUDIT	**Crosstabs & logistic regression models:**<br>**HHD:** 17.7% (men, 25.6%; women, 10.4%);<br>**Overall, 9.4%** of attendees with HHD reported talking about alcohol.
**Geels et al., 2013**<br>**Netherlands**	**Cross-sectional** (All Netherlands Twin Register, NTR registered at a valid address)<br>**Population survey** (Urban)	16,587 subjects (36.5% men)<br>41.6 (18–97) years	**QFQs** (12 mo.)<br><br>Health Council of Netherlands recommended limit<br>CAGE & AUDIT	**Excessive alcohol use:**<br>**Women:** > 14 standard glasses per week<br>**Men:** > 21 drinks/week	**Linear/logistic/multinomial regressions:**<br>>30.0% of men & >20% of women drinking 6–7 times per week)<br>**Women:** 25–45 years had 5.7-5.9% of excessive drinking, & 55–65 years (15.5%)) Older age, sex (male), and initiation of cigarette & cannabis use were predictors of alcohol use
**Janghorbani et al., 2003**<br><br>**Hong Kong (China)**	**Cross-sectional** (Cantonese-speaking adult population)<br>**Population-based** (Urban)	2900 subjects (48.7% men)<br>45.8 (25–74) years	**QFQs** (weekly)	**Heavy drinkers:** men, > 400g & women, > 280g/wk<br>**Light drinkers:** men, < 168g & women, <112g/wk<br>**Moderate drinkers:**<br>Men: ≤ 400g/ ≥ 168g & Women: ≤ 280g/ ≥112g/wk<br>**Binge drinking**: ≥ 5 drinks in a row in the past month.	**GLMs/multiple/logistic regression models:**<br>**Mean weekly alcohol consumption**: 64.3g, men & 13.7g, women (P < 0.001).<br>Current drinking vs non-drinking, male sex, smoking (women), HDL, ≤ primary education, diastolic BP & separated/widowed were associated positively with **weekly ethanol consumption.**
**Kim et al., 2008**<br><br>**Hong Kong (China)**	**Cross-sectional** (All Hong Kong Chinese adults)<br>**Population based** (Urban)	9860 adults (50.0% men)<br>28 (18–70) years	Pattern **(QFQs)**<br><br>**CIDI** (Chinese version based on DSM-IV)	**Mean drinking/past year:** < once/wk, 1–3 times/wk, 4+ times/week)<br>**Binge drinking/past mo.** (5 servings of alcohol per one occasion in 30 days)<br>**Alcohol abuse or dependence**	**Stepwise multivariate logistic regression:**<br>**10.9% of entire sample** reported at least one of AUDs (AA, AD & binge drinking).<br>**Binge drinking :**14.5% in males (18.7% AA & 12.3% AD) & 3.5% in females (16.0% AA & 9.9% AD)<br>**Male binge drinkers** were less likely to be older & students but more likely to be employed in service industry.

				(Chinese CIDI)	**Female binge drinker**: less likely to be > 60 years or married & more likely to be smokers<br>**In both genders,** smoking was significantly associated with binge drinking
**(Chou et al., 2011**<br><br>**United States**	**Prospective study** (subsample of 3-year prospective study**; waves 1 & 2 of NESARC**)<br>**Population-based** (Urban)<br>**secondary data analyses**	13,442 analyzed (40.6% men)<br>? (≥ 50 years)	**QFQs**<br><br>**AUDADIS-IV** (DSM-IV)	**Binge drinking (BD):**<br>≥ 5 drinks/occasion (men)<br>≥ 4 per occasion (women)<br>**Current drinkers:** without BD<br>**Occasional BD:** < monthly in past year) &<br>**Frequent BD:** ≥ 1/month in past year<br>**DSM-IVAUDs** (Alcohol use, AA & AD)	**Multinomial & logistic regression:**<br>BD was 24.7% in men & 12.4% in females.<br>Overall, male respondents were significantly more likely to have BD.<br>Both men & women with occasional BD & frequent BD were significantly more likely than current male/female drinkers without BD to have **alcohol abuse disorder and alcohol dependence disorder (AUDs)**
**Latvala et al., 2009**<br><br>**Finland**	**Cross-sectional** (Finnish young adults)<br>**Population-based** (Urban)	605-diagnostic assessment done (sex unspecified)<br>28.6 (21-35) years	**SCID-I** complemented by medical record data	**Lifetime Substance Use Disorders (SUDs):**<br><br>DSM-IV diagnosis	**t-tests,** X^2 **tests & logistic regression:**<br>Lifetime AA or AD were 13.1% (19.8% for males & 6.3% for females). And total prevalence of AA & AD alone was 7.6% & 5.6%.<br>Behavioral, affective & parental factors, early initiation of substance use, learning difficulties & lower education were found to be associated with **alcohol & other SUDs.**
**Meyer et al., 2000**<br><br>**Germany (Northern, city of Lubeck)**	**Cross-sectional** of longitudinal project (Adult general population)<br>(Urban)	4075 analyzed (50.2% of men)<br>? (18 to 64 years)	**M-CIDI** (DSM-IV, adapted CIDI)<br>**Ever/current** QFQs	**Hazardous consumption:**<br>20-40g/d (women) & 30-60g/day (men) and<br>**Harmful consumption:**<br>> 40g/day (women) &<br>> 60g/d (men)<br>**AA or AD**:<br>DSM-IV Diagnosis (M-CIDI diagnostic software)	**Logistic regression analyses:**<br>Lifetime AUDs (4.5% AA, 3.8% AD) & men vs women for AA (8.1% vs 1.0%) & AD (6.0% vs 1.5%) respectively<br>**Hazardous & harmful consumption:** (13.2% lifetime; 6.0% in last 12-months)<br>**Male:** more affected by lifetime AUDs.<br>Association between AUDs & alcohol consumption pattern revealed a weaker relation for AA compared to AD.
**Miller et al., 2004**<br><br>**United States**	**Cross-sectional** (US Adults; BRFSS, telephone survey & NSDUH, an in-person survey)	**355,371** (BRFSS)<br>**87,145** (NSDU) were analyzed (sex unspecified)<br>? (≥ 18 years)	**Pattern (QFQs)**	**Binge drinking**: ≥ 5 drinks on an occasion	**two-tailed t-test:**<br>National binge drinking prevalence was:<br>14.7% for BRFSS and 21.6% for NSDUH<br>Most binge drinkers were male (74% BRFSS, 68% NSDUH) & white, non-Hispanic (73% BRFSS, 76% NSDUH)
**Mohler-Kuo et al., 2015**<br><br>**Switzerland**	**Cohort study** (Young Swiss men from C-SURF)<br>**Population-based**	5943 total sample (100% men)<br>20.0 (18–25) years	DSM-IV & DSM-5 criteria<br><br>QFQs	**AA & AD** (DSM-IV) &<br>**AUD** (≥ 2 criteria-DSM-5) (12-month prevalence)<br>**RSOD** (≥ 6 drinks/single occasion)	**Multinomial logistic regression:**<br>31.7% met DSM-5 AUD (21.2% mild; 10.5% moderate/severe], less than overall DSM-IV criteria for AA & AD (36.8%)

	(Rural, 60.3%; Urban, 39.7%)		RSOD & at-risk volume drinking	**At-risk volume drinking** (≥ 21 drinks/wk & RSOD at least monthly)	Relative to those meeting both DSM-IV & DSM-5 criteria, all other subgroups reported less alcohol and illicit drug use.
**Neumark et al., 2007**<br><br>**Israel**	**Cross-sectional** (Israeli adults) **National population-based survey**	4,859 adults (49.0% men) ? (≥ 21 years)	**WMH-CIDI** (lifetime & past 12-month DSM-IV Dx)	**DSM-IV** (AA & AD) **Frequent drinking:** (3 ormore times in one week at least once) in the past year. **Frequent heavy drinking:** consumption of ≥ 3 drinks, ≥ 3 times a week at least once during past year	**Logistic regression models:** **Lifetime AD** was 41%, **Frequent drinking was 5%, & frequent HD was** (6.8% of men & < 1% of women) Lifetime AA/AD was 4.3% (4.0%, AA & 0.4% AD criteria) Significantly higher rates among males (AOR=7.3), younger adults (AOR=5), immigrants (AOR=2.0) & never married (AOR=1.6)
**Proodfoot and Teeson, 2002**<br><br>**Australia**	**Cross-sectional** (Australian National Survey of Mental Health & Wellbeing, NSMHWB)	10,641 respondents (sex unspecified) ? (≥ 18 years)	**CIDI 2.1** (modified WHO version) **QFQs**	**DSM-IV Diagnosis for AA & AD** **High level of dependence:** ≥ 4 criteria for dependence.	**Multiple logistic regressions:** AD was 4.1% (males 6.1% & females 2.3%) Variables correlated with AD were male sex, young age (18-34); not being in a married or de facto relationship & having any affective, anxiety or other substance use disorder.
**Veerbeek et al., 2019**<br><br>**Netherlands**	**Cohort study** (Data from, NEMESIS-2 **Population-based** (6 categories of urbanicity: very high to very low)	4618 persons (sex unspecified) ? (23–70 years)	**CIDI V 3.0** DSM-IV International guidelines for alcohol use definitions	**Alcohol disorder:** AA &/or AD (past 12 months) **Heavy alcohol use:** > 14 drinks/wk (women) & > 21 drinks/wk for men	**Multinomial logistic regression analyses:** Prevalence of heavy alcohol use was higher in older (55–70 years) than younger people (6.7% vs 3.8%), but alcohol disorder was less prevalent (1.3% vs 3.9%). Heavy alcohol use was associated with higher level of education in older adults compared to younger adults.
**Williamson et al., 2003**<br><br>**United Kingdom**	**Cross-sectional** (Subjects from 26 general practices registered with MRC-GPRF) Community-based project in the UK<br><br>**Secondary data**	**20,062** unrelated index subjects (40.0% men) ? (20–60 years)	**UK definition** for binge or heavy drinking behaviour &<br><br>**QFQs** for (single session drinking criteria)	**Binge/heavy session drinkers**: males > 8 & females > 6 units/session<br><br>**Non (binge/heavy session) drinking:** not fulfilling session drinking criteria, including abstainers	**No statistical analysis performed** Average number of units of alcohol per week consumed was 16 for men and 8 for women. 17% of subjects had binge drinking fashion. (15% for male vs 18% for females) Binge drinking was found to be most prevalent amongst males & females in their 20s (33% of male vs 38% of females).
**Auchincloss et al., 2022**<br><br>**USA (**Philadelphia, Pennsylvania metropolitan area)	**Cross-sectional analyses** (population-based cohort) (Urban setting)	**772 (cross-sectional analyses)** (48% men) ? (21–64 years)	Quantity/ Frequency Questions **(QFQs)** RSOD criteria	**BD** (SAMHSA definition): at least one day in past 30 days the person consumed a high volume of alcohol on a single occasion (≥5 alcoholic drinks for males and ≥ 4 for females).	**Logistic regression and Poisson regression** Among alcohol users in either time period, 22% consumed 8 or more drinks per week and 37% reported at least 1 binge occasion in the past 30 days. higher outlet density was associated with more alcohol consumption and residing farther from an outlet was associated with less alcohol consumption.

**Bott et al., 2005**<br><br>**Germany**<br>(Lübeck city and its catchment area)	**Cross-sectional** (part of a longitudinal study) (urban setting)	4,074 (analysis) (44.9% men) 42.7 (18-64 years)	DSM-IV based Munich CIDI (M-CIDI). **Quantity/ frequency index,QFI** (at-risk drinking = Based on the British Medical Association's, 1995, recommendations)	**Four alcohol-use groups:** (1) moderate drinkers/ abstainers (MOD/A): < 12 times in their lives or <20g/women & <30g/men pure alcohol/day **(2)** at-risk drinkers (ARD): >20/30g pure alcohol/day (3) DSM-IV criteria for alcohol abuse (AA) (4) DSM-IV criteria for alcohol dependence (AD)	**Multinomial regression analysis (multivariate associations):** 9% of participants were at-risk drinkers. Prevalence rates for at-risk drinkers were 16.9% for affective, 18.1% for anxiety and 17.8% for somatoform disorders. Compared with MOD/A, atrisk drinkers showed a 2-fold increased risk of having a psychiatric disorder. Subjects with AA showed a comparable level of risk & with AD showed an even greater risk. Female at-risk drinkers were twice as likely to have a psychiatric disorder compareed to male.
**Britton et al., 2020**<br><br>**United Kingdom**	**Cross-sectional** (part of Whitehall II study, civil servants at phase 11 (2012–13) (urban setting)	6117 (alcohol & sleep data) (70.9% men) Mean age: 69.4 men, 69.6 women (61–81 years)	**Volume of consumption (**drinks used in last 7 days) **Retrospective alcohol life-course grid** (AUDIT-C)	**Hazardous drinking/HD**: ≥ 5 points on AUDIT-C **Non-drinkers**: didn't drink alcohol in past year.	**Logistic regression:** 15.7% of men consumed 21 or more units per week compared to only 2.4% of women. 30.5% men & 12.8% women reported HD. men drinking > 21 units/wk or drinking hazardously were more likely to have disturbed sleep than those not drinking in past week or not drinking hazardously.
**Husberg et al., 2022**<br><br>**Norway (Tromsø)**	**Cross-sectional data** (population-based) (Tromsø 1-7, T7 = 2015-2016 (urban setting)	**19,185 (analysis)** (47.5% men) Mean age: 57.2 women, 57.4 men (40-96 years)	**AUDIT:** Hazardous alcohol use (HAU)	Hazardous alcohol use: AUDIT ≥ 8 as a cut-off	**Logistic binomial regression model:** Insomnia was more prevalent among participants with a HAU (24.1%) than without (18.9%). Participants who had HAU had higher odds of insomnia (OR= 1.49).
**Lee et al., 2020**<br><br>**Singapore**	**Cross-sectional (**Singapore Mental Health Study, SMHS 2016) (urban setting)	**6126 (interviewed)** (50% men) ? (18 yrs & above)	**QFQs** (alcohol use) **CIDI 3.0** (mental disorders) **DSM-IV** (diagnosis of mental disorders)	**Bing Drinking (BD):** consumption of 5 or more drinks (male) or 4 or more drinks (female) on a single occasion in the past 12 months.	**Multiple logistic regressions** 13.7% reported past-year BD (17.6% of males and 9.8% of females). Moderate associations between BD and mood and anxiety disorders (ORadj =1.8–4.4), were noted, while associations with AUDs were much stronger (ORadj=5.3–9.7). Associations betweenn BD & anxiety disorders were observed exclusively in females (ORadj=2.3–3.3). Binge drinkers reported a lower quality of life compared to their non-binging counterparts.

**Lindstrom et al., 2020**<br><br>**Sweden**	**Cross-sectional**	11,716<br>(50.4% men)<br>? (65-99 years)	AUDIT-C (Alcohol consumption)	non-drinker = 0; moderate drinker = 1–7 (male), 1–5 (female); risk-drinker = 8–12 (male), 6–12 (female).<br>Non-drinker was not consumed alcohol during the last 12 months.	**Logistic regression analysis**<br>Men (83%) were more prone to drink alcohol compared to women (71%). The prevalence of risk drinking was about 2% for both genders.<br>Alcohol consumption declined with age. Moderate consumption of alcohol was associated with lower probability of poor SRH compared to non-drinking (AOR=0.64 for men) and (AOR= 0.68 for women).
**Lundin et al., 2021**<br><br>**Sweden** (Gothenburg, second largest city in Sweden)	**Longitudinal** (Women and Alcohol in Gothenburg (WAG) Study, cohort in 1986, 1994/2000 & 2013) (urban setting)	**1,614 (baseline)**<br>(100% women)<br>? (across different age-group?)	CIDI-SAM, ICD-10 & ICD-1, DSM-IV & DSM-5	AUD, alcohol abuse (AA), alcohol dependence (AD) based on CIDI-SAM or (DSM-III, DSM-III-R, DSM-IV, DSM-5, & ICD-10 & ICD-11)	**contingency tables & Cohen's Kappa coefficient (κ)**<br>Baseline: prevalence of lifetime AD was 10.6 % (ICD-11); 4.0 % (ICD-10); 4.3 % (DSM-IV); 7.5 % (DSM-III-R); and 12.3 % (DSM-III). DSM-5 AUD was 14.3 %.
**Mason-Jones and Cabieses, 2015**<br><br>**Chile**	**Cross-sectional**<br><br>(Chilean National Health Survey 2010, ENS 2010) (88% lived in urban settings)	Adolescents (absolute n=435, weighted n = 1860812)<br>Young adults (absolute n = 412, weighted n = 1386 547)<br>(50.3% men)<br>? (adolescents 15-20 years & young adults 21-25 years).	**QFQs**<br>(Alcohol prevalence in last year, & BD prevalence in last month)	**Alcohol prevalence in last year:** 'yes' labeled as "1" and 'no' labeled as "0".<br>**BD prevalence last month:** had drunk four or more units of alcohol in a single episode in the last 4 weeks.	**Conditional logistic regression models:**<br>65% of adolescents and 85% of young adults reported drinking alcohol in the last year &<br>of those (who used alcohol in the last year) 83% of adolescents and 86% of young adults reported BD in the previous month. Adolescents who reported binging alcohol were also more likely, compared to young adults, to report being depressed (OR 12.97) or to feel very anxious in the last month.<br>Adolescent females were more likely to report poor life satisfaction in the previous year (OR 8.50), feel depressed (OR 3.41).<br>Being female was also associated with a self-reported diagnosis of depression for both age groups.
**Mondi et al., 2022**<br><br>**USA (Chicago)**	**Cross-sectioal**<br><br>(CLHS data collection, predominately Black sample) (grew up in urban poverty)	**301** CLHS participants<br>(40% men)<br>? (32-37 years invited to CLHS)	**M.I.N.I. 7.0.2.** (based on DSM-IV & ICD-10 criteria)	DSM-IV & ICD-10 criteria for major depressive disorder, generalized anxiety disorder, post-traumatic stress disorder, substance use disorder, and AUD.	**Independent samples t-tests**<br>Males endorsed significantly higher rates of any AUD within the past 12 months (38.3%) than females (20.6%).<br>Probable prevalence rate for any AUD was 27.7%.

Dagne K, *et al. BMJ Open* 2024; 14:e080657. doi: 10.1136/bmjopen-2023-080657

**O'Dwyer et al., 2019**<br><br>**Ireland**	**Cross-sectional**<br>(Data generated from 2013 National Alcohol Diary Survey, NADS)	**4338 drinkers**<br>(49.9% men)<br>? (18–75 years old)	**RSOD** criteria (HED)<br>**DSM-IV (CIDI)**<br>**Alcohol-related harms/ARH (8 questions)**	**HED:** consuming 60 g or more of pure alcohol in a single drinking occasion.<br>**Alcohol dependence** (AD) (DSM-IV criteria)<br>Current drinkers, non-drinkers, monthly HED, occasional HED, low-risk drinkers, ARH	**Crosstabs (Pearson χ2, bivariate assoc.)**<br>There was a relatively even breakdown of low-risk (31.0%), occasional HED (30.6%), and monthly HED (31.5%) drinkers.<br>AD constituted 6.9% of all drinkers.<br>Overall, 29% of drinkers experienced at least one harm from their own drinking in last year.<br>Respondents who were AD had a greater individual risk of experiencing each harm.
**Shockey and Esser, 2020**<br>**USA** (District of Columbia and territories)	**Cross-sectional**<br><br>(U.S. employed adults who resided in 32 states, BRFSS data)	**358,355 employed adults**<br>(48% men)<br>? (18-55 years)	Industry & occupation (I&O) optional module BRFSS & **QFQs**	**BD**: men consuming ≥ 5 drinks or women consuming ≥ 4 or more drinks, on an occasion.	**No statistical analysis performed.**<br>20.8% reported BD, with an average of nearly 49 times per year and an average intensity of 7.4 drinks per binge episode, resulting in 478 total binge drinks per binge drinker. The adjusted BD prevalence ranged from 15.9% among community and social services workers to 26.3% among construction and extraction workers.

Abrevations: AA: alcohol abuse; **AD**: alcohol dependence; **ARH**: Alcohol Related Harm; **AUD:** Alcohol Use Disorder; **AUDADIS-IV**: Alcohol Abuse and lcoholism's Alcohol Use Disorder and Associated Disabilities Interview Schedule– DSM-IV Version; **AUDIT**: Alcohol Use Disorder Identification Test; **BD**: Binge Drinking; **BRFSS**: Behavioral Risk Factor Surveillance System; **CLHS**: Chicago Longitudinal Health Study; **C-SURF**: Cohort Study on Substance Use Risk Factors; **FFQ**: Food Frequency Questionnaire; **GLM**: General linear models; **GNP**: General Population; **GPRF**: General Practice Research Framework; **HAU:** Heavy alcohol use; **ICD-10/11:** International Classification of Diseases 10th/11th Revision; **MONICA**: MONItoring of trends and determinants of CArdiovascular disease; **NIAAA**: National Institute on Alcohol Abuse and Alcoholism; **NRR**: Non response rate; **wk**: week; **NSDUH**: National Survey on Drug Use and Health; **OHC**: Occupational Health Care clinic; **PHC:** Primary Health Care clinic outpatients; **QFQs**: Quantity Frequency Questionnaires of alcohol use; **RSOD**: Risky Single-Occasion Drinking; **SAMHSA**: Substance Abuse and Mental Health Services Administration; **USA:** United States of America; **yr.:** year; ?: mean age is not mentioned.
