# Supplementary material for: Scoping review of assessment tools for, magnitudes of and factors associated with problem drinking in population-based studies
Source: BMJ Open. 2024 Mar 8;14(3):e080657. doi: 10.1136/bmjopen-2023-080657 (PMC10928735; doi:10.1136/bmjopen-2023-080657)
Supplement: Supplementary data [file bmjopen-2023-080657supp004.pdf]

Supplementary File 4

Table: Prevalence, associated factors, and pattern of problem drinking in low-and middle-income countries (LMICs), 2023.

Author, Year<br><br>Country/Location	Study Design & Study Setting<br><br>(population)	Participants: Sample size (Male, %)<br>Mean age (range) in years	Tools (Measures) or questions used	Outcomes: (Definition/nature of use)	Results & statistical methods used:
**Andersson et al., 2018**<br><br>**South Africa**<br>(Eastern Cape Province)	**Cross-sectional**<br>(Nelson Mandela Metropolitan & Sundays River Valley City)<br>**Population-based**<br>(Urban/semi-urban/ rural setting)	1000 participants<br>(52% of men)<br>27 (18-40) years	**M.I.N.I. 6.0 (DSM-IV)**	**Alcohol dependence & Alcohol abuse (AD/AA):**<br>(DSM-IV diagnosis during the past 12 months)	**$\chi 2$ statistics & logistic regression models:**<br>AD: 26.5% (39.0% men & 19.1% women)<br>AA: 9% (19.0% for men & 6.0% for women).<br>**AD:** higher in rural/semi-rural in men (43.1%) and women (26.8%) than in urban/semi-urban.<br>Widowed and separated women compared to married or cohabiting and women with low income (don't want to disclose) compared to weekly household income of ≥ 1,001 RAND remained statistically significant.
**Burazeri and Kark, 2010**<br><br>**Albania**<br>(Tirana)	**Cross-sectional**<br>(transitional post-communist Albania (Muslim, 68.5%)<br>**Population-based**	685 individuals<br>(65.7% of men)<br>52.6 (35–74) years	Quantity/ frequency questionnaires **(QFQ**s)<br>(patterns questions)<br>(12 months)	**Drunkenness/hangovers:** never, very exceptionally, 2-3 times/year, 1/month, 1/fortnight & once/week).<br>Composite Binging score: drunkenness or hangovers during w/c ≥3 units (≈60g of ethanol) consumed	**Binary/multivariable logistic regression:**<br>10.3% of men had ≥ 2-3 annual episodes of drunkenness & and hangovers each.<br>**Women**: both markers of binging, 1.4%<br>**Men:** 8.9% drinking ≥ 60g alcohol/session.<br>Binge drinking was related to low educational level, financial loss in pyramid collapse, & religiosity (inversely) in both Muslims and Christians (all in men).
**Dias da Costa et al., 2004**<br><br>**Brazil**<br>(Rio Grande do Sul State)	**Cross-sectional**<br>(Adults of municipality of Pelotas)<br>**Population-based**<br>(Urban area)	2,177 adults (43%)<br>41.6 (20-69) years	**QFQs**<br>(weekly use)	**Moderate consumption:** up to 30g/day of ethanol)<br>Heavy consumption or hazardous drinking, HD: ≥ 30g/day of ethanol/week	**Non-conditional logistic regression:**<br>Moderate consumption was 65.1%<br>**HD:** 14.3% (29.2%, men & 3.7% in women).<br>Men, elders, blacks, low SES, heavy smokers, & chronic disease presented higher prevalence of HD. Men with minor psychiatric disorders had higher prevalence of HD & in women (association between age & HD was inversely related).

Dagne K, *et al. BMJ Open* 2024; 14:e080657. doi: 10.1136/bmjopen-2023-080657

**Ji et al., 2018**<br><br>**China** (Xuzhou city, Jiangsu)	**Cross-sectional**<br>(11 regions in Xuzhou city)<br>**Population-based**<br>(urban/rural areas)	36,157 participants<br>(48.40% of men)<br>45.5 (18-75) years	**MAST**	**Alcohol dependent (AD):**<br>MAST score of ≥ 5<br>0 (no alcohol dependence)<br>1–4 (low AD), 5–6 (light AD), 7–25 (mild AD), 26–39 (moderate AD & 40–53 (severe AD)	**χ2 &, t-tests; multivariate log. Regression:**<br>**AD:** 11.56% (22%, males & 1.74%, females)<br>Newly detected hypertension rate was 9.46%<br>Significant associations were found between AD & blood pressure.<br>AD was positively correlated with systolic blood pressure & diastolic blood pressure (r = 0.077, P< 0.01).
**Mendoza-Sassi and Beria, 2003**<br><br>**Brazil**	**Cross-sectional**<br>(Residents in municipality of Rio Grande, Southern Brazil)<br>**Population-based**<br>(Urban population)	1260 people<br>(46.1% of men)<br>40.3 (15-94) years	**AUDIT**<br><br>**SRQ-20**	**Alcohol Use Disorder (AUD):** AUDIT score ≥ 8	**Log. regression in multivariate analysis:**<br>**AUDs:**7.9% (2.5%, women & 14.5%, men).<br>Risk of alcohol misuse increased across increasing social class (P linear trend = 0.03)<br>Males had OR=6.89 compared with women.<br>Smokers (OR 3.27) & ex-smokers (OR 1.30) were at higher risk than non-smokers.<br>Those with minor psychiatric disorders had a 2.48 OR of presenting a positive test (AUD).
**Moreira et al., 1996**<br><br>**Brazil**<br>(Porto Alegre)	**Cross-sectional**<br>(Adult population of Porto Alegre, Southern Brazil)<br>**Population-based**<br>(Urban)	1,091 individuals<br>(45.0% of men)<br>Mean age: 41/men; 44/women<br>(≥ 18) years	**CAGE** &<br>Type & **QFQs** of alcoholic drink	**Heavy drinking (HD):**<br>Average of ≥ 30g/day<br>**Alcohol dependence/AD:**<br>Two positive answers to the CAGE questionnaire	**X^2-test & logistic regression models:**<br>**AD** was 9.3%; **heavy drinking** was 15.5%.<br>Increasing age, lower education & income, non-white race (associated with HD & AD).<br>Households with 3-4 persons were associated with lowest risk HD, but AD was higher in crowded households (5-11). Presence of one with HD/AD in household was associated with HD but not with AD.
**Peltzer et al., 2011**<br><br>**South Africa**	**Cross-sectional**<br>(Part of SABSSM 2008 survey)<br>(62.5% located in urban areas)	13,828 persons<br>(43.7% of men)<br>? (≥ 15 years)	**AUDIT**	**Binge drinking (BD):**<br>Females (4) & males (5) standard drinks/occasion<br>**Hazardous or harmful drinking:** AUDIT cut-off score ≥ 8	**Adjusted logistic regression:**<br>**Risky (hazardous/harmful drinking):** 9% (17% among men & 2.9% for women)<br>Overall prevalence of BD: 9.6%<br>**Men:** risky drinking was associated with 20-54 years than 15-19; Colored population group; lower (economic status & education.)<br>**Women:** risky drinking was associated with urban residence, Colored population group; lower education; and higher income
**Peltzer and Phaswana-Mafuya, 2013**<br><br>**South Africa**	**Cross-sectional**<br>(older South Africans, Study of Global Ageing &	2144 participants<br>(41.1% of men)<br>? (> 60 years old)	**QFQs** &<br>**NIAAA** risky drinking criteria	**Risky drinking (2 ways):**<br>**Heavy drinkers:**<br>(>7 drinks per week) &<br>**Binge drinkers:**	**Multivariate logistic regression:**<br>**Heavy & binge drinking**: 4% vs 3.7%<br>Male gender, white population group; tobacco use & being obese were associated with risky drinking.

	Adults Health, SAGE in 2008) **Population-based** (Urban, 63.2%)			(>3 drinks/one occasion at least weekly)	Hypertension, diabetes, and depression were not associated
**Peltzer et al., 2012**<br><br>**South Africa**	**Cross-sectional** (South African Youths, Black, 97.5%; 4 of 9 provinces in SA) **Population-based**	3123 participants (54.6% of men) 20.5 (18-24) years	**AUDIT-C** (Frequency of drinking, quantity consumed per occasion & frequency of HED)	**HED:** consumption of five standard drinks (≥ 60g) alcohol per single occasion **Binge drinking:** women (4) & men (5) units in a session at least/month **Hazardous or harmful drinking (HHD):** ≥ 5 on AUDIT-C	**Unconditional multivariable log. Reg.:** **HHD:** 19.1% (24.3%, male; 12.9%, women) **Men:** high sexually permissive attitudes, not poor, multiple sexual partners, tobacco & illicit drug use were associated with HHD. **Women:** high (HIV risk perception, sexually permissive attitudes & peer pressure (lifestyle), spending more nights away in a week, tobacco & illicit drug use were associated with HHD.
**Tomkins et al., 2007**<br><br>**Russia** (Izhevsk)	**Cross-sectional** (Men controls in a case-control study of premature male mortality, Izhevsk) **Population-based** (Urban)	1750 men (100% men) ? (25-54 years)	**QFQs**	**Hazardous drinking-HD:** (any of these in past year) Having drunk surrogates; having been on zapoi; having frequent hangovers (once/month or more); having drunk spirits daily.	**Logistic regression:** Drinking spirits (79%) & surrogates (8%) at least sometimes in the past year. Drinking spirits (25%) & surrogates (4%) at least weekly & 10% had had episode of zapoi in past year. Education, lowest level in men (associated with indicators of HD. Indicators HD were also associated with being unemployed & levels of household wealth/amenities.
**Weiser et al., 2006**<br><br>**Botswana**	**Cross-sectional** (5 districts of Botswana with highest number of HIV-infected individuals) **Population-based** (Urban/Rural)	1,268 adults (48% men) 28.8 (18-49 years)	**QFQs**	**Heavy alcohol consumption (HD):** > 14 drinks/wk for women, & > 21 drinks/wk for men) **Problem drinking** (8–14, women, 15–21 for men) &	**Heavy drinking:** 31%, men & 17%, women **Problem drinking:** 39% of men, (79% met HD) & 25 % of women, (69% met HD). Correlates of HD: intergenerational relationships (age gap 10 year), male gender, higher education, & living with a sexual partner. A dose-response relationship was seen between alcohol use & risky sexual behaviors, with moderate drinkers at lower risk than both problem & heavy drinkers.
**Zavos et al., 2015**<br><br>**Sri Lanka** (Colombo district)	**Cross-sectional** (Data from the Colombo Twin And Singleton Study, CoTASS) **Population-based** (Urban/semi-urban areas)	6014 Sample (twins/48% & Singleton/46% of male) Mean age: 34 (twins) & 43 (singleton) (> 16 years)	**CIDI** Alcohol use: ever had of 12 drinks at any time in life	**Alcohol abuse & dependence:** Definition of CIDI (DSM-IV criteria)	**Robust cluster command:** **12-month prevalence of alcohol use:** 22.7% **Lifetime AA & AD in men:** 6.2% & 4.0% Lifetime AA & AD was associated with greater prevalence of nicotine dependence, depression, anxiety & PTSD (only for AD). Lower standard of living was associated with alcohol use & AD but not with AA

**Lo et al., 2013**<br><br>**Kenya**<br>(Nyanza Province)	**Prospective study**<br>(Longitudinal database of demographic & health census data in western Kenya)<br>**Population-based**<br>(Rural area)<br>Secondary data	72,292 individuals<br>(43.1% men)<br>? (≥ 18 years)	Questions on (ever use & current use)	**1) % of time drunk when drinking in past 30 days:** (Did not get drunk, Drunk < 50%, Drunk 50%+)<br>**2) Days drinking/month:** (1-7, 8-17 & 18+)<br>**3) Problem drinking:** drinking ≥ 8 days/past 30 days & were drunk at least 50% of times they drank	**Crude and adjusted logistic regression:**<br>Overall, ever drinking was 20.7%<br>Drinking/past 30 days was 7.3% & 34.6%.<br>(60.3%, being drunk on ≥ 50%+) of all drinking occasions)<br>Alcohol use increased with decreasing socio-economic status & oldest women.<br>Current smoking, men, all age groups ≥ 40 & highest wealth index quintile (significantly associated with problem drinking).
**Pillai et al., 2013**<br><br>**India**<br>(Northern Goa)	**Cross-sectional**<br><br>**Population-based survey**<br>(rural & urban communities)	2641 men<br>(100% men)<br>? (18-49 years)	**QFQs** & Drunkenness	**Current drinkers:**<br>**low risk** (< 40 g/d),<br>**medium risk** (40–60 g/d), & high risk (> 60 g/d)<br>**HED**: ≥ 60g in a single occasion in past 12 months<br>**Drunkenness**: times drank to feel drunk in last 1 year (< monthly, ≥ monthly but < weekly), & ≥ weekly)	**Logistic regression + Moderating effect:**<br>Of current drinkers:<br>**HED:** 28.6 % (rural 31 %; urban 27.2 %) & Drunkenness: 33.7% (rural 30.5 %; urban 35.5 %) ➔ monthly or more frequent<br>**HED**: associated with older age, being separated, lower education, & LSI<br>Weekly or more frequent drunkenness was associated only with rural residence.<br>All three risky drinking patterns were associated with CMDs, sexual risk, intimate partner violence, acute alcohol-related consequences, & AD.
**Sau, 2017**<br><br>**India**<br>(West Bengal)	**Cross-sectional**<br>(Adult population of the state of West Bengal, Gram Panchayat, GP)<br>**Community-based**	99 adults<br>(54.5% men)<br>38.62 (≥ 18) years	**AUDIT**	**AUDIT (WHO scoring):**<br>≥ 8 (hazardous/harmful use & possible AD)<br>0-7 (Zone-I): Low risk drinking/abstinence risk<br>8-15 (Zone-II): Alcohol use in excess of low-risk,<br>16-19 (Zone-III): Harmful & hazardous drinking &<br>20-40 (Zone-IV): Alcohol dependence risk level.	**Intraclass correlation, chi-square test, logistic regression & Bootstrapping:**<br>**Mean AUDIT** score was 7.11 (5.55 to 8.74)<br>**Low risk drinking/abstinence:** 65.5% & **Alcohol use in excess of low risk:** 17.6%, & **Harmful & hazardous drinking:** 8.5% & **Alcohol dependence** was 8.4%<br>**Hazardous, harmful use & AD** was 34.5%<br>Male gender and being employed were more prone to become high risk level drinker.
**Takahashi et al., 2017**<br><br>**Kenya**<br>(Western)	**Cross-sectional**<br>(Adults residing in Ikolomani Sub-county, Kakamega)<br>**Community-based**	478 participants<br>(41.4% men)<br>41 (18–65) years	**AUDIT**<br><br>**Type** & **QFQs**	**Current drinkers:** use of any alcohol in the last month,<br>Hazardous/high-risk drinkers:	**Univariate & multivariate analyses:**<br>Current & hazardous/high-risk alcohol use: 31.7% (men 54.6%; 8.9%, women) vs 28.7%<br>More than one drinker in the family, ≥ 5 drinker friends & positive attitude towards alcohol intake were positively associated with

	(Rural)			AUDIT score of ≥ 8	current alcohol drinking status, and with hazardous/high-risk alcohol consumption.<br>Women were less likely to be current drinkers & hazardous/high-risk drinkers.
**Yeung et al., 2015**<br><br>**Cambodia**<br>(Puok district)	**Mixed methods**<br>(Adults living in 2 selected rural communities<br>**Community-based**<br>Rural communities	120 households<br>(49.0% men)<br>? (≥ 18 years)	**AUDIT-C-Q**<br>**QFQs**<br>8 FGDs<br>**NIAAA**<br>Guidelines	**AUD:** cut off score of ≥ 5 in men & ≥ 4 in women<br>HED: ≥ 6 drinks in a single sitting at least monthly (NIAAA)	**χ2, Welch 2-sample t-test, Log. Regression**<br>**AUD & HED:** 4% and 31%, respectively.<br>**AUD** (47% men, 5% women (P < 0.0001); **HED** (47% men, 15% women (P = 0 .0001).<br>Male sex, younger age (decreasing age), and increasing income (higher monthly) were significant risk factors for AUD and HED
**Alem et al., 1999**<br><br>**Ethiopia**<br>(Butajira)	**Cross-sectional**<br>(Demographic surveillance site)<br>**Community-based**<br>(mostly rural)	12531 residents<br>(50% male)<br>? (≥ 15 years)	5-item questionnaire (questions for alcohol user vs non-users & **GAGE-4** items)	**Problem drinking (PD):** consumption beyond safe limits (≥ 2 positive responses on CAGE).<br>Cigarettes smoked daily: 1-3=mild,<br>4-9=moderate,<br>>9= heavy	**Chi-square statistics:**<br>Current drinkers: 23.4 % (15% women & 36% for men).<br>PD, 15.7% in alcohol users; overall PD, 3.7% (7.5% men & 0.90% women).<br>(2.4% in urban dwellers & 4.0% in rural)<br>Christian religion, male sex, ethnically non-Gurage, & smoking (associated with PD in both sexes). Marital status (divorced men), mental distress & income were associated with PD only in men & being widowed & divorced in women
**Kebede and Alem, 1999**<br><br>**Ethiopia**<br>(Addis Ababa)	**Cross-sectional**<br>Adults in Addis Ababa<br>**Population-based**<br>(Urban residents)	10203 adults<br>(45.1% men)<br>? (≥ 15 years)	**CAGE**<br>(1st stage) &<br><br>**CIDI**<br>(2nd stage)	**Problem drinking (PD):** ≥ 2 of on CAGE items, & **Alcohol dependence (AD):** CIDI (ICD-10 diagnoses)	**Bivariate and multivariate analysis:**<br>**PD** was 2.7%, lifetime AD, 1.0% (1.9% in male & 0.1% for women) & one-month AD, 0.8% (1.5% for men and 0.06% for women).<br>PD increased with increasing age<br>PD decreased with increasing educational attainment. 39% increased risk of PD with employment & female sex had a 96% decreased risk of PD. Only sex (women had an 84% less risk to be AD compared to men).
**Nalwadda et al., 2018**<br><br>**Uganda**<br>(Kamuli District)	**Cross-sectional**<br>(Men attending PHC & men in population; part of the PRIME project)<br>**Community-based** & facility-based<br>(Rural district)	351 men<br>(Community study)<br>778 men<br>(Facility Survey)<br>(100% men)<br>? (≥ 18 years)	**AUDIT**<br>(10 item)	**AUD definition (AUDIT): Hazardous** (score 8–15), **Harmful** (score 16–19) or **Dependent** (score ≥ 20) drinking behaviors<br>(cut-offs defined by WHO)	**Kruskal–Wallis test & Fisher's exact test:**<br>**Community study**: 4.1% of all men were AUDIT+ (AUD); (2.9% hazardous, 0.7% harmful & 0.5% with dependent drinking)<br>**Facility study:** 5.7% of all men were AUDIT+; (4.5% hazardous; 0.6%, harmful)<br>47.5% AUDIT+ men: AUD ruined their lives<br>55.0% AUDIT+ men did not seek treatment<br>AUDIT scores were higher among older men, men with paid/self-employment status and higher PHQ-9 score (P < 0.05).

**Rathod et al., 2018**<br><br>**Nepal**<br>(Central district)	**Cross-sectional**<br>(Adults in Chitwan District; part of PRIME consortium)<br>**Population-based**<br>Secondary analysis	3482 sample<br>(36% men)<br>? (18-88) years	**AUDIT**<br>(10-item)	**Abstinent:** Score of 0,<br>**Recent (12 months) consumer:** Score of ≥ 1<br>Score of ≥ 8: **positive screen for AUD,**<br>8–15: **hazardous** drinking,<br>16-19: **harmful** drinking &<br>≥ 20: **dependent drinking**	**X^2 test & Negative binomial regression:**<br>23.8% of male screened AUD+ (AUD)<br>5.3% of female drinkers screened AUD+<br>Men with AUD, 38% spoke to another person about their problems & 80% had internalized stigma.<br>Being a drinker was associated with age, religion, caste, education, occupation & tobacco use. AUDIT scores were associated with age, caste, marital status, occupation, tobacco use, depression, functional status & suicidal ideation.
**Teferra et al., 2016**<br><br>**Ethiopia**<br>(Sodo district, southern Ethiopia)	**Cross-sectional**<br>(Adults from rural Sodo district (PRIME survey)<br>**Community-based**<br>(Rural residents)	1500 adults<br>(50.5% men)<br>? (≥ 18 years)	**FAST**<br>**Kessler-10**<br>(psychological distress)<br>**LTE** (adverse life events)	**Hazardous alcohol use (HD):**<br>FAST score ≥ 3 out of 16	**Exploratory multivariable log. regression:**<br>Prevalence of **hazardous alcohol use**: 21%; (31% in males & 10.4 % in females)<br>Factors associated with HD were being male, increasing age, having experienced ≥ 1 stressful/adverse life events, & severe psychological distress (AOR = 2.96).<br>High social support was protective from hazardous alcohol use (AOR = 0.41)
**Zewdu et al., 2019**<br><br>**Ethiopia**<br>(South, Sodo district)	**Cross-sectional**<br>(Adults who lived for at least 6 months in Sodo dist)<br>**Community-based**<br>(Rural district)	1485 individuals<br>(45.7% men)<br>39 (≥ 18) years	**AUDIT-10**	**Probable AUD:** score ≥ 8<br>8–15 (medium level of alcohol problem)<br>≥ 16 (high level of alcohol problems)<br>≥ 20 (possible alcohol dependence-AD)<br>**Binge drinking (BD):** drinking ≥ 6 alcoholic drinks on a single occasion	**Poisson regression with robust variance:**<br>Weighted prevalence of AUD was 13.9%; 25.8% in men & 2.4% women, P < 0.001<br>(Hazardous/harmful/AD: 9.9%/2.2%/1.8%)<br>23.3% had BD<br>87.0% of cases scored ≥ 16 had never sought help & 70.0% had high internalized stigma<br>AUD were associated & more prevalent in men (aPR = 7.7), farmers, traders, & daily laborers. People with AUD had increased total depressive symptom score & higher total disability score, more stressful life events & suicidal ideation (aPR 1.5)
**Getachew et al., 2017**<br><br>**Ethiopia**	**Cross-sectional**<br>(2015 national noncommunicable diseases STEPS survey)<br>**Community-based**<br>(Urban,27.4% & rural, 72.58%)	9,800 participants<br>(40.6% men)<br>34.5 (15-69) years	**QFQs**<br>(WHO STEPS questionnaire)	**Current drinkers**: alcohol use a month before survey<br>Lifetime alcohol use: ever<br>**Past 12-month users:**<br>HED/Excessive Alcohol Consumption: drinking ≥ 6 drinks in men & ≥ 4 in women on one occasion.	**Logistic regression:**<br>Prevalence of lifetime alcohol consumption & current drinkers was 49.3% & 40.7%.<br>Among ever drinkers, 89.6% drank alcohol in the past 12-months.<br>**HED:** 12.4% (20.5% males & 2.7% females)<br>Factors independently associated with HED, were male sex, rural residence), married, and current tobacco smoking (AOR=2.87).

**Abd Rashid et al., 2021**<br><br>**Malaysia**<br>(Sabah Borneo Island)	**Cross-sectional**<br>(People in Bingkor who consumed alcohol in the past 12 months)<br>(urban setting)	**363 participants**<br>(51.5% men)<br>? (≥ 26 years old, 90.6%)	**AUDIT**<br>(hazardous alcohol use)<br>**MINI V5.0** based on DSM-IV (psychiatric morbidity)	**Hazardous alcohol use:**<br>AUDIT scores of ≥8	**Multiple logistic regression analysis**<br>80.2% admitted having consumed alcohol.<br>Preferred type of drink: beer (67.8%), tuak tapai (61.7%), wine (31.7%), tuak beras and whisky (16.8%), imported alcohol drinks such as vodka (9.1%) and 'samsu' (3.9%).<br>41% of participants (high risk for hazardous alcohol use) vs 39.1% (with low risk of hazardous alcohol use).<br>Being male & being a non- Muslim had a higher risk to develop hazardous alcohol use (OR = 3.313 & 3.834 respectively).<br>Having a current obsessive- compulsive disorder was associated with a higher risk of hazardous alcohol use (OR = 0.265).
**Assanangkornchai et al., 2020**<br><br>**Thailand**	**Cross-sectional**<br><br>(Thailand's 5th National Health Examination Survey, NHES-5, 2014)<br>(urban/53.6%, rural/46.4%)	**13177 participants**<br>(49.2% men)<br>46.7 (> 20 years)	**AUDIT**<br>(for AUD)<br>**MINI, Thai version 5.0.0**<br>(for MDE)	**AUD**: non-problem drinkers (0–7), hazardous drinkers (8–15), and harmful-dependent drinkers (16– 40) on AUDIT<br>**MDE**: defined according to DSM-IV criteria	**Multinomial logistic regression:**<br>10.3% and 1.9% hazardous drinkers and harmful-dependent drinkers, respectively<br>2.5% met the criteria for MDE in the past 12 months before the survey.<br>Approximately 20% were current smokers.<br>Associations between MDE and either hazardous (HD) or harmful dependent drinking (HDD) were strongest among those in third tercile (highest/wealthiest) of wealth index, first tercile (lowest/poorst), secondary school level of education or above, living in urban areas, & those who are employed.
**Ding et al., 2020**<br><br>**China**	**Cross-sectional**<br>(China Health and Retirement Longitudinal Study, 2011– 2012)<br>**Community based**<br>(Urban/40.5%, Rural/59.5%)	**17,302 subjects**<br>(49.30% men)<br>59.67 (aged ≥ 45 years)	**QFQ**<br>(for alcohol use)	**Heavy drinking:** >14 drinks per week (males) & >7 drinks per week for females	**Binary & multinomial logistic regressions**<br>Overall prevalence of heavy drinking, obesity, current smoking, and physical inactivity were 7.23%, 11.53%, 27.46%, and 44.06%, respectively.<br>Compared with healthy subjects (no hypertension, high cholesterol, or diabetes), newly detected hypertensive patients were more likely to smoke (OR, 1.34), be heavy drinkers (1.45), and be obese (1.94).
**Hernandez-Vasquez et al., 2022**<br><br>**Peru**	**Cross-sectional**<br>[(2018 Peruvian Demographic & Family Health Survey (ENDES)]	**32,020 people (analysis)**<br>(42.8% men)<br>? (≥ 18 years old)	**SAMHSA definition (RSOD):** Bing Drinking (BD)	**BD:** consumption of 5 & 4 or more alcoholic beverages on the same occasion for men & and women, respectively, in the	**Poisson's family GLMs with link function (log) were used for (cPR and aPR).**<br>BD was found in 22.4%. Men (32.6%) presented a higher consumption pattern than women (12.8%).<br>Men aged 25–44 had a higher probability of BD (aPR: 1.28). The age group of ≥ 60 was associated with a lower probability (aPR:

	**A Population-Based Analysis** (Urban/65.7%, Rural/34.5%)			last 30 days before the survey	0.70) of BD compared to younger group of men (18-24 years). Women aged ≥ 60 years was associated with a lower probability of BD (aPR: 0.24). Secondary (aPR: 2.01) or higher level of education (aPR: 2.04) was a factor associated with a higher prevalence of BD in men
**Jadnanansing et al., 2021**<br><br>**Suriname**	**Cross-sectional** [(populations in both region (rural/Nickerie & urban/Paramaribo)]	**2863 participants** (43% men)<br>39.97 years (?)	**AUDIT & ASSIST:** (for AUD)	**Risky alcohol use:** A score of > 7 on AUDIT	**Simple & Multivariable logistic regression**<br>AUD is 6.4% in urban area & 5.8% in rural area. Men had highest addiction risk at about 16% compared with 2% for females.<br>A treatment gap of 50% was found for AUDs in the rural area (64% urban area).<br>Married persons are significantly less likely to become alcoholic than singles and other groups in urban area.<br>In both areas, higher education was associated with a lower probability of alcohol abuse and dependence, while handymen showed a higher odd.
**Jirapramukpitak et al., 2008**<br><br>**Thailand** (Bangkok)	**Cross-sectional** (Suburban community of Bangkok in 2003 and 2004)	**1052 residents** (46.3%)<br>? (16–25 years	**AUDIT** (hazardou or harmful drinking) & **DIS** (illicit drug use-Diagnostic Interview Schedule)	**Illicit drug use:** assessed with self-report adapted from (DIS) and **Hazardous/harmful drinking:** with AUDIT **Migration**: the occasion when a young person born in amore rural area moves for the first time into Greater Bangkok.	**Multivariate analysis (logistic regression)**<br>10.9% (82 males and 17 females) had illicit drug use and 24.3% (179 males and 62 females) hazardous and harmful drinking.<br>Hazardous/harmful drinking was associated independently with being late migrants, who moved at the age of 15 or older.
**Moreira et al., 1998**<br><br>**Brazil**	**Cross-sectional** (Adults in Porto Alegre, a city in southern Brazil)	**1099 individuals** (45% men)<br>? (18-88 years old)	**QFQs** (type, quantity, & frequen**cy) & CAGE questionnaire**	**Heavy drinking**: average consumption of 30g/day or more, a level of exposure associated with health risks **Dependence:** Two positive answers to the CAGE questionnaire	**Simple/multiple linear & logstic regression**<br>24.1% had never drunk alcohol (9.0%/men & 36.5%/women). 29.3% of men & 4.2% of women were heavy drinkers. 16% & 4.0% were CAGE+, respectively.<br>Consumption of 30 g/day ethanol was associated with increases of 1.5 & 2.3 mmHg in DBP & SBP for men and 2.1 and 3.2 mmHg for women respectively.<br>Prevalence of HTN was higher among those ingesting ≥ 30 g/day (odds ratio = 2.9).
**Oancea et al., 2021**	**Cross-sectional**	**59,399 individuals** (47.6% men)	**NIAAA definitions**	**BD (NIAAA)**: a pattern of drinking that brings BAC	**Weighted & adjusted logisitic reggression**

**Brazil**	(2013 Brazilian National Health Survey)	weighted median age, 40.53 (18-60+ years)	(Binge drinking/BD & Heavy drinking/HD)	levels to at least 0.08 g/dl. (4 drinks for women & 5 for men in about 2hrs)<br>**HD**: ≥ 5 days of BD episodes in a month is defined as the HD index.	14.8% were current smokers, 13.8% were binge drinkers & 3.2% were heavy drinkers.<br>Self-reported current depression/SRCD,7.6%<br>There was significant weighted & adjusted increase in the odds of SRCD among young adults (18–39 years) who were binge drinkers compared to those who were not binge drinkers (AOR = 1.32).
**Pengpid et al., 2021**<br><br>**South Africa**	**Cross-sectional**<br>(National survey of all household members, who resided in that household the previous night)<br>(Rural informal/ 26%, Rural farms/ 5%, Urban/69%)	**39,210 persons**<br>(48.3% men)<br>Median age,34 (IQR,25-48)<br>(15 years & older)	**AUDIT**<br>(Hazardous, harmful, or dependent alcohol use (HHDA):<br>**ASSIST** (Drug use in the past 3 months)<br>**K10** (Kessler Psychological Distress Scale)	**HHDA:**<br>Adults (≥ 20 yrs): cut-off score is ≥ 8 on AUDIT & Adolescents (15–19 years): 5 or more on AUDIT<br>**Drug use in past 3 months**: Any drug used in past 3 months was coded as 1 and never as 0'.<br>**Psychological distress:** scores ≥ 20 on (K10)	**Unadjusted & adjusted logisric regression**<br>10.3% engaged in HHDA, 16.5% (males) & 4.6% (females). Past 3-month drug use was 8.6%, 13.3% (males) & 4.1% (females).<br>Men of middle age (25-34) with higher education, urban residence, drug use and psychological distress were positively associated with HHDA. Women of middle age (25-34) and mixed race, residing on rural farms and urban areas, drug use and psychological distress were positively associated & older age (≥55) & Indians or Asians were negatively associated with HHDA.
**Prais et al., 2008**<br><br>**Brazil**<br>(Metropolitan area of Belo Horizonte, & Bambuí)	**Cross-sectional**<br>(elderly Brazilian men, ≥ 60 years were the study population)<br>**Population based**<br>(urbann setting)	**685 residents in RMBH & 642 in Bambuí**<br>(100% men)<br>**Mean age:**<br>68.8 yrs (RMBH)<br>69.0 yrs (Bambuí)<br>(≥ 60 years)	**RSOD criteria**<br>(for BD)	**Binge Drinking:**<br>Consumption of five or more alcoholic drinks on a single occasion in the last 30 days.	**Multivariate analyses (PR estimated by Robust Poisson Regression)**<br>Prevalence of BD was two times higher among residents in metropolitan area of Belo Horizonte (27.1%) than in Bambuí (13.7%).<br>**RMBH:** higher schooling level [8+ yrs] (PR = 1.55), worse self-rated health [reasonable, bad, or very bad] (PR = 0.62) and inability to perform activities of daily living (PR = 0.12) remained significantly associated with BD.<br>**Bambuí**: worse self-rated health (PR = 0.57) and being divorced or separated (PR = 2.49) remained significantly associated with BD.
**Trangenstein et al., 2018**<br><br>**South Africa**<br>(Tshwane Metropole)	**Cross-sectional**<br>(Adults who used alcohol in the past six months).<br>(Data from South African arm of the multi-country International	**713 adults**<br>(65.8% men)<br>36.3 (18-65 years)	**International Alcohol Control (IAC) questionnair:**<br>(Asks QFQs over past six months)	**Heavy Drinking (HD):**<br>consuming ≥ 96g of absolute alcohol (AA) (roughly 8 standard drinks, or 120 ml) for men or ≥ 72g (6 standard drinks, or 90 ml) for women at least monthly.	**Multivariate logistic regression**<br>HD was 53%. HD did not vary by gender (F1, 19 = 3.96, p = 0.06), age, race/ethnicity, or total annual personal income. Bivariate analyses revealed that HD differed by marital status (F2.48, 47.11 =3.09, p = 0.04).<br>Adjusting for marital status & primary container size, single persons were found to have substantially higher odds of HD.

	Alcohol Control, IAC study) (urban setting)			**Low risk**: occasions that did not include HD	
**Vellios and Van Walbeek, 2018**<br><br>**South Africa**	**Cross-sectional** (data from wave 4 of the 2014-2015 National Income Dynamics Study, NIDS) (rural/35.4%, urban/64.6%)	**22,752 (wave 4)** (46.8% men) ? (≥ 15 years)	**QFQs:**1) How often do you drink alcohol? 2) On a day you have an alcoholic drink, how many standard drinks do you usually have?	**Binge drinker:** use of ≥5 standard drinks on an average drinking day.<br>**Current drinker**: any option from (iii) I drink alcohol very rarely, (iv) Less than once a week, (v) On 1 or 2 days a week, (vi) On 3 or 4 days a week, (vii) On 5 or 6 days a week, & (viii) Every day.	**Multiple logit regressions**<br>Current alcohol use (any amount) in 2014 - 2015 was reported by 33.1% of the population (47.7% males, 20.2% females). Of current drinkers, 43.0% reported BD (48.2% males, 32.4% females).<br>Self-reported BD as a proportion of the total population was 14.1% (22.8% M, 6.4% F).<br>Self-reported BD was highest among males & females aged 25-34 years (49.4%).<br>Smoking cigarettes for both genders substantially increased the likelihood of drinking any amount (aOR: 5.08 males, 4.80 females) and of BD (aOR: 1.53 for males, 3.36 for females).<br>As a percentage of total population, people aged 25-34 years were more likely to binge than aged 15-24 years, for both males (OR 1.44) and females (OR 1.49). Compared with married males, males living with a partner (OR 1.58) or who were single (OR 1.74) were more likely to BD.<br>Compared with married females, females living with a partner (OR 1.68) or single (OR 1.41) were more likely to BD.<br>Having children in the house slightly increased the probability of BD for males (OR 1.21), but not for females.
**Aremu et al., 2021**<br><br>**Nigeria**<br>(urban poor people in Ibadan)	**Cross-sectional** (two selected urban poor communities in Ibadan, Nigeria)	**500 Participants** (29.4% men) 35.36 (18-65 years)	**Modified version of WHO STEPS instrument**	**Alcohol consumers:**<br>Ever consumed,<br>Current consumers (12mo.)<br>Current & **frequent consumers within 30 days (low, medium, and high)**<br>**Low consumers:** consuming < 4 (men) & < 2 (women) SDs/occasion<br>**Medium:** 4-6 (men) & 2-4 (women) SDs per occasion<br>**High:** > 6 (men) & > 4 (women) SDs per occasion	**Descriptive & inferential statistics (X^2)**<br>29.0% had consumed alcohol either in past or present, 17.8% consumed alcohol within last one year, 15.8% were current consumer of alcohol & 13.6% were frequent consumers who had taken alcohol within 30 days (11.6% low consumers, 1.2% medium consumers and 0.8% high consumers).<br>More male (53.1%) reported to have ever consumed alcohol compared to female (46.9%). 62.3% of non-current alcohol users was female & 37.7% were male. 59.3% of respondents not currently consuming alcohol were currently married (30.3% were not).<br>74.1% of the low consumers were male, 66.7% medium consumers were females, & 75.0% of high alcohol consumers were male

**Bonnechère et al., 2022**<br><br>**Burkina Faso**	**Cross- sectional** (Data from the 2013 Burkina Faso WHO STEPwise) Rural (75.1%), Urban (24.9%) **Population- based**	**4692 individuals** (45.7% men) ? (25–64 years)	Quantity/Frequency Questions **(QFQs)**	**4 levels of consumption:** No consumption (None) **Low**: intake of pure alcohol of <40g/day (men) & <20g for women **Mid**: 40-59.9g/day (men) & 20-39.9g for women **Abusive consumption:** ≥60g/day (M) & ≥40g (W) **Dependent variable:** mean alcohol consumption in the last 30 days.	**Multinomial logistic regression:** 3559 (75.8%) were not consuming any alcohol, 12.9% had low, 8.5% had mid and 2.7% had abusive alcohol consumption. Age was associated with any level of alcohol consumption with a gradient effect and older people having a higher level of consumption in comparison with no consumption. Tobacco consumption was significantly associated with alcohol intake with gradient effect, those with higher tobacco use being at higher risk of abusive alcohol intake. Sex is an important risk factor for abusive consumption with increased risk for men compared with women. Jobless people & housemaker was associated with a decreased risk of having abusive consumption.
**Dahal et al., 2021**<br><br>**Nepal** (Kathmandu district)	**Cross-sectional** (adults residing in municipalities of Kathmandu district for at least six months) **Community-based** (unplanned urbanization)	**245 participants** (47.3% men) **Mean age:** 41.19/male, & 40.91/female (18–69 years)	**WHO STEPS questionnaire (QFQs)**	**Current episodic heavy drinking (HED):** six or more drinks on any day in the past 30 days.	**Bivariate & multivariate analysis** 67.3% were lifetime abstainers. Prevalence of alcohol consumption in last 12 months was 31.0% & HED was 12.7%. Prevalence of current smoking, low intake of fruits & vegetables and low physical activity was found to be 22%, 93.9% and 10.2% respectively. 52.2% of participants were overweight/obese & prevalence of raised BP was 27.8%. Odds of alcohol consumption were higher among male (AOR: 2.78), employed (AOR: 2.30), & those who belonged to Chhetri (AOR: 2.83), Janajati (AOR: 6.18), Dalit and Madhesi, (AOR: 7.51) ethnic groups.
**Jonas et al., 2014**<br><br>**India** (rural Central India)	**Cross-sectional** (data from Central India Eye and Medical Study, CIEMS, in rural region of Central Maharashtra) **Population-based**	**4711(participated)** (46.5% men) 49.5 (30+ years)	AUDIT CESD 20-item FTND (smoking behavior)	**Harmful or hazardous drinking:** sum score of 8 or more on AUDIT **Clinical episode of major depression:** score of > 21 in the CES-D.	**Test of for association not performed** Alcohol consumption was 23.0%; 6.0% subjects had an AUDIT score ≥8 (hazardous drinking), & 4.63% subjects a score ≥ 13 (women) or ≥ 15 (men) (alcohol dependence)
**Olickal et al., 2021**	**Cross-sectional**	**316 adult men** (100% men)	**WHO AUDIT**	**Hazardous alcohol**: AUDIT score of 8–15	**Independent t-test, One-way ANOVA & Kruskal Wallis test, Multiple linear regression**

**India**<br>(Puducherry, South India)	(adult men aged above 18 years in Puducherry, South India)<br>**Community-based**<br>(rural/50%, urban/50%)	45.2 (≥18 years)	**WHO QoL-BREF questionnaire**	**Harmful alcohol use**: AUDIT score of 16–19<br>**Probable alcohol dependence**: score of 20 or more on AUDIT<br>**High risk:** A score eight and above on AUDIT<br>**QoL:** A higher score is indicative of a better QoL in each of the domains.	Mean (SD) AUDIT score was 13.2 (6.7).<br>Probable dependence was 8.2%, & hazardous or harmful use was 27.8%. Overall mean score of QoL was lower among alcohol users compared to non-alcohol users (50.7 vs 63.5)<br>QoL score was significantly lower among alcohol users (also in all domains).<br>High-risk alcohol users and urban residence had 11.2 & 4.1 less QoL scores respectively and educated had 7 more QoL scores compared to the reference category.
**Olickal et al., 2022**<br><br>**India**<br>(Puducherry, South India)	**Cross-sectional & Qualitative design**<br>(Mixed design)<br><br>(All men ≥ 18 years from urban & rural field practice areas of a tertiary care centre in Puducherry, South India)	**316 subjects**<br>(100% men)<br>45.2 (19-60+ years)	**WHO AUDIT**<br>Discussion guide for FGD	**Probable alcohol dependence:** A total score of ≥20 on AUDIT	**A log binomial regression (prevalence ratio) & Manual content analysis**<br>Alcohol use was 38%, 40% were daily users)<br>(34% in rural to 42% in urban areas)<br>Among alcohol users, 21.7% were probable dependents on alcohol.<br>Older individuals had a 2.9 times higher risk of alcohol use than young individuals (<30).<br>No formal education was a high-risk factor for alcohol use, compared to educated.<br>Individuals residing in rural areas (APR = 1.05), self-reported comorbidities (APR = 1.21), family history of alcohol use (APR = 2.42) and tobacco use (APR = 2.42) were significantly associated with alcohol use.
**Sarma et al., 2019**<br><br>**India**<br>(Kerala, South India)	**Cross-sectional**<br>[(all individuals between 18-69 years old were eligible, in both rural & urban (49.3%) areas)]<br>**Community-based**	**12,012 adults**<br>(37% men)<br>42.5 (18–69 years)	**WHO STEPS instrument GPAQ**<br>(Global Physical Activity Questionnaire)<br>**Anthropometric measurements**	**Current alcohol use:** intake of at least one standard drink of alcohol in the past 30 days.<br>**Current tobacco use**: use of any form of tobacco within the past 30 days.<br>**Raised Blood Pressure (BP):**<br>BP of ≥140/≥90 mm Hg, or if the person is currently using antihypertensive medication.	**Weighted means, Percentages with 95% CI, & variance inflation applied**<br>Current use of tobacco & alcohol in men was 20.3% & 28.9% respectively.<br>The overall prevalence of raised BP was 30.4%.

**Endashaw Hareru et al., 2022**<br><br>**Ethiopia**<br>(Dilla town)	**Cross-sectional**<br>(Residents of Dilla town, Gedeo zone, Southern Ethiopia with age of ≥ 18 years)<br>**Community-based**	**666 participants**<br>(70% men)<br>Mean: 33.3 years<br>(≥ 18 years)	**AIDIT:** AUD<br>**Kessler Psychological Distress Scale (K10):**<br>**ASSIST 2.0:** current and lifetime substance use	**AUD:** AUDIT score of ≥ 8	**Bivariate & multivariate binary logistic regression analysis**<br>AUD during the past year was 30.6%.<br>Being male (AOR = 8.33), age of less than 33 years old (AOR = 1.78), current cigarette smoking (AOR = 2.49), current khat chewing (AOR = 6.23), high level of psychological distress (AOR = 7.69) and poor social support (AOR = 2.30) were significantly associated with AUD.
**Gutema et al., 2020**<br><br>**Ethiopia**<br>(Arba Minch HDSS)	**Cross-sectional**<br>(Adult residents of Arba Minch HDSS (nine Kebeles of Arba Minch Zuria District, Southern Ethiopia)<br>**Community-based**<br>(rural residents, 83.7%)	**3346 participants**<br>(50% men)<br>44.6 years<br>(25– 64 years)	**WHO STEPS instruments**<br>(alcohol use)<br>**SRQ-20**<br>(mental stress status)	**HED or Excessive Alcohol Consumption:** use of ≥ 6 drinks for men and ≥ 4 drinks for women on a single occasion at least once per month.<br>**Mental stress** (mild, moderate, and severe)	**Binary logistic regression**<br>Prevalence (HED) was 13.7%.<br>HED was associated with occupation (daily laborer: AOR 0.49; & housewives: AOR0.63 compared with farmers), wealth index (2nd quintiles: AOR 0.55 & 3rd quintiles: AOR 0.66) compared with 1st quintiles; & climatic zone (midland: AOR 1.80; highland: AOR 1.95 compared with lowland).<br>Tobacco use (AOR 4.28), & khat use (AOR 4.75) were also associated with HED.
**Legas et al., 2021**<br><br>**Ethiopia**<br>(South Gondar)	**Cross-sectional**<br>(adult residents whose age was 18 years and above in the South Gondar zone, 61.3% from urban areas)<br>**Community-based**	**848 (interviewed)**<br>(62.3% men)<br>? (≥ 18 years)	**AUDIT**-AUD<br>**PHQ-9**<br>**PSS-**Perceived stress scale questionnaire)<br>**Oslo social support scale**<br>**SPIN**-Social phobia inventory scale	**AUD**: score of 8 or above on AUDIT<br>**Depression:** A score of five or more on the PHQ-9	**Bivariate & multivariable logistic analysis**<br>AUD over the last 12-months was 23.7%.<br>16.50% had hazardous alcohol use, 5.2% had harmful alcohol use, and 2% had probable alcohol dependence.<br>Being male (AOR = 4.34), poor social support (AOR = 1.95), social phobia (AOR = 1.69), perceived high level of stress (AOR = 2.85), current cigarette smoking (AOR = 3.06) and comorbid depression (AOR = 1.81) were significantly associated with AUD.
**Wainberg et al., 2018**<br><br>**Mozambique**<br>(Zambézia Province)	**Cross-sectional**<br>(2014 survey)<br>(16 year or older female heads-of-household in Mozambique, Zambézia	**2,752 participants**<br>(no men, 100% female)<br>Median: 27 years<br>(16-62 years)	**AUDIT**<br>(Alcohol use)<br>**PHQ**<br>(Depression)	**Hazardous, harmful & high-risk drinkers:**<br>AUDIT scores > 4 (recommended cutoff for women)<br>**Depression:** A score of ≥ 10 on PHQ-8 (associated with clinical depression)	**Binomial logistic regression model:**<br>Overall prevalence of current alcohol consumption among female heads of hh was 15%. "hazardous drinkers" was 8%.<br>A positive depression screening (aOR: 2.20), death of a child (aOR: 2.44), & currently being pregnant (1.83) were associated with increased odds of hazardous drinking.<br>Being single (aOR: 0.48) & experiencing food insecurity (aOR:0.96) were associated with reduced odds of risky drinking.

	Province)<br>**Population-based**<br>/rural Mozambique				
**Bete et al., 2022**<br><br>**Ethiopia**<br>(Harari regional state)	**Cross-sectional**<br>(residents aged >18 years)<br>**Community-based**<br>(80.55% urban dwellers)	**955 adults**<br>(44.18% men)<br>42.28 years<br>(> 18 years)	**ASSIST**	**Current and ever substance users:** use of a specified substance (for non-medical purposes) in last 3 months and once in lifetime respectively	**Bi-variable & multivariate binary logistic regressions:**<br>The overall prevalence of **current alcohol use was** (8.24%) , tobacco use (14.5%), and khat use (63.30%).<br>The availability of alcohol, being unemployed, and being a current khat user were significantly associated with current alcohol use.
**Castelo Branco and de Vargas, 2023**<br><br>**Brazil**<br>(Northern Brazilian Amazon, state of Amapa)	**Cross-sectional**<br>(Karipunan respondents aged ≥ 15)<br>**Population-based**	**230 participants**<br>(51.3%, men)<br>? (≥ 15)	**AUDIT**	**hazardous/harmful alcohol use (Zones II-IV of AUDIT Score, problematic use):**<br>AUDIT score > 8.	**Fisher's exact test, & logistic regression: simple & multiple**<br>(Hosmer-Lemeshow test/C statistic, & Spearman correlation tests)<br>Prevalence of alcohol use: 70%; 59.6% (low-risk use), **38.3 (hazardous/harmful alcohol use)**, **& 2.2% (probable alcohol dependence). Overall, 40.5% had hazardous or harmful alcohol use**; **66.6%** were men, and **33.4%** were women.<br>Being male sex (AOR: 3.30), being Catholic (5.53) compared to Evangelical were associated with hazardous or harmful alcohol use.
**Rezaei et al., 2022**<br><br>**Iran**<br><br>**(national survey)**	**Cross-sectional**<br>(The STEPs survey in Iran, 2016)<br>**Population-based**<br>(urban residents, 71.09%)	**29,068** participants<br>(47.92%, men)<br>44.4 years (18 to 100 years)	**WHO's guidelines**<br>(WHO STEPS instrument)	**Current alcohol consumption:** drink alcohol in past 12 months<br>**Lifetime consumption:** ever drink alcohol in life.	**Univariate and multiple logistic regression analysis**<br>National level prevalence rates of lifetime and current alcohol consumption were 8.00% and 4.04% respectively.<br>The highest prevalence was reported among 25-34 years old.<br>Individuals of higher socioeconomic status consumed significantly greater levels of alcohol.<br>Current alcohol drinkers were 2 times more prone to trafic injury as compared to nondrinkers (ORadj: 2.0).
**Tegegne et al., 2023**<br><br>**Ethiopia (national survey)**	**Cross-sectional**<br>(2016 Ethiopian Demographic and Health Surveys (EDHS)<br>**Population-based**<br>(80.29%, rural)	**12,688** participants<br>(100%, male)<br>30.92 years<br>(15-59 years)	**QFQs**	**Ever alcohol drinking:** drinks alcohol during the lifetime.	**Multilevel multinomial logistic regression**<br>Only Khat users (22.0%), only **Alcohol users (35.6%)**, and dual Alcohol and Khat users were (9.0%).<br>At the individual level: age group of 30-44 years (AOR: 1.75) and 45-59 years (AOR:1.62) are more likely to drink alcohol compared to 15-29 years.<br>Higher educational level (AOR: 1.4) compared to no education and having occupation (AOR:1.88) compared to people without occupation, increased the odds of drinking alcohol.<br>Divorced males (AOR: 0.5) compared to single males; Protestant (AOR: 0.01), Muslim (AOR: 0.04), and other religion follower

					males (AOR: 0.35) compared to Orthodox religion have lower likelihood of alcohol drinking.
**Wolde, 2023**<br><br>**Ethiopia**<br>(South West Ethiopia)	**Cross-sectional**<br>(elderly people living in towns in Ethiopia)<br>**Community-based**	**382 elderly people**<br>(34.5%, male)<br>67 years<br>(≥ 60 years)	**AUDIT**<br>**ASSIST**	**Alcohol Use Disorder (AUD):** AUDIT score > 8.	**Bivariate and multivariable logistic regression model**<br>Magnitude of **AUD**, current alcohol use, and life-time alcohol use was **27.5%**, 52.4%, and 89.3%, respectively.<br>AUD was associated with cognitive impairment (AOR: 2.53), poor sleep quality (AOR: 2.67), chronic medical illness (AOR: 3.27), and suicidal ideation or attempt (AOR: 2.07).

Abrevations: **AA**: Alcohol Abuse; **AD**: Alcohol Dependence; **aPR**: adjusted Prevalence Ratio; **ASSIST**: Alcohol, Smoking, and Substance Involvement Screening Test; **AUD**: Alcohol Use Disorder; **AUDIT**: Alcohol Use Disorder Identification Test; **BD:** Binge drinking**; CAGE**: Cut down, Annoyed, Guilty feeling & Eye opener; **CESD:** Center for Epidemiologic Studies Depression Scale; **FAST**: Fast Alcohol Screening Test; **FTND:** Fagerstrom Test for Nicotine Dependence; **HD**: Heavy drinking; **HED**: Heavy Episodic Drinking; **wk**: week; **M:** men; **MDE:** Major Depressive Episode; **NIAAA**: National Institute on Alcohol Abuse and Alcoholism; **PHQ-9:** Patient Health Questionnaire-9 item; **PR:** Prevalence Ratio; **PRIME**: Programme for Improving Mental Healthcare; **QFQs**: quantity/frequency questionnaires; **QoL:** Quality of Life; **RMBH:** metropolitan region of Belo Horizonte; **RR:** response rate**; SD:** Standard drink; **W:** women; **yr.**: year; **?:** mean age or age range for subjects is not determined.
