# Supplementary material for: Scoping review of assessment tools for, magnitudes of and factors associated with problem drinking in population-based studies
Source: BMJ Open. 2024 Mar 8;14(3):e080657. doi: 10.1136/bmjopen-2023-080657 (PMC10928735; doi:10.1136/bmjopen-2023-080657)
Supplement: Supplementary data [file bmjopen-2023-080657supp005.pdf]

Supplementary File 5

Newcastle-Ottawa Scale (NOS) quality assessment summary for the study "A scoping review of assessment tools for, magnitudes of, and factors associated with problem drinking in population-based studies," 2023.

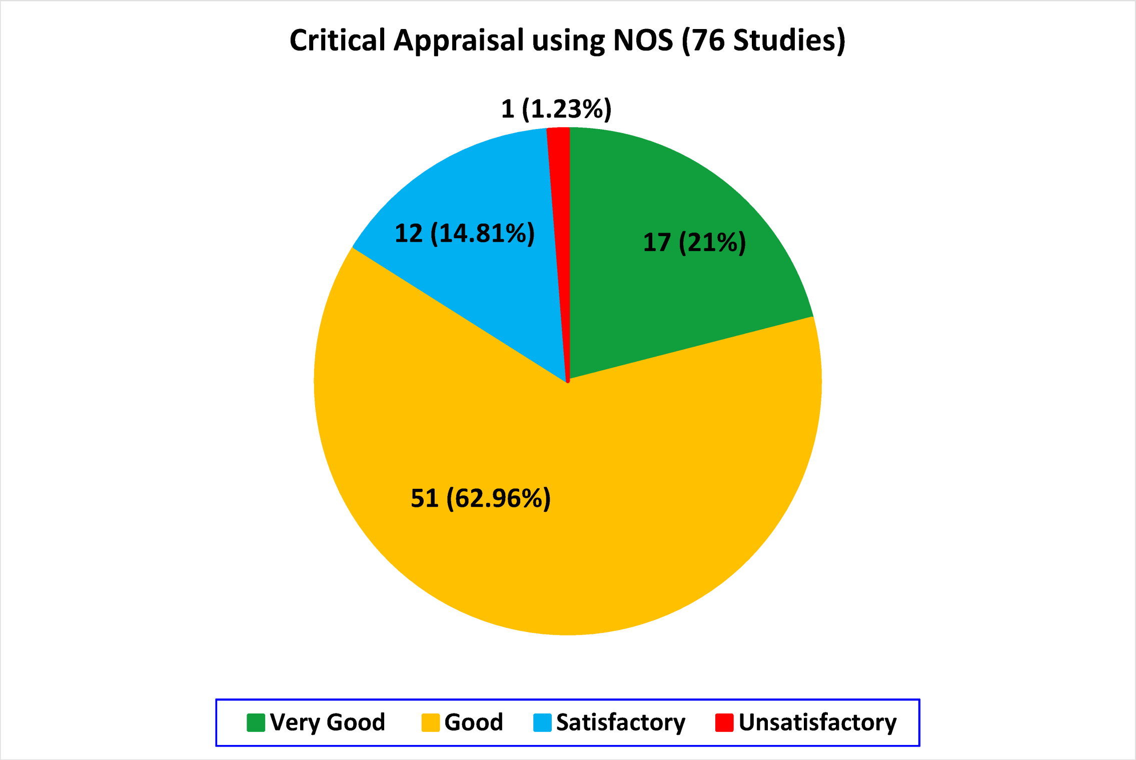

Figure: Newcastle-Ottawa Scale (NOS) quality assessment reports of studies for the study "A scoping review of assessment tools for, magnitudes of, and factors associated with problem drinking in population-based studies," 2023.

**Table: Newcastle-Ottawa Scale (NOS) quality assessment- item level summary for "A scoping review of assessment tools for, magnitudes of, and factors associated with problem drinking in population-based studies," 2023.**	**Studies (81)**
**Selection: (Maximum 5 points/scores/stars)**	
**1. Representativeness of the sample:**	
a. Truly representative of the average in the target population. * (all subjects/consecutive or random sampling)	**69**
b. Somewhat representative of the average in the target group. * (non-random sampling)	**12**
c. Selected group of users/convenience sample.	**0**
d. No description of the derivation of the included subjects (sampling strategy).	**0**
**2. Sample size:**	
a. Justified and satisfactory (including sample size calculation). * (1 score)	**44**
b. Not justified	**23**
c. No information provided	**14**
**3. Non-respondents:**	
a. Proportion of target sample recruited attains pre-specified target or basic summary of non-respondent characteristics in sampling frame recorded. *	**72**
b. Unsatisfactory recruitment rate, no summary data on non-respondents.	**02**
c. No information provided	**07**
**4. Ascertainment of the exposure (risk factor/disease) or screening/surveillance (measuremnt) tool:**	
a. Secure record (medical charts) or validated measurement (screening/surveillance) tool. **	**28**
b. Non-validated measurement tool, but the tool is available or described or Self report. *	**52**
c. No description of the measurement tool.	**01**
**Comparability: (Maximum 2 stars)**	

**1. Comparability of subjects in different outcome groups on the basis of design or analysis. Confounding factors controlled.**	
a. Data/results adjusted for relevant predictors/risk factors/confounders e.g., age, sex, marital status, job etc. **	**68**
b. Data/results not adjusted for all relevant confounders/risk factors/information not provided.	**13**
**Outcome: (Maximum 3 stars)**	
**1. Assessment of outcome:**	
a. Independent blind (structured) assessment. **	**14**
b. Record linkage. **	**0**
c. Self report. *	**67**
d. No description.	**0**
**2. Statistical test:**	
a.Statistical test used to analyse the data clearly described, appropriate and measures of association presented including confidence intervals and probability level (p value). *	**74**
b.Statistical test not appropriate, not described, or incomplete.	**07**
