# Supplementary material for: Scoping review of assessment tools for, magnitudes of and factors associated with problem drinking in population-based studies
Source: BMJ Open. 2024 Mar 8;14(3):e080657. doi: 10.1136/bmjopen-2023-080657 (PMC10928735; doi:10.1136/bmjopen-2023-080657)
Supplement: Author's manuscript [file bmjopen-2023-080657.draft_revisions.pdf]

BMJ Open

BMJ Open is committed to open peer review. As part of this commitment we make the peer review history of every article we publish publicly available.

When an article is published we post the peer reviewers' comments and the authors' responses online. We also post the versions of the paper that were used during peer review. These are the versions that the peer review comments apply to.

The versions of the paper that follow are the versions that were submitted during the peer review process. They are not the versions of record or the final published versions. They should not be cited or distributed as the published version of this manuscript.

BMJ Open is an open access journal and the full, final, typeset and author-corrected version of record of the manuscript is available on our site with no access controls, subscription charges or pay-per-view fees (http://bmjopen.bmj.com).

If you have any questions on BMJ Open's open peer review process please email info.bmjopen@bmj.com

BMJ Open

A scoping review of assessment tools for, magnitudes of, and factors associated with problem alcohol use in population-based studies

Journal:	*BMJ Open*
Manuscript ID	bmjopen-2023-080657
Article Type:	Original research
Date Submitted by the Author:	06-Oct-2023
Complete List of Authors:	Gizachew, Kefyalew Dagne; Addis Ababa University, Department of Psychiatry, School of Medicine, College of Health Sciences; Debre Berhan University, Department of Psychiatry, College of Health Sciences and Medicine<br>Myers, Bronwyn; Curtin University, Curtin enAble Institute, Faculty of Health Sciences; South African Medical Research Council, Alcohol, Tobacco and Other Drug research Institute<br>Awoke, Mihretu; Addis Ababa University, Department of Psychiatry, School of Medicine, College of Health Sciences<br>Teferra, Solomon; Addis Ababa University, Department of Psychiatry, School of Medicine, College of Health Sciences
Keywords:	Systematic Review, Ethanol, AUDIT, EPIDEMIOLOGIC STUDIES, MENTAL HEALTH, Substance misuse < PSYCHIATRY

SCHOLARONE™
Manuscripts

BMJ

I, the Submitting Author has the right to grant and does grant on behalf of all authors of the Work (as defined in the below author licence), an exclusive licence and/or a non-exclusive licence for contributions from authors who are: i) UK Crown employees; ii) where BMJ has agreed a CC-BY licence shall apply, and/or iii) in accordance with the terms applicable for US Federal Government officers or employees acting as part of their official duties; on a worldwide, perpetual, irrevocable, royalty-free basis to BMJ Publishing Group Ltd ("BMJ") its licensees and where the relevant Journal is co-owned by BMJ to the co-owners of the Journal, to publish the Work in this journal and any other BMJ products and to exploit all rights, as set out in our licence.

The Submitting Author accepts and understands that any supply made under these terms is made by BMJ to the Submitting Author unless you are acting as an employee on behalf of your employer or a postgraduate student of an affiliated institution which is paying any applicable article publishing charge ("APC") for Open Access articles. Where the Submitting Author wishes to make the Work available on an Open Access basis (and intends to pay the relevant APC), the terms of reuse of such Open Access shall be governed by a Creative Commons licence – details of these licences and which Creative Commons licence will apply to this Work are set out in our licence referred to above.

Other than as permitted in any relevant BMJ Author's Self Archiving Policies, I confirm this Work has not been accepted for publication elsewhere, is not being considered for publication elsewhere and does not duplicate material already published. I confirm all authors consent to publication of this Work and authorise the granting of this licence.

For peer review only

A scoping review of assessment tools for, magnitudes of, and factors associated with problem alcohol use in population-based studies

Kefyalew Dagne Gizachew [1, 2], Bronwyn Myers [3,4,5], Awoke Mihretu [1] & Solomon Teferra [1]

[1] Department of Psychiatry, School of Medicine, College of Health Sciences, Addis Ababa University, Addis Ababa, Ethiopia

[2] Department of Psychiatry, College of Health Sciences and Medicine, Debre Berhan University, Debre Berhan, Ethiopia

[3] Curtin enAble Institute, Faculty of Health Sciences, Curtin University, Kent Street, Bentley, WA 6102, Australia

[4] Alcohol, Tobacco and Other Drug Research Unit, South African Medical Research Council, Cape Town, South Africa.

[5] Division of Addiction Psychiatry, Department of Psychiatry and Mental Health, University of Cape Town, Cape Town, South Africa

***Corresponding Author:** Kefyalew Dagne Gizachew

Tel: +251910487276; P.O. Box: 9086; E-mail: kdgc08@yahoo.com

ABSTRACT

Background: The term "problem drinking" includes a spectrum of alcohol problems ranging from heavy/excessive drinking to alcohol use disorder. Problem drinking is a leading risk factor for death and disability globally. It has been measured and conceptualized in different ways- making it difficult to identify common risk factors for problem alcohol use. This scoping review aims to synthesize what is known about the assessment of problem drinking, its magnitude, and associated factors.

Methods: Four databases (PubMed, EMBASE, PsycINFO, Global Index Medicus/GIM) and Google Scholar were searched from inception to July 16, 2022. Eligibility criteria were limited to people aged 15 and above, population-based studies reporting problem alcohol use, and English-language articles. This review was reported based on guidelines from the "Preferred Reporting Items for Systematic Reviews and Meta-

For peer review only - http://bmjopen.bmj.com/site/about/guidelines.xhtml

Analyses extension for Scoping Reviews (PRISMA-ScR) Checklist." Critical appraisal was done using the Newcastle-Ottawa Scale (NOS).

Results: From the 12,263 records identified, 9037 underwent title/abstract screening, of which 317 full-text articles were assessed, and 76 articles were included for data extraction. Assessment tools included self-report quantity/frequency questionnaires, criteria to determine risky single occasion drinking, validated screening tools, or structured clinical and diagnostic interviews. The most widely used screening tool was the Alcohol Use Disorder Identification Test. Studies defined problem drinking in various ways, including excessive/heavy drinking, binge drinking, alcohol use disorder, alcohol abuse, and dependence. Across studies, the prevalence of heavy drinking ranged from < 1.0% to 53%, binge drinking from 2.7% to 48.2%, alcohol abuse from 4% to 19.0%, alcohol dependence from 0.06% to 39%, and alcohol use disorder from 2% to 47%. Factors associated with problem drinking varied across studies. These factors included socio-demographic and economic factors like age, sex, relationship status, education, employment, income level, religion, race, location, alcohol outlet density, clinical factors like medical problems, mental disorders, substance use, and quality of life.

Conclusions: Due to differences in measurement, study designs, and assessed risk factors, there was a wide variability in the prevalence of problem drinking and associated factors across studies and settings. The alcohol field would benefit from measuring alcohol use in a harmonised way to allow for comparisons to be made across countries and for meta-analyses.

Scoping Review Registration: Open Science Framework (https://osf.io/9syv7, or https://doi.org/10.17605/OSF.IO/9SYV7)

Keywords: Scoping review, Alcohol, Alcohol use disorder, Problem drinking, Heavy drinking, Binge drinking, Heavy episodic drinking, Alcohol use assessment

For peer review only

ARTICLE SUMMARY

Strengths and limitations of this study

- ► To the authors' knowledge, no other scoping review covers global settings on problem drinking (PD) to map and aggregate findings and offer an overview of the alcohol use disorder (AUD).
- ► Strengths also comprised an extensive search of four databases, including 76 original articles for synthesis.
- ► We included only community-based studies; studies conducted at institutions like hospitals, primary health care centers (PHC), addiction centers, and colleges or universities were not included.
- ► Meta-analysis was not performed due to heterogeneity of methods and presentation of results for included studies.

For peer review only

For peer review only - http://bmjopen.bmj.com/site/about/guidelines.xhtml

INTRODUCTION

The nature of alcohol use, related issues, and how they manifest throughout life have long been the subject of scientific research (1). In 2016, the "Global Burden of Disease Study" identified alcohol use as a leading risk factor for death and disability, and it was ranked seventh among the top risk factors for disability-adjusted life years (DALYs) and deaths globally (2, 3). Previous studies have implicated alcohol in more than 200 injuries and diseases, including alcohol use disorder, liver cirrhosis, malignancies, injuries, tuberculosis, HIV/AIDS (4, 5), noncommunicable diseases (NCDs) (6), mental disorders (7), violence-related harms and injuries (8). These problems can result from acute episodes of alcohol intoxication or chronic heavy alcohol use (9). Although alcohol consumption occurs on a continuum, our understanding of when to intervene and risk factors to target in interventions is hampered by differences in how problem alcohol use has been conceptualized and measured and the lack of synthesized evidence on factors associated with problem alcohol use.

The phrase "alcohol use disorder" (AUD) describes the complete range of alcohol abuse (AA), including binge drinking (BD), risky drinking, harmful drinking behaviors, and alcohol dependence (AD) (10). AUD varies and can range from less severe problems such as heavy, hazardous, or harmful drinking to more serious disorders like AA or AD. Many challenges in understanding the nature and extent of alcohol-related problems, including all spectrums of AUD, arise from different definitions of problematic alcohol use and inconsistent ways of measuring it. In this review, we use the term "problem drinking (PD)" to refer to any problem with alcohol use, including AUD. Different definitions and terms for problem alcohol use (11-26) are summarised in (Table 1).

Table 1: Different definitions and terms for problem alcohol use in the study, 2023.

Terms	Definitions
**Problem Drinking (PD)**	Problem drinking (PD), commonly referred to as "alcohol abuse," "alcohol misuse," or "alcohol use disorder," is a pattern of alcohol intake that harms one's health or relationships with others. It is a general term that covers a range of alcohol-related problems, from mild to severe. Even though PD does

For peer review only

For peer review only - http://bmjopen.bmj.com/site/about/guidelines.xhtml

	not necessarily fulfill the diagnostic criteria for AUD, it can negatively impact a person's life (11-16).
**Hazardous drinking**	A quantity or pattern of alcohol intake puts individuals at risk for adverse health events, which carry the possibility of physical or psychological harm (17, 18).
**Harmful drinking**	Alcohol intake, which causes physical or psychological harm, or the presence of physical or psychological complications, defines it (17, 19).
**Low-risk drinking**	A daily intake of no more than 20g of alcohol with at least two non-drinking days weekly is different for males and females, i.e., not more than three and two drinks a day on average, respectively (20).
**Heavy episodic/binge drinking (HED/BD)**	It is defined as the intake of five or more drinks for men and four or more drinks for women per occasion in most studies (roughly 60 grams of pure alcohol) which brings blood alcohol concentration (BAC) levels to 0.08 gram/dL in about two hours (21).
**Exessive/Heavy drinking (HD)**	HD is the quantity of alcohol consumed that exceeds a set threshold. It is often defined as the weekly use of more than 14 drinks on average for males and more than seven drinks for females. Some countries define it as the average number of binge episodes per person during 30 days or weekly drinking of more than 21 drinks for males and more than 14 drinks for females (21-24).
**Alcohol dependence (AD)**	Based on the Diagnostic and Statistical Manual of Mental Disorders- 4th edition (DSM-IV), AD is characterized by a problematic pattern of alcohol use that results in clinically significant impairment or distress. It is also a symptom of continuing to use alcohol despite knowing that continued use will cause serious social or interpersonal problems (for example, violent arguments with their spouse while intoxicated

For peer review only

	or abusing children) (25).
**Alcohol abuse (AA)**	AA is a pattern of alcohol intake that has adverse outcomes and harms a person's physical health, mental health, interpersonal connections, and general functioning. AA involves excessive and frequent alcohol consumption despite its harmful effects. It can be less severe than AD because it requires fewer symptoms and can only be diagnosed once the DSM-IV criteria have determined that AD is not present (25).
**Alcohol use disorder (AUD)**	AUD is a chronic medical disorder defined by an individual's compulsive and problematic pattern of alcohol consumption, diagnosed when an individual's alcohol consumption leads to significant distress or impairment in their daily functioning. It is characterized by a cluster of behavioral and physical symptoms, including withdrawal, tolerance, and craving, based on the Diagnostic and Statistical Manual of Mental Disorders-5th edition (DSM-5) (11, 26).

Problem drinking, including any AUD, is a critical public health issue that has an impact on people and communities all around the world. A comprehensive review of PD-related information serves several essential purposes. First, it offers crucial epidemiological data, such as burden or prevalence rates, trends, and patterns of PD over time. With this information, public policymakers, researchers, and healthcare workers may more accurately understand the scope of the problem, pinpoint individuals at high risk, and more effectively allocate resources to PD prevention and treatment. Second, the information obtained from the review may be utilized to create awareness of PD and develop policy initiatives on screening and treatment strategies to reduce its prevalence. Third, studying PD data enables a clearer understanding of factors related to the development and progression of PD. This information will guide prevention initiatives and treatments focusing on specific risk factors, such as the environment, clinical variables, and comorbid mental health problems. As such, this review aims to identify the range of community-based screening or measurement tools for PD and to

For peer review only

synthesize the global nature and extent of PD and related problems among the general population.

METHODS

This scoping review was reported based on guidelines from the "Preferred Reporting Items for Systematic Reviews and Meta-Analyses extension for Scoping Reviews (PRISMA-ScR) Checklist," a tool that is used to guide the scoping review process (27).

Eligibility criteria

For this review, only articles written in the English language were considered. The PICO framework for prevalence studies (Population, Measurement of presence of disease, Design, and Setting) guided the choice of eligibility criteria. Accordingly, for studies to be included, they had to (a) study people aged 15 years or older (Population); (b) report problem drinking (PD) or alcohol use disorder (AUD) using any screening scales, measures, instruments, clinical diagnostic interviews or laboratory tests to detect alcohol use (Measurement of the presence of disease); (c) have any epidemiological, population-based design (Design); and (d) be located in any country or type of setting, as long as the study had a community-based sample (Setting).

Information sources

The literature search included four databases: PubMed, EMBASE, PsycINFO, and Global Index Medicus (GIM). Additional records were identified through other sources such as Google Scholar or forward and backward citation searches of included studies. Databases were searched from database inception to July 16, 2022. To ensure methodological rigor, a scoping review protocol for the review was registered with Open Science Framework (OSF), which can be accessed using the associated project ID (registration number) of (https://osf.io/2anj3).

Search Criteria

The PI (KD) developed the search strategy with close consultations with supervisors (ST and BM). The search strategy consisted of key terms, free texts, and controlled vocabulary search terms such as (Medical Subject Heading/MeSH terms for MEDLINE and Emtree terms for Embase) for the main big terms of "prevalence," "alcohol," and "community/population-based health surveys." Terms within each set were grouped using Boolean "OR" operators, and terms across sets were combined using "AND"

For peer review only - http://bmjopen.bmj.com/site/about/guidelines.xhtml

operators. Terms related to alcohol use and the search strategy for searched databases are included in (Supplementary File 1).

Selection of sources of evidence

After the databases were searched, the titles and abstracts of identified records were imported into EndNote software for deduplication and to facilitate the review process. Two reviewers (KD and AM) separately completed screening article titles and abstracts in the first stage and screening full-text articles in the second stage using a priori inclusion and exclusion criteria to determine eligibility. These two reviewers met to resolve screening and selection differences and to reach a consensus on whether to include an article.

Data charting process

We developed a data extraction form that included items relating to study characteristics (author, year of publication and citation, study country/location), study design, study setting and population, sample size, study tools or measures, and results. Two reviewers (KD and AM) independently extracted data from included studies using this form. These reviewers met to resolve data extraction differences and to reach a consensus on what to extract from the included articles.

Collating, summarising, and reporting the results

As a scoping review, the aim was to map and aggregate findings to offer and present an overview of the topic and all the material studied. Data were analyzed using descriptive statistics with Microsoft Excel, and the results were reported using narrative synthesis.

Although critical appraisal of the quality of included studies is not mandatory in scoping reviews, we decided to assess study quality so that findings from the current scoping review could inform the selection of alcohol screening tools and measures in future studies. We used the "Newcastle-Ottawa Quality Assessment Scale (NOS)" for cross-sectional studies (28-30). We slightly modified the semantics of some items to better align with this review (Supplementary File 4). The tool has three domains, each with maximum stars (points/scores): i) selection (maximum five stars/*****), ii) comparability (maximum two stars/**), and iii) outcome (maximum three stars/***) giving a total score of 10. Studies that scored 9-10 points were considered very good, those that scored 7-8

For peer review only

points were rated as good, those that scored 5-6 points were rated as satisfactory, and those that scored 4 points or less were rated as unsatisfactory (29).

RESULTS

The search yielded 12,260 articles from all databases and three additional records from Google Scholar. After deduplication, there were 9037 records, and all these articles underwent title and abstract screening. After titles/abstracts screening, 317 articles were assessed for full-text eligibility, of which 76 articles were included for data extraction. The PRISMA flow diagram summarizes this article selection process (Figure 1).

Characteristics of included studies

The publication year for included articles ranged from 1996 to 2022. Only five studies were published before 2000, 19 from 2000-2010, and 52 from 2011-2022. Of the 76 full-text articles included in this scoping review, 29 were from High-Income countries (HICs; Table 2: Supplementary File 2), and the remaining 47 studies were from low- and Middle-Income countries (LMICs; Table 3: Supplementary File 3). Of these 47 studies, 36 were from Middle-Income countries (MICs), 24 were from Upper-Middle-Income countries, 12 were from Lower-Middle-income countries, and 11 were from Low-Income countries (LICs).

Most of the studies employed a cross-sectional study design (68/76), and the rest of the studies were longitudinal/cohort designs (6/76) and mixed quantitative and qualitative designs (2/76). Almost all included studies were population or community-based surveys. For the majority of included studies (n=30, 39.5%), the study population resided in an urban location, followed by a mixed urban/rural setting (n=24, 31.6% of studies) and rural (n=9, 11.8%). Thirteen (17.1%) studies did not specify the location of the population.

Among the included studies, the total sample size ranged from 99 to 358,355 participants. Only nine studies had a sample size of less than 500 individuals. Almost 76% (n=58) of included studies had more than 1000 participants in their sample. Eight studies were conducted only among men, two only among women, and gender was not specified in four studies. There were four studies conducted among young adults (16-25 years old) and six among older people (adults ≥ 50 years old). Across studies,

For peer review only

participants ranged from 15 to 99 years old, and the mean or median age ranged from 20 to 81.

Critical appraisal of included studies

When assessing the overall methodological quality of included studies, 17 (22.4%) were rated as very good, 47 (61.8%) as good, 11 (14.5%) as satisfactory, and one (1.3%) as unsatisfactory (Supplementary File 5).

Definitions of problem drinking (PD)

Studies have delineated PD in a variety of ways, including binge (heavy episodic) drinking (BD/HED), excessive/heavy drinking (HD), or alcohol use disorder (AUD). Definitions of HD and BD/HED differed according to the recommended drinking limits of countries and how individual studies operationalized the construct. For instance, a study in Finland (31) defined HD for males as ≥ 280g of absolute ethanol or 24 drinks per week and/or a CAGE score ≥ 3 and for women as ≥ 190g of absolute ethanol or 16 drinks per week and/or a CAGE score ≥ 2. Another study in the USA (32) defined HD for males as > 14 drinks per week and > four drinks per day and for females as > seven drinks per week and > three drinks per day. This weekly drinking definition of HD is also applied in China (33). A study in France (34) defined HD as ≥ 60g ethanol per day or six glasses per day of any alcoholic drink for males and ≥ 30g per day or about three glasses per day for females. HD in two studies in the Netherlands (35, 36) and one study in Botswana (24) for women was > 14 standard glasses per week, and for men was > 21 drinks per week. Two studies in Brazil (37, 38) operationalized HD or hazardous drinking as an average of ≥ 30g per day, irrespective of gender. Studies from South Africa classified HD as > seven drinks per week (39).

HED was sometimes used interchangeably with BD. Studies in Hong Kong (40, 41) and the US (42) defined HED/BD as drinking ≥ five drinks in a row on a single occasion in the past month, irrespective of sex. Most studies described it differently for males and females. National Institute on Alcohol Abuse and Alcoholism (NIAAA) guidelines for risky drinking criteria, Substance Abuse and Mental Health Services Administration (SAMHSA) definition, or risky single occasion drinking (RSOD) criteria were mainly applied to define HED/BD (39, 43-45). In the US (46, 47), Singapore (48), Peru (43), South Africa (49), and Brazil (44, 45, 50), HED/BD was defined as ≥ five drinks per

For peer review only - http://bmjopen.bmj.com/site/about/guidelines.xhtml

occasion for men and ≥ four drinks per occasion for women, a pattern of drinking that brings blood alcohol level (BAC) to at least 0.08 g/dl and reflects ≥ 60g pure alcohol. It was also defined like this by studies conducted in India and Ireland (51, 52). In South Africa, one study (39) used a cut-off of > three drinks per occasion weekly, and another study (53) used ≥ five drinks on an average drinking day to define HED. Other studies defined BD/HED using different criteria. In Cambodia (54) and Nepal (55), this was defined as the use of ≥ six drinks in a single sitting at least monthly using NIAAA definitions, and in Ethiopia (56, 57), as an intake of ≥ six drinks in males and ≥ four drinks in females on a single occasion. The definition of BD differed in a study conducted in the United Kingdom (58), with BD defined as more than eight drinks for males and females as more than six standard drinks per session. Some studies examined RSOD, defined as ≥ six drinks per single occasion, and at-risk volume drinking, defined as ≥ 21 drinks per week, and RSOD at least monthly for men in Switzerland (59).

Hazardous/harmful alcohol use, also known as Harmful/hazardous drinking, probable alcohol use disorder (AUD), risky alcohol use, high-risk drinking, or hazardous, harmful, or dependent alcohol use, was defined as a score of ≥ eight on the Alcohol Use Disorders Identification Test (AUDIT) in most studies including studies conducted in New Zealand (60), Norway (61), Brazil (62), South Africa (50, 63), India (64-67), Kenya (68), Uganda (69), Nepal (70), Ethiopia (71-73), Malaysia (74), Thailand (75, 76), and Suriname (77). This definition is in keeping with the WHO recommended cut-offs for problem drinking on the AUDIT (17). In contrast, one study used an AUDIT score > four to define hazardous, harmful, and high-risk drinking for females in Mozambique (78).

We noted more variability in the cut-offs used across studies when using short AUDIT forms to define hazardous or harmful drinking. A cut-off score of ≥ five on AUDIT-C (a three-item version of the full AUDIT) was used in South Africa (49) and the UK (79). Risky drinking was defined as 8-12 for males and 6-12 for females on AUDIT-C in Sweden (80), while hazardous alcohol use in Ethiopia (81) was defined as a score of ≥ three on the Fast Alcohol Screening Test (FAST), a 4-item version of the AUDIT. But a different definition was applied for hazardous drinking in Russia (82), which was stated as having any of the following in the past year: having drunk surrogate alcohols (non-

For peer review only

beverage alcohols and illegally produced alcohols), having been on zapoi (several days of continuous drunkenness during which one withdraws from the society), having frequent hangovers once or more per month and having drunk spirits daily. One study in China (83) used the Michigan Alcohol Screening Test (MAST) to define cases of alcohol dependence, and it was classified using a MAST score of ≥ five with 1-4 (low), 5-6 (light), and 40-53 (severe).

Measures of problem drinking (PD)

The included studies used a mix of measurement methods to assess PD, grouped into self-report quantity/frequency (QF) questionnaires- including RSOD criteria, screening tools, or structured clinically administered (gold-standard) instruments.

Quantity/frequency questionnaires and risky single occasion drinking criteria

Of 76 studies included, 19/29 in the HICs (Table 2: Supplementary File 2) and 19/47 in the LMICs (Table 3: Supplementary File 3) used QF questionnaires. The time interval in which the pattern of alcohol consumption (frequency and quantity) was defined and reported was expressed in days, weeks, months, past 12 months (current use), and ever (lifetime) use. Some studies used country-specific guidelines of recommended limits, which are part of the QF questionnaires like French alcohol consumption habits (34), Australian National Health and MRC 2009 guidelines for mean daily alcohol intake (84), Health Council of Netherlands recommended limit for alcohol (35), and UK National Statistics definition for BD/HD (58). Nine studies from HICs and four studies from LMICs applied RSOD criteria. Among HICs, a survey in the US used NIAAA guidelines, SAMHSA definitions for BD (32, 85), and RSOD criteria were also applied in Ireland (52) and Switzerland (59).

Screening and diagnostic interviews for problem drinking

Studies used a variety of screening tools to assess PD. The most commonly used tools included the CAGE questionnaire (86-88), the AUDIT (17), the MAST (89, 90), and the Alcohol, Smoking, and Substance Involvement Screening Test (ASSIST) (91).

Specifically, three studies from HICs (31, 35, 92) and four from LMICs (38, 93-95) used the CAGE. Five studies from HICs, including New Zealand (60), the Netherlands (35), the UK (79), Norway (61), and Sweden (80), used either the full or abbreviated versions of AUDIT. Similarly, 22 studies from LMICs used AUDIT. The three-item AUDIT-C was

For peer review only - http://bmjopen.bmj.com/site/about/guidelines.xhtml

used in South Africa, Cambodia, the UK, and Sweden (49, 54, 79, 80), and a four-item version of the AUDIT- the Fast Alcohol Screening Test (FAST) was used in Ethiopia (81). Only two studies in LMICs, Suriname (77) and South Africa (63), applied ASSIST.

The included studies in the review used five different AUD diagnostic interviews. First, several studies used the Composite International Diagnostic Interview (CIDI) (96-100). Country-specific versions of CIDI-structured diagnostic tools based on DSM-III, DSM-III-R, DSM-IV, DSM-5, or ICD-10 and ICD-11 (101, 102) were administered for the detection and diagnosis of PD like AUD, AA, or AD in 11 studies from HICs including Hong Kong (41), Germany (103, 104), Israel (105), Australia (106), the Netherlands (36), Sweden (107), Ireland (52), USA (46), Finland (108), and Switzerland (59). It was also used in three studies from LMICs, including Sri Lanka (109), Ethiopia (94), and South Africa (110). Second, Alcohol Abuse and Alcoholism's Alcohol Use Disorder and Associated Disabilities Interview Schedule-DSM-IV version (AUDADIS-IV) (111) was used in one study in HICs- in the US (46). Third, the Structured Clinical Interview for DSM-IV (SCID-I) (112-114) was used. A study in Finland (108) applied SCID-I complemented by medical record data and expert interviews to detect lifetime DSM-IV substance use disorder (SUD).

Fourth, the Diagnostic and Statistical Manual of Mental Disorders- 4th and 5th editions (DSM-IV and DSM-5) (25, 26) was used. Only two HIC studies from Switzerland (59) and Sweden (107) applied DSM-IV or DSM-5 criteria to diagnose alcohol abuse, alcohol dependence, or alcohol use disorder (AUD).

Fifth, studies used the Mini International Neuropsychiatric Interview (M.I.N.I.), versions 5, 6, & 7.0.2, structured based on DSM (115-117) to detect AUD. It is a DSM-IV-based diagnostic tool for alcohol use during the past 12 months (alcohol dependence and abuse), and only one study from HICs- the USA used M.I.N.I. (118). It was employed for the detection of alcohol use in three studies from LMICs, namely South Africa (110), Malaysia (74), and Thailand (75).

Prevalence of problem drinking, its pattern, and associated factors

Prevalence and patterns of problem drinking

Six HIC studies assessed HD (Table 2: Supplementary File 2). Across these studies, the reported prevalence of HD ranged from 5.0% to 39.9% for males and from < 1.0% to

For peer review only

For peer review only - http://bmjopen.bmj.com/site/about/guidelines.xhtml

12.9% for females (31, 32, 34, 84, 105). HD was reported by eight out of 47 LMIC studies comprising Brazil (37, 38, 44), South Africa (39, 119), Botswana (24), China (33), and Brazil (95) (Table 3: Supplementary File 3). The prevalence of HD in these studies ranged from 3.2% to 53% in the overall population, from 29.2% to 31% in males, and from 3.7% to 17% in females.

BD/HED was reported in nine studies conducted in HICs, including Hong Kong (41), USA (42, 46, 47, 85), UK (58), Singapore (48), Chile (120), and Ireland (52) (Table 2: Supplementary File 2). Across these studies, the prevalence of BD/HED ranged from 14.5% to 24.7% in males, 3.5% to 18% in females, and 13.7% to 86% in the overall sample. BD/HED was also reported by fourteen out of 47 studies from LMICs consisting of South Africa (39, 50, 53), India (51), Cambodia (54), Peru (43), Brazil (44, 45), Nigeria (121), Burkina Faso (122), Nepal (55), and Ethiopia (56, 57, 71) (Table 3: Supplementary File 3). The overall prevalence of BD/HED ranged from 3.7% to 43%. BD/HED prevalence ranged from 13.7% to 48.2% in males and 2.7% to 15.0% in females.

Alcohol use disorder (AUD), including older terms such as AA and AD, was reported by 10 out of 29 HIC studies, including Hong Kong (41), Finland (108), Germany (103), Switzerland (59), Israel (105), Australia (106), UK (79), Sweden (107), Chicago, USA (118), and Ireland (52) (Table 2: Supplementary File 2). The prevalence of any lifetime or current AUD ranged from 4.3% to 36.8% in the overall population, 19.8% to 38.3% in males, and 6.3% to 20.6% in females. The prevalence of AA ranged from 4% to 4.5%, and AD ranged from 0.4% to 12.3% in the overall sample, 6.1% in males, and 6.1% in females.

Likewise, AUD comprising AA, AD, hazardous, harmful, or dependent alcohol use was reported by 29 of 47 LMIC studies, including South Africa (49, 50, 63, 110), Sri Lanka (109), Ethiopia (71-73, 81, 93, 94), China (83), Brazil (38, 62, 95), India (64-67), Kenya (68), Uganda (69), Nepal (70), Cambodia (54), Malaysia (74), Thailand (75, 76), Suriname (77), and Mozambique (78) (Table 3: Supplementary File 3). Either current or lifetime prevalence of any AUD ranged from 4.1% to 41.0% in the overall sample, from 14.5% to 47.0% in males, and from 2.0% to 12.9% in females. The prevalence of lifetime or current AA ranged from 6.2% to 9.0% in the overall sample, estimated at

For peer review only

For peer review only - http://bmjopen.bmj.com/site/about/guidelines.xhtml

19.0% in males and 6.0% in females. The prevalence of lifetime or current AD ranged from 0.8% to 26.5% in the overall population, from 1.5% to 39.0% in males, and from 0.1% to 19.1% in females.

Factors associated with problem drinking (PD)

Most studies from HICs and LMICs identified factors associated with different types of PD. These factors can be grouped into socio-demographic and socio-economic; clinical (medical problems or clinical parameters and mental disorders); substance use and risky behaviours; and psychosocial support, functioning, disability, and quality of life factors (Table 2: Supplementary File 2 & Table 3: Supplementary File 3).

Studies from both HICs and LMICs examined a range of socio-demographic factors associated with problem drinking, but the nature and direction of the relationship were inconsistent across studies. Seven out of 29 studies in HICs found that age was associated with PD. Some studies found that older age was associated with heavy drinking (35, 46), while others found that this association existed for men but not women (41). In contrast, other studies reported associations between PD and young adulthood (105, 106), with some studies noting that alcohol use declined with age (80), and age was associated with abstention among women (32) and inversely associated with heavy drinking among men (34, 84). Furthermore, eighteen out of 47 studies in LMICs found that age was associated with PD. Some studies reported that older age was associated with alcohol use and different types of PD (37, 38, 50, 51, 53, 67-70, 81, 94, 123), while others found that this association existed for younger age or early adulthood (37, 43, 54, 63, 72, 122). Several studies found associations between male sex and PD. Seven studies from HICs (35, 46, 80, 103, 105, 106, 118) found that male sex was associated with alcohol use and various types of PD. Another eighteen studies from LMICs found that male sex was associated with different forms of PD (24, 37, 39, 49, 54-56, 64, 68, 71-74, 81, 93, 94, 122, 123).

Some studies from HICs found associations between not being in a relationship and PD, including studies conducted in Australia (106), Israel (105), and China (41). Included studies from LMICs also reported associations between not being in a relationship and various types of AUD (45, 51, 53, 70, 77, 93, 110, 119). In contrast,

For peer review only - http://bmjopen.bmj.com/site/about/guidelines.xhtml

other studies found that these associations existed for being in a relationship (24, 56, 78) and age-gap relationships (24).

In terms of socio-economic and environmental indicators, only a couple of studies from HICs examined associations between PD and factors like educational attainment (34, 36, 84), employment (41), being immigrants (105), lower (32) or higher (84) income, location (34, 84), or higher neighborhood alcohol outlet density (85). Twelve included studies from LMICs found that education was associated with PD, with some studies finding that a lower educational level was associated with alcohol abuse and heavy drinking (38, 50, 51, 67, 77, 82, 94, 124). In contrast, others found that this association existed for higher educational levels (24, 43, 45, 63). Thirty studies conducted in LMICs examined associations between PD and economic factors, finding equivocal results. While several studies found associations between lower income (37, 38, 50, 51, 93, 109, 110, 123, 124) or unemployment (82) and PD, others found associations between PD and higher income (39, 49, 50, 54, 57, 62, 64, 78, 82, 123) or being employed (54, 55, 57, 64, 69-71, 77, 94, 122). Only a few studies from LMICs examined associations between factors like religious affiliation (74, 93, 124); living in urban or rural setting and location (50, 56, 57, 63, 67); ethnicity and race (37-39, 49, 50, 55, 63, 70, 93); household living circumstances (38, 53) and PD.

Three studies conducted in HICs (106) and fourteen in LMICs (37, 44, 62, 63, 69-74, 78, 81, 93, 109) found associations between mental disorders and different forms of PD. Only one HIC study found associations between medical problems like higher BMI and being non-diabetic than diabetic (32) and PD. In contrast, six studies from LMICs found associations between medical problems like chronic disease (37), high blood pressure (33, 83), obesity (39), self-reported physical comorbidities (67), and PD. Only a few studies from LMICs found associations between PD and less psychosocial support (72, 73, 81), more impaired functioning, disability, and poorer quality of life (45, 66, 70, 71).

In terms of other substance use factors, seven studies conducted in HICs (34, 35, 41, 46, 84, 106, 108), and sixteen studies from LMICs (37, 39, 49, 53, 56, 57, 62, 63, 67, 70, 72, 73, 93, 109, 122, 123) reported associations between cigarette smoking, other substance use and various types of PD.

For peer review only - http://bmjopen.bmj.com/site/about/guidelines.xhtml

DISCUSSION

In this scoping review, we identified 76 population-based studies (29 from HICs and 47 from LMICs) examining the prevalence of alcohol consumption and PD, assessment methods, and factors associated with PD. Included articles were published between 1996 and 2022, with more than tripling the number of published articles in the last decade compared to the previous decade.

Despite this growing body of studies on PD prevalence and alcohol measurement, this review highlights significant heterogeneity of study designs, measures, and outcomes that hamper the synthesis of evidence on alcohol prevalence and associated harms across studies.

Such a synthesis of the evidence on alcohol prevalence and alcohol-related harms is needed to convince policymakers to take action to reduce population-level alcohol use. More specifically, this review identified significant heterogeneity and inconsistency in how various forms of PD were defined and measured (24, 31-59). For example, this review found substantial variations in how PD was conceptualised, ranging from heavy drinking (HD), heavy episodic/binge drinking (HED/BD), alcohol abuse (AA), alcohol dependence (AD), and alcohol use disorder (AUD) and measured with diverse measurement tools like quantity/frequency questions, risky single occasion drinking (RSOD) criteria, screening tools, or structured diagnostic interviews (32, 34-36, 38, 39, 41, 43-46, 49, 50, 52, 54, 58-85, 91, 93-95, 103-110, 118). These tools also were variable in the timeframe used to assess PD, with the assessment period ranging from days, weeks, months, or years among the studies included in this review (32, 34, 35, 39, 43-45, 52, 58, 59, 84, 85).

This variability in how alcohol use and various forms of PD are defined and measured is a significant weakness in the literature, with previous studies noting a lack of attention to the validity of alcohol screening tools and questionnaires (125). It is crucial to have a uniform and precise definition of problem drinking that can be applied across studies. This approach will allow for a more accurate estimation of prevalence and more effective identification of people with problem drinking, and it will enhance the robustness of the evidence base on which to advocate for alcohol harm reduction.

For peer review only - http://bmjopen.bmj.com/site/about/guidelines.xhtml

Many challenges in understanding the true prevalence of problem drinking arise from different definitions and inconsistent approaches to measuring it. This was evident in the current review, where we noted considerable differences in the prevalence estimates for PD, partly due to variability in how PD was conceptualised and measured. For instance, in HIC studies, the prevalence of HD or HED/BD ranged from 5% to 39.9% in males and less than 1.0% to 18% in females. Similarly, in LMIC studies, the prevalence of HD or HED/BD ranged from 13.7% to 48.2% in males and 2.7% to 17% in females. The prevalence of AA, AD, both AA and AD combined, or AUD in the overall population ranged from 0.4% to 36.8% in HIC studies and 0.8% to 41% in LMIC studies. Further, country differences in PD, particularly HD and HED cut-offs, made comparisons across sites difficult.

Harmonized measures and consensus on the best ways of measuring alcohol use and PD would aid with comparative studies of PD prevalence. Despite the difficulties and challenges associated with building consensus on the best measures for assessing PD and various indicators of PD development, there is an increasing interest in developing agreement on this topic (126). Notably, even if consensus is reached on which measures of PD to use, these self-report measures would be subject to reporting bias, specifically under or over-reporting of alcohol consumption. These self-report measures can be supplemented with objective measures of alcohol use (alcohol biomarkers) such as Phosphatidylethanol (PEth) (127-132)

Problem drinking is affected by numerous factors at population and individual levels, and identifying these factors is important for informing the design of harm minimization interventions (133). It is important to note that this review has weaknesses concerning the examination of factors associated with problem drinking, including the use of less powerful statistical tests (nonparametric tests) or no use of statistical tests (31, 47, 52, 58, 60, 65, 69, 92, 93, 118, 121, 134), only a few variables were modeled to control confounding (43, 66, 67, 75, 104, 108, 120, 122), use of non-validated tools that could result in measurement errors (34, 35, 38, 40, 55, 58, 73, 110), sampling only (predominantly) males or females that could cause selection bias (61, 67, 78, 107), high attrition rate from the study (85, 107, 124), and small sample size (54, 64, 74). Prospective cohort studies that address these methodological limitations and examine

For peer review only

the correlates and consequences of PD are needed to guide the design of alcohol harm minimization interventions.

Strengths and limitations

Our scoping review has several strengths. The review protocol was registered at Open Science Framework (OSF), and we followed PRISMA-ScR guidelines in our scoping review. A comprehensive search strategy was employed to locate global studies. We decided and critically appraised the quality of included studies, though it is not mandatory in scoping reviews. This scoping review has several limitations. First, to make our review more feasible, we included only community-based studies and studies conducted at institutions like hospitals, primary health care services, addiction centers, and colleges/universities were not included, so comparison of findings across these populations was difficult. Second, the reports of this review may be limited to the inclusion criteria employed in which only published articles written in English were included. Accordingly, publication bias is possible as unpublished reports might have been missed on alcohol use and related conditions. Third, a meta-analysis was not performed due to the heterogeneity of included studies; as such, the pooled prevalence of problem drinking couldn't be estimated.

CONCLUSIONS

This review highlights the heterogeneity of conceptualization, measurement, and reporting of PD and methodological weaknesses across included studies, which limits our confidence in the accuracy of prevalence estimates for PD, our ability to compare findings across studies, and pool data for pooled prevalence estimates. Future alcohol use-related research could improve the quality and reliability of findings by strictly following a priori proposed methods and protocols like using valid alcohol use measures, applying appropriate statistical tests, controlling possible confounders, minimizing selection bias, and using a sufficiently large and justifiable sample size.

Abbreviations

AA: Alcohol abuse; AD: Alcohol dependence; ASSIST: Alcohol, Smoking, and Substance Involvement Screening Test; AUD: Alcohol use disorder; AUDIT: Alcohol Use Disorder Identification Test; BD: Binge drinking; CIDI: Composite International Diagnostic Interview; DSM-III/DSM-III-R: The Diagnostic and Statistical Manual of

For peer review only

For peer review only - http://bmjopen.bmj.com/site/about/guidelines.xhtml

Mental Disorders, Third Edition; DSM-IV/DSM-5: Diagnostic and Statistical Manual of Mental Disorders 4th and 5th edition; HD: Heavyy drinking; HED: Heavy episodic drinking; HICs: High-Income countries; ICD: The International Classification of Diseases; LMICs: Low-and Middle-Income countries; MINI: Mini International Neuropsychiatric Interview; MRC: Medical Research Council; PD: Problem drinking; PRISMA-ScR: Preferred Reporting Items for Systematic reviews and Meta-Analyses extension for Scoping Reviews; QF: quantity/frequency questionnaires; RSOD: risky single occasion drinking criteria; SCID: Structured Clinical Interview for DSM Disorders; WHO: World Health Organization

Acknowledgments

Our appreciation is dedicated to AMARI (African Mental heAlth Research Initiative) and Addis Ababa University (AAU) for providing training to Kefyalew Dagne in "Systematic Review & Meta-Analysis." The authors would like to acknowledge the Ethiopian Public Health Association (EPHA) Annual Scientific Conference for providing the opportunity to present this research at their 34th (2023) conference.

Author Contributions

KD was involved in the project's conceptualization, writing the protocol, developing a search strategy, searching, screening, and extracting included articles, synthesizing the results, writing the discussion section of the manuscript, and harmonizing the entire document. ST approved the conceptualized research project, the protocol, and the draft manuscript. BM reviewed the search strategy and provided in-depth reviews of the manuscript. AM was involved in screening and extracting included articles. All authors involved read and approved the final manuscript.

Funding

Kefylew Dagne was supported through AMARI, funded through the DELTAS Africa Initiative (DEL-15-01). The DELTAS Africa Initiative is an independent funding scheme of the African Academy of Sciences (AAS)'s Alliance for Accelerating Excellence in Science in Africa (AESA) and supported by the New Partnership for Africa's Development Planning and Coordinating Agency (NEPAD Agency) with funding from the Wellcome Trust (DEL-15-01) and the UK government. The views expressed in this

For peer review only

publication are those of the author (s) and not necessarily those of AAS, NEPAD Agency, Wellcome Trust, or the UK government.

Availability of data and materials

All relevant materials and data supporting the results of this study are contained within the manuscript, and relevant documents will be available upon request.

Ethics approval and consent to participate

Not applicable.

Consent for publication

Not applicable.

Competing interests

The authors declare that they have no competing interests.

Author Details

1 Department of Psychiatry, School of Medicine, College of Health Sciences, Addis Ababa University, Addis Ababa, Ethiopia

2 Department of Psychiatry, College of Health Sciences and Medicine, Debre Berhan University, Debre Berhan, Ethiopia

3 Curtin enAble Institute, Faculty of Health Sciences, Curtin University, Kent Street, Bentley, WA 6102, Australia

4 Alcohol, Tobacco and Other Drug Research Unit, South African Medical Research Council, Cape Town, South Africa

5 Division of Addiction Psychiatry, Department of Psychiatry and Mental Health, University of Cape Town, Cape Town, South Africa

Figure Legends

Figure 1: PRISMA flow diagram of included studies in the scoping review, 2023.

For peer review only

References

1. Toner P, Böhnke JR, Andersen P, McCambridge J. Alcohol screening and assessment measures for young people: A systematic review and meta-analysis of validation studies. Drug and alcohol dependence. 2019;202:39-49.
2. Griswold MG, Fullman N, Hawley C, Arian N, Zimsen SR, Tymeson HD, et al. Alcohol use and burden for 195 countries and territories, 1990–2016: a systematic analysis for the Global Burden of Disease Study 2016. The Lancet. 2018;392(10152):1015-35.
3. Organization WH. Global status report on alcohol and health 2018: World Health Organization; 2019.
4. World Health Organization. Management of Substance Abuse Unit. Global status report on alcohol and health, 2014: World Health Organization; 2014.
5. Corrao G, Bagnardi V, Zambon A, La Vecchia C. A meta-analysis of alcohol consumption and the risk of 15 diseases. Preventive medicine. 2004;38(5):613-9.
6. Low WY, Lee YK, Samy AL. Non-communicable diseases in the Asia-Pacific region: Prevalence, risk factors and community-based prevention. Int J Occup Med Environ Health. 2015;28(1):20-6.
7. HELZER JE, PRZYBECK TR. The co–occurrence of alcoholism with other psychiatric disorders in the general population and its impact on treatment Journal of Studies on Alcohol 1988;49:219–24.
8. Fone D, Dunstan F, White J, Webster C, Rodgers S, Lee S, et al. Change in alcohol outlet density and alcohol-related harm to population health (CHALICE). BMC public health. 2012;12:428.
9. Lester L, Baker R, Coupland C, Orton E. Alcohol misuse and injury outcomes in young people aged 10–24. Journal of Adolescent Health. 2018;62(4):450-6.
10. Newbury-Birch D, Ferguson J, Connor N, Divers A, Waller G. A rapid systematic review of worldwide alcohol use disorders and brief alcohol interventions in the criminal justice system. Frontiers in psychiatry. 2022;13.
11. Hagman BT, Falk D, Litten R, Koob GF. Defining recovery from alcohol use disorder: development of an NIAAA research definition. American Journal of Psychiatry. 2022;179(11):807-13.
12. Enoch M-A, Goldman D. Problem drinking and alcoholism: diagnosis and treatment. American family physician. 2002;65(3):441-9.
13. Van Oers J, Bongers I, Van de Goor L, Garretsen H. Alcohol consumption, alcohol-related problems, problem drinking, and socioeconomic status. Alcohol and alcoholism (Oxford, Oxfordshire). 1999;34(1):78-88.
14. Mosel S. Problem Drinking vs. Alcoholism [updated 25 October 2022. Available from: https://alcohol.org/alcoholism/or-is-it-just-a-problem/.
15. American Addiction Centers. Problem Drinker Defined [updated October 9, 2020. Available from: https://alcoholrehab.com/alcoholism/signs-of-alcoholism/problem-drinker-defined/.
16. HRB National Drugs Library. Research Glossary [Available from: https://www.drugsandalcohol.ie/glossary/ Accessed 6 July 2023.
17. Saunders JB, Aasland OG, Babor TF, De la Fuente JR, Grant M. Development of the alcohol use disorders identification test (AUDIT): WHO collaborative project on early detection of persons with harmful alcohol consumption-II. Addiction (Abingdon, England). 1993;88(6):791-804.
18. Edwards G, Arif A, Hadgson R. Nomenclature and classification of drug-and alcohol-related problems: a WHO Memorandum. Bulletin of the World Health Organization. 1981;59(2):225-42.
19. Organization WH. The ICD-10 classification of mental and behavioural disorders: clinical descriptions and diagnostic guidelines: World Health Organization; 1992.
20. Babor TF, de la Fuente JR, Saunders J, Grant M. The Alcohol Use Disorders Identification Test: Guidelines for use in. Primary care. 2001.

For peer review only

21. Fairbairn N, Wood E, Dobrer S, Dong H, Kerr T, Debeck K. The relationship between hazardous alcohol use and violence among street-involved youth. The American journal on addictions. 2017;26(8):852-8.
22. Reid MC, Fiellin DA, O'Connor PG. Hazardous and harmful alcohol consumption in primary care. Archives of internal medicine. 1999;159(15):1681-9.
23. Tsai J, Ford ES, Li C, Pearson WS, Zhao G. Binge drinking and suboptimal self-rated health among adult drinkers. Alcoholism, clinical and experimental research. 2010;34(8):1465-71.
24. Weiser SD, Leiter K, Heisler M, McFarland W, Percy-de Korte F, DeMonner SM, et al. A population-based study on alcohol and high-risk sexual behaviors in Botswana. PLoS medicine. 2006;3(10):e392.
25. American Psychiatric Association. Diagnostic and statistical manual of mental disorders: DSM-IV: American psychiatric association Washington, DC; 1994.
26. American Psychiatric Association. Diagnostic and statistical manual of mental disorders: DSM-5. Fifth ed: Arlington, VA, American Psychiatric Association; 2013.
27. Tricco AC, Lillie E, Zarin W, O'Brien KK, Colquhoun H, Levac D, et al. PRISMA extension for scoping reviews (PRISMA-ScR): checklist and explanation. Annals of internal medicine. 2018;169(7):467-73.
28. Ribeiro CM, Beserra BTS, Silva NG, Lima CL, Rocha PRS, Coelho MS, et al. Exposure to endocrine-disrupting chemicals and anthropometric measures of obesity: A systematic review and meta-analysis. BMJ open. 2020;10(6):e033509.
29. Herzog R, Álvarez-Pasquin M, Díaz C, Del Barrio JL, Estrada JM, Gil Á. Are healthcare workers' intentions to vaccinate related to their knowledge, beliefs and attitudes? A systematic review. BMC public health. 2013;13(1):1-17.
30. Dubey VP, Kievišienė J, Rauckiene-Michealsson A, Norkiene S, Razbadauskas A, Agostinis-Sobrinho C. Bullying and Health Related Quality of Life among Adolescents—A Systematic Review. Children. 2022;9(6):766.
31. Aalto M, Seppa K, Kiianmaa K, Sillanaukee P. Drinking habits and prevalence of heavy drinking among primary health care outpatients and general population. Addiction (Abingdon, England). 1999;94(9):1371-9.
32. Andrews-Chavez JY, Lee CS, Houser RF, Falcon LM, Tucker KL. Factors associated with alcohol consumption patterns in a Puerto Rican urban cohort. Public health nutrition. 2015;18(3):464-73.
33. Ding L, Liang Y, Tan ECK, Hu Y, Zhang C, Liu Y, et al. Smoking, heavy drinking, physical inactivity, and obesity among middle-aged and older adults in China: cross-sectional findings from the baseline survey of CHARLS 2011-2012. BMC public health. 2020;20(1):1062.
34. Bataille V, Ruidavets JB, Arveiler D, Amouyel P, Ducimetiere P, Perret B, et al. Joint use of clinical parameters, biological markers and cage questionnaire for the identification of heavy drinkers in a large population-based sample. Alcohol and Alcoholism. 2003;38(2):121-7.
35. Geels LM, Vink JM, van Beek JH, Bartels M, Willemsen G, Boomsma DI. Increases in alcohol consumption in women and elderly groups: evidence from an epidemiological study. BMC public health. 2013;13:207.
36. Veerbeek MA, Ten Have M, van Dorsselaer SA, Oude Voshaar RC, Rhebergen D, Willemse BM. Differences in alcohol use between younger and older people: Results from a general population study. Drug and alcohol dependence. 2019;202:18-23.
37. Dias da Costa JS, Silveira MF, Gazalle FK, Oliveira SS, Hallal PC, Menezes AMB, et al. Heavy alcohol consumptions and associated factors: A population-based study. Revista de saude publica. 2004;38(2):284-91.

For peer review only

38. Moreira LB, Fuchs FD, Moraes RS, Bredemeier M, Cardozo S, Fuchs SC, et al. Alcoholic beverage consumption and associated factors in Porto Alegre, a southern Brazilian city: a population-based survey. Journal of studies on alcohol. 1996;57(3):253-9.
39. Peltzer K, Phaswana-Mafuya N. Problem drinking and associated factors in older adults in South Africa. African journal of psychiatry. 2013;16(2):104-9.
40. Janghorbani M, Ho SY, Lam TH, Janus ED. Prevalence and correlates of alcohol use: A populationbased study in Hong Kong. Addiction (Abingdon, England). 2003;98(2):215-24.
41. Kim JH, Lee S, Chow J, Lau J, Tsang A, Choi J, et al. Prevalence and the factors associated with binge drinking, alcohol abuse, and alcohol dependence: a population-based study of Chinese adults in Hong Kong. Alcohol and alcoholism (Oxford, Oxfordshire). 2008;43(3):360-70.
42. Miller JW, Gfroerer JC, Brewer RD, Naimi TS, Mokdad A, Giles WH. Prevalence of adult binge drinking: a comparison of two national surveys. American journal of preventive medicine. 2004;27(3):197-204.
43. Hernandez-Vasquez A, Chacon-Torrico H, Vargas-Fernandez R, Grendas LN, Bendezu-Quispe G. Gender Differences in the Factors Associated with Alcohol Binge Drinking: A Population-Based Analysis in a Latin American Country. International journal of environmental research and public health. 2022;19(9):4931.
44. Oancea SC, de Oliveira GD, Sukumaran P, Vogeltanz-Holm N, Nucci LB. The association between alcohol consumption and self-reported current depression among adults residing in Brazil. Journal of public health (Oxford, England). 2021;43(2):e204-e12.
45. Prais HAC, De Loyola Filho AI, Firmo JOA, Lima-Costa MF, Uchoa E. A population-based study on binge drinking among elderly Brazilian men: Evidence from the Belo Horizonte and Bambui health surveys. Revista Brasileira de Psiquiatria. 2008;30(2):118-23.
46. Chou KL, Liang K, Mackenzie CS. Binge drinking and axis I psychiatric disorders in community-dwelling middle-aged and older adults: Results from the National Epidemiologic Survey on Alcohol and Related Conditions (NESARC). Journal of Clinical Psychiatry. 2011;72(5):640-7.
47. Shockey TM, Esser MB. Binge Drinking by Occupation Groups among Currently Employed U.S. Adults in 32 States, 2013-2016. Substance use & misuse. 2020;55(12):1968-79.
48. Lee YY, Wang P, Abdin E, Chang S, Shafie S, Sambasivam R, et al. Prevalence of binge drinking and its association with mental health conditions and quality of life in Singapore. Addictive behaviors. 2020;100:106114.
49. Peltzer K, Ramlagan S, Satekge M. Alcohol use, problem drinking and health risk factors among South African youths. Journal of Psychology in Africa. 2012;22(4):671-6.
50. Peltzer K, Davids A, Njuho P. Alcohol use and problem drinking in South Africa: findings from a national population-based survey. African journal of psychiatry. 2011;14(1):30-7.
51. Pillai A, Nayak MB, Greenfield TK, Bond JC, Nadkarni A, Patel V. Patterns of alcohol use, their correlates, and impact in male drinkers: a population-based survey from Goa, India. Social psychiatry and psychiatric epidemiology. 2013;48(2):275-82.
52. O'Dwyer C, Mongan D, Millar SR, Rackard M, Galvin B, Long J, et al. Drinking patterns and the distribution of alcohol-related harms in Ireland: evidence for the prevention paradox. BMC public health. 2019;19(1):1323.
53. Vellios N, Van Walbeek C. Self-reported alcohol use and binge drinking in South Africa: Evidence from the National Income Dynamics Study, 2014-2015. South African Medical Journal. 2018;108(1):33-9.
54. Yeung W, Leong W-Y, Khoun K, Ong W, Sambi S, Lim S-M, et al. Alcohol use disorder and heavy episodic drinking in rural communities in cambodia: Risk factors and community-perceived strategies. Asia-Pacific Journal of Public Health. 2015;27(8):835-47.

For peer review only

55. Dahal S, Sah RB, Niraula SR, Karkee R, Chakravartty A. Prevalence and determinants of noncommunicable disease risk factors among adult population of Kathmandu. PloS one. 2021;16(9 September):e0257037.
56. Getachew T, Defar A, Teklie H, Gonfa G, Bekele A, Bekele A, et al. Magnitude and predictors of excessive alcohol use in Ethiopia: findings from the 2015 national non-communicable diseases STEPS survey. Ethiopian Journal of Health Development. 2017;31(1):312-9.
57. Gutema BT, Chuka A, Ayele G, Tariku EZ, Aschalew Z, Baharu A, et al. Prevalence of heavy episodic drinking and associated factors among adults residing in Arba Minch health and demographic surveillance site: a cross sectional study. BMC public health. 2020;20(1):1895.
58. Williamson RJ, Sham P, Ball D. Binge drinking trends in a UK community-based sample. Journal of Substance Use. 2003;8(4):234-7.
59. Mohler-Kuo M, Foster S, Gmel G, Dey M, Dermota P. DSM-IV and DSM-5 alcohol use disorder among young Swiss men. Addiction (Abingdon, England). 2015;110(3):429-40.
60. Foulds J, Wells JE, Lacey C, Adamson S, Mulder R. Harmful drinking and talking about alcohol in primary care: New Zealand population survey findings. Acta psychiatrica Scandinavica. 2012;126(6):434-9.
61. Husberg VH, Hopstock LA, Friborg O, Rosenvinge JH, Bergvik S, Rognmo K. Epidemiology of comorbid hazardous alcohol use and insomnia in 19 185 women and men attending the population-based Tromso Study 2015-2016. BMC public health. 2022;22(1):844.
62. Mendoza-Sassi RA, Beria JU. Prevalence of alcohol use disorders and associated factors: a population-based study using AUDIT in southern Brazil. Addiction (Abingdon, England). 2003;98(6):799-804.
63. Pengpid S, Peltzer K, Ramlagan S. Prevalence and correlates of hazardous, harmful or dependent alcohol use and drug use amongst persons 15 years and older in South Africa: Results of a national survey in 2017. African journal of primary health care & family medicine. 2021;13(1):e1-e8.
64. Sau A. AUDIT (Alcohol use disorders identification test) to estimate the pattern and correlates of alcohol consumption among the adult population of west bengal, india: A community based cross sectional study. Journal of Clinical and Diagnostic Research. 2017;11(4):LC01-LC4.
65. Jonas JB, Nangia V, Rietschel M, Paul T, Behere P, Panda-Jonas S. Prevalence of depression, suicidal ideation, alcohol intake and nicotine consumption in rural central India. The Central India Eye and Medical Study. PloS one. 2014;9(11).
66. Olickal JJ, Saya GK, Selvaraj R, Chinnakali P. Association of alcohol use with quality of life (QoL): A community based study from Puducherry, India. Clinical Epidemiology and Global Health. 2021;10:100697.
67. Olickal JJ, Selvaraj R, Saya GK, Chinnakali P. Alcohol use among adult men and its impact on personal and family life: findings of a community-based cross-sectional survey and focus groups discussions from a low alcohol taxed region, Puducherry, India. Family practice. 2022;39(2):316-22.
68. Takahashi R, Wilunda C, Magutah K, Mwaura-Tenambergen W, Wilunda B, Perngparn U. Correlates of alcohol consumption in rural western Kenya: A cross-sectional study. BMC psychiatry. 2017;17(1):175.
69. Nalwadda O, Rathod SD, Nakku J, Lund C, Prince M, Kigozi F. Alcohol use in a rural district in Uganda: findings from community-based and facility-based cross-sectional studies. International journal of mental health systems. 2018;12:12.
70. Rathod SD, Luitel NP, Jordans MJD. Prevalence and correlates of alcohol use in a central Nepal district: secondary analysis of a population-based cross-sectional study. Global mental health (Cambridge, England). 2018;5:e37.

For peer review only

For peer review only - http://bmjopen.bmj.com/site/about/guidelines.xhtml

71. Zewdu S, Hanlon C, Fekadu A, Medhin G, Teferra S. Treatment gap, help-seeking, stigma and magnitude of alcohol use disorder in rural Ethiopia. Substance abuse treatment, prevention, and policy. 2019;14(1):4.
72. Endashaw Hareru H, Kaso AW, Debela BG, Abebe L, Sisay WTD, Kassa Abebe R, et al. Alcohol use disorder and its associated factors among residents in Southern Ethiopia during the era of COVID-19. SAGE open medicine. 2022;10:20503121221105031.
73. Legas G, Asnakew S, Belete A, Beyene GM, Wubet GM, Bayih WA, et al. Magnitude and correlates of alcohol use disorder in south Gondar zone, northwest Ethiopia: A community based cross-sectional study. PloS one. 2021;16(9):e0257804.
74. Abd Rashid RB, Mohd Daud MNB, Guad RM, Gan SH, Wan Husin W, Giloi N, et al. Prevalence and risk factors associated with alcohol consumption among indigenous people in Sabah Borneo Island. The Australian journal of rural health. 2021;29(3):464-72.
75. Assanangkornchai S, Nontarak J, Aekplakorn W, Chariyalertsak S, Kessomboon P, Taneepanichskul S. Socio-economic inequalities in the association between alcohol use disorder and depressive disorder among Thai adults: a population-based study. BMC psychiatry. 2020;20(1):553.
76. Jirapramukpitak T, Prince M, Harpham T. Rural-urban migration, illicit drug use and hazardous/harmful drinking in the young Thai population. Addiction (Abingdon, England). 2008;103(1):91-100.
77. Jadnanansing R, Blankers M, Dwarkasing R, Etwaroo K, Lumsden V, Dekker J, et al. Prevalence of substance use disorders in an urban and a rural area in Suriname. Trop Med Health. 2021;49(1):12.
78. Wainberg M, Oquendo MA, Peratikos MB, Gonzalez-Calvo L, Pinsky I, Duarte CS, et al. Hazardous alcohol use among female heads-of-household in rural Mozambique. Alcohol (Fayetteville, NY). 2018;73:37-44.
79. Britton A, Fat LN, Neligan A. The association between alcohol consumption and sleep disorders among older people in the general population. Scientific reports. 2020;10(1):5275.
80. Lindstrom J, Hellstrom C, Simonsson B, Molarius A. Alcohol consumption and self-rated health among older people: population-based study in Sweden. Journal of public health (Oxford, England). 2020;42(4):756-65.
81. Teferra S, Medhin G, Selamu M, Bhana A, Hanlon C, Fekadu A. Hazardous alcohol use and associated factors in a rural Ethiopian district: a cross-sectional community survey. BMC public health. 2016;16:218.
82. Tomkins S, Saburova L, Kiryanov N, Andreev E, McKee M, Shkolnikov V, et al. Prevalence and socio-economic distribution of hazardous patterns of alcohol drinking: study of alcohol consumption in men aged 25-54 years in Izhevsk, Russia. Addiction (Abingdon, England). 2007;102(4):544-53.
83. Ji A, Lou P, Dong Z, Xu C, Zhang P, Chang G, et al. The prevalence of alcohol dependence and its association with hypertension: a population-based cross-sectional study4 in Xuzhou city, China. BMC public health. 2018;18(1):364.
84. Coulson CE, Williams LJ, Henry MJ, Berk M, Lubman DI, Brennan SL, et al. Patterns of alcohol use and associated physical and lifestyle characteristics according to new Australian guidelines. The Australian and New Zealand journal of psychiatry. 2010;44(10):946-51.
85. Auchincloss AH, Niamatullah S, Adams M, Melly SJ, Li J, Lazo M. Alcohol outlets and alcohol consumption in changing environments: prevalence and changes over time. Substance abuse treatment, prevention, and policy. 2022;17(1):7.
86. Ewing J, Rose B. Identifying the Hidden Alcoholic [w:] Paper read at the 29th International Congress on Alcoholism and Drug Dependence. Australia Butterworth, Sydney. 1970.
87. Ewing JA. Detecting alcoholism: the CAGE questionnaire. Jama. 1984;252(14):1905-7.
88. Mayfield D, McLeod G, Hall P. The CAGE questionnaire: validation of a new alcoholism screening instrument. American journal of psychiatry. 1974;131(10):1121-3.

For peer review only - http://bmjopen.bmj.com/site/about/guidelines.xhtml

89. Selzer ML. The Michigan Alcoholism Screening Test: The quest for a new diagnostic instrument. American journal of psychiatry. 1971;127(12):1653-8.
90. Pokorny AD, Miller BA, Kaplan HB. The brief MAST: A shortened version of the Michigan Alcoholism Screening Test. American journal of psychiatry. 1972;129(3):342-5.
91. Group WAW. The alcohol, smoking and substance involvement screening test (ASSIST): development, reliability and feasibility. Addiction (Abingdon, England). 2002;97(9):1183-94.
92. Aira M, Hartikainen S, Sulkava R. Community prevalence of alcohol use and concomitant use of medication - A source of possible risk in the elderly aged 75 and older? International journal of geriatric psychiatry. 2005;20(7):680-5.
93. Alem A, Kebede D, Kullgren G. The epidemiology of problem drinking in Butajira, Ethiopia. Acta psychiatrica Scandinavica Supplementum. 1999;397:77-83.
94. Kebede D, Alem A. The epidemiology of alcohol dependence and problem drinking in Addis Ababa, Ethiopia. Acta psychiatrica Scandinavica Supplementum. 1999;397:30-4.
95. Moreira LB, Fuchs FD, Moraes RS, Bredemeier M, Duncan BB. Alcohol intake and blood pressure: The importance of time elapsed since last drink. Journal of hypertension. 1998;16(2):175-80.
96. Organization WH, Organization WH. CIDI-interview (version 1.0),(b) CIDI-user manual,(c) CIDI-training manual,(d) CIDI-computer programs. Geneva: World Health Organization.
97. Robins L, Wittchen H, Wing J, Sartorius N, Pull C, Towle L, et al. The composite international diagnostic interview (CIDI) reliability and applicability in different countries. Psychiatry: A world perspective. 1990;1:118-24.
98. Robins LN, Wing J, Wittchen HU, Helzer JE, Babor TF, Burke J, et al. The Composite International Diagnostic Interview: an epidemiologic instrument suitable for use in conjunction with different diagnostic systems and in different cultures. Archives of general psychiatry. 1988;45(12):1069-77.
99. Wittchen H-U, Robins LN, Cottler LB, Sartorius N, Burke JD, Regier D. Cross-cultural feasibility, reliability and sources of variance of the Composite International Diagnostic Interview (CIDI). The British Journal of Psychiatry. 1991;159(5):645-53.
100. Kessler RC, Üstün TB. The world mental health (WMH) survey initiative version of the world health organization (WHO) composite international diagnostic interview (CIDI). International journal of methods in psychiatric research. 2004;13(2):93-121.
101. WHO. The ICD-10 classification of mental and behavioural disorders: clinical descriptions and diagnostic guidelines: World Health Organization; 1992.
102. Reed GM, First MB, Kogan CS, Hyman SE, Gureje O, Gaebel W, et al. Innovations and changes in the ICD-11 classification of mental, behavioural and neurodevelopmental disorders. World psychiatry. 2019;18(1):3-19.
103. Meyer C, Rumpf HJ, Hapke U, Dilling H, John U. Prevalence of alcohol consumption, abuse and dependence in a country with high per capita consumption: findings from the German TACOS study. Transitions in Alcohol Consumption and Smoking. Social psychiatry and psychiatric epidemiology. 2000;35(12):539-47.
104. Bott K, Meyer C, Rumpf H-J, Hapke U, John U. Psychiatric Disorders among At-Risk Consumers of Alcohol in the General Population. Journal of studies on alcohol. 2005;66(2):246-53.
105. Neumark YD, Lopez-Quintero C, Grinshpoon A, Levinson D. Alcohol drinking patterns and prevalence of alcohol-abuse and dependence in the Israel National Health Survey. The Israel journal of psychiatry and related sciences. 2007;44(2):126-35.
106. Proodfoot H, Teeson M. Who seeks treatment for alcohol dependence? Findings form the Australian National Survey of Mental Health and Wellbeing. Social psychiatry and psychiatric epidemiology. 2002;37(10):451-6.

For peer review only

107. Lundin A, Waern M, Love J, Lovestad S, Hensing G, Danielsson AK. Towards ICD-11 for alcohol dependence: Diagnostic agreement with ICD-10, DSM-5, DSM-IV, DSM-III-R and DSM-III diagnoses in a Swedish general population of women. Drug and alcohol dependence. 2021;227:108925.
108. Latvala A, Tuulio-Henriksson A, Perala J, Saarni SI, Aalto-Setala T, Aro H, et al. Prevalence and correlates of alcohol and other substance use disorders in young adulthood: A population-based study. BMC psychiatry. 2009;9:73.
109. Zavos HM, Siribaddana S, Ball HA, Lynskey MT, Sumathipala A, Rijsdijk FV, et al. The prevalence and correlates of alcohol use and alcohol use disorders: a population based study in Colombo, Sri Lanka. BMC psychiatry. 2015;15:158.
110. Andersson LMC, Twum-Antwi A, Staland-Nyman C, van Rooyen D. Prevalence and socioeconomic characteristics of alcohol disorders among men and women in the Eastern Cape Province, South Africa. Health & social care in the community. 2018;26(1):e143-e53.
111. Grant BF, Dawson DA, Hasin DS. The alcohol use disorder and associated disabilities interview schedule-DSM-IV version. Bethesda, MD: National Institute on Alcohol Abuse and Alcoholism. 2001.
112. Robins LN, Helzer JE, Croughan J, Ratcliff KS. National Institute of Mental Health diagnostic interview schedule: Its history, characteristics, and validity. Archives of general psychiatry. 1981;38(4):381-9.
113. Spitzer RL, Williams JB, Gibbon M, First MB. The structured clinical interview for DSM-III-R (SCID): I: history, rationale, and description. Archives of general psychiatry. 1992;49(8):624-9.
114. First MB, Spitzer RL, Gibbon M, Williams JB. Computer-assisted SCID-Clinician version. 1997.
115. Sheehan D, Janavs J, Baker R, Harnett-Sheehan K, Knapp E, Sheehan M. Mini international neuropsychiatric interview. Tampa: University of South Florida. 1994.
116. Sheehan D, Lecrubier Y, Sheehan KH, Janavs J, Weiller E, Keskiner A, et al. The validity of the Mini International Neuropsychiatric Interview (MINI) according to the SCID-P and its reliability. European psychiatry. 1997;12(5):232-41.
117. Sheehan DV, Lecrubier Y, Sheehan KH, Amorim P, Janavs J, Weiller E, et al. The Mini-International Neuropsychiatric Interview (MINI): the development and validation of a structured diagnostic psychiatric interview for DSM-IV and ICD-10. Journal of clinical psychiatry. 1998;59(20):22-33.
118. Mondi CF, Giovanelli A, Ou SR, Reynolds AJ. Psychiatric and substance use disorders in a predominately low-income, black sample in early midlife. Journal of psychiatric research. 2022;148:332-9.
119. Trangenstein PJ, Morojele NK, Lombard C, Jernigan DH, Parry CD. Heavy drinking and contextual risk factors among adults in South Africa: findings from the International Alcohol Control study. Substance abuse treatment, prevention, and policy. 2018;13(1):43.
120. Mason-Jones AJ, Cabieses B. Alcohol, binge drinking and associated mental health problems in young urban Chileans. PloS one. 2015;10(4).
121. Aremu T, Anibijuwon IB, John-Akinola YO, Oluwasanu M, Oladepo O. Prevalence and Factors Associated With Alcohol Use in Selected Urban Communities in Ibadan, Nigeria. International quarterly of community health education. 2021:272684x211006515.
122. Bonnechère B, Samadoulougou S, Cisse K, Tassembedo S, Kouanda S, Kirakoya-Samadoulougou F. Alcohol consumption and associated risk factors in Burkina Faso: results of a population-based cross-sectional survey. BMJ open. 2022;12(2):e058005.
123. Lo TQ, Oeltmann JE, Odhiambo FO, Beynon C, Pevzner E, Cain KP, et al. Alcohol use, drunkenness and tobacco smoking in rural western Kenya. Tropical medicine & international health : TM & IH. 2013;18(4):506-15.
124. Burazeri G, Kark JD. Prevalence and determinants of binge drinking in middle age in a transitional post-communist country: a population-based study in Tirana, Albania. Alcohol and alcoholism (Oxford, Oxfordshire). 2010;45(2):180-7.

For peer review only - http://bmjopen.bmj.com/site/about/guidelines.xhtml

125. Midanik L. The validity of self-reported alcohol consumption and alcohol problems: a literature review. British journal of addiction. 1982;77(4):357-82.
126. Hussong AM, Gottfredson NC, Bauer DJ, Curran PJ, Haroon M, Chandler R, et al. Approaches for creating comparable measures of alcohol use symptoms: Harmonization with eight studies of criminal justice populations. Drug and alcohol dependence. 2019;194:59-68.
127. Litten RZ, Bradley AM, Moss HB. Alcohol biomarkers in applied settings: recent advances and future research opportunities. Alcoholism: Clinical and Experimental Research. 2010;34(6):955-67.
128. Nanau RM, Neuman MG. Biomolecules and biomarkers used in diagnosis of alcohol drinking and in monitoring therapeutic interventions. Biomolecules. 2015;5(3):1339-85.
129. Elrasheed A, Al Ghaferi H, Ali AY. Assessment of alcohol exposure: testing for ethylglucuronide (Etg), ethylsulfate (Ets). Int J Emerg Ment Health. 2017;19.
130. Viel G, Boscolo-Berto R, Cecchetto G, Fais P, Nalesso A, Ferrara SD. Phosphatidylethanol in blood as a marker of chronic alcohol use: a systematic review and meta-analysis. International journal of molecular sciences. 2012;13(11):14788-812.
131. Aradottir S, Asanovska G, Gjerss S, Hansson P, Alling C. Phosphatidylethanol (PEth) concentrations in blood are correlated to reported alcohol intake in alcohol-dependent patients. Alcohol and alcoholism. 2006;41(4):431-7.
132. Kechagias S, Dernroth DN, Blomgren A, Hansson T, Isaksson A, Walther L, et al. Phosphatidylethanol compared with other blood tests as a biomarker of moderate alcohol consumption in healthy volunteers: a prospective randomized study. Alcohol and Alcoholism. 2015;50(4):399-406.
133. Park SH, Kim DJ. Global and regional impacts of alcohol use on public health: Emphasis on alcohol policies. Clinical and molecular hepatology. 2020;26(4):652.
134. Sarma PS, Sadanandan R, Thulaseedharan JV, Soman B, Srinivasan K, Varma RP, et al. Prevalence of risk factors of non-communicable diseases in Kerala, India: results of a cross-sectional study. BMJ open. 2019;9(11):e027880.

For peer review only

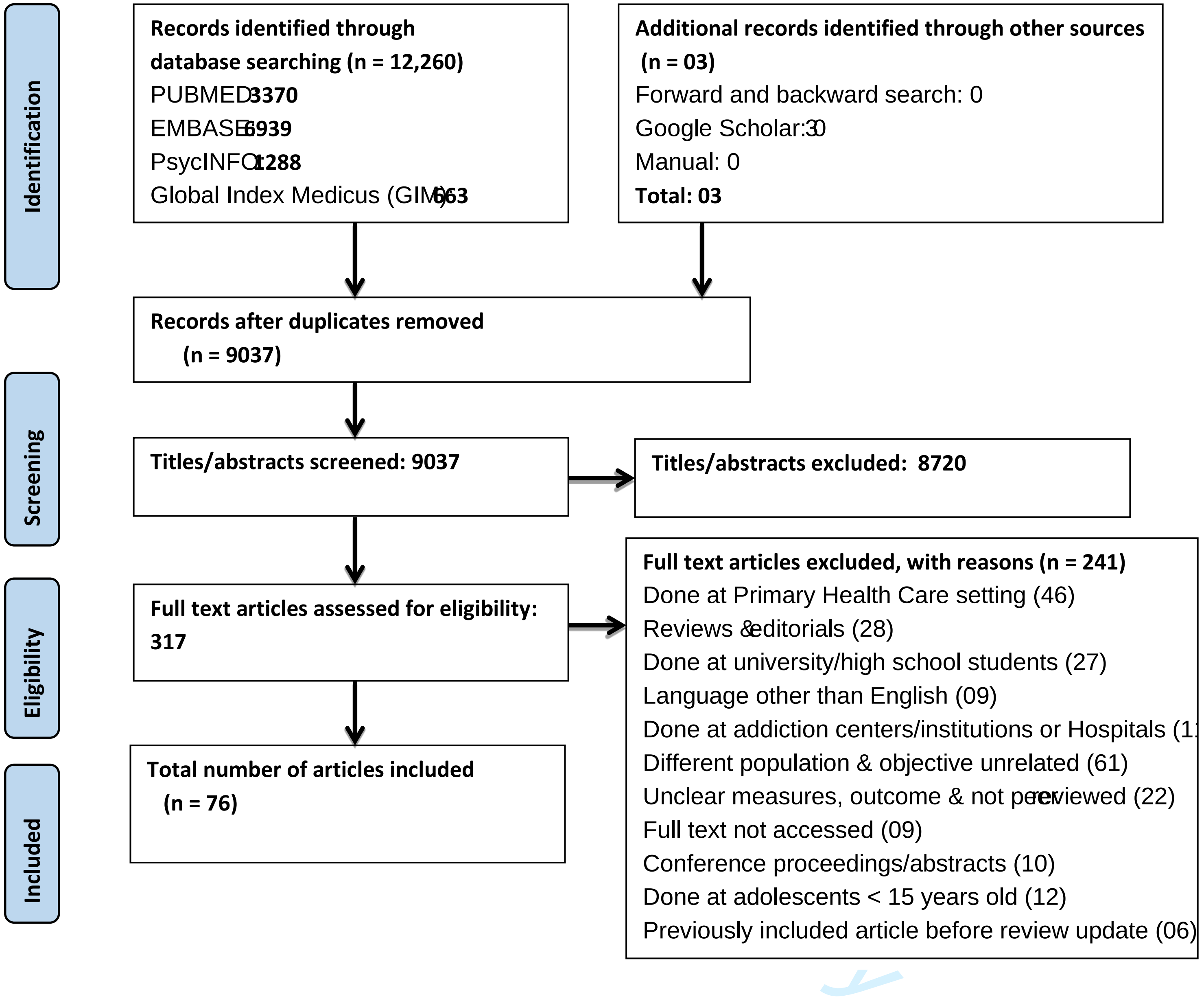

Figure 1: PRISMA flow diagram of included studies in the scoping review, 2023.

Supplementary File 1

Search Strategy used for a study "a scoping review of assessment tools for, magnitudes of, and factors associated with problem alcohol use: community-based studies," 2023.

A) PubMed/MEDLINE:

((((Prevalence [Title/Abstract]) OR "Prevalence" [Mesh])) AND (((alcohol* [Title/Abstract] OR "alcohol abuse" [Title/Abstract] OR "alcohol use" [Title/Abstract] OR "alcohol use disorder" [Title/Abstract] OR "alcohol dependence" [Title/Abstract] OR "alcohol consumption" [Title/Abstract] OR "heavy drinking" [Title/Abstract] OR "risk drinking" [Title/Abstract] OR "harmful drinking" [Title/Abstract] OR "hazardous drinking" [Title/Abstract] OR "binge drinking" [Title/Abstract])) OR ("Alcohol Drinking" [Mesh] OR "Alcoholism" [Mesh] OR "Binge Drinking" [Mesh]))) AND (((Ethiopia [Title/Abstract] OR community-based [Title/Abstract] OR "community based" [Title/Abstract] OR population-based [Title/Abstract] OR "population based" [Title/Abstract])) OR ("Ethiopia"[Mesh] OR "Health Surveys/epidemiology" [Mesh] OR "Population Health/epidemiology" [Mesh]))

B) EMBASE:

1. exp prevalence/
2. prevalence.ti. or prevalence.ab.
3. **1 or 2**
4. exp alcohol consumption/ or exp alcohol/ or exp alcohol abuse/
5. exp alcoholism/ or exp drinking behavior/ or exp binge drinking/
6. (alcohol$ or "alcohol abuse" or "alcohol use" or "alcohol consumption" or "binge drinking").ti. or (alcohol$ or "alcohol abuse" or "alcohol use" or "alcohol consumption" or "binge drinking").ab.
7. **4 or 5 or 6**
8. exp Ethiopia/
9. "community based".mp.
10. "population based".mp.
11. exp primary health care/

For peer review only

12. (Ethiopia or 'community based' or 'population based' or 'primary health care').ti. or (Ethiopia or 'community based' or 'population based' or 'primary health care').ab.

13. 8 or 9 or 10 or 11 or 12

14. 3 and 7 and 13

C) PsycINFO:

1. prevalence.mp.
2. prevalence.ti. or prevalence.ab.
3. exp "Alcohol Use Disorder"/ or exp Alcohol Abuse/ or exp Alcohol Drinking Patterns/
4. exp Binge Drinking/ or exp Drinking Behavior/ or exp Alcoholism/
5. (alcohol$ or "alcohol abuse" or "alcohol use" or "alcohol consumption" or "binge drinking").ti. or (alcohol$ or "alcohol abuse" or "alcohol use" or "alcohol consumption" or "binge drinking").ab.

6. 1 or 2

7. 3 or 4 or 5

8. ethiopia.mp.
9. "community based".mp.
10. "population based".mp.
11. exp Primary Health Care/
12. (Ethiopia or 'community based' or 'population based' or 'primary health care').ti. or (Ethiopia or 'community based' or 'population based' or 'primary health care').ab.

13. 8 or 9 or 10 or 11 or 12

14. 6 and 7 and 13

D) Global Index Medicus (GIM):

(tw:(prevalence)) AND (tw:(alcohol$ OR "alcohol abuse" OR "alcohol use" OR "alcohol consumption" OR "binge drinking")) AND (tw:(Ethiopia OR "community based" OR "population based" OR "primary health care"))

For peer review only

Supplementary File 2

Table 2: Prevalence, associated factors, and pattern of problematic alcohol use in high-income countries (HICs), 2023.

**Author, Year**<br><br>**Country/Location**	**Study Design & Study Setting (population)**	**Participants: Sample size** (Male subjects, %) Mean age (range) in years	**Tools (measures) or questions used**	**Outcomes: (Definition/nature of use)**	**Results & statistical methods used.**
**Aalto et al., 1999**<br><br>**Finland (town of Lahti)**	**Cross-sectional** PHC outpatients & General population (Urban residents)	PHC,2370 (40.3%)<br>OHC,3268 (29.3%)<br>GNP,544 (51.7%)<br>38-41(20-60) years	Quantity or frequency questionnaires **(QFQs)** (last 2 month) **CAGE**	**Heavy drinking:**<br>**Male**: ≥ 280g of absolute ethanol /24 drinks/week/ &/or ≥ 3 in CAGE.<br>**Women**: ≥ 190g/16 drinks per wk &/or ≥ 2 in CAGE.<br>**Abstinence:** no self-reported drinking at all & no answers to CAGE	**t-test & Chi-square analysis:**<br>**Men:** heavy drinking in PHC, OHC & GNP were 19.5%, 17.3% & 16.4%, respectively.<br>**Women:** corresponding figures were 8.6%, 6.2% & 12.9%.
**Aira et al., 2005**<br><br>**Finland (City of Kuopio)**	**Cross-sectional** home-dwelling elderly persons, **Community-based** (Urban residents)	700 persons (27.4% men)<br>81 (75-95.7) years	**QFQs** (1 year) & **CAGE**	**Four categories:**<br>Abstainers,<br>< 1 unit/week,<br>1–7 units/week, &<br>> 7 units/week.	**Chi-square & t-test** (frequencies vs means):<br>44% had used alcohol during past year (65% of men & 36% of women).<br>≥ 3 units/occasion used by 2.9% of women & by 11.7% of men.
**Andrews-Chavez et al., 2015**<br><br>**United States (Greater Boston area, MA)**	**Cross-sectional** (Puerto Rican adults, Hispanics). (Urban residents)	1472 adults (29.6% men)<br>? (45–75) years	**QFQs NIAAA** definitions **(NIAAA guidelines)**	**Lifetime abstainer (LA):** (< **12 drinks** in lifetime)<br>**Former drinker (FD):** **(> 12 drinks** in lifetime, but not currently drinking)<br>**Moderate drinker (MD):** (**Man/women**: ≤14/7drinks per week & ≤ 4/3 drinks/d)<br>**Heavy drinker (HD)**: (**Man/women**:>14/7drinks per week & > 4/3drinks/d)	**A multinomial logistic regression model:** 8% men & 39% women were **LAs;** 40% of men & 25% women **(FDs)**; **&** 21 % men & 8 % of women **(HDs).**<br>**Young men**: likely than older to be MDs.<br>**Women**: higher BMI, age, lower income & psychological acculturation (associated with abstention); age, lower perceived emotional support associated with increased FD; & women without v. with diabetes were more likely to be heavy drinkers.
**Bataille et al., 2003**<br><br>**France (Lille, Strasbourg & Toulouse)**	**Cross-sectional** (3rd MONICA) **Population survey** (Urban/Semi-urban & rural)	3508 subjects (51.0% men)<br>50.3 (35–64) years	Self-reported **QFQs** French alcohol consumption habits	**Heavy drinkers:**<br>**Men:** ≥ 60g ethanol/day, (6 glasses/d-any drink) &<br>**Women:** ≥ 30g/day (3 glasses/day)<br>**Reference class (RC):** non-drinkers & moderate drinkers together.	**Multivariate analyses:**<br>14% men & 40.8% women (non-drinkers) 9.0% women & 14.4% of men were HDs.<br>Low educational level, smoking, apoprotein B, HDL, MCV), GGT & CAGE score for men, & living area, age, MCV, GGT & the CAGE score for women were significantly associated with **heavy drinking (HD).**

**Coulson et al., 2010**<br><br>**Australia (south Eastern)**	**Cohort study** (Geelong Osteoporosis Study, GOS)<br>**Community-based cohort** (secondary data)	1420 men (100%)<br>56 (20 – 93) years	Validated self-report **FFQ**<br>**Mean daily alcohol intake** (Australian National Health & MRC 2009 guidelines)	**Consumption/12 months**: (never, < 1/month, 1–3 days/month, 1–6 days/week & every day<br>**Mean daily alcohol intake** non-drinkers/nil, > 0 but ≤ 2 drinks/ day, > 2 drinks/day **(with in past 12 months)**	**ANOVA & Multivariate analyses:**<br>Age-standardized proportion of non-drinkers was 8.7%, 51.5% consumed ≤ 2 drinks/day (≤ 20g/day), & 39.9% > 2 standard drinks per day (> 20g ethanol/day).<br>**Alcohol use (> 20g/day)** was positively associated with cigarette smoking, weight, higher SES & inversely with age & physical activity.
**Foulds et al., 2012**<br><br>**New Zealand**	**Cross-sectional** (Permanent private dwellers)<br>**Population survey**	12,488 adults (42.2% male)<br>? (≥ 15 years)	**AUDIT**	**Harmful/hazardous drinking (HHD):**<br>Score of ≥ 8 on AUDIT	**Crosstabs & logistic regression models:**<br>**HHD:** 17.7% (men, 25.6%; women, 10.4%);<br>**Overall, 9.4%** of attendees with HHD reported talking about alcohol.
**Geels et al., 2013**<br>**Netherlands**	**Cross-sectional** (All Netherlands Twin Register, NTR registered at a valid address)<br>**Population survey** (Urban)	16,587 subjects (36.5% men)<br>41.6 (18–97) years	**QFQs** (12 mo.)<br><br>Health Council of Netherlands recommended limit<br>CAGE & AUDIT	**Excessive alcohol use:**<br>**Women:** > 14 standard glasses per week<br>**Men:** > 21 drinks/week	**Linear/logistic/multinomial regressions:**<br>>30.0% of men & >20% of women drinking 6–7 times per week)<br>**Women:** 25–45 years had 5.7-5.9% of excessive drinking, & 55–65 years (15.5%)) Older age, sex (male), and initiation of cigarette & cannabis use were predictors of alcohol use
**Janghorbani et al., 2003**<br><br>**Hong Kong (China)**	**Cross-sectional** (Cantonese-speaking adult population)<br>**Population-based** (Urban)	2900 subjects (48.7% men)<br>45.8 (25–74) years	**QFQs** (weekly)	**Heavy drinkers:** men, > 400g & women, > 280g/wk<br>**Light drinkers:** men, < 168g & women, <112g/wk<br>**Moderate drinkers:**<br>Men: ≤ 400g/ ≥ 168g & Women: ≤ 280g/ ≥112g/wk<br>**Binge drinking**: ≥ 5 drinks in a row in the past month.	**GLMs/multiple/logistic regression models:**<br>**Mean weekly alcohol consumption**: 64.3g, men & 13.7g, women (P < 0.001).<br>Current drinking vs non-drinking, male sex, smoking (women), HDL, ≤ primary education, diastolic BP & separated/widowed were associated positively with **weekly ethanol consumption.**
**Kim et al., 2008**<br><br>**Hong Kong (China)**	**Cross-sectional** (All Hong Kong Chinese adults)<br>**Population based** (Urban)	9860 adults (50.0% men)<br>28 (18–70) years	Pattern **(QFQs)**<br><br>**CIDI** (Chinese version based on DSM-IV)	**Mean drinking/past year:** < once/wk, 1–3 times/wk, 4+ times/week)<br>**Binge drinking/past mo.** (5 servings of alcohol per one occasion in 30 days)<br>**Alcohol abuse or dependence** (Chinese CIDI)	**Stepwise multivariate logistic regression:**<br>**10.9% of entire sample** reported at least one of AUDs (AA, AD & binge drinking).<br>**Binge drinking :**14.5% in males (18.7% AA & 12.3% AD) & 3.5% in females (16.0% AA & 9.9% AD)<br>**Male binge drinkers** were less likely to be older & students but more likely to be employed in service industry.<br>**Female binge drinker**: less likely to be > 60 years or married & more likely to be smokers<br>**In both genders,** smoking was significantly associated with binge drinking

For peer review only

**(Chou et al., 2011**<br><br>**United States**	**Prospective study** (subsample of 3-year prospective study**; waves 1 & 2 of NESARC**)<br>**Population-based** (Urban)<br>**secondary data analyses**	13,442 analyzed (40.6% men)<br>? (≥ 50 years)	**QFQs**<br><br>**AUDADIS-IV** (DSM-IV)	**Binge drinking (BD):**<br>≥ 5 drinks/occasion (men)<br>≥ 4 per occasion (women)<br>**Current drinkers:** without BD<br>**Occasional BD:** < monthly in past year) &<br>**Frequent BD:** ≥ 1/month in past year<br>**DSM-IVAUDs** (Alcohol use, AA & AD)	**Multinomial & logistic regression:**<br>BD was 24.7% in men & 12.4% in females.<br>Overall, male respondents were significantly more likely to have BD.<br>Both men & women with occasional BD & frequent BD were significantly more likely than current male/female drinkers without BD to have **alcohol abuse disorder and alcohol dependence disorder (AUDs)**
**Latvala et al., 2009**<br><br>**Finland**	**Cross-sectional** (Finnish young adults)<br>**Population-based** (Urban)	605-diagnostic assessment done (sex unspecified)<br>28.6 (21-35) years	**SCID-I** complemented by medical record data	**Lifetime Substance Use Disorders (SUDs):**<br><br>DSM-IV diagnosis	**t-tests, X^2 tests & logistic regression:**<br>Lifetime AA or AD were 13.1% (19.8% for males & 6.3% for females). And total prevalence of AA & AD alone was 7.6% & 5.6%.<br>Behavioral, affective & parental factors, early initiation of substance use, learning difficulties & lower education were found to be associated with **alcohol & other SUDs.**
**Meyer et al., 2000**<br><br>**Germany (Northern, city of Lubeck)**	**Cross-sectional** of longitudinal project (Adult general population)<br>(Urban)	4075 analyzed (50.2% of men)<br>? (18 to 64 years)	**M-CIDI** (DSM-IV, adapted CIDI)<br>**Ever/current** QFQs	**Hazardous consumption:**<br>20-40g/d (women) &<br>30-60g/day (men) and<br>**Harmful consumption:**<br>> 40g/day (women) &<br>> 60g/d (men)<br>**AA or AD**:<br>DSM-IV Diagnosis (M-CIDI diagnostic software)	**Logistic regression analyses:**<br>Lifetime AUDs (4.5% AA, 3.8% AD) & men vs women for AA (8.1% vs 1.0%) & AD (6.0% vs 1.5%) respectively<br>**Hazardous & harmful consumption:** (13.2% lifetime; 6.0% in last 12-months)<br>**Male:** more affected by lifetime AUDs.<br>Association between AUDs & alcohol consumption pattern revealed a weaker relation for AA compared to AD.
**Miller et al., 2004**<br><br>**United States**	**Cross-sectional** (US Adults; BRFSS, telephone survey & NSDUH, an in-person survey)	**355,371** (BRFSS)<br>**87,145** (NSDU) were analyzed (sex unspecified)<br>? (≥ 18 years)	**Pattern (QFQs)**	**Binge drinking**: ≥ 5 drinks on an occasion	**two-tailed t-test:**<br>National binge drinking prevalence was:<br>14.7% for BRFSS and 21.6% for NSDUH<br>Most binge drinkers were male (74% BRFSS, 68% NSDUH) & white, non-Hispanic (73% BRFSS, 76% NSDUH)
**Mohler-Kuo et al., 2015**<br><br>**Switzerland**	**Cohort study** (Young Swiss men from C-SURF)<br>**Population-based** (Rural, 60.3%; Urban, 39.7%)	5943 total sample (100% men)<br>20.0 (18–25) years	DSM-IV & DSM-5 criteria<br><br>QFQs<br><br>RSOD & at-risk volume drinking	**AA & AD (**DSM-IV) &<br>**AUD (**≥ 2 criteria-DSM-5) (12-month prevalence)<br>**RSOD** (≥ 6 drinks/single occasion)<br>**At-risk volume drinking** (≥ 21 drinks/wk & RSOD at least monthly)	**Multinomial logistic regression:**<br>31.7% met DSM-5 AUD (21.2% mild; 10.5% moderate/severe], less than overall DSM-IV criteria for AA & AD (36.8%)<br>Relative to those meeting both DSM-IV & DSM-5 criteria, all other subgroups reported less alcohol and illicit drug use.

For peer review only

**Neumark et al., 2007**<br><br>**Israel**	**Cross-sectional**<br>(Israeli adults)<br>**National population-based survey**	4,859 adults<br>(49.0% men)<br>? (≥ 21 years)	**WMH-CIDI**<br>(lifetime & past 12-month DSM-IV Dx)	**DSM-IV** (AA & AD)<br>**Frequent drinking:** (3 ormore times in one week at least once) in the past year.<br>**Frequent heavy drinking:** consumption of ≥ 3 drinks, ≥ 3 times a week at least once during past year	**Logistic regression models:**<br>**Lifetime AD** was 41%,<br>**Frequent drinking was 5%, & frequent HD was** (6.8% of men & < 1% of women)<br>Lifetime AA/AD was 4.3% (4.0%, AA & 0.4% AD criteria)<br>Significantly higher rates among males (AOR=7.3), younger adults (AOR=5), immigrants (AOR=2.0) & never married (AOR=1.6)
**Proodfoot and Teeson, 2002**<br><br>**Australia**	**Cross-sectional**<br>(Australian National Survey of Mental Health & Wellbeing, NSMHWB)	10,641 respondents<br>(sex unspecified)<br>? (≥ 18 years)	**CIDI 2.1**<br>(modified WHO version)<br>**QFQs**	**DSM-IV Diagnosis for AA & AD**<br>**High level of dependence:** ≥ 4 criteria for dependence.	**Multiple logistic regressions:**<br>AD was 4.1% (males 6.1% & females 2.3%)<br>Variables correlated with AD were male sex, young age (18-34); not being in a married or de facto relationship & having any affective, anxiety or other substance use disorder.
**Veerbeek et al., 2019**<br><br>**Netherlands**	**Cohort study**<br>(Data from, NEMESIS-2<br>**Population-based**<br>(6 categories of urbanicity: very high to very low**)**	4618 persons<br>(sex unspecified)<br>? (23–70 years)	**CIDI V 3.0**<br>DSM-IV<br>International guidelines for alcohol use definitions	**Alcohol disorder:** AA &/or AD (past 12 months)<br>**Heavy alcohol use:**<br>> 14 drinks/wk (women) & > 21 drinks/wk for men	**Multinomial logistic regression analyses:**<br>Prevalence of heavy alcohol use was higher in older (55–70 years) than younger people (6.7% vs 3.8%), but alcohol disorder was less prevalent (1.3% vs 3.9%).<br>Heavy alcohol use was associated with higher level of education in older adults compared to younger adults.
**Williamson et al., 2003**<br><br>**United Kingdom**	**Cross-sectional**<br>(Subjects from 26 general practices registered with MRC-GPRF)<br>Community-based project in the UK<br><br>**Secondary data**	**20,062** unrelated index subjects<br>(40.0% men)<br>? (20–60 years)	**UK definition** for binge or heavy drinking behaviour &<br><br>**QFQs** for (single session drinking criteria)	**Binge/heavy session drinkers**: males > 8 & females > 6 units/session<br><br>**Non (binge/heavy session) drinking:** not fulfilling session drinking criteria, including abstainers	**No statistical analysis performed**<br>Average number of units of alcohol per week consumed was 16 for men and 8 for women.<br>17% of subjects had binge drinking fashion.<br>(15% for male vs 18% for females)<br>Binge drinking was found to be most prevalent amongst males & females in their 20s (33% of male vs 38% of females).
**Auchincloss et al., 2022**<br><br>**USA (**Philadelphia, Pennsylvania metropolitan area)	**Cross-sectional analyses**<br>(population-based cohort)<br>(Urban setting)	**772 (cross-sectional analyses)**<br>(48% men)<br>? (21–64 years)	Quantity/ Frequency Questions<br>**(QFQs)**<br>RSOD criteria	**BD** (SAMHSA definition): at least one day in past 30 days the person consumed a high volume of alcohol on a single occasion (≥5 alcoholic drinks for males and ≥ 4 for females).	**Logistic regression and Poisson regression**<br>Among alcohol users in either time period, 22% consumed 8 or more drinks per week and 37% reported at least 1 binge occasion in the past 30 days.<br>higher outlet density was associated with more alcohol consumption and residing farther from an outlet was associated with less alcohol consumption.
**Bott et al., 2005**<br><br>**Germany**	**Cross-sectional**<br>(part of a longitudinal study)	4,074 (analysis)<br>(44.9% men)<br>42.7 (18-64 years)	DSM-IV based Munich CIDI (M-CIDI).	**Four alcohol-use groups:**<br>(1) moderate drinkers/ abstainers (MOD/A): < 12	**Multinomial regression analysis (multivariate associations):**<br>9% of participants were at-risk drinkers.

For peer review only

(Lübeck city and its catchment area)	(urban setting)		**Quantity/ frequency index,QFI** (at-risk drinking = Based on the British Medical Association's, 1995, recommendati ons)	times in their lives or <20g/women & <30g/men pure alcohol/day **(2)** at-risk drinkers (ARD): >20/30g pure alcohol/day (3) DSM-IV criteria for alcohol abuse (AA) (4) DSM-IV criteria for alcohol dependence (AD)	Prevalence rates for at-risk drinkers were 16.9% for affective, 18.1% for anxiety and 17.8% for somatoform disorders. Compared with MOD/A, atrisk drinkers showed a 2-fold increased risk of having a psychiatric disorder. Subjects with AA showed a comparable level of risk & with AD showed an even greater risk. Female at-risk drinkers were twice as likely to have a psychiatric disorder compareed to male.
**Britton et al., 2020**<br><br>**United Kingdom**	**Cross-sectional** (part of Whitehall II study, civil servants at phase 11 (2012–13) (urban setting)	6117 (alcohol & sleep data) (70.9% men) Mean age: 69.4 men, 69.6 women (61–81 years)	**Volume of consumption** (drinks used in last 7 days) **Retrospective alcohol life-course grid** (AUDIT-C)	**Hazardous drinking/HD**: ≥ 5 points on AUDIT-C **Non-drinkers**: didn't drink alcohol in past year.	**Logistic regression:** 15.7% of men consumed 21 or more units per week compared to only 2.4% of women. 30.5% men & 12.8% women reported HD. men drinking > 21 units/wk or drinking hazardously were more likely to have disturbed sleep than those not drinking in past week or not drinking hazardously.
**Husberg et al., 2022**<br><br>**Norway (Tromsø)**	**Cross-sectional data** (population-based) (Tromsø 1-7, T7 = 2015-2016 (urban setting)	**19,185 (analysis)** (47.5% men) Mean age: 57.2 women, 57.4 men (40-96 years)	**AUDIT:** Hazardous alcohol use (HAU)	Hazardous alcohol use: AUDIT ≥ 8 as a cut-off	**Logistic binomial regression model:** Insomnia was more prevalent among participants with a HAU (24.1%) than without (18.9%). Participants who had HAU had higher odds of insomnia (OR= 1.49).
**Lee et al., 2020**<br><br>**Singapore**	**Cross-sectional** (Singapore Mental Health Study, SMHS 2016) (urban setting)	**6126 (interviewed)** (50% men) ? (18 yrs & above)	**QFQs** (alcohol use) **CIDI 3.0** (mental disorders) **DSM-IV** (diagnosis of mental disorders)	**Bing Drinking (BD):** consumption of 5 or more drinks (male) or 4 or more drinks (female) on a single occasion in the past 12 months.	**Multiple logistic regressions** 13.7% reported past-year BD (17.6% of males and 9.8% of females). Moderate associations between BD and mood and anxiety disorders (ORadj =1.8–4.4), were noted, while associations with AUDs were much stronger (ORadj=5.3–9.7). Associations betweenn BD & anxiety disorders were observed exclusively in females (ORadj=2.3–3.3). Binge drinkers reported a lower quality of life compared to their non-binging counterparts.
**Lindstrom et al., 2020**	**Cross-sectional**	11,716 (50.4% men) ? (65-99 years)	AUDIT-C (Alcohol consumption)	non-drinker = 0; moderate drinker = 1–7 (male), 1–5 (female); risk-drinker = 8–12 (male), 6–12 (female).	**Logistic regression analysis** Men (83%) were more prone to drink alcohol compared to women (71%). The prevalence of risk drinking was about 2% for both genders.

For peer review only

**Sweden**				Non-drinker was not consumed alcohol during the last 12 months.	Alcohol consumption declined with age. Moderate consumption of alcohol was associated with lower probability of poor SRH compared to non-drinking (AOR=0.64 for men) and (AOR= 0.68 for women).
**Lundin et al., 2021**<br><br>**Sweden** (Gothenburg, second largest city in Sweden)	**Longitudinal** (Women and Alcohol in Gothenburg (WAG) Study, cohort in 1986, 1994/2000 & 2013) (urban setting)	**1,614 (baseline)** (100% women) ? (across different age-group?)	CIDI-SAM, ICD-10 & ICD-1, DSM-IV & DSM-5	AUD, alcohol abuse (AA), alcohol dependence (AD) based on CIDI-SAM or (DSM-III, DSM-III-R, DSM-IV, DSM-5, & ICD-10 & ICD-11)	**contingency tables & Cohen's Kappa coefficient (κ)** Baseline: prevalence of lifetime AD was 10.6 % (ICD-11); 4.0 % (ICD-10); 4.3 % (DSM-IV); 7.5 % (DSM-III-R); and 12.3 % (DSM-III). DSM-5 AUD was 14.3 %.
**Mason-Jones and Cabieses, 2015**<br><br>**Chile**	**Cross-sectional**<br><br>(Chilean National Health Survey 2010, ENS 2010) (88% lived in urban settings)	Adolescents (absolute n=435, weighted n = 1860812) Young adults (absolute n = 412, weighted n = 1386 547) (50.3% men) ? (adolescents 15-20 years & young adults 21-25 years).	**QFQs** (Alcohol prevalence in last year, & BD prevalence in last month)	**Alcohol prevalence in last year:** 'yes' labeled as "1" and 'no' labeled as "0". **BD prevalence last month:** had drunk four or more units of alcohol in a single episode in the last 4 weeks.	**Conditional logistic regression models:** 65% of adolescents and 85% of young adults reported drinking alcohol in the last year & of those (who used alcohol in the last year) 83% of adolescents and 86% of young adults reported BD in the previous month. Adolescents who reported binging alcohol were also more likely, compared to young adults, to report being depressed (OR 12.97) or to feel very anxious in the last month.<br>Adolescent females were more likely to report poor life satisfaction in the previous year (OR 8.50), feel depressed (OR 3.41).<br>Being female was also associated with a self-reported diagnosis of depression for both age groups.
**Mondi et al., 2022**<br><br>**USA (Chicago)**	**Cross-sectioal**<br><br>(CLHS data collection, predominately Black sample) (grew up in urban poverty)	**301** CLHS participants (40% men) ? (32-37 years invited to CLHS)	**M.I.N.I. 7.0.2.** (based on DSM-IV & ICD-10 criteria)	DSM-IV & ICD-10 criteria for major depressive disorder, generalized anxiety disorder, post-traumatic stress disorder, substance use disorder, and AUD.	**Independent samples t-tests** Males endorsed significantly higher rates of any AUD within the past 12 months (38.3%) than females (20.6%). Probable prevalence rate for any AUD was 27.7%.
**O'Dwyer et al., 2019**<br><br>**Ireland**	**Cross-sectional** (Data generated from 2013 National Alcohol Diary Survey, NADS)	**4338 drinkers** (49.9% men) ? (18–75 years old)	**RSOD** criteria (HED) **DSM-IV (CIDI) Alcohol-related**	**HED:** consuming 60 g or more of pure alcohol in a single drinking occasion. **Alcohol dependence** (AD) (DSM-IV criteria) Current drinkers, non-	**Crosstabs (Pearson χ2, bivariate assoc.)** There was a relatively even breakdown of low-risk (31.0%), occasional HED (30.6%), and monthly HED (31.5%) drinkers. AD constituted 6.9% of all drinkers. Overall, 29% of drinkers experienced at least one harm

For peer review only - http://bmjopen.bmj.com/site/about/guidelines.xhtml

			**harms/ARH (8 questions)**	drinkers, monthly HED, occasional HED, low-risk drinkers, ARH	from their own drinking in last year. Respondents who were AD had a greater individual risk of experiencing each harm.
**Shockey and Esser, 2020** **USA** (District of Columbia and territories)	**Cross-sectional** (U.S. employed adults who resided in 32 states, BRFSS data)	**358,355 employed adults** (48% men**)** ? (18-55 years)	Industry & occupation (I&O) optional module BRFSS & **QFQs**	**BD**: men consuming ≥ 5 drinks or women consuming ≥ 4 or more drinks, on an occasion.	**No statistical analysis performed.** 20.8% reported BD, with an average of nearly 49 times per year and an average intensity of 7.4 drinks per binge episode, resulting in 478 total binge drinks per binge drinker. The adjusted BD prevalence ranged from 15.9% among community and social services workers to 26.3% among construction and extraction workers.

Abrevations: **AA:** alcohol abuse; **AD**: alcohol dependence; **ARH**: Alcohol Related Harm; **AUD:** Alcohol Use Disorder; **AUDADIS-IV**: Alcohol Abuse and lcoholism's Alcohol Use Disorder and Associated Disabilities Interview Schedule– DSM-IV Version; **AUDIT**: Alcohol Use Disorder Identification Test; **BD**: Binge Drinking; **BRFSS**: Behavioral Risk Factor Surveillance System; **CLHS**: Chicago Longitudinal Health Study; **C-SURF**: Cohort Study on Substance Use Risk Factors; **FFQ**: Food Frequency Questionnaire; **GLM**: General linear models; **GNP**: General Population; **GPRF**: General Practice Research Framework; **HAU:** Heavy alcohol use; **ICD-10/11:** International Classification of Diseases 10th/11th Revision; **MONICA**: MONItoring of trends and determinants of CArdiovascular disease; **NIAAA**: National Institute on Alcohol Abuse and Alcoholism; **NRR**: Non response rate; **wk**: week; **NSDUH**: National Survey on Drug Use and Health; **OHC**: Occupational Health Care clinic; **PHC:** Primary Health Care clinic outpatients; **QFQs**: Quantity Frequency Questionnaires of alcohol use; **RSOD**: Risky Single-Occasion Drinking; **SAMHSA**: Substance Abuse and Mental Health Services Administration; **USA:** United States of America; **yr.:** year; ?: mean age is not mentioned.

For peer review only

Supplementary File 3

Table 3: Prevalence, associated factors, and pattern of problematic alcohol use in low-and middle-income countries (LMICs), 2023.

Author, Year<br><br>Country/Location	Study Design & Study Setting<br><br>(population)	Participants: Sample size (Male, %) Mean age (range) in years	Tools (Measures) or questions used	Outcomes: (Definition/nature of use)	Results & statistical methods used:
**Andersson et al., 2018**<br><br>**South Africa**<br>(Eastern Cape Province)	**Cross-sectional**<br>(Nelson Mandela Metropolitan & Sundays River Valley City)<br>Population-based<br>(Urban/semi-urban/ rural setting)	1000 participants (52% of men)<br>27 (18-40) years	**M.I.N.I. 6.0 (DSM-IV)**	**Alcohol dependence & Alcohol abuse (AD/AA):** (DSM-IV diagnosis during the past 12 months)	**χ2 statistics & logistic regression models:**<br>AD: 26.5% (39.0% men & 19.1% women)<br>AA: 9% (19.0% for men & 6.0% for women).<br>**AD:** higher in rural/semi-rural in men (43.1%) and women (26.8%) than in urban/semi-urban.<br>Widowed and separated women compared to married or cohabiting and women with low income (don't want to disclose) compared to weekly household income of ≥ 1,001 RAND remained statistically significant.
**Burazeri and Kark, 2010**<br><br>**Albania**<br>(Tirana)	**Cross-sectional**<br>(transitional post-communist Albania (Muslim, 68.5%)<br>Population-based	685 individuals (65.7% of men)<br>52.6 (35–74) years	Quantity/ frequency questionnaires **(QFQ**s)<br>(patterns questions)<br>(12 months)	**Drunkenness/hangovers:** never, very exceptionally, 2-3 times/year, 1/month, 1/fortnight & once/week). Composite Binging score: drunkenness or hangovers during w/c ≥3 units (≈60g of ethanol) consumed	**Binary/multivariable logistic regression:**<br>10.3% of men had ≥ 2-3 annual episodes of drunkenness & and hangovers each.<br>**Women**: both markers of binging, 1.4%<br>**Men:** 8.9% drinking ≥ 60g alcohol/session.<br>Binge drinking was related to low educational level, financial loss in pyramid collapse, & religiosity (inversely) in both Muslims and Christians (all in men).
**Dias da Costa et al., 2004**<br><br>**Brazil**<br>(Rio Grande do Sul State)	**Cross-sectional**<br>(Adults of municipality of Pelotas)<br>Population-based<br>(Urban area)	2,177 adults (43%)<br>41.6 (20-69) years	**QFQs**<br>(weekly use)	**Moderate consumption:** up to 30g/day of ethanol)<br>Heavy consumption or hazardous drinking, HD: ≥ 30g/day of ethanol/week	**Non-conditional logistic regression:**<br>Moderate consumption was 65.1%<br>**HD:** 14.3% (29.2%, men & 3.7% in women).<br>Men, elders, blacks, low SES, heavy smokers, & chronic disease presented higher prevalence of HD. Men with minor psychiatric disorders had higher prevalence of HD & in women (association between age & HD was inversely related).

For peer review only - http://bmjopen.bmj.com/site/about/guidelines.xhtml

**Ji et al., 2018**<br><br>**China** (Xuzhou city, Jiangsu)	**Cross-sectional**<br>(11 regions in Xuzhou city)<br>Population-based (urban/rural areas)	36,157 participants (48.40% of men)<br>45.5 (18-75) years	**MAST**	**Alcohol dependent (AD):** MAST score of ≥ 5<br>0 (no alcohol dependence)<br>1–4 (low AD), 5–6 (light AD), 7–25 (mild AD), 26–39 (moderate AD & 40–53 (severe AD)	**χ2 &, t-tests; multivariate log. Regression:**<br>**AD:** 11.56% (22%, males & 1.74%, females)<br>Newly detected hypertension rate was 9.46%<br>Significant associations were found between AD & blood pressure.<br>AD was positively correlated with systolic blood pressure & diastolic blood pressure ($r = 0.077$, $P< 0.01$).
**Mendoza-Sassi and Beria, 2003**<br><br>**Brazil**	**Cross-sectional**<br>(Residents in municipality of Rio Grande, Southern Brazil)<br>Population based (Urban population)	1260 people (46.1% of men)<br>40.3 (15-94) years	**AUDIT**<br><br>**SRQ-20**	**Alcohol Use Disorder (AUD):** AUDIT score ≥ 8	**Log. regression in multivariate analysis:**<br>**AUDs:**7.9% (2.5%, women & 14.5%, men).<br>Risk of alcohol misuse increased across increasing social class (P linear trend = 0.03)<br>Males had OR=6.89 compared with women.<br>Smokers (OR 3.27) & ex-smokers (OR 1.30) were at higher risk than non-smokers.<br>Those with minor psychiatric disorders had a 2.48 OR of presenting a positive test (AUD).
**Moreira et al., 1996**<br><br>**Brazil**<br>(Porto Alegre)	**Cross-sectional**<br>(Adult population of Porto Alegre, Southern Brazil)<br>Population based (Urban)	1,091 individuals (45.0% of men)<br>Mean age: 41/men; 44/women<br>(≥ 18) years	**CAGE** & Type & **QFQs** of alcoholic drink	**Heavy drinking (HD):** Average of ≥ 30g/day<br>**Alcohol dependence/AD:** Two positive answers to the CAGE questionnaire	**X^2-test & logistic regression models:**<br>**AD** was 9.3%; **heavy drinking** was 15.5%.<br>Increasing age, lower education & income, non-white race (associated with HD & AD).<br>Households with 3-4 persons were associated with lowest risk HD, but AD was higher in crowded households (5-11). Presence of one with HD/AD in household was associated with HD but not with AD.
**Peltzer et al., 2011**<br><br>**South Africa**	**Cross-sectional**<br>(Part of SABSSM 2008 survey)<br>(62.5% located in urban areas)	13,828 persons (43.7% of men)<br>? (≥ 15 years)	**AUDIT**	**Binge drinking (BD):** Females (4) & males (5) standard drinks/occasion<br>**Hazardous or harmful drinking:** AUDIT cut-off score ≥ 8	**Adjusted logistic regression:**<br>**Risky (hazardous/harmful drinking):** 9% (17% among men & 2.9% for women)<br>Overall prevalence of BD: 9.6%<br>**Men:** risky drinking was associated with 20-54 years than 15-19; Colored population group; lower (economic status & education.)<br>**Women:** risky drinking was associated with urban residence, Colored population group; lower education; and higher income
**Peltzer and Phaswana-Mafuya, 2013**<br><br>**South Africa**	**Cross-sectional**<br>(older South Africans, Study of Global Ageing &	2144 participants (41.1% of men)<br>? (> 60 years old)	**QFQs** & **NIAAA** risky drinking criteria	**Risky drinking (2 ways):**<br>**Heavy drinkers:** (>7 drinks per week) &<br>**Binge drinkers:**	**Multivariate logistic regression:**<br>**Heavy & binge drinking**: 4% vs 3.7%<br>Male gender, white population group; tobacco use & being obese were associated with risky drinking.

For peer review only - http://bmjopen.bmj.com/site/about/guidelines.xhtml

	Adults Health, SAGE in 2008) Population-based (Urban, 63.2%)			(>3 drinks/one occasion at least weekly)	Hypertension, diabetes, and depression were not associated
**Peltzer et al., 2012**<br><br>**South Africa**	**Cross-sectional** (South African Youths, Black, 97.5%; 4 of 9 provinces in SA) Population-based	3123 participants (54.6% of men) 20.5 (18-24) years	**AUDIT-C** (Frequency of drinking, quantity consumed per occasion & frequency of HED)	**HED:** consumption of five standard drinks (≥ 60g) alcohol per single occasion **Binge drinking:** women (4) & men (5) units in a session at least/month **Hazardous or harmful drinking (HHD):** ≥ 5 on AUDIT-C	**Unconditional multivariable log. Reg.:**<br>**HHD:** 19.1% (24.3%, male; 12.9%, women)<br>**Men:** high sexually permissive attitudes, not poor, multiple sexual partners, tobacco & illicit drug use were associated with HHD.<br>**Women:** high (HIV risk perception, sexually permissive attitudes & peer pressure (lifestyle), spending more nights away in a week, tobacco & illicit drug use were associated with HHD.
**Tomkins et al., 2007**<br><br>**Russia** (Izhevsk)	**Cross-sectional** (Men controls in a case-control study of premature male mortality, Izhevsk) Population-based (Urban)	1750 men (100% men) ? (25-54 years)	**QFQs**	**Hazardous drinking-HD:** (any of these in past year) Having drunk surrogates; having been on zapoi; having frequent hangovers (once/month or more); having drunk spirits daily.	**Logistic regression:**<br>Drinking spirits (79%) & surrogates (8%) at least sometimes in the past year.<br>Drinking spirits (25%) & surrogates (4%) at least weekly & 10% had had episode of zapoi in past year.<br>Education, lowest level in men (associated with indicators of HD.<br>Indicators HD were also associated with being unemployed & levels of household wealth/amenities.
**Weiser et al., 2006**<br><br>**Botswana**	**Cross-sectional** (5 districts of Botswana with highest number of HIV-infected individuals) Population-based (Urban/Rural)	1,268 adults (48% men) 28.8 (18-49 years)	**QFQs**	**Heavy alcohol consumption (HD):** > 14 drinks/wk for women, & > 21 drinks/wk for men) **Problem drinking** (8–14, women, 15–21 for men) &	**Heavy drinking:** 31%, men & 17%, women<br>**Problem drinking:** 39% of men, (79% met HD) & 25 % of women, (69% met HD). Correlates of HD: intergenerational relationships (age gap 10 year), male gender, higher education, & living with a sexual partner. A dose-response relationship was seen between alcohol use & risky sexual behaviors, with moderate drinkers at lower risk than both problem & heavy drinkers.
**Zavos et al., 2015**<br><br>**Sri Lanka** (Colombo district)	**Cross-sectional** (Data from the Colombo Twin And Singleton Study, CoTASS) Population based (Urban/semi-urban areas)	6014 Sample (twins/48% & Singleton/46% of male) Mean age: 34 (twins) & 43 (singleton) (> 16 years)	**CIDI** Alcohol use: ever had of 12 drinks at any time in life	**Alcohol abuse & dependence:** Definition of CIDI (DSM-IV criteria)	**Robust cluster command:**<br>**12-month prevalence of alcohol use:** 22.7%<br>**Lifetime AA & AD in men:** 6.2% & 4.0%<br>Lifetime AA & AD was associated with greater prevalence of nicotine dependence, depression, anxiety & PTSD (only for AD).<br>Lower standard of living was associated with alcohol use & AD but not with AA

For peer review only

**Lo et al., 2013**<br><br>**Kenya**<br>(Nyanza Province)	**Prospective study**<br>(Longitudinal database of demographic & health census data in western Kenya)<br>Population-based<br>(Rural area)<br>Secondary data	72,292 individuals<br>(43.1% men)<br>? (≥ 18 years)	Questions on (ever use & current use)	**1) % of time drunk when drinking in past 30 days:** (Did not get drunk, Drunk < 50%, Drunk 50%+)<br>**2) Days drinking/month:** (1-7, 8-17 & 18+)<br>**3) Problem drinking:** drinking ≥ 8 days/past 30 days & were drunk at least 50% of times they drank	**Crude and adjusted logistic regression:**<br>Overall, ever drinking was 20.7%<br>Drinking/past 30 days was 7.3% & 34.6%.<br>(60.3%, being drunk on ≥ 50%+) of all drinking occasions)<br>Alcohol use increased with decreasing socio-economic status & oldest women.<br>Current smoking, men, all age groups ≥ 40 & highest wealth index quintile (significantly associated with problem drinking).
**Pillai et al., 2013**<br><br>**India**<br>(Northern Goa)	**Cross-sectional**<br>Population-based survey<br>(rural & urban communities)	2641 men<br>(100% men)<br>? (18-49 years)	**QFQs** &<br>Drunkenness	**Current drinkers:**<br>**low risk** (< 40 g/d),<br>**medium risk** (40–60 g/d),<br>& high risk (> 60 g/d)<br>**HED**: ≥ 60g in a single occasion in past 12 months<br>**Drunkenness**: times drank to feel drunk in last 1 year (< monthly, ≥ monthly but < weekly), & ≥ weekly)	**Logistic regression + Moderating effect:**<br>Of current drinkers:<br>**HED:** 28.6 % (rural 31 %; urban 27.2 %) & Drunkenness: 33.7% (rural 30.5 %; urban 35.5 %) ➔ monthly or more frequent<br>**HED**: associated with older age, being separated, lower education, & LSI<br>Weekly or more frequent drunkenness was associated only with rural residence.<br>All three risky drinking patterns were associated with CMDs, sexual risk, intimate partner violence, acute alcohol-related consequences, & AD.
**Sau, 2017**<br><br>**India**<br>(West Bengal)	**Cross-sectional**<br>(Adult population of the state of West Bengal, Gram Panchayat, GP)<br>Community based	99 adults<br>(54.5% men)<br>38.62 (≥ 18) years	**AUDIT**	**AUDIT (WHO scoring):**<br>≥ 8 (hazardous/harmful use & possible AD)<br>0-7 (Zone-I): Low risk drinking/abstinence risk<br>8-15 (Zone-II): Alcohol use in excess of low-risk,<br>16-19 (Zone-III): Harmful & hazardous drinking &<br>20-40 (Zone-IV): Alcohol dependence risk level.	**Intraclass correlation, chi-square test, logistic regression & Bootstrapping:**<br>**Mean AUDIT** score was 7.11 (5.55 to 8.74)<br>**Low risk drinking/abstinence:** 65.5% & **Alcohol use in excess of low risk:** 17.6%, & **Harmful & hazardous drinking:** 8.5% & **Alcohol dependence** was 8.4%<br>**Hazardous, harmful use & AD** was 34.5%<br>Male gender and being employed were more prone to become high risk level drinker.
**Takahashi et al., 2017**<br><br>**Kenya**<br>(Western)	**Cross-sectional**<br>(Adults residing in Ikolomani Sub-county, Kakamega)<br>Community-based	478 participants<br>(41.4% men)<br>41 (18–65) years	**AUDIT**<br><br>**Type & QFQs**	**Current drinkers:** use of any alcohol in the last month,<br>Hazardous/high-risk drinkers:	**Univariate & multivariate analyses:**<br>Current & hazardous/high-risk alcohol use: 31.7% (men 54.6%; 8.9%, women) vs 28.7%<br>More than one drinker in the family, ≥ 5 drinker friends & positive attitude towards alcohol intake were positively associated with

For peer review only - http://bmjopen.bmj.com/site/about/guidelines.xhtml

	(Rural)			AUDIT score of ≥ 8	current alcohol drinking status, and with hazardous/high-risk alcohol consumption.<br>Women were less likely to be current drinkers & hazardous/high-risk drinkers.
**Yeung et al., 2015**<br><br>**Cambodia**<br>(Puok district)	**Mixed methods**<br>(Adults living in 2 selected rural communities<br>Community based<br>Rural communities	120 households<br>(49.0% men)<br>? (≥ 18 years)	**AUDIT-C-Q**<br>**QFQs**<br>8 FGDs<br>**NIAAA**<br>Guidelines	**AUD:** cut off score of ≥ 5 in men & ≥ 4 in women<br>HED: ≥ 6 drinks in a single sitting at least monthly (NIAAA)	**χ2, Welch 2-sample t-test, Log. Regression**<br>**AUD & HED:** 4% and 31%, respectively.<br>**AUD** (47% men, 5% women (P < 0.0001); **HED** (47% men, 15% women (P = 0 .0001).<br>Male sex, younger age (decreasing age), and increasing income (higher monthly) were significant risk factors for AUD and HED
**Alem et al., 1999**<br><br>**Ethiopia**<br>(Butajira)	**Cross-sectional**<br>(Demographic surveillance site)<br>Community-based<br>(mostly rural)	12531 residents<br>(50% male)<br>? (≥ 15 years)	5-item questionnaire (questions for alcohol user vs non-users & **GAGE-4** items)	**Problem drinking (PD):** consumption beyond safe limits (≥ 2 positive responses on CAGE).<br>Cigarettes smoked daily: 1-3=mild,<br>4-9=moderate,<br>>9= heavy	**Chi-square statistics:**<br>Current drinkers: 23.4 % (15% women & 36% for men).<br>PD, 15.7% in alcohol users; overall PD, 3.7% (7.5% men & 0.90% women).<br>(2.4% in urban dwellers & 4.0% in rural)<br>Christian religion, male sex, ethnically non-Gurage, & smoking (associated with PD in both sexes). Marital status (divorced men), mental distress & income were associated with PD only in men & being widowed & divorced in women
**Kebede and Alem, 1999**<br><br>**Ethiopia**<br>(Addis Ababa)	**Cross-sectional**<br>Adults in Addis Ababa<br>Population based<br>(Urban residents)	10203 adults<br>(45.1% men)<br>? (≥ 15 years)	**CAGE**<br>(1st stage) &<br><br>**CIDI**<br>(2nd stage)	**Problem drinking (PD):** ≥ 2 of on CAGE items, & **Alcohol dependence (AD):** CIDI (ICD-10 diagnoses)	**Bivariate and multivariate analysis:**<br>**PD** was 2.7%, lifetime AD, 1.0% (1.9% in male & 0.1% for women) & one-month AD, 0.8% (1.5% for men and 0.06% for women).<br>PD increased with increasing age<br>PD decreased with increasing educational attainment. 39% increased risk of PD with employment & female sex had a 96% decreased risk of PD. Only sex (women had an 84% less risk to be AD compared to men).
**Nalwadda et al., 2018**<br><br>**Uganda**<br>(Kamuli District)	**Cross-sectional**<br>(Men attending PHC & men in population; part of the PRIME project)<br>Community-based & facility-based<br>(Rural district)	351 men<br>(Community study)<br>778 men<br>(Facility Survey)<br>(100% men)<br>? (≥ 18 years)	**AUDIT**<br>(10 item)	**AUD definition (AUDIT):**<br>**Hazardous** (score 8–15), **Harmful** (score 16–19) or **Dependent** (score ≥ 20) drinking behaviors (cut-offs defined by WHO)	**Kruskal–Wallis test & Fisher's exact test:**<br>**Community study**: 4.1% of all men were AUDIT+ (AUD); (2.9% hazardous, 0.7% harmful & 0.5% with dependent drinking)<br>**Facility study:** 5.7% of all men were AUDIT+; (4.5% hazardous; 0.6%, harmful)<br>47.5% AUDIT+ men: AUD ruined their lives<br>55.0% AUDIT+ men did not seek treatment<br>AUDIT scores were higher among older men, men with paid/self-employment status and higher PHQ-9 score (P < 0.05).

For peer review only - http://bmjopen.bmj.com/site/about/guidelines.xhtml

**Rathod et al., 2018**<br><br>**Nepal**<br>(Central district)	**Cross-sectional**<br>(Adults in Chitwan District; part of PRIME consortium)<br>Population based<br>Secondary analysis	3482 sample<br>(36% men)<br>? (18-88) years	**AUDIT**<br>(10-item)	**Abstinent:** Score of 0,<br>**Recent (12 months) consumer:** Score of ≥ 1<br>Score of ≥ 8: **positive screen for AUD,**<br>8–15: **hazardous** drinking,<br>16-19: **harmful** drinking &<br>≥ 20: **dependent drinking**	**X^2 test & Negative binomial regression:**<br>23.8% of male screened AUD+ (AUD)<br>5.3% of female drinkers screened AUD+<br>Men with AUD, 38% spoke to another person about their problems & 80% had internalized stigma.<br>Being a drinker was associated with age, religion, caste, education, occupation & tobacco use. AUDIT scores were associated with age, caste, marital status, occupation, tobacco use, depression, functional status & suicidal ideation.
**Teferra et al., 2016**<br><br>**Ethiopia**<br>(Sodo district, southern Ethiopia)	**Cross-sectional**<br>(Adults from rural Sodo district (PRIME survey)<br>Community based<br>(Rural residents)	1500 adults<br>(50.5% men)<br>? (≥ 18 years)	**FAST**<br>**Kessler-10** (psychological distress)<br>**LTE** (adverse life events)	**Hazardous alcohol use (HD):**<br>FAST score ≥ 3 out of 16	**Exploratory multivariable log. regression:**<br>Prevalence of **hazardous alcohol use**: 21%; (31% in males & 10.4 % in females)<br>Factors associated with HD were being male, increasing age, having experienced ≥ 1 stressful/adverse life events, & severe psychological distress (AOR = 2.96).<br>High social support was protective from hazardous alcohol use (AOR = 0.41)
**Zewdu et al., 2019**<br><br>**Ethiopia**<br>(South, Sodo district)	**Cross-sectional**<br>(Adults who lived for at least 6 months in Sodo dist)<br>Community-based<br>(Rural district)	1485 individuals<br>(45.7% men)<br>39 (≥ 18) years	**AUDIT-10**	**Probable AUD:** score ≥ 8<br>8–15 (medium level of alcohol problem)<br>≥ 16 (high level of alcohol problems)<br>≥ 20 (possible alcohol dependence-AD)<br>**Binge drinking (BD):** drinking ≥ 6 alcoholic drinks on a single occasion	**Poisson regression with robust variance:**<br>Weighted prevalence of AUD was 13.9%; 25.8% in men & 2.4% women, P < 0.001<br>(Hazardous/harmful/AD: 9.9%/2.2%/1.8%)<br>23.3% had BD<br>87.0% of cases scored ≥ 16 had never sought help & 70.0% had high internalized stigma<br>AUD were associated & more prevalent in men (aPR = 7.7), farmers, traders, & daily laborers. People with AUD had increased total depressive symptom score & higher total disability score, more stressful life events & suicidal ideation (aPR 1.5)
**Getachew et al., 2017**<br><br>**Ethiopia**	**Cross-sectional**<br>(2015 national noncommunicable diseases STEPS survey)<br>Community based<br>(Urban,27.4% & rural, 72.58%)	9,800 participants<br>(40.6% men)<br>34.5 (15-69) years	**QFQs**<br>(WHO STEPS questionnaire)	**Current drinkers**: alcohol use a month before survey<br>Lifetime alcohol use: ever<br>**Past 12-month users:**<br>HED/Excessive Alcohol Consumption: drinking ≥ 6 drinks in men & ≥ 4 in women on one occasion.	**Logistic regression:**<br>Prevalence of lifetime alcohol consumption & current drinkers was 49.3% & 40.7%.<br>Among ever drinkers, 89.6% drank alcohol in the past 12-months.<br>**HED:** 12.4% (20.5% males & 2.7% females)<br>Factors independently associated with HED, were male sex, rural residence), married, and current tobacco smoking (AOR=2.87).
**Abd Rashid et al.,**	**Cross-sectional**	**363 participants**	**AUDIT**	**Hazardous alcohol use:**	**Multiple logistic regression analysis**

For peer review only - http://bmjopen.bmj.com/site/about/guidelines.xhtml

**2021**<br><br>**Malaysia** (Sabah Borneo Island)	(People in Bingkor who consumed alcohol in the past 12 months)<br>(urban setting)	(51.5% men)<br>? (≥ 26 years old, 90.6%)	(hazardous alcohol use)<br>**MINI V5.0** based on DSM-IV (psychiatric morbidity)	AUDIT scores of ≥8	80.2% admitted having consumed alcohol.<br>Preferred type of drink: beer (67.8%), tuak tapai (61.7%), wine (31.7%), tuak beras and whisky (16.8%), imported alcohol drinks such as vodka (9.1%) and 'samsu' (3.9%).<br>41% of participants (high risk for hazardous alcohol use) vs 39.1% (with low risk of hazardous alcohol use).<br>Being male & being a non- Muslim had a higher risk to develop hazardous alcohol use (OR = 3.313 & 3.834 respectively).<br>Having a current obsessive- compulsive disorder was associated with a higher risk of hazardous alcohol use (OR = 0.265).
**Assanangkornchai et al., 2020**<br><br>**Thailand**	**Cross-sectional**<br><br>(Thailand's 5th National Health Examination Survey, NHES-5, 2014)<br>(urban/53.6%, rural/46.4%)	**13177 participants**<br>(49.2% men)<br>46.7 (> 20 years)	**AUDIT** (for AUD)<br>**MINI, Thai version 5.0.0** (for MDE)	**AUD**: non-problem drinkers (0–7), hazardous drinkers (8–15), and harmful-dependent drinkers (16– 40) on AUDIT<br>**MDE**: defined according to DSM-IV criteria	**Multinomial logistic regression:**<br>10.3% and 1.9% hazardous drinkers and harmful-dependent drinkers, respectively<br>2.5% met the criteria for MDE in the past 12 months before the survey.<br>Approximately 20% were current smokers.<br>Associations between MDE and either hazardous (HD) or harmful dependent drinking (HDD) were strongest among those in third tercile (highest/wealthiest) of wealth index, first tercile (lowest/poorst), secondary school level of education or above, living in urban areas, & those who are employed.
**Ding et al., 2020**<br><br>**China**	**Cross-sectional**<br>(China Health and Retirement Longitudinal Study, 2011– 2012)<br>Community based (Urban/40.5%, Rural/59.5%)	**17,302 subjects**<br>(49.30% men)<br>59.67 (aged ≥ 45 years)	**QFQ**<br>(for alcohol use)	**Heavy drinking:** >14 drinks per week (males) & >7 drinks per week for females	**Binary & multinomial logistic regressions**<br>Overall prevalence of heavy drinking, obesity, current smoking, and physical inactivity were 7.23%, 11.53%, 27.46%, and 44.06%, respectively.<br>Compared with healthy subjects (no hypertension, high cholesterol, or diabetes), newly detected hypertensive patients were more likely to smoke (OR, 1.34), be heavy drinkers (1.45), and be obese (1.94).
**Hernandez-Vasquez et al., 2022**<br><br>**Peru**	**Cross-sectional**<br>[(2018 Peruvian Demographic & Family Health Survey (ENDES)]<br>**A Population-Based Analysis**	**32,020 people (analysis)**<br>(42.8% men)<br>? (≥ 18 years old)	**SAMHSA definition (RSOD):** Bing Drinking (BD)	**BD:** consumption of 5 & 4 or more alcoholic beverages on the same occasion for men & and women, respectively, in the last 30 days before the survey	**Poisson's family GLMs with link function (log) were used for (cPR and aPR).**<br>BD was found in 22.4%. Men (32.6%) presented a higher consumption pattern than women (12.8%).<br>Men aged 25–44 had a higher probability of BD (aPR: 1.28). The age group of ≥ 60 was associated with a lower probability (aPR: 0.70) of BD compared to younger group of men (18-24 years).

For peer review only

For peer review only - http://bmjopen.bmj.com/site/about/guidelines.xhtml

	(Urban/65.7%, Rural/34.5%)				Women aged ≥ 60 years was associated with a lower probability of BD (aPR: 0.24). Secondary (aPR: 2.01) or higher level of education (aPR: 2.04) was a factor associated with a higher prevalence of BD in men
**Jadnanansing et al., 2021**<br><br>**Suriname**	**Cross-sectional** [(populations in both region (rural/Nickerie & urban/Paramaribo)]	**2863 participants** (43% men) 39.97 years (?)	**AUDIT & ASSIST:** (for AUD)	**Risky alcohol use:** A score of > 7 on AUDIT	**Simple & Multivariable logistic regression**<br>AUD is 6.4% in urban area & 5.8% in rural area. Men had highest addiction risk at about 16% compared with 2% for females.<br>A treatment gap of 50% was found for AUDs in the rural area (64% urban area).<br>Married persons are significantly less likely to become alcoholic than singles and other groups in urban area.<br>In both areas, higher education was associated with a lower probability of alcohol abuse and dependence, while handymen showed a higher odd.
**Jirapramukpitak et al., 2008**<br><br>**Thailand** (Bangkok)	**Cross-sectional** (Suburban community of Bangkok in 2003 and 2004)	**1052 residents** (46.3%) ? (16–25 years	**AUDIT** (hazardou or harmful drinking) & **DIS** (illicit drug use-Diagnostic Interview Schedule)	**Illicit drug use:** assessed with self-report adapted from (DIS) and **Hazardous/harmful drinking:** with AUDIT **Migration**: the occasion when a young person born in amore rural area moves for the first time into Greater Bangkok.	**Multivariate analysis (logistic regression)**<br>10.9% (82 males and 17 females) had illicit drug use and 24.3% (179 males and 62 females) hazardous and harmful drinking. Hazardous/harmful drinking was associated independently with being late migrants, who moved at the age of 15 or older.
**Moreira et al., 1998**<br><br>**Brazil**	**Cross-sectional** (Adults in Porto Alegre, a city in southern Brazil)	**1099 individuals** (45% men) ? (18-88 years old)	**QFQs** (type, quantity, & frequen**cy) & CAGE questionnaire**	**Heavy drinking**: average consumption of 30g/day or more, a level of exposure associated with health risks **Dependence:** Two positive answers to the CAGE questionnaire	**Simple/multiple linear & logstic regression**<br>24.1% had never drunk alcohol (9.0%/men & 36.5%/women). 29.3% of men & 4.2% of women were heavy drinkers. 16% & 4.0% were CAGE+, respectively.<br>Consumption of 30 g/day ethanol was associated with increases of 1.5 & 2.3 mmHg in DBP & SBP for men and 2.1 and 3.2 mmHg for women respectively.<br>Prevalence of HTN was higher among those ingesting ≥ 30 g/day (odds ratio = 2.9).
**Oancea et al., 2021**<br><br>**Brazil**	**Cross-sectional** (2013 Brazilian National Health Survey)	**59,399 individuals** (47.6% men) weighted median age, 40.53 (18-60+	**NIAAA definitions** (Binge drinking/BD &	**BD (NIAAA)**: a pattern of drinking that brings BAC levels to at least 0.08 g/dl. (4 drinks for women & 5	**Weighted & adjusted logisitic reggression**<br>14.8% were current smokers, 13.8% were binge drinkers & 3.2% were heavy drinkers.<br>Self-reported current depression/SRCD,7.6%

For peer review only - http://bmjopen.bmj.com/site/about/guidelines.xhtml

For peer review only

		years)	Heavy drinking/HD)	for men in about 2hrs)<br>**HD**: ≥ 5 days of BD episodes in a month is defined as the HD index.	There was significant weighted & adjusted increase in the odds of SRCD among young adults (18–39 years) who were binge drinkers compared to those who were not binge drinkers (AOR = 1.32).
**Pengpid et al., 2021**<br><br>**South Africa**	**Cross-sectional** (National survey of all household members, who resided in that household the previous night) (Rural informal/ 26%, Rural farms/ 5%, Urban/69%)	**39,210 persons** (48.3% men) Median age,34 (IQR,25-48) (15 years & older)	**AUDIT** (Hazardous, harmful, or dependent alcohol use (HHDA):<br>**ASSIST** (Drug use in the past 3 months)<br>**K10** (Kessler Psychological Distress Scale)	**HHDA:**<br>Adults (≥ 20 yrs): cut-off score is ≥ 8 on AUDIT & Adolescents (15–19 years): 5 or more on AUDIT<br>**Drug use in past 3 months**: Any drug used in past 3 months was coded as 1 and never as 0'.<br>**Psychological distress:** scores ≥ 20 on (K10)	**Unadjusted & adjusted logisric regression**<br>10.3% engaged in HHDA, 16.5% (males) & 4.6% (females). Past 3-month drug use was 8.6%, 13.3% (males) & 4.1% (females). Men of middle age (25-34) with higher education, urban residence, drug use and psychological distress were positively associated with HHDA. Women of middle age (25-34) and mixed race, residing on rural farms and urban areas, drug use and psychological distress were positively associated & older age (≥55) & Indians or Asians were negatively associated with HHDA.
**Prais et al., 2008**<br><br>**Brazil**<br>(Metropolitan area of Belo Horizonte, & Bambuí)	**Cross-sectional** (elderly Brazilian men, ≥ 60 years were the study population)<br>**Population based** (urbann setting)	**685 residents in RMBH & 642 in Bambuí** (100% men)<br>**Mean age:**<br>68.8 yrs (RMBH)<br>69.0 yrs (Bambuí)<br>(≥ 60 years)	**RSOD criteria** (for BD)	**Binge Drinking:**<br>Consumption of five or more alcoholic drinks on a single occasion in the last 30 days.	**Multivariate analyses (PR estimated by Robust Poisson Regression)**<br>Prevalence of BD was two times higher among residents in metropolitan area of Belo Horizonte (27.1%) than in Bambuí (13.7%).<br>**RMBH:** higher schooling level [8+ yrs] (PR = 1.55), worse self-rated health [reasonable, bad, or very bad] (PR = 0.62) and inability to perform activities of daily living (PR = 0.12) remained significantly associated with BD.<br>**Bambuí**: worse self-rated health (PR = 0.57) and being divorced or separated (PR = 2.49) remained significantly associated with BD.
**Trangenstein et al., 2018**<br><br>**South Africa**<br>(Tshwane Metropole)	**Cross-sectional** (Adults who used alcohol in past six months).<br>(Data from South African arm of the multi-country International Alcohol Control, IAC study)	**713 adults** (65.8% men)<br>36.3 (18-65 years)	**International Alcohol Control (IAC) questionnair:** (Asks QFQs over past six months)	**Heavy Drinking (HD):** consuming ≥ 96g of absolute alcohol (AA) (roughly 8 standard drinks, or 120 ml) for men or ≥ 72g (6 standard drinks, or 90 ml) for women at least monthly.<br>**Low risk**: occasions that did not include HD	**Multivariate logistic regression**<br>HD was 53%. HD did not vary by gender (F1, 19 = 3.96, p = 0.06), age, race/ethnicity, or total annual personal income. Bivariate analyses revealed that HD differed by marital status (F2.48, 47.11 =3.09, p = 0.04).<br>Adjusting for marital status & primary container size, single persons were found to have substantially higher odds of HD.

For peer review only - http://bmjopen.bmj.com/site/about/guidelines.xhtml

	(urban setting)				
**Vellios and Van Walbeek, 2018**<br><br>**South Africa**	**Cross-sectional** (data from wave 4 of the 2014-2015 National Income Dynamics Study, NIDS) (rural/35.4%, urban/64.6%)	**22,752 (wave 4)** (46.8% men) ? (≥ 15 years)	**QFQs:**1) How often do you drink alcohol? 2) On a day you have an alcoholic drink, how many standard drinks do you usually have?	**Binge drinker:** use of ≥5 standard drinks on an average drinking day.<br>**Current drinker**: any option from (iii) I drink alcohol very rarely, (iv) Less than once a week, (v) On 1 or 2 days a week, (vi) On 3 or 4 days a week, (vii) On 5 or 6 days a week, & (viii) Every day.	**Multiple logit regressions**<br>Current alcohol use (any amount) in 2014 - 2015 was reported by 33.1% of the population (47.7% males, 20.2% females). Of current drinkers, 43.0% reported BD (48.2% males, 32.4% females).<br>Self-reported BD as a proportion of the total population was 14.1% (22.8% M, 6.4% F).<br>Self-reported BD was highest among males & females aged 25-34 years (49.4%).<br>Smoking cigarettes for both genders substantially increased the likelihood of drinking any amount (aOR: 5.08 males, 4.80 females) and of BD (aOR: 1.53 for males, 3.36 for females).<br>As a percentage of total population, people aged 25-34 years were more likely to binge than aged 15-24 years, for both males (OR 1.44) and females (OR 1.49). Compared with married males, males living with a partner (OR 1.58) or who were single (OR 1.74) were more likely to BD.<br>Compared with married females, females living with a partner (OR 1.68) or single (OR 1.41) were more likely to BD.<br>Having children in the house slightly increased the probability of BD for males (OR 1.21), but not for females.
**Aremu et al., 2021**<br><br>**Nigeria** (urban poor people in Ibadan)	**Cross-sectional** (two selected urban poor communities in Ibadan, Nigeria)	**500 Participants** (29.4% men) 35.36 (18-65 years)	**Modified version of WHO STEPS instrument**	**Alcohol consumers:** Ever consumed, Current consumers (12mo.) Current & **frequent consumers within 30 days (low, medium, and high)**<br>**Low consumers:** consuming < 4 (men) & < 2 (women) SDs/occasion<br>**Medium:** 4-6 (men) & 2-4 (women) SDs per occasion<br>**High:** > 6 (men) & > 4 (women) SDs per occasion	**Descriptive & inferential statistics (X^2)**<br>29.0% had consumed alcohol either in past or present, 17.8% consumed alcohol within last one year, 15.8% were current consumer of alcohol & 13.6% were frequent consumers who had taken alcohol within 30 days (11.6% low consumers, 1.2% medium consumers and 0.8% high consumers).<br>More male (53.1%) reported to have ever consumed alcohol compared to female (46.9%). 62.3% of non-current alcohol users was female & 37.7% were male. 59.3% of respondents not currently consuming alcohol were currently married (30.3% were not).<br>74.1% of the low consumers were male, 66.7% medium consumers were females, & 75.0% of high alcohol consumers were male
**Bonnechère et al., 2022**	**Cross- sectional** (Data from the	**4692 individuals** (45.7% men)	Quantity/Frequency	**4 levels of consumption:** No consumption (None)	**Multinomial logistic regression:**<br>3559 (75.8%) were not consuming any alcohol, 12.9% had low,

For peer review only - http://bmjopen.bmj.com/site/about/guidelines.xhtml

**Burkina Faso**	2013 Burkina Faso WHO STEPwise) Rural (75.1%), Urban (24.9%) **Population- based**	? (25–64 years)	Questions **(QFQs)**	**Low**: intake of pure alcohol of <40g/day (men) & <20g for women **Mid**: 40-59.9g/day (men) & 20-39.9g for women **Abusive consumption:** ≥60g/day (M) & ≥40g (W) **Dependent variable:** mean alcohol consumption in the last 30 days.	8.5% had mid and 2.7% had abusive alcohol consumption. Age was associated with any level of alcohol consumption with a gradient effect and older people having a higher level of consumption in comparison with no consumption. Tobacco consumption was significantly associated with alcohol intake with gradient effect, those with higher tobacco use being at higher risk of abusive alcohol intake. Sex is an important risk factor for abusive consumption with increased risk for men compared with women. Jobless people & housemaker was associated with a decreased risk of having abusive consumption.
**Dahal et al., 2021** **Nepal** (Kathmandu district)	**Cross-sectional** (adults residing in municipalities of Kathmandu district for at least six months) **Community based** (unplanned urbanization)	**245 participants** (47.3% men) **Mean age:** 41.19/male, & 40.91/female (18–69 years)	**WHO STEPS questionnaire (QFQs)**	**Current episodic heavy drinking (HED):** six or more drinks on any day in the past 30 days.	**Bivariate & multivariate analysis** 67.3% were lifetime abstainers. Prevalence of alcohol consumption in last 12 months was 31.0% & HED was 12.7%. Prevalence of current smoking, low intake of fruits & vegetables and low physical activity was found to be 22%, 93.9% and 10.2% respectively. 52.2% of participants were overweight/obese & prevalence of raised BP was 27.8%. Odds of alcohol consumption were higher among male (AOR: 2.78), employed (AOR: 2.30), & those who belonged to Chhetri (AOR: 2.83), Janajati (AOR: 6.18), Dalit and Madhesi, (AOR: 7.51) ethnic groups.
**Jonas et al., 2014** **India** (rural Central India)	**Cross-sectional** (data from Central India Eye and Medical Study, CIEMS, in rural region of Central Maharashtra) **Population-based**	**4711(participated)** (46.5% men) 49.5 (30+ years)	AUDIT CESD 20-item FTND (smoking behavior)	**Harmful or hazardous drinking:** sum score of 8 or more on AUDIT **Clinical episode of major depression:** score of > 21 in the CES-D.	**Test of for association not performed** Alcohol consumption was 23.0%; 6.0% subjects had an AUDIT score ≥8 (hazardous drinking), & 4.63% subjects a score ≥ 13 (women) or ≥ 15 (men) (alcohol dependence)
**Olickal et al., 2021** **India** (Puducherry, South	**Cross-sectional** (adult men aged above 18 years in Puducherry, South	**316 adult men** (100% men) 45.2 (≥18 years)	**WHO AUDIT WHO QoL-BREF questionnaire**	**Hazardous alcohol**: AUDIT score of 8–15 **Harmful alcohol use**: AUDIT score of 16–19	**Independent t-test, One-way ANOVA & Kruskal Wallis test, Multiple linear regression** Mean (SD) AUDIT score was 13.2 (6.7). Probable dependence was 8.2%, & hazardous or harmful use was

For peer review only - http://bmjopen.bmj.com/site/about/guidelines.xhtml

For peer review only

India)	India)<br>**Community-based** (rural/50%, urban/50%)			**Probable alcohol dependence**: score of 20 or more on AUDIT<br>**High risk:** A score eight and above on AUDIT<br>**QoL:** A higher score is indicative of a better QoL in each of the domains.	27.8%. Overall mean score of QoL was lower among alcohol users compared to non-alcohol users (50.7 vs 63.5)<br>QoL score was significantly lower among alcohol users (also in all domains).<br>High-risk alcohol users and urban residence had 11.2 & 4.1 less QoL scores respectively and educated had 7 more QoL scores compared to the reference category.
**Olickal et al., 2022**<br><br>**India**<br>(Puducherry, South India)	**Cross-sectional & Qualitative design** (Mixed design)<br><br>(All men ≥ 18 years from urban & rural field practice areas of a tertiary care centre in Puducherry, South India)	**316 subjects**<br>(100% men)<br>45.2 (19-60+ years)	**WHO AUDIT**<br>Discussion guide for FGD	**Probable alcohol dependence:** A total score of ≥20 on AUDIT	**A log binomial regression (prevalence ratio) & Manual content analysis**<br>Alcohol use was 38%, 40% were daily users)<br>(34% in rural to 42% in urban areas)<br>Among alcohol users, 21.7% were probable dependents on alcohol.<br>Older individuals had a 2.9 times higher risk of alcohol use than young individuals (<30).<br>No formal education was a high-risk factor for alcohol use, compared to educated.<br>Individuals residing in rural areas (APR = 1.05), self-reported comorbidities (APR = 1.21), family history of alcohol use (APR = 2.42) and tobacco use (APR = 2.42) were significantly associated with alcohol use.
**Sarma et al., 2019**<br><br>**India**<br>(Kerala, South India)	**Cross-sectional**<br>[(all individuals between 18-69 years old were eligible, in both rural & urban (49.3%) areas)] community-based	**12,012 adults**<br>(37% men)<br>42.5 (18–69 years)	**WHO STEPS instrument**<br>**GPAQ** (Global Physical Activity Questionnaire)<br>**Anthropometric measurements**	**Current alcohol use:** intake of at least one standard drink of alcohol in the past 30 days.<br>**Current tobacco use**: use of any form of tobacco within the past 30 days.<br>**Raised Blood Pressure (BP):**<br>BP of ≥140/≥90 mm Hg, or if the person is currently using antihypertensive medication.	**Weighted means, Percentages with 95% CI, & variance inflation applied**<br>Current use of tobacco & alcohol in men was 20.3% & 28.9% respectively.<br>The overall prevalence of raised BP was 30.4%.
**Endashaw Hareru et al., 2022**	**Cross-sectional**<br>(Residents of Dilla town, Gedeo zone,	**666 participants**<br>(70% men)<br>Mean: 33.3 years	**AIDIT:** AUD<br>**Kessler Psychological**	**AUD:** AUDIT score of ≥ 8	**Bivariate & multivariate binary logistic regression analysis**<br>AUD during the past year was 30.6%.<br>Being male (AOR = 8.33), age of less than 33 years old (AOR =

For peer review only

For peer review only - http://bmjopen.bmj.com/site/about/guidelines.xhtml

**Ethiopia** (Dilla town)	Southern Ethiopia with age of ≥ 18 years) **Community-based**	(≥ 18 years)	**Distress Scale (K10): ASSIST 2.0:** current and lifetime substance use		1.78), current cigarette smoking (AOR = 2.49), current khat chewing (AOR = 6.23), high level of psychological distress (AOR = 7.69) and poor social support (AOR = 2.30) were significantly associated with AUD.
**Gutema et al., 2020** **Ethiopia** (Arba Minch HDSS)	**Cross-sectional** (Adult residents of Arba Minch HDSS (nine Kebeles of Arba Minch Zuria District, Southern Ethiopia) **Community-based** (rural residents, 83.7%)	**3346 participants** (50% men) 44.6 years (25– 64 years)	**WHO STEPS instruments** (alcohol use) **SRQ-20** (mental stress status)	**HED or Excessive Alcohol Consumption:** use of ≥ 6 drinks for men and ≥ 4 drinks for women on a single occasion at least once per month. **Mental stress** (mild, moderate, and severe)	**Binary logistic regression** Prevalence (HED) was 13.7%. HED was associated with occupation (daily laborer: AOR 0.49; & housewives: AOR0.63 compared with farmers), wealth index (2nd quintiles: AOR 0.55 & 3rd quintiles: AOR 0.66) compared with 1st quintiles; & climatic zone (midland: AOR 1.80; highland: AOR 1.95 compared with lowland). Tobacco use (AOR 4.28), & khat use (AOR 4.75) were also associated with HED.
**Legas et al., 2021** **Ethiopia** (South Gondar)	**Cross-sectional** (adult residents whose age was 18 years and above in the South Gondar zone, 61.3% from urban areas)	**848 (interviewed)** (62.3% men) ? (≥ 18 years)	**AUDIT**-AUD **PHQ-9** **PSS**-Perceived stress scale questionnaire) **Oslo social support scale** **SPIN**-Social phobia inventory scale	**AUD**: score of 8 or above on AUDIT **Depression:** A score of five or more on the PHQ-9	**Bivariate & multivariable logistic analysis** AUD over the last 12-months was 23.7%. 16.50% had hazardous alcohol use, 5.2% had harmful alcohol use, and 2% had probable alcohol dependence. Being male (AOR = 4.34), poor social support (AOR = 1.95), social phobia (AOR = 1.69), perceived high level of stress (AOR = 2.85), current cigarette smoking (AOR = 3.06) and comorbid depression (AOR = 1.81) were significantly associated with AUD.
**Wainberg et al., 2018** **Mozambique** (Zambézia Province)	**Cross-sectional** (2014 survey) (16 year or older female heads-of-household in Mozambique, Zambézia Province) **Population-based** /rural Mozambique	**2,752 participants** (no men, 100% female) Median: 27 years (16-62 years)	**AUDIT** (Alcohol use) **PHQ** (Depression)	**Hazardous, harmful & high-risk drinkers:** AUDIT scores > 4 (recommended cutoff for women) **Depression:** A score of ≥ 10 on PHQ-8 (associated with clinical depression)	**Binomial logistic regression model:** Overall prevalence of current alcohol consumption among female heads of hh was 15%. "hazardous drinkers" was 8%. A positive depression screening (aOR: 2.20), death of a child (aOR: 2.44), & currently being pregnant (1.83) were associated with increased odds of hazardous drinking. Being single (aOR: 0.48) & experiencing food insecurity (aOR:0.96) were associated with reduced odds of risky drinking.
**Abrevations: AA**: Alcohol Abuse; **AD**: Alcohol Dependence; **aPR**: adjusted Prevalence Ratio; **AUD**: Alcohol Use Disorder; **AUDIT**: Alcohol Use Disorder Identification Test; **BD:**					

For peer review only - http://bmjopen.bmj.com/site/about/guidelines.xhtml

Binge drinking; **CAGE**: Cut down, Annoyed, Guilty feeling & Eye opener; **CESD:** Center for Epidemiologic Studies Depression Scale; **FAST**: Fast Alcohol Screening Test; **FTND:** Fagerstrom Test for Nicotine Dependence; **HD**: Heavy drinking; **HED**: Heavy Episodic Drinking; **wk**: week; **M:** men; **MDE:** Major Depressive Episode; **NIAAA**: National Institute on Alcohol Abuse and Alcoholism; **PHQ-9:** Patient Health Questionnaire-9 item; **PR:** Prevalence Ratio; **PRIME**: Programme for Improving Mental Healthcare; **QFQs**: quantity/frequency questionnaires; **QoL:** Quality of Life; **RMBH:** metropolitan region of Belo Horizonte; **RR:** response rate**; SD:** Standard drink; **W:** women; **yr.**: year; **?:** mean age or age range for subjects is not determined.

For peer review only

Supplementary File 4

Newcastle-Ottawa Scale (NOS) adapted for quality assessment of cross-sectional studies for the study "a scoping review of assessment tools for, magnitudes of, and factors associated with problem alcohol use: community-based studies," 2023.

Selection: (Maximum 5 points/scores/stars)
**1. Representativeness of the sample:**
a. Truly representative of the average in the target population. * (all subjects/consecutive or random sampling)
b. Somewhat representative of the average in the target group. * (non-random sampling)
c. Selected group of users/convenience sample.
d. No description of the derivation of the included subjects (sampling strategy).
**2. Sample size:**
a. Justified and satisfactory (including sample size calculation). * (1 score)
b. Not justified
c. No information provided
**3. Non-respondents:**
a. Proportion of target sample recruited attains pre-specified target or basic summary of non-respondent characteristics in sampling frame recorded. *
b. Unsatisfactory recruitment rate, no summary data on non-respondents.
c. No information provided
**4. Ascertainment of the exposure (risk factor/disease) or screening/surveillance (measurement) tool:**
a. Secure record (medical charts) or validated measurement (screening/surveillance) tool. **
b. Non-validated measurement tool, but the tool is available or described or Self-report. *
c. No description of the measurement tool.
**Comparability: (Maximum 2 stars)**
**1. Comparability of subjects in different outcome groups on the basis of design or analysis. Confounding factors controlled.**
a. Data/results adjusted for relevant predictors/risk factors/confounders e.g., age, sex, marital status, job etc. **

For peer review only

b. Data/results not adjusted for all relevant confounders/risk factors/information not provided.
**Outcome: (Maximum 3 stars)**
**1. Assessment of outcome:**
a. Independent blind (structured) assessment. **
b. Record linkage. **
c. Self report. *
d. No description.
**2. Statistical test:**
a.Statistical test used to analyse the data clearly described, appropriate, and measures of the association presented including confidence intervals and probability level (p-value). *
b.Statistical test not appropriate, not described, or incomplete.

Scoring for cross-sectional Studies:

Very Good Studies: 9-10 points

Good Studies: 7-8 points

Satisfactory Studies: 5-6 points

Unsatisfactory Studies: 0 to 4 points

For peer review only

References

1. Ribeiro CM, Beserra BTS, Silva NG, Lima CL, Rocha PRS, Coelho MS, et al. Exposure to endocrine-disrupting chemicals and anthropometric measures of obesity: A systematic review and meta-analysis. BMJ open. 2020;10(6):e033509.
2. Zhao C, Xing F, Yeo YH, Jin M, Le R, Le M, et al. Only one-third of hepatocellular carcinoma cases are diagnosed via screening or surveillance: a systematic review and meta-analysis. European journal of gastroenterology & hepatology. 2020;32(3):406-19.
3. Patra J, Bhatia M, Suraweera W, Morris SK, Patra C, Gupta PC, et al. Exposure to second-hand smoke and the risk of tuberculosis in children and adults: a systematic review and meta-analysis of 18 observational studies. PLoS medicine. 2015;12(6):e1001835.
4. Modesti P, Reboldi G, Cappuccio F. Newcastle-Ottawa Quality Assessment Scale (adapted for cross sectional studies). PloS one. 2016;11(1):e0147601.
5. Dubey VP, Kievišienė J, Rauckiene-Michealsson A, Norkiene S, Razbadauskas A, Agostinis-Sobrinho C. Bullying and Health Related Quality of Life among Adolescents—A Systematic Review. Children. 2022;9(6):766.
6. Naafs JC, Vendrig LM, Limpens J, Van Der Lee H, Duijnhoven RG, Marchal J, et al. Cognitive outcome in congenital central hypothyroidism: a systematic review with meta-analysis of individual patient data. European journal of endocrinology. 2020;182(3):351-61.

For peer review only

Supplementary File 5

Newcastle-Ottawa Scale (NOS) quality assessment summary for the study "A scoping review of assessment tools for, magnitudes of, and factors associated with problem alcohol use: community-based studies," 2023.

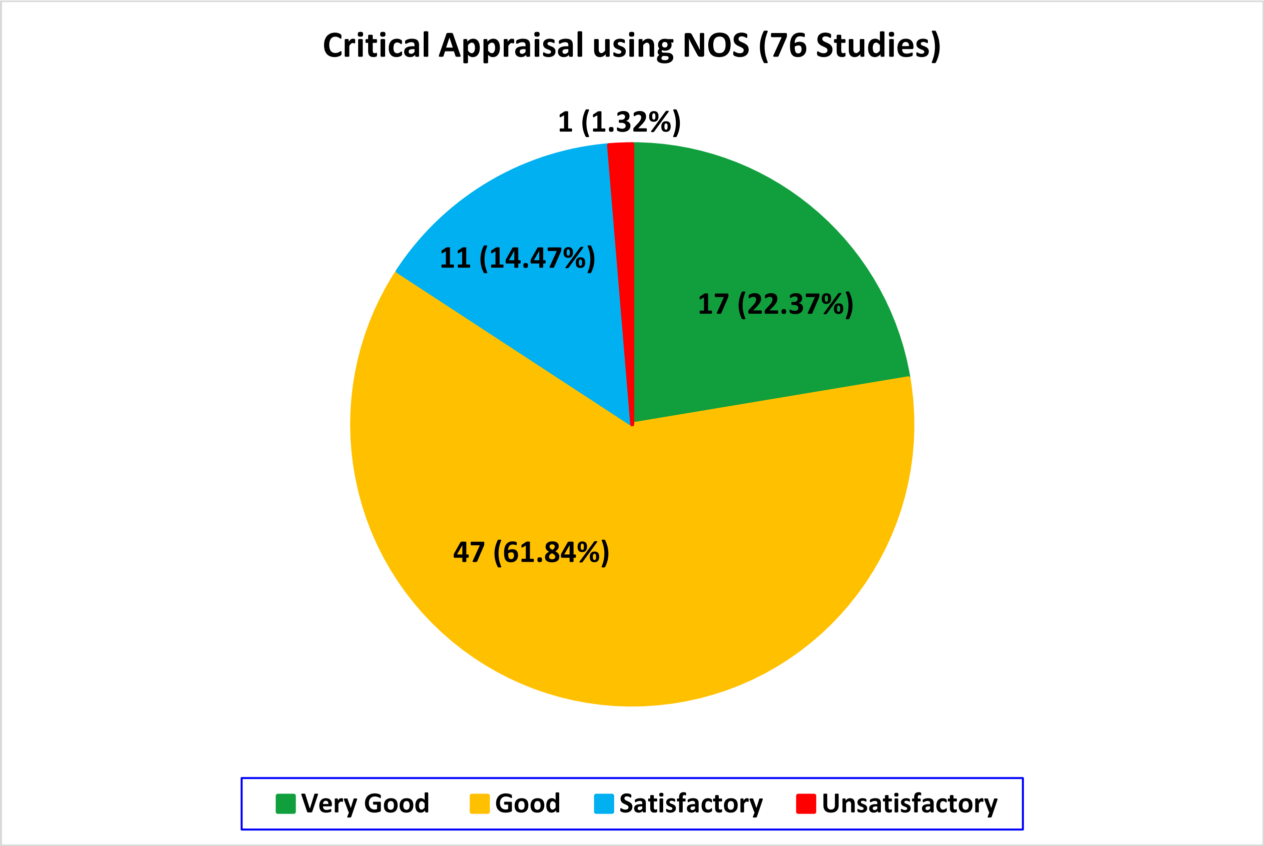

Figure: Newcastle-Ottawa Scale (NOS) quality assessment reports of studies for the study "A scoping review of assessment tools for, magnitudes of, and factors associated with problem alcohol use: community-based studies," 2023.

Table: Newcastle-Ottawa Scale (NOS) quality assessment- item level summary for "A scoping review of assessment tools for, magnitudes of, and factors associated with problem alcohol use: community-based studies," 2023.	Studies (76)
**Selection: (Maximum 5 points/scores/stars)**	
**1. Representativeness of the sample:**	
a. Truly representative of the average in the target population. * (all subjects/consecutive or random sampling)	**64**
b. Somewhat representative of the average in the target group. * (non-random sampling)	**12**
c. Selected group of users/convenience sample.	**0**
d. No description of the derivation of the included subjects (sampling strategy).	**0**
**2. Sample size:**	
a. Justified and satisfactory (including sample size calculation). * (1 score)	**40**
b. Not justified	**23**
c. No information provided	**13**
**3. Non-respondents:**	
a. Proportion of target sample recruited attains pre-specified target or basic summary of non-respondent characteristics in sampling frame recorded. *	**70**
b. Unsatisfactory recruitment rate, no summary data on non-respondents.	**01**
c. No information provided	**05**
**4. Ascertainment of the exposure (risk factor/disease) or screening/surveillance (measuremnt) tool:**	
a. Secure record (medical charts) or validated measurement (screening/surveillance) tool. **	**28**
b. Non-validated measurement tool, but the tool is available or described or Self report. *	**48**
c. No description of the measurement tool.	**0**
**Comparability: (Maximum 2 stars)**	

For peer review only

**1. Comparability of subjects in different outcome groups on the basis of design or analysis. Confounding factors controlled.**	
a. Data/results adjusted for relevant predictors/risk factors/confounders e.g., age, sex, marital status, job etc. **	**63**
b. Data/results not adjusted for all relevant confounders/risk factors/information not provided.	**13**
**Outcome: (Maximum 3 stars)**	
**1. Assessment of outcome:**	
a. Independent blind (structured) assessment. **	**14**
b. Record linkage. **	**0**
c. Self report. *	**62**
d. No description.	**0**
**2. Statistical test:**	
a.Statistical test used to analyse the data clearly described, appropriate and measures of association presented including confidence intervals and probability level (p value). *	**69**
b.Statistical test not appropriate, not described, or incomplete.	**07**

For peer review only

BMJ Open

A scoping review of assessment tools for, magnitudes of, and factors associated with problem drinking in population-based studies

Journal:	*BMJ Open*
Manuscript ID	bmjopen-2023-080657.R1
Article Type:	Original research
Date Submitted by the Author:	21-Dec-2023

Complete List of Authors:	Gizachew, Kefyalew Dagne; Addis Ababa University, Department of Psychiatry, School of Medicine, College of Health Sciences; Debre Berhan University, Department of Psychiatry, College of Health Sciences and Medicine<br>Myers, Bronwyn; Curtin University, Curtin enAble Institute, Faculty of Health Sciences; South African Medical Research Council, Alcohol, Tobacco and Other Drug research Institute<br>Awoke, Mihretu; Addis Ababa University, Department of Psychiatry, School of Medicine, College of Health Sciences<br>Teferra, Solomon; Addis Ababa University, Department of Psychiatry, School of Medicine, College of Health Sciences

**Primary Subject Heading**:	Mental health
Secondary Subject Heading:	Mental health, Public health, Addiction, Epidemiology
Keywords:	Systematic Review, Ethanol, AUDIT, EPIDEMIOLOGIC STUDIES, MENTAL HEALTH, Substance misuse < PSYCHIATRY

SCHOLARONE™
Manuscripts

I, the Submitting Author has the right to grant and does grant on behalf of all authors of the Work (as defined in the below author licence), an exclusive licence and/or a non-exclusive licence for contributions from authors who are: i) UK Crown employees; ii) where BMJ has agreed a CC-BY licence shall apply, and/or iii) in accordance with the terms applicable for US Federal Government officers or employees acting as part of their official duties; on a worldwide, perpetual, irrevocable, royalty-free basis to BMJ Publishing Group Ltd ("BMJ") its licensees and where the relevant Journal is co-owned by BMJ to the co-owners of the Journal, to publish the Work in this journal and any other BMJ products and to exploit all rights, as set out in our licence.

The Submitting Author accepts and understands that any supply made under these terms is made by BMJ to the Submitting Author unless you are acting as an employee on behalf of your employer or a postgraduate student of an affiliated institution which is paying any applicable article publishing charge ("APC") for Open Access articles. Where the Submitting Author wishes to make the Work available on an Open Access basis (and intends to pay the relevant APC), the terms of reuse of such Open Access shall be governed by a Creative Commons licence – details of these licences and which Creative Commons licence will apply to this Work are set out in our licence referred to above.

Other than as permitted in any relevant BMJ Author's Self Archiving Policies, I confirm this Work has not been accepted for publication elsewhere, is not being considered for publication elsewhere and does not duplicate material already published. I confirm all authors consent to publication of this Work and authorise the granting of this licence.

For peer review only

A scoping review of assessment tools for, magnitudes of, and factors associated with problem drinking in population-based studies

Kefyalew Dagne Gizachew [1, 2], Bronwyn Myers [3,4,5], Awoke Mihretu [1] & Solomon Teferra [1]

[1] Department of Psychiatry, School of Medicine, College of Health Sciences, Addis Ababa University, Addis Ababa, Ethiopia

[2] Department of Psychiatry, College of Health Sciences and Medicine, Debre Berhan University, Debre Berhan, Ethiopia

[3] Curtin enAble Institute, Faculty of Health Sciences, Curtin University, Kent Street, Bentley, WA 6102, Australia

[4] Alcohol, Tobacco and Other Drug Research Unit, South African Medical Research Council, Cape Town, South Africa.

[5] Division of Addiction Psychiatry, Department of Psychiatry and Mental Health, University of Cape Town, Cape Town, South Africa

***Corresponding Author:** Kefyalew Dagne Gizachew

Tel: +251910487276; P.O. Box: 9086; E-mail: kdgc08@yahoo.com

ABSTRACT

Background: The term "problem drinking" includes a spectrum of alcohol problems ranging from excessive/heavy drinking to alcohol use disorder. Problem drinking is a leading risk factor for death and disability globally. It has been measured and conceptualized in different ways- making it difficult to identify common risk factors for problem alcohol use. This scoping review aims to synthesize what is known about the assessment of problem drinking, its magnitude, and associated factors.

Methods: Four databases (PubMed, EMBASE, PsycINFO, Global Index Medicus/GIM) and Google Scholar were searched from inception to November 25, 2023. Eligibility criteria were limited to people aged 15 and above, population-based studies reporting problem alcohol use, and English-language articles. This review was reported based on guidelines from the "Preferred Reporting Items for Systematic Reviews and Meta-

For peer review only

Analyses extension for Scoping Reviews (PRISMA-ScR) Checklist." Critical appraisal was done using the Newcastle-Ottawa Scale (NOS).

Results: From the 14,296 records identified, 10,749 underwent title/abstract screening, of which 352 full-text articles were assessed, and 81 articles were included for data extraction. Assessment tools included self-report quantity/frequency questionnaires, criteria to determine risky single occasion drinking, validated screening tools, or structured clinical and diagnostic interviews. The most widely used screening tool was the Alcohol Use Disorder Identification Test. Studies defined problem drinking in various ways, including excessive/heavy drinking, binge drinking, alcohol use disorder, alcohol abuse, and dependence. Across studies, the prevalence of heavy drinking ranged from < 1.0% to 53%, binge drinking from 2.7% to 48.2%, alcohol abuse from 4% to 19.0%, alcohol dependence from 0.06% to 39%, and alcohol use disorder from 2% to 66.6%. Factors associated with problem drinking varied across studies. These included socio-demographic and economic factors like age, sex, relationship status, education, employment, income level, religion, race, location, and alcohol outlet density. The other factors consisted of clinical factors like medical problems, mental disorders, substance use, and quality of life.

Conclusions: Due to differences in measurement, study designs, and assessed risk factors, there was a wide variability in the prevalence of problem drinking and associated factors across studies and settings. The alcohol field would benefit from measuring alcohol use in a harmonised way to allow for comparisons to be made across countries and for meta-analyses.

Scoping Review Registration: Open Science Framework (https://osf.io/9syv7, or https://doi.org/10.17605/OSF.IO/9SYV7)

Keywords: Scoping review, Alcohol, Alcohol use disorder, Problem drinking, Heavy drinking, Binge drinking, Heavy episodic drinking, Alcohol use assessment

For peer review only

ARTICLE SUMMARY

Strengths and limitations of this study

- ► To the authors' knowledge, no other scoping review covers global settings to map and aggregate findings and offer an overview of problem drinking.
- ► Strengths also comprised an extensive search of four databases, including 81 original articles for synthesis.
- ► We included only community-based studies; studies conducted at institutions like hospitals, primary health care centers (PHC), addiction centers, and colleges or universities were not included.

For peer review only

INTRODUCTION

The nature of alcohol use, related issues, and how they manifest throughout life have long been the subject of scientific research (1). In 2016, the "Global Burden of Disease Study" identified alcohol use as a leading risk factor for death and disability, and it was ranked seventh among the top risk factors for disability-adjusted life years (DALYs) and deaths globally (2, 3). Previous studies have implicated alcohol in more than 200 injuries and diseases, including alcohol use disorder, liver cirrhosis, malignancies, injuries, tuberculosis, HIV/AIDS (4, 5), noncommunicable diseases (NCDs) (6), mental disorders (7), violence-related harms and injuries (8). These problems can result from acute episodes of alcohol intoxication or chronic heavy alcohol use (9). The phrase "alcohol use disorder" (AUD) describes the complete range of alcohol abuse (AA), including heavy episodic/binge drinking (HED/BD), risky drinking, harmful drinking behaviors, and alcohol dependence (AD) (10). AUD varies and can range from less severe problems such as heavy, hazardous, or harmful drinking to more serious disorders like alcohol abuse or alcohol dependence. Many challenges in understanding the nature and extent of alcohol-related problems, including all spectrums of AUD, arise from different definitions of problematic alcohol use and inconsistent ways of measuring it. In this review, we use the term "problem drinking" to refer to any problem with alcohol use, including AUD. Different definitions and terms for problem alcohol use (11-26) are summarised in (Table 1).

Table 1: Different definitions and terms for problem alcohol use in the study, 2023.

Terms	Definitions
**Problem Drinking (PD)**	Problem drinking, commonly referred to as "alcohol abuse," "alcohol misuse," or "AUD," is a pattern of alcohol intake that harms one's health or relationships with others. It is a general term that covers a range of alcohol-related problems, from mild to severe. Although problem drinking does not necessarily fulfill the diagnostic criteria for AUD, it can negatively impact a person's life (11-16).
**Hazardous drinking**	A quantity or pattern of alcohol intake puts individuals at risk for adverse health events, which carry the possibility of physical or

For peer review only

	psychological harm (17, 18).
**Harmful drinking**	Alcohol intake, which causes physical or psychological harm or the presence of physical or psychological complications, defines it (17, 19).
**Low-risk drinking**	A daily intake of no more than 20g of alcohol with at least two non-drinking days weekly is different for males and females, i.e., not more than three and two drinks a day on average, respectively (20).
**Heavy episodic/binge drinking (HED/BD)**	It is defined as the intake of five or more drinks for men and four or more drinks for women per occasion in most studies (roughly 60 grams of pure alcohol), which brings blood alcohol concentration (BAC) levels to 0.08 gram/dL in about two hours (21).
**Exessive/heavy drinking (HD)**	Heavy drinking is the quantity of alcohol consumed that exceeds a set threshold. It is often defined as the weekly use of more than 14 drinks on average for males and more than seven drinks for females. Some countries define it as the average number of binge episodes per person during 30 days or weekly drinking of more than 21 drinks for males and more than 14 drinks for females (21-24).
**Alcohol dependence (AD)**	Based on the Diagnostic and Statistical Manual of Mental Disorders- 4th edition (DSM-IV), alcohol dependence is characterized by a problematic pattern of alcohol use that result in clinically significant impairment or distress. It is also a symptom of continuing to use alcohol despite knowing that continued use will cause serious social or interpersonal problems (for example, violent arguments with their spouse while intoxicated or abusing children) (25).
**Alcohol abuse (AA)**	Alcohol abuse is a pattern of alcohol intake that has adverse outcomes and harms a person's physical health, mental health, interpersonal connections, and general functioning. Alcohol

For peer review only

	abuse involves excessive and frequent alcohol consumption despite its harmful effects. It can be less severe than alcohol dependence because it requires fewer symptoms and can only be diagnosed once the DSM-IV criteria have determined that alcohol dependence is not present (25).
**Alcohol use disorder (AUD)**	AUD is a chronic medical disorder defined by an individual's compulsive and problematic pattern of alcohol consumption, diagnosed when an individual's alcohol consumption leads to significant distress or impairment in their daily functioning. It is characterized by a cluster of behavioral and physical symptoms, including withdrawal, tolerance, and craving, based on the Diagnostic and Statistical Manual of Mental Disorders-5th edition (DSM-5) (11, 26).

Alcohol consumption is responsible for a wide range of adverse health outcomes (3), and alcohol-related harms are well established (27). Problem drinking, including any form of AUD, is a critical public health issue that has an impact on people and communities all around the world (28).

The explicit factors responsible for the emergence and advancement of problem drinking are not completely understood (2). Despite the severe burden of alcohol use globally, there is fragmented evidence, a lack of understanding, and a notable gap in the breadth of specific contributing factors and the full picture of problem drinking (2).

Although alcohol consumption occurs on a continuum, our understanding of when to intervene and risk factors to target in interventions is hampered by limited universally accepted methods in how problem drinking is conceptualized and measured and the lack of synthesized evidence on factors associated with problem drinking.

A comprehensive global-wide review of problem drinking-related information serves several essential purposes. First, it offers crucial epidemiological data, such as burden or prevalence rates, trends, and problem drinking patterns over time. With this information, public policymakers, researchers, and healthcare workers may more accurately understand the scope of the problem, pinpoint individuals at high risk, and

For peer review only

more effectively allocate resources to problem drinking prevention and treatment. Second, the information from the review may be utilized to create awareness of problem drinking and develop policy initiatives on screening and treatment strategies to reduce its prevalence. Third, studying problem drinking data enables a clearer understanding of factors related to the development and progression of problem drinking. This information will guide prevention initiatives and treatments focusing on specific risk factors, such as the environment, clinical variables, and comorbid mental health problems.

Previous reviews recommended a need for further research on the magnitude of problem drinking, focusing on LMICs (2). Existing studies target specific regions, contexts, and populations with complex and variable measures and definitions of problem drinking- which warrants a global-scale review, making it essential to explore and compile from a wide range of resources for a more comprehensive viewpoint. A review covering a broader range of measures, definitions, and associated factors adds a more integrated understanding of the phenomenon. It highlights commonalities and variations of problem drinking across diverse settings and populations.

Presenting associated factors alongside the prevalence rates of problem drinking in the results of this scoping review adds depth to our comprehension of the complex, multifaceted nature of alcohol use and the interplay between social, psychological, biological, and other essential determinants of drinking. Besides, synthesizing a broader literature and global perspective of problem drinking with its sophisticated and diverse range of associated factors is fundamental for effective and context-specific prevention, intervention, and alcohol-related harm reduction strategies (2).

This scoping review aims to overview the range of community-based screening or measurement tools for problem drinking and to synthesize the global nature and extent of problem drinking and associated factors among the general population. Due to the inclusion of all problem drinking prevalence studies globally and the broad coverage of settings, only population-based studies are included in this scoping review, and studies conducted at PHC, or hospital settings, universities, or schools are excluded. Extracted results of articles from HICs and LMICs are presented separately using tables, not for specific purposes, but for better visualization.

For peer review only

METHODS

This scoping review was reported based on guidelines from the "Preferred Reporting Items for Systematic Reviews and Meta-Analyses extension for Scoping Reviews (PRISMA-ScR) Checklist," a tool that is used to guide the scoping review process (29). A copy of PRISMA-ScR checklist for scoping reviews is supplemented as an additional file (Research Checklist 1).

Eligibility criteria

For this review, only articles written in the English language were considered. The PICO framework for prevalence studies (Population, Measurement of presence of disease, Design, and Setting) guided the choice of eligibility criteria. Accordingly, for studies to be included, they had to (a) study people aged 15 years or older (Population); (b) report problem drinking or AUD using any screening scales, measures, instruments, clinical diagnostic interviews or laboratory tests to detect alcohol use (Measurement of the presence of disease); (c) have any epidemiological, population-based design (Design); and (d) be located in any country or type of setting, as long as the study had a community-based sample (Setting).

Information sources

The literature search included four databases: PubMed, EMBASE, PsycINFO, and Global Index Medicus (GIM) and searched from database inception (spanning from 1996, 1974, 1906, and 1948 respectively) to August 26, 2019. Database searching was updated twice: first on July 22, 2022, and second on November 25, 2023. Additional records were identified through other sources such as Google Scholar.

To ensure methodological rigor, a scoping review protocol for the review was registered with Open Science Framework (OSF), which can be accessed using the associated project ID (registration number) of (https://osf.io/2anj3).

Search Criteria

The PI (KD) developed the search strategy with close consultations with supervisors (ST and BM). The search strategy consisted of key terms, free texts, and controlled vocabulary search terms such as (Medical Subject Heading/MeSH terms for MEDLINE and Emtree terms for Embase) for the main big terms of "prevalence," "alcohol," and "community/population-based health surveys." Terms within each set were grouped

For peer review only

using Boolean "OR" operators, and terms across sets were combined using "AND" operators. Terms related to alcohol use and the search strategy for searched databases are included in (Supplementary File 1).

Selection of sources of evidence

After the databases were searched, the titles and abstracts of identified records were imported into EndNote software for deduplication and to facilitate the review process. Two reviewers (KD and AM) independently completed screening article titles and abstracts in the first stage and screening full-text articles in the second stage using a priori inclusion and exclusion criteria to determine eligibility. These two reviewers met to resolve screening and selection differences with discussion and to reach a consensus on whether to include an article. These two independent reviewers assessed the eligibility of 352 full-text articles for the final inclusion of 81 articles in the scoping review. For the measure of agreement, percent agreement was applied, and 96.59% between the two reviewers.

Data charting process

We developed a data extraction form that included items relating to study characteristics (author, year of publication and citation, study country/location), study design, study setting and population, sample size, study tools or measures, and results. Two reviewers (KD and AM) independently extracted data from included studies using this form. These reviewers met to resolve data extraction differences with discussion and to reach a consensus on what to extract from the included articles.

Collating, summarising, and reporting the results

As a scoping review, the aim was to map and aggregate findings to offer and present an overview of the topic and all the material studied. Data were analyzed using descriptive statistics with Microsoft Excel, and the results were reported using narrative synthesis.

Although critical appraisal of the quality of included studies is not mandatory in scoping reviews, we decided to assess study quality so that findings from the current scoping review could inform the selection of alcohol screening tools and measures in future studies. We used the "Newcastle-Ottawa Quality Assessment Scale (NOS)" for cross-sectional studies (30-32). We slightly modified the semantics of some items to better align with this review (Supplementary File 2). The tool has three domains, each with

For peer review only

For peer review only - http://bmjopen.bmj.com/site/about/guidelines.xhtml

maximum stars (points/scores): i) selection (maximum five stars/*****), ii) comparability (maximum two stars/**), and iii) outcome (maximum three stars/***) giving a total score of 10. Studies that scored 9-10 points were considered very good, those that scored 7-8 points were rated as good, those that scored 5-6 points were rated as satisfactory, and those that scored 4 points or less were rated as unsatisfactory (31).

Patient and Public Involvement

There was no patient or public involvement in this scoping review.

RESULTS

The search yielded 14,296 articles from all databases and three additional records from Google Scholar. After deduplication, there were 10,749 records, and all these articles underwent title and abstract screening. After titles/abstracts screening, 352 articles were assessed for full-text eligibility, of which 81 articles were included for data extraction. The PRISMA flow diagram summarizes this article selection process (Figure 1).

Characteristics of included studies

The publication year for included articles ranged from 1996 to 2023. Only five studies were published before 2000, 19 from 2000-2010, and 57 from 2011-2023. Of the 81 full-text articles included in this scoping review, 29 were from High-Income countries (HICs; Table 2: Supplementary File 3), and the remaining 52 studies were from low- and Middle-Income countries (LMICs; Table 3: Supplementary File 4). Of these 52 studies, 38 were from Middle-Income countries (MICs), 25 were from Upper-Middle-Income countries, 13 were from Lower-Middle-income countries, and 14 were from Low-Income countries (LICs).

Most of the studies employed a cross-sectional study design (73/81), and the rest of the studies were longitudinal/cohort designs (6/81) and mixed quantitative and qualitative designs (2/81). Almost all included studies were population or community-based surveys. For the majority of included studies (n=30, 37.04%), the study population resided in an urban location, followed by a mixed urban/rural setting (n=27, 33.33% of studies) and rural (n=9, 11.11%). Fifteen (18.52%) studies did not specify the location of the population.

Among the included studies, the total sample size ranged from 99 to 358,355 participants. Only 11 studies had a sample size of less than 500 individuals. Almost

For peer review only

For peer review only - http://bmjopen.bmj.com/site/about/guidelines.xhtml

74.07 % (n=60) of the studies included had more than 1000 participants in their sample. Nine studies were conducted only among men, two only among women, and gender was not specified in four studies. Four studies were conducted among young adults (16-25 years old) and seven among older people (adults ≥ 50 years old). Across studies, participants ranged from 15 to 100 years old, and the mean or median age ranged from 20 to 81.

Critical appraisal of included studies

When assessing the overall methodological quality of included studies, 17 (21%) were rated as very good, 51 (62.96%) as good, 12 (14.81%) as satisfactory, and one (1.23%) as unsatisfactory (Supplementary File 5).

Measures of problem drinking

The included studies used a mix of measurement methods to assess problem drinking, grouped into self-report quantity/frequency (QF) questionnaires- including risky single occasion drinking (RSOD) criteria, screening tools, or structured clinically administered (gold-standard) instruments.

Quantity/frequency questionnaires and risky single occasion drinking criteria

Of 81 studies included, 19/29 in the HICs (Table 2: Supplementary File 3) and 21/52 in the LMICs (Table 3: Supplementary File 4) used QF questionnaires. The time interval in which the pattern of alcohol consumption (frequency and quantity) was defined and reported was expressed in days, weeks, months, past 12 months (current use), and ever (lifetime) use. Some studies used country-specific guidelines of recommended limits, which are part of the QF questionnaires like French alcohol consumption habits (33), Australian National Health and MRC 2009 guidelines for mean daily alcohol intake (34), Health Council of Netherlands recommended limit for alcohol (35), and UK National Statistics definition for BD or heavy drinking (36). Nine studies from HICs and four studies from LMICs applied risky single occasion drinking criteria. Among HICs, a survey in the US used NIAAA guidelines, SAMHSA definitions for BD (37, 38), and risky single occasion drinking criteria was also applied in Ireland (39) and Switzerland (40).

Screening and diagnostic interviews for problem drinking

Studies used a variety of screening tools to assess problem drinking. The most commonly used tools included the CAGE questionnaire (41-43), the AUDIT (17), the

For peer review only

MAST (44, 45), and the Alcohol, Smoking, and Substance Involvement Screening Test (ASSIST) (46).

Specifically, three studies from HICs (35, 47, 48) and four from LMICs (49-52) used the CAGE. Five studies from HICs, including New Zealand (53), the Netherlands (35), the UK (54), Norway (55), and Sweden (56), used either the full or abbreviated versions of AUDIT. Similarly, 24 studies from LMICs used AUDIT. The three-item AUDIT-C was used in South Africa, Cambodia, the UK, and Sweden (54, 56-58), and a four-item version of the AUDIT- the Fast Alcohol Screening Test (FAST) was used in Ethiopia (59). Only four studies in LMICs, Suriname (60), South Africa (61) and Ethiopia (62, 63) applied ASSIST.

The included studies in the review used five different AUD diagnostic interviews. First, several studies used the Composite International Diagnostic Interview (CIDI) (64-68). Country-specific versions of CIDI-structured diagnostic tools based on DSM-III, DSM-III-R, DSM-IV, DSM-5, or ICD-10 and ICD-11 (69, 70) were administered for the detection and diagnosis of problem drinking like AUD, alcohol abuse, or alcohol dependence in 11 studies from HICs including Hong Kong (71), Germany (72, 73), Israel (74), Australia (75), the Netherlands (76), Sweden (77), Ireland (39), USA (78), Finland (79), and Switzerland (40). It was also used in three studies from LMICs, including Sri Lanka (80), Ethiopia (51), and South Africa (81). Second, Alcohol Abuse and Alcoholism's Alcohol Use Disorder and Associated Disabilities Interview Schedule-DSM-IV version (AUDADIS-IV) (82) was used in one study in HICs- in the US (78). Third, the Structured Clinical Interview for DSM-IV (SCID-I) (83-85) was used. A study in Finland (79) applied SCID-I complemented by medical record data and expert interviews to detect lifetime DSM-IV substance use disorder (SUD).

Fourth, the Diagnostic and Statistical Manual of Mental Disorders- 4th and 5th editions (DSM-IV and DSM-5) (25, 26) was used. Only two HIC studies from Switzerland (40) and Sweden (77) applied DSM-IV or DSM-5 criteria to diagnose alcohol abuse, alcohol dependence, or AUD.

Fifth, studies used the Mini International Neuropsychiatric Interview (M.I.N.I.), versions 5, 6, & 7.0.2, structured based on DSM (86-88) to detect AUD. It is a DSM-IV-based diagnostic tool for alcohol use during the past 12 months (alcohol dependence and

For peer review only

abuse), and only one study from HICs- the USA used M.I.N.I. (89). It was employed for the detection of alcohol use in three studies from LMICs, namely South Africa (81), Malaysia (90), and Thailand (91).

Definitions of problem drinking

Studies have delineated problem drinking in a variety of ways, including (HED/BD), excessive/heavy drinking, or AUD. Definitions of heavy drinking and HED/BD differed according to the recommended drinking limits of countries and how individual studies operationalized the construct. For instance, a study in Finland (47) defined heavy drinking for males as ≥ 280g of absolute ethanol or 24 drinks per week and/or a CAGE score ≥ 3 and for women as ≥ 190g of absolute ethanol or 16 drinks per week and/or a CAGE score ≥ 2. Another study in the USA (37) defined heavy drinking for males as > 14 drinks per week and > four drinks per day and for females as > seven drinks per week and > three drinks per day. This weekly drinking definition of heavy drinking is also applied in China (92). A study in France (33) defined heavy drinking as ≥ 60g ethanol per day or six glasses per day of any alcoholic drink for males and ≥ 30g per day or about three glasses per day for females. Heavy drinking in two studies in the Netherlands (35, 76) and one study in Botswana (24) for women was > 14 standard glasses per week, and for men, it was > 21 drinks per week. Two studies in Brazil (49, 93) operationalized heavy drinking or hazardous drinking as an average of ≥ 30g per day, irrespective of gender. Studies from South Africa classified heavy drinking as > seven drinks per week (94).

HED was sometimes used interchangeably with BD. Studies in Hong Kong (71, 95) and the US (96) defined HED/BD as drinking ≥ five drinks in a row on a single occasion in the past month, irrespective of sex. Most studies described it differently for males and females. National Institute on Alcohol Abuse and Alcoholism (NIAAA) guidelines for risky drinking criteria, Substance Abuse and Mental Health Services Administration (SAMHSA) definition, or risky single occasion drinking criteria were mainly applied to define HED/BD (94, 97-99). In the US (78, 100), Singapore (101), Peru (97), South Africa (57), and Brazil (98, 99, 102), HED/BD was defined as ≥ five drinks per occasion for men and ≥ four drinks per occasion for women, a pattern of drinking that brings blood alcohol level (BAC) to at least 0.08 g/dl and reflects ≥ 60g pure alcohol. It was

For peer review only

also defined like this by studies conducted in India and Ireland (39, 103). In South Africa, one study (94) used a cut-off of > three drinks per occasion weekly, and another study (104) used ≥ five drinks on an average drinking day to define HED. Other studies defined HED/BD using different criteria. In Cambodia (58) and Nepal (105), this was defined as the use of ≥ six drinks in a single sitting at least monthly using NIAAA definitions, and in Ethiopia (106, 107), as an intake of ≥ six drinks in males and ≥ four drinks in females on a single occasion. The definition of BD differed in a study conducted in the United Kingdom (36), with BD defined as more than eight drinks per session for males and more than six standard drinks per session for females. Some studies examined risky single occasion drinking, defined as ≥ six drinks per single occasion, and at-risk volume drinking, defined as ≥ 21 drinks per week, and risky single occasion drinking at least monthly for men in Switzerland (40).

Hazardous/harmful alcohol use, also known as Harmful/hazardous drinking, probable AUD, risky alcohol use, high-risk drinking, or hazardous, harmful, or dependent alcohol use, was defined as a score of ≥ eight on the Alcohol Use Disorders Identification Test (AUDIT) in most studies including studies conducted in New Zealand (53), Norway (55), Brazil (108, 109), South Africa (61, 102), India (110-113), Kenya (114), Uganda (115), Nepal (116), Ethiopia (63, 117-119), Malaysia (90), Thailand (91, 120), and Suriname (60). This definition is in keeping with the WHO recommended cut-offs for problem drinking on the AUDIT (17). In contrast, one study used an AUDIT score > four to define hazardous, harmful, and high-risk drinking for females in Mozambique (121).

For peer review only

For peer review only - http://bmjopen.bmj.com/site/about/guidelines.xhtml

We noted more variability in the cut-offs used across studies when using short AUDIT forms to define hazardous or harmful drinking. A cut-off score of ≥ five on AUDIT-C (a three-item version of the full AUDIT) was used in South Africa (57) and the UK (54). Risky drinking was defined as 8-12 for males and 6-12 for females on AUDIT-C in Sweden (56), while hazardous alcohol use in Ethiopia (59) was defined as a score of ≥ three on the Fast Alcohol Screening Test (FAST), a 4-item version of the AUDIT. But a different definition was applied for hazardous drinking in Russia (122), which was stated as having any of the following in the past year: having drunk surrogate alcohols (non-beverage alcohols and illegally produced alcohols), having been on zapoi (several days of continuous drunkenness during which one withdraws from the society), having frequent hangovers once or more per month and having drunk spirits daily. One study in China (123) used the Michigan Alcohol Screening Test (MAST) to define cases of alcohol dependence, and it was classified using a MAST score of ≥ five with 1-4 (low), 5-6 (light), and 40-53 (severe).

Prevalence of problem drinking, its pattern, and associated factors

Prevalence and patterns of problem drinking

Six HIC studies assessed heavy drinking (Table 2: Supplementary File 3). Across these studies, the reported prevalence of heavy drinking ranged from 5.0% to 39.9% for males and from < 1.0% to 12.9% for females (33, 34, 37, 47, 74). Heavy drinking was reported by eight out of 47 LMIC studies comprising Brazil (49, 93, 98), South Africa (94, 124), Botswana (24), China (92), and Brazil (52) (Table 3: Supplementary File 4). The prevalence of heavy drinking in these studies ranged from 3.2% to 53% in the overall population, 29.2% to 31% in males, and 3.7% to 17% in females.

HED/BD was reported in nine studies conducted in HICs, including Hong Kong (71), USA (38, 78, 96, 100), UK (36), Singapore (101), Chile (125), and Ireland (39) (Table 2: Supplementary File 3). Across these studies, the prevalence of HED/BD ranged from 14.5% to 24.7% in males, 3.5% to 18% in females, and 13.7% to 86% in the overall sample. HED/BD was also reported by fourteen out of 52 studies from LMICs consisting of South Africa (94, 102, 104), India (103), Cambodia (58), Peru (97), Brazil (98, 99), Nigeria (126), Burkina Faso (127), Nepal (105), and Ethiopia (106, 107, 117) (Table 3: Supplementary File 4). The overall prevalence of HED/BD ranged from 3.7% to 43%.

For peer review only - http://bmjopen.bmj.com/site/about/guidelines.xhtml

The prevalence of HED/BD ranged from 13.7% to 48.2% in males and 2.7% to 15.0% in females.

The AUD, including older terms such as alcohol abuse and alcohol dependence, was reported by 10 out of 29 HIC studies, including Hong Kong (71), Finland (79), Germany (72), Switzerland (40), Israel (74), Australia (75), UK (54), Sweden (77), Chicago, USA (89), and Ireland (39) (Table 2: Supplementary File 3). The prevalence of any lifetime or current AUD ranged from 4.3% to 36.8% in the overall population, 19.8% to 38.3% in males, and 6.3% to 20.6% in females. The prevalence of alcohol abuse ranged from 4% to 4.5%, and alcohol dependence ranged from 0.4% to 12.3% in the overall sample, 6.1% in males, and 6.1% in females.

Likewise, AUD comprising alcohol abuse, alcohol dependence, hazardous, harmful, or dependent alcohol use was reported by 31 of 52 LMIC studies, including South Africa (57, 61, 81, 102), Sri Lanka (80), Ethiopia (50, 51, 59, 63, 117-119), China (123), Brazil (49, 52, 108, 109), India (110-113), Kenya (114), Uganda (115), Nepal (116), Cambodia (58), Malaysia (90), Thailand (91, 120), Suriname (60), and Mozambique (121) (Table 3: Supplementary File 4). Either current or lifetime prevalence of any AUD ranged from 4.1% to 41.0% in the overall sample, from 14.5% to 66.6% in males, and from 2.0% to 33.4% in females. The prevalence of lifetime or current alcohol abuse ranged from 6.2% to 9.0% in the overall sample, estimated at 19.0% in males and 6.0% in females. The prevalence of lifetime or current alcohol dependence ranged from 0.8% to 26.5% in the overall population, from 1.5% to 39.0% in males, and from 0.1% to 19.1% in females.

Factors associated with problem drinking

Most studies from HICs and LMICs identified factors associated with different types of problem drinking. These factors can be grouped into socio-demographic and socio-economic; clinical (medical problems or clinical parameters and mental disorders); substance use and risky behaviours; and psychosocial support, functioning, disability, and quality of life factors (Table 2: Supplementary File 3 and Table 3: Supplementary File 4).

Studies from both HICs and LMICs examined a range of socio-demographic factors associated with problem drinking, but the nature and direction of the relationship were inconsistent across studies. Seven out of 29 studies in HICs found that age was

For peer review only - http://bmjopen.bmj.com/site/about/guidelines.xhtml
For peer review only

associated with problem drinking. Some studies found that older age was associated with heavy drinking (35, 78), while others found that this association existed for men but not women (71). In contrast, other studies reported associations between problem drinking and young adulthood (74, 75), with some studies noting that alcohol use declined with age (56), and age was associated with abstention among women (37) and inversely associated with heavy drinking among men (33, 34). Furthermore, nineteen out of 52 studies in LMICs found that age was associated with problem drinking. Some studies reported that older age was associated with alcohol use and different types of problem drinking (49, 51, 59, 93, 102-104, 113-116, 128, 129), while others found that this association existed for younger age or early adulthood (58, 61, 93, 97, 118, 127).

Several studies found associations between male sex and problem drinking. Seven studies from HICs (35, 56, 72, 74, 75, 78, 89) found that male sex was associated with alcohol use and various types of problem drinking. Another nineteen studies from LMICs found that male sex was associated with different forms of problem drinking (24, 50, 51, 57-59, 90, 93, 94, 105, 106, 109, 110, 114, 117-119, 127, 128).

Some studies from HICs found associations between not being in a relationship and problem drinking, including studies conducted in Australia (75), Israel (74), and China (71). Included studies from LMICs also reported associations between not being in a relationship and various types of AUD (50, 60, 81, 99, 103, 104, 116, 124). In contrast, other studies found that these associations existed for being in a relationship (24, 106, 121) and age-gap relationships (24).

In terms of socio-economic and environmental indicators, only a couple of studies from HICs examined associations between problem drinking and factors like educational attainment (33, 34, 76), employment (71), being immigrants (74), lower (37) or higher (34) income, location (33, 34), or higher neighborhood alcohol outlet density (38). Thirteen included studies from LMICs found that education was associated with problem drinking, with some studies finding that a lower educational level was associated with alcohol abuse and heavy drinking (49, 51, 60, 102, 103, 113, 122, 130). In contrast, others found that this association existed for higher educational levels (24, 61, 97, 99, 129). Thirty-three studies conducted in LMICs examined associations between problem drinking and economic factors, finding equivocal results. While several studies found

For peer review only

associations between lower income (49, 50, 80, 81, 93, 102, 103, 128, 130) or unemployment (62, 122) and problem drinking, others found associations between problem drinking and higher income (57, 58, 94, 102, 107, 108, 110, 121, 122, 128, 131) or being employed (51, 58, 60, 105, 107, 110, 115-117, 127, 129). Only a few studies from LMICs examined associations between factors like religious affiliation (50, 90, 109, 129, 130), living in urban or rural setting and location (61, 102, 106, 107, 113); ethnicity and race (49, 50, 57, 61, 93, 94, 102, 105, 116); household living circumstances (49, 104) and problem drinking.

Three studies conducted in HICs (75) and fifteen in LMICs (50, 59, 61, 63, 80, 90, 93, 98, 108, 115-119, 121) found associations between mental disorders and different forms of problem drinking. Only one HIC study found associations between medical problems like higher BMI and being non-diabetic than diabetic (37) and problem drinking. In contrast, eight studies from LMICs found associations between medical problems like chronic disease (63, 93), high blood pressure (92, 123), obesity (94), self-reported physical comorbidities (113), traffic injury (131), and problem drinking. Only a few studies from LMICs found associations between problem drinking and less psychosocial support (59, 118, 119), more impaired functioning, disability, poorer quality of life, cognitive impairment, and poor sleep quality (63, 99, 112, 116, 117). In terms of other substance use factors, seven studies were conducted in HICs (33-35, 71, 75, 78, 79), and seventeen studies from LMICs (50, 57, 61, 62, 80, 93, 94, 104, 106-108, 113, 116, 118, 119, 127, 128) reported associations between cigarette smoking, current khat use, other substance use and various types of problem drinking.

DISCUSSION

In this scoping review, we identified 81 population-based studies (29 from HICs and 52 from LMICs) examining the prevalence of alcohol consumption and problem drinking, assessment methods, and factors associated with problem drinking. Based on the publication year of included articles, there were more than triple the number of published articles in the last decade compared to the previous decade. This increase in publications over time implies that researchers are more interested and involved in alcohol use studies than before.

For peer review only

Despite this growing body of studies on problem drinking prevalence and alcohol measurement, this review highlights significant heterogeneity of study designs, measures, and outcomes that hamper the synthesis of evidence on alcohol prevalence and associated harms across studies. The development of AUDIT with the WHO collaborative project (17) attempted to solve this heterogeneity in measurements of problem drinking, but the uptake has not been significant.

Such a synthesis of the evidence on alcohol prevalence and alcohol-related harms is needed to convince policymakers to take action to reduce population-level alcohol use. More specifically, this review identified significant heterogeneity and inconsistency in how various forms of problem drinking were defined and measured (24, 33, 35-37, 39, 40, 47, 49, 57, 58, 71, 76, 78, 92-107) which aligns with previous reviews (2). For example, this review found substantial variations in how problem drinking was conceptualised, ranging from heavy drinking, HED/BD, alcohol abuse, alcohol dependence, and AUD and these all were measured with diverse measurement tools like quantity/frequency questions, risky single occasion drinking criteria, screening tools, or structured diagnostic interviews (33-40, 46, 49-63, 71-81, 89-91, 94, 97-99, 102, 108-123, 129, 131). These tools also were variable in the timeframe used to assess problem drinking, with the assessment period ranging from days, weeks, months, or years among the studies included in this review (33-40, 62, 63, 94, 97-99, 109, 129, 131).

This variability in how alcohol use and various forms of problem drinking are defined and measured is a significant weakness in the literature, with previous studies noting a lack of attention to the validity of alcohol screening tools and questionnaires (132). It is crucial to have a uniform and precise definition of problem drinking that can be applied across studies. This approach will allow for a more accurate estimation of prevalence and more effective identification of people with problem drinking, and it will enhance the robustness of the evidence base on which to advocate for alcohol harm reduction.

Many challenges in understanding the true prevalence of problem drinking arise from different definitions and inconsistent approaches to measuring it (2). This was evident in the current review, where we noted considerable differences in the prevalence estimates for problem drinking, partly due to variability in how problem drinking was conceptualised and measured.

For peer review only

Harmonized measures and consensus on the best ways of measuring alcohol use and problem drinking would aid with comparative studies of problem drinking prevalence. Despite the difficulties and challenges associated with building consensus on the best measures for assessing problem drinking and various indicators of problem drinking development, there is an increasing interest in developing agreement on this topic (133). Notably, even if consensus is reached on which measures of problem drinking to use, these self-report measures would be subject to reporting bias, specifically under-reporting or over-reporting of alcohol consumption. These self-report measures can be supplemented with objective measures of alcohol use (alcohol biomarkers) such as Phosphatidylethanol (PEth) (134-139) as there are emerging studies focused on incorporating self-report alcohol use measures with alcohol biomarkers like PEth for valid assessment of problem drinking (137-150).

Problem drinking is affected by numerous factors at population and individual levels, and identifying these factors is important for informing the design of harm minimization interventions (28). The factors associated with problem drinking from our review, summarized as socio-demographic and economic characteristics (age, sex, relationship status, education, employment, income level, religion, race, location, and alcohol outlet density), clinical factors (medical problems, mental disorders, substance use), and quality of life fit into the biopsychosocial model used in medicine, psychiatry, and psychology to understand health and illness (151, 152). Similar to varied measures and definitions, reported factors associated with problem drinking in the current review revealed an irreconcilable and mixed nature.

It is important to note that this review has weaknesses concerning the examination of factors associated with problem drinking, including the use of less powerful statistical tests (nonparametric tests) or no use of statistical tests (36, 39, 47, 48, 50, 53, 89, 100, 111, 115, 126, 153), only a few variables were modeled to control confounding (73, 79, 91, 97, 112, 113, 125, 127), use of non-validated tools that could result in measurement errors (33, 35, 36, 49, 81, 95, 105, 119, 129), sampling only (predominantly) males or females that could cause selection bias (55, 63, 77, 113, 121, 129), high attrition rate from the study (38, 77, 130), and small sample size (58, 63, 90, 109, 110).

For peer review only - http://bmjopen.bmj.com/site/about/guidelines.xhtml

As the way forward, prospective cohort studies that address these methodological limitations and examine the correlates and consequences of problem drinking are needed to guide the design of alcohol harm minimization interventions. The inconsistency reported in the current scoping review requires a united effort among researchers to refine alcohol use assessment methods (measurements) to make them clearer and systematize definitions. Hence, future studies could focus on contextual adoption/adaptation of WHO-recommended and widely available tools like AUDIT or its shortened versions. Suppose the challenges of measuring and defining the problem drinking are addressed. In that case, we can improve the validity and reliability of forthcoming studies, which will extensively enhance our knowledge of problematic alcohol use.

The implication of understanding the heterogeneous nature of measurements and definitions of problem drinking as a gap in the current review will inform the need to develop uniform measurement tools and standard definitions. Besides, knowledge of the challenges of problem drinking studies implies the planning of realistic strategies of prevention, treatment, and intervention to minimize alcohol-related harms.

Strengths and limitations

Our scoping review has several strengths. The review protocol was registered at Open Science Framework (OSF), and we followed PRISMA-ScR guidelines in our scoping review. A comprehensive search strategy was employed to locate global studies. We decided to critically appraise the quality of the included studies, though it is not mandatory in the scoping reviews. This scoping review has several limitations. First, to make our review more feasible, we included only community-based studies, and studies conducted at institutions like hospitals, primary health care services, addiction centers, and colleges/universities were not included, so comparison of findings across these populations was difficult. Second, the reports of this review may be limited to the inclusion criteria employed, in which only published articles written in English were included. Accordingly, publication bias is possible as unpublished reports might have been missed on alcohol use and related conditions.

For peer review only

CONCLUSIONS

This review highlights the heterogeneity of conceptualization, measurement, associated factors, reporting of problem drinking, and methodological weaknesses across included studies, which limits our confidence in the accuracy of prevalence estimates for problem drinking, our ability to compare findings across studies, and pool data for pooled prevalence estimates. Due to the community-based and cross-sectional nature of the included studies, results of alcohol-related harms are missing in our review, which is our target area in our subsequent longitudinal studies. Future alcohol use-related research could improve the quality and reliability of findings by strictly following a priori proposed methods and protocols like using valid alcohol use measures, applying appropriate statistical tests, controlling possible confounders, minimizing selection bias, and using a sufficiently large and justifiable sample size.

Abbreviations

AA: Alcohol abuse; AD: Alcohol dependence; ASSIST: Alcohol, Smoking, and Substance Involvement Screening Test; AUD: Alcohol use disorder; AUDIT: Alcohol Use Disorder Identification Test; BD: Binge drinking; CIDI: Composite International Diagnostic Interview; DSM-III/DSM-III-R: The Diagnostic and Statistical Manual of Mental Disorders, Third Edition; DSM-IV/DSM-5: Diagnostic and Statistical Manual of Mental Disorders 4th and 5th edition; HD: Heavyy drinking; HED: Heavy episodic drinking; HED/BD: heavy episodic or binge drinking; HICs: High-Income countries; ICD: The International Classification of Diseases; LMICs: Low-and Middle-Income countries; MINI: Mini International Neuropsychiatric Interview; MRC: Medical Research Council; PD: Problem drinking; PHC: Primary Health Care; PRISMA-ScR: Preferred Reporting Items for Systematic reviews and Meta-Analyses extension for Scoping Reviews; QF: quantity/frequency questionnaires; RSOD: risky single occasion drinking criteria; SCID: Structured Clinical Interview for DSM Disorders; WHO: World Health Organization

Acknowledgments

Our appreciation is dedicated to AMARI (African Mental heAlth Research Initiative) and Addis Ababa University (AAU) for providing training to Kefyalew Dagne in "Systematic Review & Meta-Analysis." The authors would like to acknowledge the Ethiopian Public

For peer review only

For peer review only - http://bmjopen.bmj.com/site/about/guidelines.xhtml

Health Association (EPHA) Annual Scientific Conference for providing the opportunity to present this research at their 34th (2023) conference.

Author Contributions

KD was involved in the project's conceptualization, writing the protocol, developing a search strategy, searching, screening, and extracting included articles, synthesizing the results, writing the discussion section of the manuscript, and harmonizing the entire document. ST approved the conceptualized research project, the protocol, and the draft manuscript. BM reviewed the search strategy and provided in-depth reviews of the manuscript. AM was involved in screening and extracting included articles. All authors involved read and approved the final manuscript.

Funding

Kefylew Dagne was supported through AMARI, funded through the DELTAS Africa Initiative (DEL-15-01). The DELTAS Africa Initiative is an independent funding scheme of the African Academy of Sciences (AAS)'s Alliance for Accelerating Excellence in Science in Africa (AESA) and supported by the New Partnership for Africa's Development Planning and Coordinating Agency (NEPAD Agency) with funding from the Wellcome Trust (DEL-15-01) and the UK government. The views expressed in this publication are those of the author (s) and not necessarily those of AAS, NEPAD Agency, Wellcome Trust, or the UK government.

Availability of data and materials

All relevant materials and data supporting the results of this study are contained within the manuscript, and relevant documents will be available upon request.

Ethics approval and consent to participate

Not applicable.

Consent for publication

Not applicable.

Competing interests

The authors declare that they have no competing interests.

Author Details

1 Department of Psychiatry, School of Medicine, College of Health Sciences, Addis Ababa University, Addis Ababa, Ethiopia

For peer review only

2 Department of Psychiatry, College of Health Sciences and Medicine, Debre Berhan University, Debre Berhan, Ethiopia

3 Curtin enAble Institute, Faculty of Health Sciences, Curtin University, Kent Street, Bentley, WA 6102, Australia

4 Alcohol, Tobacco and Other Drug Research Unit, South African Medical Research Council, Cape Town, South Africa

5 Division of Addiction Psychiatry, Department of Psychiatry and Mental Health, University of Cape Town, Cape Town, South Africa

Figure Legends

Figure 1: PRISMA flow diagram of included studies in the scoping review, 2023

References

1. Toner P, Böhnke JR, Andersen P, McCambridge J. Alcohol screening and assessment measures for young people: A systematic review and meta-analysis of validation studies. Drug and alcohol dependence. 2019;202:39-49.
2. Griswold MG, Fullman N, Hawley C, Arian N, Zimsen SR, Tymeson HD, et al. Alcohol use and burden for 195 countries and territories, 1990–2016: a systematic analysis for the Global Burden of Disease Study 2016. The Lancet. 2018;392(10152):1015-35.
3. World Health Organization. Global status report on alcohol and health 2018: World Health Organization; 2019.
4. World Health Organization. Management of Substance Abuse Unit. Global status report on alcohol and health, 2014: World Health Organization; 2014.
5. Corrao G, Bagnardi V, Zambon A, La Vecchia C. A meta-analysis of alcohol consumption and the risk of 15 diseases. Preventive medicine. 2004;38(5):613-9.
6. Low WY, Lee YK, Samy AL. Non-communicable diseases in the Asia-Pacific region: Prevalence, risk factors and community-based prevention. Int J Occup Med Environ Health. 2015;28(1):20-6.
7. HELZER JE, PRZYBECK TR. The co–occurrence of alcoholism with other psychiatric disorders in the general population and its impact on treatment Journal of Studies on Alcohol 1988;49:219–24.
8. Fone D, Dunstan F, White J, Webster C, Rodgers S, Lee S, et al. Change in alcohol outlet density and alcohol-related harm to population health (CHALICE). BMC public health. 2012;12:428.
9. Lester L, Baker R, Coupland C, Orton E. Alcohol misuse and injury outcomes in young people aged 10–24. Journal of Adolescent Health. 2018;62(4):450-6.
10. Newbury-Birch D, Ferguson J, Connor N, Divers A, Waller G. A rapid systematic review of worldwide alcohol use disorders and brief alcohol interventions in the criminal justice system. Frontiers in psychiatry. 2022;13.
11. Hagman BT, Falk D, Litten R, Koob GF. Defining recovery from alcohol use disorder: development of an NIAAA research definition. American Journal of Psychiatry. 2022;179(11):807-13.
12. Enoch M-A, Goldman D. Problem drinking and alcoholism: diagnosis and treatment. American family physician. 2002;65(3):441-9.

For peer review only

13. Van Oers J, Bongers I, Van de Goor L, Garretsen H. Alcohol consumption, alcohol-related problems, problem drinking, and socioeconomic status. Alcohol and alcoholism (Oxford, Oxfordshire). 1999;34(1):78-88.
14. Mosel S. Problem Drinking vs. Alcoholism [updated 25 October 2022. Available from: https://alcohol.org/alcoholism/or-is-it-just-a-problem/.
15. American Addiction Centers. Problem Drinker Defined [updated October 9, 2020. Available from: https://alcoholrehab.com/alcoholism/signs-of-alcoholism/problem-drinker-defined/.
16. HRB National Drugs Library. Research Glossary [Available from: https://www.drugsandalcohol.ie/glossary/ Accessed 6 July 2023.
17. Saunders JB, Aasland OG, Babor TF, De la Fuente JR, Grant M. Development of the alcohol use disorders identification test (AUDIT): WHO collaborative project on early detection of persons with harmful alcohol consumption-II. Addiction (Abingdon, England). 1993;88(6):791-804.
18. Edwards G, Arif A, Hadgson R. Nomenclature and classification of drug-and alcohol-related problems: a WHO Memorandum. Bulletin of the World Health Organization. 1981;59(2):225-42.
19. Organization WH. The ICD-10 classification of mental and behavioural disorders: clinical descriptions and diagnostic guidelines: World Health Organization; 1992.
20. Babor TF, de la Fuente JR, Saunders J, Grant M. The Alcohol Use Disorders Identification Test: Guidelines for use in. Primary care. 2001.
21. Fairbairn N, Wood E, Dobrer S, Dong H, Kerr T, Debeck K. The relationship between hazardous alcohol use and violence among street-involved youth. The American journal on addictions. 2017;26(8):852-8.
22. Reid MC, Fiellin DA, O'Connor PG. Hazardous and harmful alcohol consumption in primary care. Archives of internal medicine. 1999;159(15):1681-9.
23. Tsai J, Ford ES, Li C, Pearson WS, Zhao G. Binge drinking and suboptimal self-rated health among adult drinkers. Alcoholism, clinical and experimental research. 2010;34(8):1465-71.
24. Weiser SD, Leiter K, Heisler M, McFarland W, Percy-de Korte F, DeMonner SM, et al. A population-based study on alcohol and high-risk sexual behaviors in Botswana. PLoS medicine. 2006;3(10):e392.
25. American Psychiatric Association. Diagnostic and statistical manual of mental disorders: DSM-IV: American psychiatric association Washington, DC; 1994.
26. American Psychiatric Association. Diagnostic and statistical manual of mental disorders: DSM-5. Fifth ed: Arlington, VA, American Psychiatric Association; 2013.
27. Iranpour A, Nakhaee N. A review of alcohol-related harms: a recent update. Addiction & health. 2019;11(2):129.
28. Park SH, Kim DJ. Global and regional impacts of alcohol use on public health: Emphasis on alcohol policies. Clinical and molecular hepatology. 2020;26(4):652.
29. Tricco AC, Lillie E, Zarin W, O'Brien KK, Colquhoun H, Levac D, et al. PRISMA extension for scoping reviews (PRISMA-ScR): checklist and explanation. Annals of internal medicine. 2018;169(7):467-73.
30. Ribeiro CM, Beserra BTS, Silva NG, Lima CL, Rocha PRS, Coelho MS, et al. Exposure to endocrine-disrupting chemicals and anthropometric measures of obesity: A systematic review and meta-analysis. BMJ open. 2020;10(6):e033509.
31. Herzog R, Álvarez-Pasquin M, Díaz C, Del Barrio JL, Estrada JM, Gil Á. Are healthcare workers' intentions to vaccinate related to their knowledge, beliefs and attitudes? A systematic review. BMC public health. 2013;13(1):1-17.
32. Dubey VP, Kievišienė J, Rauckiene-Michealsson A, Norkiene S, Razbadauskas A, Agostinis-Sobrinho C. Bullying and Health Related Quality of Life among Adolescents—A Systematic Review. Children. 2022;9(6):766.

For peer review only - http://bmjopen.bmj.com/site/about/guidelines.xhtml

33. Bataille V, Ruidavets JB, Arveiler D, Amouyel P, Ducimetiere P, Perret B, et al. Joint use of clinical parameters, biological markers and cage questionnaire for the identification of heavy drinkers in a large population-based sample. Alcohol and Alcoholism. 2003;38(2):121-7.
34. Coulson CE, Williams LJ, Henry MJ, Berk M, Lubman DI, Brennan SL, et al. Patterns of alcohol use and associated physical and lifestyle characteristics according to new Australian guidelines. The Australian and New Zealand journal of psychiatry. 2010;44(10):946-51.
35. Geels LM, Vink JM, van Beek JH, Bartels M, Willemsen G, Boomsma DI. Increases in alcohol consumption in women and elderly groups: evidence from an epidemiological study. BMC public health. 2013;13:207.
36. Williamson RJ, Sham P, Ball D. Binge drinking trends in a UK community-based sample. Journal of Substance Use. 2003;8(4):234-7.
37. Andrews-Chavez JY, Lee CS, Houser RF, Falcon LM, Tucker KL. Factors associated with alcohol consumption patterns in a Puerto Rican urban cohort. Public health nutrition. 2015;18(3):464-73.
38. Auchincloss AH, Niamatullah S, Adams M, Melly SJ, Li J, Lazo M. Alcohol outlets and alcohol consumption in changing environments: prevalence and changes over time. Substance abuse treatment, prevention, and policy. 2022;17(1):7.
39. O'Dwyer C, Mongan D, Millar SR, Rackard M, Galvin B, Long J, et al. Drinking patterns and the distribution of alcohol-related harms in Ireland: evidence for the prevention paradox. BMC public health. 2019;19(1):1323.
40. Mohler-Kuo M, Foster S, Gmel G, Dey M, Dermota P. DSM-IV and DSM-5 alcohol use disorder among young Swiss men. Addiction (Abingdon, England). 2015;110(3):429-40.
41. Ewing J, Rose B. Identifying the Hidden Alcoholic [w:] Paper read at the 29th International Congress on Alcoholism and Drug Dependence. Australia Butterworth, Sydney. 1970.
42. Ewing JA. Detecting alcoholism: the CAGE questionnaire. Jama. 1984;252(14):1905-7.
43. Mayfield D, McLeod G, Hall P. The CAGE questionnaire: validation of a new alcoholism screening instrument. American journal of psychiatry. 1974;131(10):1121-3.
44. Selzer ML. The Michigan Alcoholism Screening Test: The quest for a new diagnostic instrument. American journal of psychiatry. 1971;127(12):1653-8.
45. Pokorny AD, Miller BA, Kaplan HB. The brief MAST: A shortened version of the Michigan Alcoholism Screening Test. American journal of psychiatry. 1972;129(3):342-5.
46. Group WAW. The alcohol, smoking and substance involvement screening test (ASSIST): development, reliability and feasibility. Addiction (Abingdon, England). 2002;97(9):1183-94.
47. Aalto M, Seppa K, Kiianmaa K, Sillanaukee P. Drinking habits and prevalence of heavy drinking among primary health care outpatients and general population. Addiction (Abingdon, England). 1999;94(9):1371-9.
48. Aira M, Hartikainen S, Sulkava R. Community prevalence of alcohol use and concomitant use of medication - A source of possible risk in the elderly aged 75 and older? International journal of geriatric psychiatry. 2005;20(7):680-5.
49. Moreira LB, Fuchs FD, Moraes RS, Bredemeier M, Cardozo S, Fuchs SC, et al. Alcoholic beverage consumption and associated factors in Porto Alegre, a southern Brazilian city: a population-based survey. Journal of studies on alcohol. 1996;57(3):253-9.
50. Alem A, Kebede D, Kullgren G. The epidemiology of problem drinking in Butajira, Ethiopia. Acta psychiatrica Scandinavica Supplementum. 1999;397:77-83.
51. Kebede D, Alem A. The epidemiology of alcohol dependence and problem drinking in Addis Ababa, Ethiopia. Acta psychiatrica Scandinavica Supplementum. 1999;397:30-4.
52. Moreira LB, Fuchs FD, Moraes RS, Bredemeier M, Duncan BB. Alcohol intake and blood pressure: The importance of time elapsed since last drink. Journal of hypertension. 1998;16(2):175-80.

For peer review only

53. Foulds J, Wells JE, Lacey C, Adamson S, Mulder R. Harmful drinking and talking about alcohol in primary care: New Zealand population survey findings. Acta psychiatrica Scandinavica. 2012;126(6):434-9.
54. Britton A, Fat LN, Neligan A. The association between alcohol consumption and sleep disorders among older people in the general population. Scientific reports. 2020;10(1):5275.
55. Husberg VH, Hopstock LA, Friborg O, Rosenvinge JH, Bergvik S, Rognmo K. Epidemiology of comorbid hazardous alcohol use and insomnia in 19 185 women and men attending the population-based Tromso Study 2015-2016. BMC public health. 2022;22(1):844.
56. Lindstrom J, Hellstrom C, Simonsson B, Molarius A. Alcohol consumption and self-rated health among older people: population-based study in Sweden. Journal of public health (Oxford, England). 2020;42(4):756-65.
57. Peltzer K, Ramlagan S, Satekge M. Alcohol use, problem drinking and health risk factors among South African youths. Journal of Psychology in Africa. 2012;22(4):671-6.
58. Yeung W, Leong W-Y, Khoun K, Ong W, Sambi S, Lim S-M, et al. Alcohol use disorder and heavy episodic drinking in rural communities in cambodia: Risk factors and community-perceived strategies. Asia-Pacific Journal of Public Health. 2015;27(8):835-47.
59. Teferra S, Medhin G, Selamu M, Bhana A, Hanlon C, Fekadu A. Hazardous alcohol use and associated factors in a rural Ethiopian district: a cross-sectional community survey. BMC public health. 2016;16:218.
60. Jadnanansing R, Blankers M, Dwarkasing R, Etwaroo K, Lumsden V, Dekker J, et al. Prevalence of substance use disorders in an urban and a rural area in Suriname. Trop Med Health. 2021;49(1):12.
61. Pengpid S, Peltzer K, Ramlagan S. Prevalence and correlates of hazardous, harmful or dependent alcohol use and drug use amongst persons 15 years and older in South Africa: Results of a national survey in 2017. African journal of primary health care & family medicine. 2021;13(1):e1-e8.
62. Bete T, Lami M, Negash A, Eyeberu A, Birhanu A, Berhanu B, et al. Current alcohol, tobacco, and khat use and associated factors among adults living in Harari regional state, eastern Ethiopia: A community-based cross-sectional study. Frontiers in psychiatry. 2022;13:955371.
63. Wolde A. Alcohol Use Disorder and Associated Factors Among Elderly in Ethiopia. Substance Abuse: Research and Treatment. 2023;17.
64. Organization WH, Organization WH. CIDI-interview (version 1.0),(b) CIDI-user manual,(c) CIDI-training manual,(d) CIDI-computer programs. Geneva: World Health Organization.
65. Robins L, Wittchen H, Wing J, Sartorius N, Pull C, Towle L, et al. The composite international diagnostic interview (CIDI) reliability and applicability in different countries. Psychiatry: A world perspective. 1990;1:118-24.
66. Robins LN, Wing J, Wittchen HU, Helzer JE, Babor TF, Burke J, et al. The Composite International Diagnostic Interview: an epidemiologic instrument suitable for use in conjunction with different diagnostic systems and in different cultures. Archives of general psychiatry. 1988;45(12):1069-77.
67. Wittchen H-U, Robins LN, Cottler LB, Sartorius N, Burke JD, Regier D. Cross-cultural feasibility, reliability and sources of variance of the Composite International Diagnostic Interview (CIDI). The British Journal of Psychiatry. 1991;159(5):645-53.
68. Kessler RC, Üstün TB. The world mental health (WMH) survey initiative version of the world health organization (WHO) composite international diagnostic interview (CIDI). International journal of methods in psychiatric research. 2004;13(2):93-121.
69. WHO. The ICD-10 classification of mental and behavioural disorders: clinical descriptions and diagnostic guidelines: World Health Organization; 1992.
70. Reed GM, First MB, Kogan CS, Hyman SE, Gureje O, Gaebel W, et al. Innovations and changes in the ICD-11 classification of mental, behavioural and neurodevelopmental disorders. World psychiatry. 2019;18(1):3-19.

For peer review only

For peer review only - http://bmjopen.bmj.com/site/about/guidelines.xhtml

71. Kim JH, Lee S, Chow J, Lau J, Tsang A, Choi J, et al. Prevalence and the factors associated with binge drinking, alcohol abuse, and alcohol dependence: a population-based study of Chinese adults in Hong Kong. Alcohol and alcoholism (Oxford, Oxfordshire). 2008;43(3):360-70.
72. Meyer C, Rumpf HJ, Hapke U, Dilling H, John U. Prevalence of alcohol consumption, abuse and dependence in a country with high per capita consumption: findings from the German TACOS study. Transitions in Alcohol Consumption and Smoking. Social psychiatry and psychiatric epidemiology. 2000;35(12):539-47.
73. Bott K, Meyer C, Rumpf H-J, Hapke U, John U. Psychiatric Disorders among At-Risk Consumers of Alcohol in the General Population. Journal of studies on alcohol. 2005;66(2):246-53.
74. Neumark YD, Lopez-Quintero C, Grinshpoon A, Levinson D. Alcohol drinking patterns and prevalence of alcohol-abuse and dependence in the Israel National Health Survey. The Israel journal of psychiatry and related sciences. 2007;44(2):126-35.
75. Proodfoot H, Teeson M. Who seeks treatment for alcohol dependence? Findings form the Australian National Survey of Mental Health and Wellbeing. Social psychiatry and psychiatric epidemiology. 2002;37(10):451-6.
76. Veerbeek MA, Ten Have M, van Dorsselaer SA, Oude Voshaar RC, Rhebergen D, Willemse BM. Differences in alcohol use between younger and older people: Results from a general population study. Drug and alcohol dependence. 2019;202:18-23.
77. Lundin A, Waern M, Love J, Lovestad S, Hensing G, Danielsson AK. Towards ICD-11 for alcohol dependence: Diagnostic agreement with ICD-10, DSM-5, DSM-IV, DSM-III-R and DSM-III diagnoses in a Swedish general population of women. Drug and alcohol dependence. 2021;227:108925.
78. Chou KL, Liang K, Mackenzie CS. Binge drinking and axis I psychiatric disorders in community-dwelling middle-aged and older adults: Results from the National Epidemiologic Survey on Alcohol and Related Conditions (NESARC). Journal of Clinical Psychiatry. 2011;72(5):640-7.
79. Latvala A, Tuulio-Henriksson A, Perala J, Saarni SI, Aalto-Setala T, Aro H, et al. Prevalence and correlates of alcohol and other substance use disorders in young adulthood: A population-based study. BMC psychiatry. 2009;9:73.
80. Zavos HM, Siribaddana S, Ball HA, Lynskey MT, Sumathipala A, Rijsdijk FV, et al. The prevalence and correlates of alcohol use and alcohol use disorders: a population based study in Colombo, Sri Lanka. BMC psychiatry. 2015;15:158.
81. Andersson LMC, Twum-Antwi A, Staland-Nyman C, van Rooyen D. Prevalence and socioeconomic characteristics of alcohol disorders among men and women in the Eastern Cape Province, South Africa. Health & social care in the community. 2018;26(1):e143-e53.
82. Grant BF, Dawson DA, Hasin DS. The alcohol use disorder and associated disabilities interview schedule-DSM-IV version. Bethesda, MD: National Institute on Alcohol Abuse and Alcoholism. 2001.
83. Robins LN, Helzer JE, Croughan J, Ratcliff KS. National Institute of Mental Health diagnostic interview schedule: Its history, characteristics, and validity. Archives of general psychiatry. 1981;38(4):381-9.
84. Spitzer RL, Williams JB, Gibbon M, First MB. The structured clinical interview for DSM-III-R (SCID): I: history, rationale, and description. Archives of general psychiatry. 1992;49(8):624-9.
85. First MB, Spitzer RL, Gibbon M, Williams JB. Computer-assisted SCID-Clinician version. 1997.
86. Sheehan D, Janavs J, Baker R, Harnett-Sheehan K, Knapp E, Sheehan M. Mini international neuropsychiatric interview. Tampa: University of South Florida. 1994.
87. Sheehan D, Lecrubier Y, Sheehan KH, Janavs J, Weiller E, Keskiner A, et al. The validity of the Mini International Neuropsychiatric Interview (MINI) according to the SCID-P and its reliability. European psychiatry. 1997;12(5):232-41.

For peer review only

88. Sheehan DV, Lecrubier Y, Sheehan KH, Amorim P, Janavs J, Weiller E, et al. The Mini-International Neuropsychiatric Interview (MINI): the development and validation of a structured diagnostic psychiatric interview for DSM-IV and ICD-10. Journal of clinical psychiatry. 1998;59(20):22-33.
89. Mondi CF, Giovanelli A, Ou SR, Reynolds AJ. Psychiatric and substance use disorders in a predominately low-income, black sample in early midlife. Journal of psychiatric research. 2022;148:332-9.
90. Abd Rashid RB, Mohd Daud MNB, Guad RM, Gan SH, Wan Husin W, Giloi N, et al. Prevalence and risk factors associated with alcohol consumption among indigenous people in Sabah Borneo Island. The Australian journal of rural health. 2021;29(3):464-72.
91. Assanangkornchai S, Nontarak J, Aekplakorn W, Chariyalertsak S, Kessomboon P, Taneepanichskul S. Socio-economic inequalities in the association between alcohol use disorder and depressive disorder among Thai adults: a population-based study. BMC psychiatry. 2020;20(1):553.
92. Ding L, Liang Y, Tan ECK, Hu Y, Zhang C, Liu Y, et al. Smoking, heavy drinking, physical inactivity, and obesity among middle-aged and older adults in China: cross-sectional findings from the baseline survey of CHARLS 2011-2012. BMC public health. 2020;20(1):1062.
93. Dias da Costa JS, Silveira MF, Gazalle FK, Oliveira SS, Hallal PC, Menezes AMB, et al. Heavy alcohol consumptions and associated factors: A population-based study. Revista de saude publica. 2004;38(2):284-91.
94. Peltzer K, Phaswana-Mafuya N. Problem drinking and associated factors in older adults in South Africa. African journal of psychiatry. 2013;16(2):104-9.
95. Janghorbani M, Ho SY, Lam TH, Janus ED. Prevalence and correlates of alcohol use: A populationbased study in Hong Kong. Addiction (Abingdon, England). 2003;98(2):215-24.
96. Miller JW, Gfroerer JC, Brewer RD, Naimi TS, Mokdad A, Giles WH. Prevalence of adult binge drinking: a comparison of two national surveys. American journal of preventive medicine. 2004;27(3):197-204.
97. Hernandez-Vasquez A, Chacon-Torrico H, Vargas-Fernandez R, Grendas LN, Bendezu-Quispe G. Gender Differences in the Factors Associated with Alcohol Binge Drinking: A Population-Based Analysis in a Latin American Country. International journal of environmental research and public health. 2022;19(9):4931.
98. Oancea SC, de Oliveira GD, Sukumaran P, Vogeltanz-Holm N, Nucci LB. The association between alcohol consumption and self-reported current depression among adults residing in Brazil. Journal of public health (Oxford, England). 2021;43(2):e204-e12.
99. Prais HAC, De Loyola Filho AI, Firmo JOA, Lima-Costa MF, Uchoa E. A population-based study on binge drinking among elderly Brazilian men: Evidence from the Belo Horizonte and Bambui health surveys. Revista Brasileira de Psiquiatria. 2008;30(2):118-23.
100. Shockey TM, Esser MB. Binge Drinking by Occupation Groups among Currently Employed U.S. Adults in 32 States, 2013-2016. Substance use & misuse. 2020;55(12):1968-79.
101. Lee YY, Wang P, Abdin E, Chang S, Shafie S, Sambasivam R, et al. Prevalence of binge drinking and its association with mental health conditions and quality of life in Singapore. Addictive behaviors. 2020;100:106114.
102. Peltzer K, Davids A, Njuho P. Alcohol use and problem drinking in South Africa: findings from a national population-based survey. African journal of psychiatry. 2011;14(1):30-7.
103. Pillai A, Nayak MB, Greenfield TK, Bond JC, Nadkarni A, Patel V. Patterns of alcohol use, their correlates, and impact in male drinkers: a population-based survey from Goa, India. Social psychiatry and psychiatric epidemiology. 2013;48(2):275-82.
104. Vellios N, Van Walbeek C. Self-reported alcohol use and binge drinking in South Africa: Evidence from the National Income Dynamics Study, 2014-2015. South African Medical Journal. 2018;108(1):33-9.

For peer review only

105. Dahal S, Sah RB, Niraula SR, Karkee R, Chakravartty A. Prevalence and determinants of noncommunicable disease risk factors among adult population of Kathmandu. PloS one. 2021;16(9 September):e0257037.
106. Getachew T, Defar A, Teklie H, Gonfa G, Bekele A, Bekele A, et al. Magnitude and predictors of excessive alcohol use in Ethiopia: findings from the 2015 national non-communicable diseases STEPS survey. Ethiopian Journal of Health Development. 2017;31(1):312-9.
107. Gutema BT, Chuka A, Ayele G, Tariku EZ, Aschalew Z, Baharu A, et al. Prevalence of heavy episodic drinking and associated factors among adults residing in Arba Minch health and demographic surveillance site: a cross sectional study. BMC public health. 2020;20(1):1895.
108. Mendoza-Sassi RA, Beria JU. Prevalence of alcohol use disorders and associated factors: a population-based study using AUDIT in southern Brazil. Addiction (Abingdon, England). 2003;98(6):799-804.
109. Castelo Branco FMF, de Vargas D. Alcohol use patterns and associated variables among the Karipuna indigenous people in the extreme Northern Brazilian Amazon. Journal of ethnicity in substance abuse. 2023;22(1):29-44.
110. Sau A. AUDIT (Alcohol use disorders identification test) to estimate the pattern and correlates of alcohol consumption among the adult population of west bengal, india: A community based cross sectional study. Journal of Clinical and Diagnostic Research. 2017;11(4):LC01-LC4.
111. Jonas JB, Nangia V, Rietschel M, Paul T, Behere P, Panda-Jonas S. Prevalence of depression, suicidal ideation, alcohol intake and nicotine consumption in rural central India. The Central India Eye and Medical Study. PloS one. 2014;9(11).
112. Olickal JJ, Saya GK, Selvaraj R, Chinnakali P. Association of alcohol use with quality of life (QoL): A community based study from Puducherry, India. Clinical Epidemiology and Global Health. 2021;10:100697.
113. Olickal JJ, Selvaraj R, Saya GK, Chinnakali P. Alcohol use among adult men and its impact on personal and family life: findings of a community-based cross-sectional survey and focus groups discussions from a low alcohol taxed region, Puducherry, India. Family practice. 2022;39(2):316-22.
114. Takahashi R, Wilunda C, Magutah K, Mwaura-Tenambergen W, Wilunda B, Perngparn U. Correlates of alcohol consumption in rural western Kenya: A cross-sectional study. BMC psychiatry. 2017;17(1):175.
115. Nalwadda O, Rathod SD, Nakku J, Lund C, Prince M, Kigozi F. Alcohol use in a rural district in Uganda: findings from community-based and facility-based cross-sectional studies. International journal of mental health systems. 2018;12:12.
116. Rathod SD, Luitel NP, Jordans MJD. Prevalence and correlates of alcohol use in a central Nepal district: secondary analysis of a population-based cross-sectional study. Global mental health (Cambridge, England). 2018;5:e37.
117. Zewdu S, Hanlon C, Fekadu A, Medhin G, Teferra S. Treatment gap, help-seeking, stigma and magnitude of alcohol use disorder in rural Ethiopia. Substance abuse treatment, prevention, and policy. 2019;14(1):4.
118. Endashaw Hareru H, Kaso AW, Debela BG, Abebe L, Sisay WTD, Kassa Abebe R, et al. Alcohol use disorder and its associated factors among residents in Southern Ethiopia during the era of COVID-19. SAGE open medicine. 2022;10:20503121221105031.
119. Legas G, Asnakew S, Belete A, Beyene GM, Wubet GM, Bayih WA, et al. Magnitude and correlates of alcohol use disorder in south Gondar zone, northwest Ethiopia: A community based cross-sectional study. PloS one. 2021;16(9):e0257804.
120. Jirapramukpitak T, Prince M, Harpham T. Rural-urban migration, illicit drug use and hazardous/harmful drinking in the young Thai population. Addiction (Abingdon, England). 2008;103(1):91-100.

For peer review only

121. Wainberg M, Oquendo MA, Peratikos MB, Gonzalez-Calvo L, Pinsky I, Duarte CS, et al. Hazardous alcohol use among female heads-of-household in rural Mozambique. Alcohol (Fayetteville, NY). 2018;73:37-44.
122. Tomkins S, Saburova L, Kiryanov N, Andreev E, McKee M, Shkolnikov V, et al. Prevalence and socio-economic distribution of hazardous patterns of alcohol drinking: study of alcohol consumption in men aged 25-54 years in Izhevsk, Russia. Addiction (Abingdon, England). 2007;102(4):544-53.
123. Ji A, Lou P, Dong Z, Xu C, Zhang P, Chang G, et al. The prevalence of alcohol dependence and its association with hypertension: a population-based cross-sectional study4 in Xuzhou city, China. BMC public health. 2018;18(1):364.
124. Trangenstein PJ, Morojele NK, Lombard C, Jernigan DH, Parry CD. Heavy drinking and contextual risk factors among adults in South Africa: findings from the International Alcohol Control study. Substance abuse treatment, prevention, and policy. 2018;13(1):43.
125. Mason-Jones AJ, Cabieses B. Alcohol, binge drinking and associated mental health problems in young urban Chileans. PloS one. 2015;10(4).
126. Aremu T, Anibijuwon IB, John-Akinola YO, Oluwasanu M, Oladepo O. Prevalence and Factors Associated With Alcohol Use in Selected Urban Communities in Ibadan, Nigeria. International quarterly of community health education. 2021:272684x211006515.
127. Bonnechère B, Samadoulougou S, Cisse K, Tassembedo S, Kouanda S, Kirakoya-Samadoulougou F. Alcohol consumption and associated risk factors in Burkina Faso: results of a population-based cross-sectional survey. BMJ open. 2022;12(2):e058005.
128. Lo TQ, Oeltmann JE, Odhiambo FO, Beynon C, Pevzner E, Cain KP, et al. Alcohol use, drunkenness and tobacco smoking in rural western Kenya. Tropical medicine & international health : TM & IH. 2013;18(4):506-15.
129. Tegegne KD, Boke MM, Lakew AZ, Gebeyehu NA, Kassaw MW. Alcohol and khat dual use among male adults in Ethiopia: A multilevel multinomial analysis. PloS one. 2023;18(9):e0290415.
130. Burazeri G, Kark JD. Prevalence and determinants of binge drinking in middle age in a transitional post-communist country: a population-based study in Tirana, Albania. Alcohol and alcoholism (Oxford, Oxfordshire). 2010;45(2):180-7.
131. Rezaei N, Ahmadi N, Shams Beyranvand M, Hasan M, Gohari K, Yoosefi M, et al. Alcohol consumption and related disorders in Iran: Results from the National Surveillance of Non-Communicable Diseases' Survey (STEPs) 2016. PLOS global public health. 2022;2(11):e0000107.
132. Midanik L. The validity of self-reported alcohol consumption and alcohol problems: a literature review. British journal of addiction. 1982;77(4):357-82.
133. Hussong AM, Gottfredson NC, Bauer DJ, Curran PJ, Haroon M, Chandler R, et al. Approaches for creating comparable measures of alcohol use symptoms: Harmonization with eight studies of criminal justice populations. Drug and alcohol dependence. 2019;194:59-68.
134. Litten RZ, Bradley AM, Moss HB. Alcohol biomarkers in applied settings: recent advances and future research opportunities. Alcoholism: Clinical and Experimental Research. 2010;34(6):955-67.
135. Nanau RM, Neuman MG. Biomolecules and biomarkers used in diagnosis of alcohol drinking and in monitoring therapeutic interventions. Biomolecules. 2015;5(3):1339-85.
136. Elrasheed A, Al Ghaferi H, Ali AY. Assessment of alcohol exposure: testing for ethylglucuronide (Etg), ethylsulfate (Ets). Int J Emerg Ment Health. 2017;19.
137. Viel G, Boscolo-Berto R, Cecchetto G, Fais P, Nalesso A, Ferrara SD. Phosphatidylethanol in blood as a marker of chronic alcohol use: a systematic review and meta-analysis. International journal of molecular sciences. 2012;13(11):14788-812.
138. Aradottir S, Asanovska G, Gjerss S, Hansson P, Alling C. Phosphatidylethanol (PEth) concentrations in blood are correlated to reported alcohol intake in alcohol-dependent patients. Alcohol and alcoholism. 2006;41(4):431-7.

For peer review only - http://bmjopen.bmj.com/site/about/guidelines.xhtml

139. Kechagias S, Dernroth DN, Blomgren A, Hansson T, Isaksson A, Walther L, et al. Phosphatidylethanol compared with other blood tests as a biomarker of moderate alcohol consumption in healthy volunteers: a prospective randomized study. Alcohol and Alcoholism. 2015;50(4):399-406.
140. Kader R, Seedat S, Koch J, Parry C. A preliminary investigation of the AUDIT and DUDIT in comparison to biomarkers for alcohol and drug use among HIV-infected clinic attendees in Cape Town, South Africa. African journal of psychiatry. 2012;15(5):346-51.
141. Williams PP, Mathews C, Jordaan E, Washio Y, Terplan M, Parry C. Validity Of Self-reported Alcohol And Other Drug Use Among Pregnant Women Attending Midwife Obstetric Units In The Cape Metropole, South Africa. 2019.
142. Dolman JM, Hawkes ND. Combining the audit questionnaire and biochemical markers to assess alcohol use and risk of alcohol withdrawal in medical inpatients. Alcohol and Alcoholism. 2005;40(6):515-9.
143. Francis JM, Weiss HA, Helander A, Kapiga SH, Changalucha J, Grosskurth H. Comparison of self-reported alcohol use with the alcohol biomarker phosphatidylethanol among young people in northern Tanzania. Drug and alcohol dependence. 2015;156:289-96.
144. Littlefield AK, Brown JL, DiClemente RJ, Safonova P, Sales JM, Rose ES, et al. Phosphatidylethanol (PEth) as a biomarker of alcohol consumption in HIV-infected young Russian women: comparison to self-report assessments of alcohol use. AIDS and behavior. 2017;21:1938-49.
145. Röhricht M, Paschke K, Sack PM, Weinmann W, Thomasius R, Wurst FM. Phosphatidylethanol reliably and objectively quantifies alcohol consumption in adolescents and young adults. Alcoholism: Clinical and Experimental Research. 2020;44(11):2177-86.
146. Jørgenrud B, Kabashi S, Nadezhdin A, Bryun E, Koshkina E, Tetenova E, et al. The association between the alcohol biomarker phosphatidylethanol (PEth) and self-reported alcohol consumption among Russian and Norwegian medical patients. Alcohol and Alcoholism. 2021;56(6):726-36.
147. Piano MR, Tiwari S, Nevoral L, Phillips SA. Phosphatidylethanol levels are elevated and correlate strongly with AUDIT scores in young adult binge drinkers. Alcohol and Alcoholism. 2015;50(5):519-25.
148. Finanger T, Vaaler AE, Spigset O, Aamo TO, Andreassen TN, Gråwe RW, et al. Identification of unhealthy alcohol use by self-report and phosphatidylethanol (PEth) blood concentrations in an acute psychiatric department. BMC psychiatry. 2022;22(1):1-10.
149. McGinnis KA, Tate JP, Bryant KJ, Justice AC, O'Connor PG, Rodriguez-Barradas MC, et al. Change in alcohol use based on self-report and a quantitative biomarker, phosphatidylethanol, in people with HIV. AIDS and behavior. 2022:1-9.
150. Hasken JM, Marais A-S, de Vries MM, Kalberg WO, Buckley D, Parry CD, et al. Assessing the sensitivity and specificity of phosphatidylethanol (PEth) cutoffs to identify alcohol exposed pregnancies. Current Research in Toxicology. 2023;4:100105.
151. Engel GL. The need for a new medical model: a challenge for biomedicine. Science. 1977;196(4286):129-36.
152. Kissin B, Hanson M. The bio-psycho-social perspective in alcoholism. Alcoholism and clinical psychiatry: Springer; 1982. p. 1-19.
153. Sarma PS, Sadanandan R, Thulaseedharan JV, Soman B, Srinivasan K, Varma RP, et al. Prevalence of risk factors of non-communicable diseases in Kerala, India: results of a cross-sectional study. BMJ open. 2019;9(11):e027880.

For peer review only

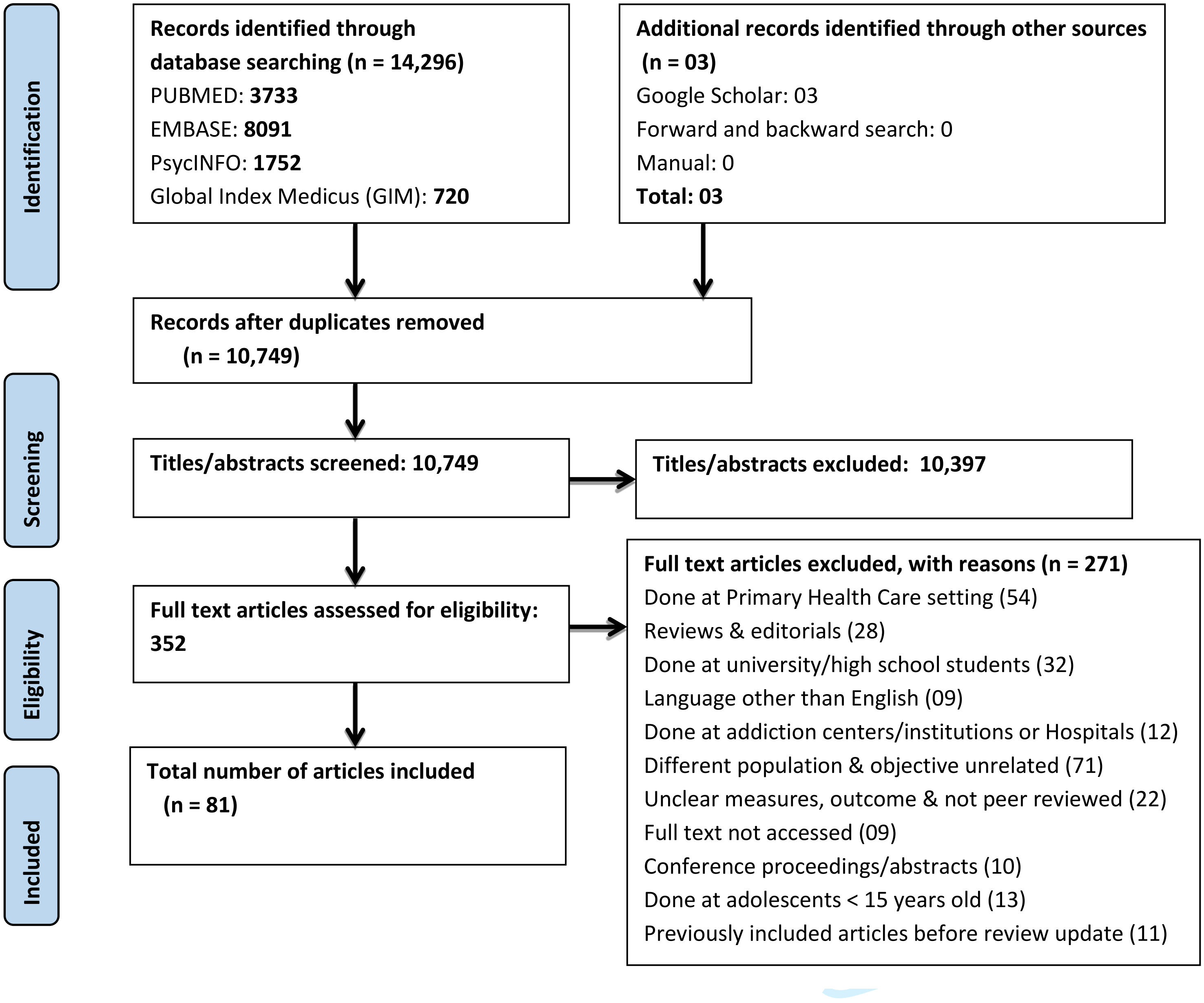

Figure 1: PRISMA flow diagram of included studies in the problem drinking scoping review, 2023.

Supplementary File 1

Search Strategy used for a study "A scoping review of assessment tools for, magnitudes of, and factors associated with problem drinking in population-based studies," 2023.

A) PubMed/MEDLINE:

((((Prevalence [Title/Abstract]) OR "Prevalence" [Mesh])) AND (((alcohol* [Title/Abstract] OR "alcohol abuse" [Title/Abstract] OR "alcohol use" [Title/Abstract] OR "alcohol use disorder" [Title/Abstract] OR "alcohol dependence" [Title/Abstract] OR "alcohol consumption" [Title/Abstract] OR "heavy drinking" [Title/Abstract] OR "risk drinking" [Title/Abstract] OR "harmful drinking" [Title/Abstract] OR "hazardous drinking" [Title/Abstract] OR "binge drinking" [Title/Abstract])) OR ("Alcohol Drinking" [Mesh] OR "Alcoholism" [Mesh] OR "Binge Drinking" [Mesh]))) AND (((Ethiopia [Title/Abstract] OR community-based [Title/Abstract] OR "community based" [Title/Abstract] OR population-based [Title/Abstract] OR "population based" [Title/Abstract])) OR ("Ethiopia"[Mesh] OR "Health Surveys/epidemiology" [Mesh] OR "Population Health/epidemiology" [Mesh]))

B) EMBASE:

1. exp prevalence/
2. prevalence.ti. or prevalence.ab.
3. **1 or 2**
4. exp alcohol consumption/ or exp alcohol/ or exp alcohol abuse/
5. exp alcoholism/ or exp drinking behavior/ or exp binge drinking/
6. (alcohol$ or "alcohol abuse" or "alcohol use" or "alcohol consumption" or "binge drinking").ti. or (alcohol$ or "alcohol abuse" or "alcohol use" or "alcohol consumption" or "binge drinking").ab.
7. **4 or 5 or 6**
8. exp Ethiopia/
9. "community based".mp.
10. "population based".mp.
11. exp primary health care/

For peer review only

12. (Ethiopia or 'community based' or 'population based' or 'primary health care').ti. or (Ethiopia or 'community based' or 'population based' or 'primary health care').ab.

13. 8 or 9 or 10 or 11 or 12

14. 3 and 7 and 13

15. limit 14 to dd=20190826-20220722

16. limit 14 to rd=20190826-20220722

17. 15 or 16

18. limit 14 to dd=20220722-20231125

19. limit 14 to rd=20220722-20231125

20. 18 or 19

C) PsycINFO:

1. prevalence.mp.
2. prevalence.ti. or prevalence.ab.
3. exp "Alcohol Use Disorder"/ or exp Alcohol Abuse/ or exp Alcohol Drinking Patterns/
4. exp Binge Drinking/ or exp Drinking Behavior/ or exp Alcoholism/
5. (alcohol$ or "alcohol abuse" or "alcohol use" or "alcohol consumption" or "binge drinking").ti. or (alcohol$ or "alcohol abuse" or "alcohol use" or "alcohol consumption" or "binge drinking").ab.

6. 1 or 2

7. 3 or 4 or 5

8. ethiopia.mp.
9. "community based".mp.
10. "population based".mp.
11. exp Primary Health Care/
12. (Ethiopia or 'community based' or 'population based' or 'primary health care').ti. or (Ethiopia or 'community based' or 'population based' or 'primary health care').ab.

13. 8 or 9 or 10 or 11 or 12

14. 6 and 7 and 13

15. limit 14 to up=20190826-20220722

For peer review only

16. limit 14 to ch=20190826-20220722

17. 15 or 16

18. limit 14 to up=20220722-20231125

19. limit 14 to ch=20220722-20231125

20. 18 or 19

D) Global Index Medicus (GIM):

(tw:(prevalence)) AND (tw:(alcohol$ OR "alcohol abuse" OR "alcohol use" OR "alcohol consumption" OR "binge drinking")) AND (tw:(Ethiopia OR "community based" OR "population based" OR "primary health care"))

Abbreviations

Date Delivered (dd): the date a citation XML file was produced for distribution to Ovid with the state = "new." The Date Delivered is removed when a record is revised.

Revised Date (rd): the date the citation XML file was produced for distribution to Ovid with the state="update".This date can change if an updated record is delivered to Ovid.

Update Date/Code (up): The date a record was added to the database since the yearly reload completion.

Correction Date (ch): CH field appears in corrected records and contains the date the record was revised.

For peer review only

Supplementary File 2

Newcastle-Ottawa Scale (NOS) adapted for quality assessment of cross-sectional studies for the study "A scoping review of assessment tools for, magnitudes of, and factors associated with problem drinking in population-based studies," 2023.

Selection: (Maximum 5 points/scores/stars)
**1. Representativeness of the sample:**
a. Truly representative of the average in the target population. * (all subjects/consecutive or random sampling)
b. Somewhat representative of the average in the target group. * (non-random sampling)
c. Selected group of users/convenience sample.
d. No description of the derivation of the included subjects (sampling strategy).
**2. Sample size:**
a. Justified and satisfactory (including sample size calculation). * (1 score)
b. Not justified
c. No information provided
**3. Non-respondents:**
a. Proportion of target sample recruited attains pre-specified target or basic summary of non-respondent characteristics in sampling frame recorded. *
b. Unsatisfactory recruitment rate, no summary data on non-respondents.
c. No information provided
**4. Ascertainment of the exposure (risk factor/disease) or screening/surveillance (measurement) tool:**
a. Secure record (medical charts) or validated measurement (screening/surveillance) tool. **
b. Non-validated measurement tool, but the tool is available or described or Self-report. *
c. No description of the measurement tool.
**Comparability: (Maximum 2 stars)**
**1. Comparability of subjects in different outcome groups on the basis of design or analysis. Confounding factors controlled.**
a. Data/results adjusted for relevant predictors/risk factors/confounders e.g., age, sex, marital status, job etc. **

For peer review only

b. Data/results not adjusted for all relevant confounders/risk factors/information not provided.
**Outcome: (Maximum 3 stars)**
**1. Assessment of outcome:**
a. Independent blind (structured) assessment. **
b. Record linkage. **
c. Self report. *
d. No description.
**2. Statistical test:**
a.Statistical test used to analyse the data clearly described, appropriate, and measures of the association presented including confidence intervals and probability level (p-value). *
b.Statistical test not appropriate, not described, or incomplete.

<u>Scoring for cross-sectional Studies:</u>

Very Good Studies: 9-10 points

Good Studies: 7-8 points

Satisfactory Studies: 5-6 points

Unsatisfactory Studies: 0 to 4 points

For peer review only

References

1. Ribeiro CM, Beserra BTS, Silva NG, Lima CL, Rocha PRS, Coelho MS, et al. Exposure to endocrine-disrupting chemicals and anthropometric measures of obesity: A systematic review and meta-analysis. BMJ open. 2020;10(6):e033509.

2. Zhao C, Xing F, Yeo YH, Jin M, Le R, Le M, et al. Only one-third of hepatocellular carcinoma cases are diagnosed via screening or surveillance: a systematic review and meta-analysis. European journal of gastroenterology & hepatology. 2020;32(3):406-19.

3. Patra J, Bhatia M, Suraweera W, Morris SK, Patra C, Gupta PC, et al. Exposure to second-hand smoke and the risk of tuberculosis in children and adults: a systematic review and meta-analysis of 18 observational studies. PLoS medicine. 2015;12(6):e1001835.

4. Modesti P, Reboldi G, Cappuccio F. Newcastle-Ottawa Quality Assessment Scale (adapted for cross sectional studies). PloS one. 2016;11(1):e0147601.

5. Dubey VP, Kievišienė J, Rauckiene-Michealsson A, Norkiene S, Razbadauskas A, Agostinis-Sobrinho C. Bullying and Health Related Quality of Life among Adolescents—A Systematic Review. Children. 2022;9(6):766.

6. Naafs JC, Vendrig LM, Limpens J, Van Der Lee H, Duijnhoven RG, Marchal J, et al. Cognitive outcome in congenital central hypothyroidism: a systematic review with meta-analysis of individual patient data. European journal of endocrinology. 2020;182(3):351-61.

For peer review only

Supplementary File 3

Table 2: Prevalence, associated factors, and pattern of problem drinking in high-income countries (HICs), 2023.

**Author, Year**<br><br>**Country/Location**	**Study Design & Study Setting (population)**	**Participants: Sample size** (Male subjects, %) Mean age (range) in years	**Tools (measures) or questions used**	**Outcomes: (Definition/nature of use)**	**Results & statistical methods used.**
**Aalto et al., 1999**<br><br>**Finland (town of Lahti)**	**Cross-sectional** PHC outpatients & General population (Urban residents)	PHC,2370 (40.3%) OHC,3268 (29.3%) GNP,544 (51.7%) 38-41(20-60) years	Quantity or frequency questionnaires **(QFQs)** (last 2 month) **CAGE**	**Heavy drinking:** **Male:** ≥ 280g of absolute ethanol /24 drinks/week/ &/or ≥ 3 in CAGE. **Women:** ≥ 190g/16 drinks per wk &/or ≥ 2 in CAGE. **Abstinence:** no self-reported drinking at all & no answers to CAGE	**t-test & Chi-square analysis:** **Men:** heavy drinking in PHC, OHC & GNP were 19.5%, 17.3% & 16.4%, respectively. **Women:** corresponding figures were 8.6%, 6.2% & 12.9%.
**Aira et al., 2005**<br><br>**Finland (City of Kuopio)**	**Cross-sectional** home-dwelling elderly persons, **Community-based** (Urban residents)	700 persons (27.4% men) 81 (75-95.7) years	**QFQs** (1 year) & **CAGE**	**Four categories:** Abstainers, < 1 unit/week, 1–7 units/week, & > 7 units/week.	**Chi-square & t-test** (frequencies vs means): 44% had used alcohol during past year (65% of men & 36% of women). ≥ 3 units/occasion used by 2.9% of women & by 11.7% of men.
**Andrews-Chavez et al., 2015**<br><br>**United States (Greater Boston area, MA)**	**Cross-sectional** (Puerto Rican adults, Hispanics). (Urban residents)	1472 adults (29.6% men) ? (45–75) years	**QFQs NIAAA** definitions **(NIAAA guidelines)**	**Lifetime abstainer (LA):** (< **12 drinks** in lifetime) **Former drinker (FD):** **(> 12 drinks** in lifetime, but not currently drinking) **Moderate drinker (MD):** (**Man/women**: ≤14/7drinks per week & ≤ 4/3 drinks/d) **Heavy drinker (HD)**: (**Man/women**:>14/7drinks per week & > 4/3drinks/d)	**A multinomial logistic regression model:** 8% men & 39% women were **LAs;** 40% of men & 25% women **(FDs); &** 21 % men & 8 % of women **(HDs).** **Young men**: likely than older to be MDs. **Women**: higher BMI, age, lower income & psychological acculturation (associated with abstention); age, lower perceived emotional support associated with increased FD; & women without v. with diabetes were more likely to be heavy drinkers.
**Bataille et al., 2003**<br><br>**France (Lille, Strasbourg & Toulouse)**	**Cross-sectional** (3rd MONICA) **Population survey** (Urban/Semi-urban & rural)	3508 subjects (51.0% men) 50.3 (35–64) years	Self-reported **QFQs** French alcohol consumption habits	**Heavy drinkers:** **Men:** ≥ 60g ethanol/day, (6 glasses/d-any drink) & **Women:** ≥ 30g/day (3 glasses/day) **Reference class (RC):** non-drinkers & moderate drinkers together.	**Multivariate analyses:** 14% men & 40.8% women (non-drinkers) 9.0% women & 14.4% of men were HDs. Low educational level, smoking, apoprotein B, HDL, MCV), GGT & CAGE score for men, & living area, age, MCV, GGT & the CAGE score for women were significantly associated with **heavy drinking (HD).**

For peer review only - http://bmjopen.bmj.com/site/about/guidelines.xhtml

**Coulson et al., 2010**<br><br>**Australia (south Eastern)**	**Cohort study** (Geelong Osteoporosis Study, GOS)<br>**Community-based cohort** (secondary data)	1420 men (100%)<br>56 (20 – 93) years	Validated self-report **FFQ**<br>**Mean daily alcohol intake** (Australian National Health & MRC 2009 guidelines)	**Consumption/12 months**: (never, < 1/month, 1–3 days/month, 1–6 days/week & every day<br>**Mean daily alcohol intake** non-drinkers/nil,<br>> 0 but ≤ 2 drinks/ day,<br>> 2 drinks/day<br>**(with in past 12 months)**	**ANOVA & Multivariate analyses:**<br>Age-standardized proportion of non-drinkers was 8.7%, 51.5% consumed ≤ 2 drinks/day (≤ 20g/day), & 39.9% > 2 standard drinks per day (> 20g ethanol/day).<br>**Alcohol use (> 20g/day)** was positively associated with cigarette smoking, weight, higher SES & inversely with age & physical activity.
**Foulds et al., 2012**<br><br>**New Zealand**	**Cross-sectional** (Permanent private dwellers)<br>**Population survey**	12,488 adults (42.2% male)<br>? (≥ 15 years)	**AUDIT**	**Harmful/hazardous drinking (HHD):**<br>Score of ≥ 8 on AUDIT	**Crosstabs & logistic regression models:**<br>**HHD:** 17.7% (men, 25.6%; women, 10.4%);<br>**Overall, 9.4%** of attendees with HHD reported talking about alcohol.
**Geels et al., 2013**<br>**Netherlands**	**Cross-sectional** (All Netherlands Twin Register, NTR registered at a valid address)<br>**Population survey** (Urban)	16,587 subjects (36.5% men)<br>41.6 (18–97) years	**QFQs** (12 mo.)<br><br>Health Council of Netherlands recommended limit<br>CAGE & AUDIT	**Excessive alcohol use:**<br>**Women:** > 14 standard glasses per week<br>**Men:** > 21 drinks/week	**Linear/logistic/multinomial regressions:**<br>>30.0% of men & >20% of women drinking 6–7 times per week)<br>**Women:** 25–45 years had 5.7-5.9% of excessive drinking, & 55–65 years (15.5%)) Older age, sex (male), and initiation of cigarette & cannabis use were predictors of alcohol use
**Janghorbani et al., 2003**<br><br>**Hong Kong (China)**	**Cross-sectional** (Cantonese-speaking adult population)<br>**Population-based** (Urban)	2900 subjects (48.7% men)<br>45.8 (25–74) years	**QFQs** (weekly)	**Heavy drinkers:** men, > 400g & women, > 280g/wk<br>**Light drinkers:** men, < 168g & women, <112g/wk<br>**Moderate drinkers:**<br>Men: ≤ 400g/ ≥ 168g &<br>Women: ≤ 280g/ ≥112g/wk<br>**Binge drinking**: ≥ 5 drinks in a row in the past month.	**GLMs/multiple/logistic regression models:**<br>**Mean weekly alcohol consumption**:<br>64.3g, men & 13.7g, women (P < 0.001).<br>Current drinking vs non-drinking, male sex, smoking (women), HDL, ≤ primary education, diastolic BP & separated/widowed were associated positively with **weekly ethanol consumption.**
**Kim et al., 2008**<br><br>**Hong Kong (China)**	**Cross-sectional** (All Hong Kong Chinese adults)<br>**Population based** (Urban)	9860 adults (50.0% men)<br>28 (18–70) years	Pattern **(QFQs)**<br><br>**CIDI** (Chinese version based on DSM-IV)	**Mean drinking/past year:** < once/wk, 1–3 times/wk, 4+ times/week)<br>**Binge drinking/past mo.** (5 servings of alcohol per one occasion in 30 days)<br>**Alcohol abuse or dependence** (Chinese CIDI)	**Stepwise multivariate logistic regression:**<br>**10.9% of entire sample** reported at least one of AUDs (AA, AD & binge drinking).<br>**Binge drinking :**14.5% in males (18.7% AA & 12.3% AD) & 3.5% in females (16.0% AA & 9.9% AD)<br>**Male binge drinkers** were less likely to be older & students but more likely to be employed in service industry.<br>**Female binge drinker**: less likely to be > 60 years or married & more likely to be smokers<br>**In both genders,** smoking was significantly associated with binge drinking

For peer review only - http://bmjopen.bmj.com/site/about/guidelines.xhtml

**(Chou et al., 2011**<br><br>**United States**	**Prospective study** (subsample of 3-year prospective study**; waves 1 & 2 of NESARC**)<br>**Population-based** (Urban)<br>**secondary data analyses**	13,442 analyzed (40.6% men)<br>? (≥ 50 years)	**QFQs**<br><br>**AUDADIS-IV** (DSM-IV)	**Binge drinking (BD):**<br>≥ 5 drinks/occasion (men)<br>≥ 4 per occasion (women)<br>**Current drinkers:** without BD<br>**Occasional BD:** < monthly in past year) &<br>**Frequent BD:** ≥ 1/month in past year<br>**DSM-IVAUDs** (Alcohol use, AA & AD)	**Multinomial & logistic regression:**<br>BD was 24.7% in men & 12.4% in females.<br>Overall, male respondents were significantly more likely to have BD.<br>Both men & women with occasional BD & frequent BD were significantly more likely than current male/female drinkers without BD to have **alcohol abuse disorder and alcohol dependence disorder (AUDs)**
**Latvala et al., 2009**<br><br>**Finland**	**Cross-sectional** (Finnish young adults)<br>**Population-based** (Urban)	605-diagnostic assessment done (sex unspecified)<br>28.6 (21-35) years	**SCID-I** complemented by medical record data	**Lifetime Substance Use Disorders (SUDs):**<br><br>DSM-IV diagnosis	**t-tests, X^2 tests & logistic regression:**<br>Lifetime AA or AD were 13.1% (19.8% for males & 6.3% for females). And total prevalence of AA & AD alone was 7.6% & 5.6%.<br>Behavioral, affective & parental factors, early initiation of substance use, learning difficulties & lower education were found to be associated with **alcohol & other SUDs.**
**Meyer et al., 2000**<br><br>**Germany (Northern, city of Lubeck)**	**Cross-sectional** of longitudinal project (Adult general population) (Urban)	4075 analyzed (50.2% of men)<br>? (18 to 64 years)	**M-CIDI** (DSM-IV, adapted CIDI)<br>**Ever/current** QFQs	**Hazardous consumption:** 20-40g/d (women) & 30-60g/day (men) and<br>**Harmful consumption:** > 40g/day (women) & > 60g/d (men)<br>**AA or AD**:<br>DSM-IV Diagnosis (M-CIDI diagnostic software)	**Logistic regression analyses:**<br>Lifetime AUDs (4.5% AA, 3.8% AD) & men vs women for AA (8.1% vs 1.0%) & AD (6.0% vs 1.5%) respectively<br>**Hazardous & harmful consumption:** (13.2% lifetime; 6.0% in last 12-months)<br>**Male:** more affected by lifetime AUDs.<br>Association between AUDs & alcohol consumption pattern revealed a weaker relation for AA compared to AD.
**Miller et al., 2004**<br><br>**United States**	**Cross-sectional** (US Adults; BRFSS, telephone survey & NSDUH, an in-person survey)	**355,371** (BRFSS)<br>**87,145** (NSDU) were analyzed (sex unspecified)<br>? (≥ 18 years)	**Pattern (QFQs)**	**Binge drinking**: ≥ 5 drinks on an occasion	**two-tailed t-test:**<br>National binge drinking prevalence was:<br>14.7% for BRFSS and 21.6% for NSDUH<br>Most binge drinkers were male (74% BRFSS, 68% NSDUH) & white, non-Hispanic (73% BRFSS, 76% NSDUH)
**Mohler-Kuo et al., 2015**<br><br>**Switzerland**	**Cohort study** (Young Swiss men from C-SURF)<br>**Population-based** (Rural, 60.3%; Urban, 39.7%)	5943 total sample (100% men)<br>20.0 (18–25) years	DSM-IV & DSM-5 criteria<br><br>QFQs<br><br>RSOD & at-risk volume drinking	**AA & AD (**DSM-IV) &<br>**AUD (**≥ 2 criteria-DSM-5) (12-month prevalence)<br>**RSOD** (≥ 6 drinks/single occasion)<br>**At-risk volume drinking** (≥ 21 drinks/wk & RSOD at least monthly)	**Multinomial logistic regression:**<br>31.7% met DSM-5 AUD (21.2% mild; 10.5% moderate/severe], less than overall DSM-IV criteria for AA & AD (36.8%)<br>Relative to those meeting both DSM-IV & DSM-5 criteria, all other subgroups reported less alcohol and illicit drug use.

For peer review only

**Neumark et al., 2007**<br><br>**Israel**	**Cross-sectional** (Israeli adults)<br>**National population-based survey**	4,859 adults<br>(49.0% men)<br>? (≥ 21 years)	**WMH-CIDI** (lifetime & past 12-month DSM-IV Dx)	**DSM-IV** (AA & AD)<br>**Frequent drinking:** (3 ormore times in one week at least once) in the past year.<br>**Frequent heavy drinking:** consumption of ≥ 3 drinks, ≥ 3 times a week at least once during past year	**Logistic regression models:**<br>**Lifetime AD** was 41%,<br>**Frequent drinking was 5%, & frequent HD was** (6.8% of men & < 1% of women)<br>Lifetime AA/AD was 4.3% (4.0%, AA & 0.4% AD criteria)<br>Significantly higher rates among males (AOR=7.3), younger adults (AOR=5), immigrants (AOR=2.0) & never married (AOR=1.6)
**Proodfoot and Teeson, 2002**<br><br>**Australia**	**Cross-sectional** (Australian National Survey of Mental Health & Wellbeing, NSMHWB)	10,641 respondents<br>(sex unspecified)<br>? (≥ 18 years)	**CIDI 2.1** (modified WHO version)<br>**QFQs**	**DSM-IV Diagnosis for AA & AD**<br>**High level of dependence:** ≥ 4 criteria for dependence.	**Multiple logistic regressions:**<br>AD was 4.1% (males 6.1% & females 2.3%)<br>Variables correlated with AD were male sex, young age (18-34); not being in a married or de facto relationship & having any affective, anxiety or other substance use disorder.
**Veerbeek et al., 2019**<br><br>**Netherlands**	**Cohort study** (Data from, NEMESIS-2<br>**Population-based** (6 categories of urbanicity: very high to very low)	4618 persons<br>(sex unspecified)<br>? (23–70 years)	**CIDI V 3.0**<br>DSM-IV International guidelines for alcohol use definitions	**Alcohol disorder:** AA &/or AD (past 12 months)<br>**Heavy alcohol use:** > 14 drinks/wk (women) & > 21 drinks/wk for men	**Multinomial logistic regression analyses:**<br>Prevalence of heavy alcohol use was higher in older (55–70 years) than younger people (6.7% vs 3.8%), but alcohol disorder was less prevalent (1.3% vs 3.9%).<br>Heavy alcohol use was associated with higher level of education in older adults compared to younger adults.
**Williamson et al., 2003**<br><br>**United Kingdom**	**Cross-sectional** (Subjects from 26 general practices registered with MRC-GPRF)<br>Community-based project in the UK<br><br>**Secondary data**	**20,062** unrelated index subjects<br>(40.0% men)<br>? (20–60 years)	**UK definition** for binge or heavy drinking behaviour &<br><br>**QFQs** for (single session drinking criteria)	**Binge/heavy session drinkers**: males > 8 & females > 6 units/session<br><br>**Non (binge/heavy session) drinking:** not fulfilling session drinking criteria, including abstainers	**No statistical analysis performed**<br>Average number of units of alcohol per week consumed was 16 for men and 8 for women.<br>17% of subjects had binge drinking fashion.<br>(15% for male vs 18% for females)<br>Binge drinking was found to be most prevalent amongst males & females in their 20s (33% of male vs 38% of females).
**Auchincloss et al., 2022**<br><br>**USA (Philadelphia, Pennsylvania metropolitan area)**	**Cross-sectional analyses** (population-based cohort)<br>(Urban setting)	**772 (cross-sectional analyses)**<br>(48% men)<br>? (21–64 years)	Quantity/ Frequency Questions **(QFQs)**<br>RSOD criteria	**BD** (SAMHSA definition): at least one day in past 30 days the person consumed a high volume of alcohol on a single occasion (≥5 alcoholic drinks for males and ≥ 4 for females).	**Logistic regression and Poisson regression**<br>Among alcohol users in either time period, 22% consumed 8 or more drinks per week and 37% reported at least 1 binge occasion in the past 30 days.<br>higher outlet density was associated with more alcohol consumption and residing farther from an outlet was associated with less alcohol consumption.
**Bott et al., 2005**<br><br>**Germany**	**Cross-sectional** (part of a longitudinal study)	4,074 (analysis)<br>(44.9% men)<br>42.7 (18-64 years)	DSM-IV based Munich CIDI (M-CIDI).	**Four alcohol-use groups:**<br>(1) moderate drinkers/ abstainers (MOD/A): < 12	**Multinomial regression analysis (multivariate associations):**<br>9% of participants were at-risk drinkers.

For peer review only

(Lübeck city and its catchment area)	(urban setting)		**Quantity/ frequency index,QFI** (at-risk drinking = Based on the British Medical Association's, 1995, recommendations)	times in their lives or <20g/women & <30g/men pure alcohol/day<br>**(2)** at-risk drinkers (ARD): >20/30g pure alcohol/day<br>(3) DSM-IV criteria for alcohol abuse (AA)<br>(4) DSM-IV criteria for alcohol dependence (AD)	Prevalence rates for at-risk drinkers were 16.9% for affective, 18.1% for anxiety and 17.8% for somatoform disorders.<br>Compared with MOD/A, atrisk drinkers showed a 2-fold increased risk of having a psychiatric disorder. Subjects with AA showed a comparable level of risk & with AD showed an even greater risk. Female at-risk drinkers were twice as likely to have a psychiatric disorder compareed to male.
**Britton et al., 2020**<br><br>**United Kingdom**	**Cross-sectional** (part of Whitehall II study, civil servants at phase 11 (2012–13) (urban setting)	6117 (alcohol & sleep data) (70.9% men) Mean age: 69.4 men, 69.6 women (61–81 years)	**Volume of consumption** (drinks used in last 7 days) **Retrospective alcohol life-course grid** (AUDIT-C)	**Hazardous drinking/HD**: ≥ 5 points on AUDIT-C<br>**Non-drinkers**: didn't drink alcohol in past year.	**Logistic regression:**<br>15.7% of men consumed 21 or more units per week compared to only 2.4% of women.<br>30.5% men & 12.8% women reported HD.<br>men drinking > 21 units/wk or drinking hazardously were more likely to have disturbed sleep than those not drinking in past week or not drinking hazardously.
**Husberg et al., 2022**<br><br>**Norway (Tromsø)**	**Cross-sectional data** (population-based) (Tromsø 1-7, T7 = 2015-2016 (urban setting)	**19,185 (analysis)** (47.5% men) Mean age: 57.2 women, 57.4 men (40-96 years)	**AUDIT:** Hazardous alcohol use (HAU)	Hazardous alcohol use: AUDIT ≥ 8 as a cut-off	**Logistic binomial regression model:**<br>Insomnia was more prevalent among participants with a HAU (24.1%) than without (18.9%).<br>Participants who had HAU had higher odds of insomnia (OR= 1.49).
**Lee et al., 2020**<br><br>**Singapore**	**Cross-sectional** (Singapore Mental Health Study, SMHS 2016) (urban setting)	**6126 (interviewed)** (50% men) ? (18 yrs & above)	**QFQs** (alcohol use) **CIDI 3.0** (mental disorders) **DSM-IV** (diagnosis of mental disorders)	**Bing Drinking (BD):** consumption of 5 or more drinks (male) or 4 or more drinks (female) on a single occasion in the past 12 months.	**Multiple logistic regressions**<br>13.7% reported past-year BD (17.6% of males and 9.8% of females).<br>Moderate associations between BD and mood and anxiety disorders (ORadj =1.8–4.4), were noted, while associations with AUDs were much stronger (ORadj=5.3–9.7).<br>Associations betweenn BD & anxiety disorders were observed exclusively in females (ORadj=2.3–3.3). Binge drinkers reported a lower quality of life compared to their non-binging counterparts.
**Lindstrom et al., 2020**	**Cross-sectional**	11,716 (50.4% men) ? (65-99 years)	AUDIT-C (Alcohol consumption)	non-drinker = 0; moderate drinker = 1–7 (male), 1–5 (female); risk-drinker = 8–12 (male), 6–12 (female).	**Logistic regression analysis**<br>Men (83%) were more prone to drink alcohol compared to women (71%). The prevalence of risk drinking was about 2% for both genders.

For peer review only

For peer review only - http://bmjopen.bmj.com/site/about/guidelines.xhtml

**Sweden**				Non-drinker was not consumed alcohol during the last 12 months.	Alcohol consumption declined with age. Moderate consumption of alcohol was associated with lower probability of poor SRH compared to non-drinking (AOR=0.64 for men) and (AOR= 0.68 for women).
**Lundin et al., 2021**<br><br>**Sweden** (Gothenburg, second largest city in Sweden)	**Longitudinal** (Women and Alcohol in Gothenburg (WAG) Study, cohort in 1986, 1994/2000 & 2013) (urban setting)	**1,614 (baseline)** (100% women) ? (across different age-group?)	CIDI-SAM, ICD-10 & ICD-1, DSM-IV & DSM-5	AUD, alcohol abuse (AA), alcohol dependence (AD) based on CIDI-SAM or (DSM-III, DSM-III-R, DSM-IV, DSM-5, & ICD-10 & ICD-11)	**contingency tables & Cohen's Kappa coefficient (κ)** Baseline: prevalence of lifetime AD was 10.6 % (ICD-11); 4.0 % (ICD-10); 4.3 % (DSM-IV); 7.5 % (DSM-III-R); and 12.3 % (DSM-III). DSM-5 AUD was 14.3 %.
**Mason-Jones and Cabieses, 2015**<br><br>**Chile**	**Cross-sectional**<br><br>(Chilean National Health Survey 2010, ENS 2010) (88% lived in urban settings)	Adolescents (absolute n=435, weighted n = 1860812) Young adults (absolute n = 412, weighted n = 1386 547) (50.3% men) ? (adolescents 15-20 years & young adults 21-25 years).	**QFQs** (Alcohol prevalence in last year, & BD prevalence in last month)	**Alcohol prevalence in last year:** 'yes' labeled as "1" and 'no' labeled as "0". **BD prevalence last month:** had drunk four or more units of alcohol in a single episode in the last 4 weeks.	**Conditional logistic regression models:** 65% of adolescents and 85% of young adults reported drinking alcohol in the last year & of those (who used alcohol in the last year) 83% of adolescents and 86% of young adults reported BD in the previous month. Adolescents who reported binging alcohol were also more likely, compared to young adults, to report being depressed (OR 12.97) or to feel very anxious in the last month.<br>Adolescent females were more likely to report poor life satisfaction in the previous year (OR 8.50), feel depressed (OR 3.41).<br>Being female was also associated with a self-reported diagnosis of depression for both age groups.
**Mondi et al., 2022**<br><br>**USA (Chicago)**	**Cross-sectioal**<br><br>(CLHS data collection, predominately Black sample) (grew up in urban poverty)	**301** CLHS participants (40% men) ? (32-37 years invited to CLHS)	**M.I.N.I. 7.0.2.** (based on DSM-IV & ICD-10 criteria)	DSM-IV & ICD-10 criteria for major depressive disorder, generalized anxiety disorder, post-traumatic stress disorder, substance use disorder, and AUD.	**Independent samples t-tests** Males endorsed significantly higher rates of any AUD within the past 12 months (38.3%) than females (20.6%). Probable prevalence rate for any AUD was 27.7%.
**O'Dwyer et al., 2019**<br><br>**Ireland**	**Cross-sectional** (Data generated from 2013 National Alcohol Diary Survey, NADS)	**4338 drinkers** (49.9% men) ? (18–75 years old)	**RSOD** criteria (HED) **DSM-IV (CIDI) Alcohol-related**	**HED:** consuming 60 g or more of pure alcohol in a single drinking occasion. **Alcohol dependence** (AD) (DSM-IV criteria) Current drinkers, non-	**Crosstabs (Pearson χ2, bivariate assoc.)** There was a relatively even breakdown of low-risk (31.0%), occasional HED (30.6%), and monthly HED (31.5%) drinkers.<br>AD constituted 6.9% of all drinkers.<br>Overall, 29% of drinkers experienced at least one harm

For peer review only - http://bmjopen.bmj.com/site/about/guidelines.xhtml

For peer review only

			**harms/ARH (8 questions)**	drinkers, monthly HED, occasional HED, low-risk drinkers, ARH	from their own drinking in last year. Respondents who were AD had a greater individual risk of experiencing each harm.
**Shockey and Esser, 2020** **USA** (District of Columbia and territories)	**Cross-sectional** (U.S. employed adults who resided in 32 states, BRFSS data)	**358,355 employed adults** (48% men) ? (18-55 years)	Industry & occupation (I&O) optional module BRFSS & **QFQs**	**BD**: men consuming ≥ 5 drinks or women consuming ≥ 4 or more drinks, on an occasion.	**No statistical analysis performed.** 20.8% reported BD, with an average of nearly 49 times per year and an average intensity of 7.4 drinks per binge episode, resulting in 478 total binge drinks per binge drinker. The adjusted BD prevalence ranged from 15.9% among community and social services workers to 26.3% among construction and extraction workers.

Abrevations: **AA:** alcohol abuse; **AD**: alcohol dependence; **ARH**: Alcohol Related Harm; **AUD:** Alcohol Use Disorder; **AUDADIS-IV**: Alcohol Abuse and lcoholism's Alcohol Use Disorder and Associated Disabilities Interview Schedule– DSM-IV Version; **AUDIT**: Alcohol Use Disorder Identification Test; **BD**: Binge Drinking; **BRFSS**: Behavioral Risk Factor Surveillance System; **CLHS**: Chicago Longitudinal Health Study; **C-SURF**: Cohort Study on Substance Use Risk Factors; **FFQ**: Food Frequency Questionnaire; **GLM**: General linear models; **GNP**: General Population; **GPRF**: General Practice Research Framework; **HAU:** Heavy alcohol use; **ICD-10/11:** International Classification of Diseases 10th/11th Revision; **MONICA**: MONItoring of trends and determinants of CArdiovascular disease; **NIAAA**: National Institute on Alcohol Abuse and Alcoholism; **NRR**: Non response rate; **wk**: week; **NSDUH**: National Survey on Drug Use and Health; **OHC**: Occupational Health Care clinic; **PHC:** Primary Health Care clinic outpatients; **QFQs**: Quantity Frequency Questionnaires of alcohol use; **RSOD**: Risky Single-Occasion Drinking; **SAMHSA**: Substance Abuse and Mental Health Services Administration; **USA:** United States of America; **yr.:** year; ?: mean age is not mentioned.

For peer review only

Supplementary File 4

Table 3: Prevalence, associated factors, and pattern of problem drinking in low-and middle-income countries (LMICs), 2023.

Author, Year<br><br>Country/Location	Study Design & Study Setting<br><br>(population)	Participants: Sample size (Male, %) Mean age (range) in years	Tools (Measures) or questions used	Outcomes: (Definition/nature of use)	Results & statistical methods used:
**Andersson et al., 2018**<br><br>**South Africa**<br>(Eastern Cape Province)	**Cross-sectional**<br>(Nelson Mandela Metropolitan & Sundays River Valley City)<br>**Population-based**<br>(Urban/semi-urban/ rural setting)	1000 participants (52% of men)<br>27 (18-40) years	**M.I.N.I. 6.0 (DSM-IV)**	**Alcohol dependence & Alcohol abuse (AD/AA):** (DSM-IV diagnosis during the past 12 months)	**χ^2 statistics & logistic regression models:**<br>AD: 26.5% (39.0% men & 19.1% women)<br>AA: 9% (19.0% for men & 6.0% for women).<br>**AD:** higher in rural/semi-rural in men (43.1%) and women (26.8%) than in urban/semi-urban.<br>Widowed and separated women compared to married or cohabiting and women with low income (don't want to disclose) compared to weekly household income of ≥ 1,001 RAND remained statistically significant.
**Burazeri and Kark, 2010**<br><br>**Albania**<br>(Tirana)	**Cross-sectional**<br>(transitional post-communist Albania (Muslim, 68.5%)<br>**Population-based**	685 individuals (65.7% of men)<br>52.6 (35–74) years	Quantity/ frequency questionnaires **(QFQ**s) (patterns questions) (12 months)	**Drunkenness/hangovers:** never, very exceptionally, 2-3 times/year, 1/month, 1/fortnight & once/week). Composite Binging score: drunkenness or hangovers during w/c ≥3 units (≈60g of ethanol) consumed	**Binary/multivariable logistic regression:**<br>10.3% of men had ≥ 2-3 annual episodes of drunkenness & and hangovers each.<br>**Women**: both markers of binging, 1.4%<br>**Men:** 8.9% drinking ≥ 60g alcohol/session.<br>Binge drinking was related to low educational level, financial loss in pyramid collapse, & religiosity (inversely) in both Muslims and Christians (all in men).
**Dias da Costa et al., 2004**<br><br>**Brazil**<br>(Rio Grande do Sul State)	**Cross-sectional**<br>(Adults of municipality of Pelotas)<br>**Population-based**<br>(Urban area)	2,177 adults (43%)<br>41.6 (20-69) years	**QFQs**<br>(weekly use)	**Moderate consumption:** up to 30g/day of ethanol)<br>Heavy consumption or hazardous drinking, HD: ≥ 30g/day of ethanol/week	**Non-conditional logistic regression:**<br>Moderate consumption was 65.1%<br>**HD:** 14.3% (29.2%, men & 3.7% in women).<br>Men, elders, blacks, low SES, heavy smokers, & chronic disease presented higher prevalence of HD. Men with minor psychiatric disorders had higher prevalence of HD & in women (association between age & HD was inversely related).

For peer review only - http://bmjopen.bmj.com/site/about/guidelines.xhtml

**Ji et al., 2018**<br><br>**China** (Xuzhou city, Jiangsu)	**Cross-sectional** (11 regions in Xuzhou city)<br>**Population-based** (urban/rural areas)	36,157 participants (48.40% of men)<br>45.5 (18-75) years	**MAST**	**Alcohol dependent (AD):** MAST score of ≥ 5<br>0 (no alcohol dependence)<br>1–4 (low AD), 5–6 (light AD), 7–25 (mild AD), 26–39 (moderate AD & 40–53 (severe AD)	**χ2 &, t-tests; multivariate log. Regression:**<br>**AD:** 11.56% (22%, males & 1.74%, females)<br>Newly detected hypertension rate was 9.46%<br>Significant associations were found between AD & blood pressure. AD was positively correlated with systolic blood pressure & diastolic blood pressure ($r = 0.077$, $P < 0.01$).
**Mendoza-Sassi and Beria, 2003**<br><br>**Brazil**	**Cross-sectional** (Residents in municipality of Rio Grande, Southern Brazil)<br>**Population-based** (Urban population)	1260 people (46.1% of men)<br>40.3 (15-94) years	**AUDIT**<br><br>**SRQ-20**	**Alcohol Use Disorder (AUD):** AUDIT score ≥ 8	**Log. regression in multivariate analysis:**<br>**AUDs:**7.9% (2.5%, women & 14.5%, men).<br>Risk of alcohol misuse increased across increasing social class (P linear trend = 0.03)<br>Males had OR=6.89 compared with women.<br>Smokers (OR 3.27) & ex-smokers (OR 1.30) were at higher risk than non-smokers.<br>Those with minor psychiatric disorders had a 2.48 OR of presenting a positive test (AUD).
**Moreira et al., 1996**<br><br>**Brazil**<br>(Porto Alegre)	**Cross-sectional** (Adult population of Porto Alegre, Southern Brazil)<br>**Population-based** (Urban)	1,091 individuals (45.0% of men)<br>Mean age: 41/men; 44/women<br>(≥ 18) years	**CAGE** & Type & **QFQs** of alcoholic drink	**Heavy drinking (HD):** Average of ≥ 30g/day<br>**Alcohol dependence/AD:** Two positive answers to the CAGE questionnaire	**X^2-test & logistic regression models:**<br>**AD** was 9.3%; **heavy drinking** was 15.5%.<br>Increasing age, lower education & income, non-white race (associated with HD & AD).<br>Households with 3-4 persons were associated with lowest risk HD, but AD was higher in crowded households (5-11). Presence of one with HD/AD in household was associated with HD but not with AD.
**Peltzer et al., 2011**<br><br>**South Africa**	**Cross-sectional** (Part of SABSSM 2008 survey)<br>(62.5% located in urban areas)	13,828 persons (43.7% of men)<br>? (≥ 15 years)	**AUDIT**	**Binge drinking (BD):** Females (4) & males (5) standard drinks/occasion<br>**Hazardous or harmful drinking:** AUDIT cut-off score ≥ 8	**Adjusted logistic regression:**<br>**Risky (hazardous/harmful drinking):** 9% (17% among men & 2.9% for women)<br>Overall prevalence of BD: 9.6%<br>**Men:** risky drinking was associated with 20-54 years than 15-19; Colored population group; lower (economic status & education.)<br>**Women:** risky drinking was associated with urban residence, Colored population group; lower education; and higher income
**Peltzer and Phaswana-Mafuya, 2013**<br><br>**South Africa**	**Cross-sectional** (older South Africans, Study of Global Ageing &	2144 participants (41.1% of men)<br>? (> 60 years old)	**QFQs** & **NIAAA** risky drinking criteria	**Risky drinking (2 ways):**<br>**Heavy drinkers:** (>7 drinks per week) &<br>**Binge drinkers:**	**Multivariate logistic regression:**<br>**Heavy & binge drinking**: 4% vs 3.7%<br>Male gender, white population group; tobacco use & being obese were associated with risky drinking.

For peer review only

	Adults Health, SAGE in 2008) **Population-based** (Urban, 63.2%)			(>3 drinks/one occasion at least weekly)	Hypertension, diabetes, and depression were not associated
**Peltzer et al., 2012** **South Africa**	**Cross-sectional** (South African Youths, Black, 97.5%; 4 of 9 provinces in SA) **Population-based**	3123 participants (54.6% of men) 20.5 (18-24) years	**AUDIT-C** (Frequency of drinking, quantity consumed per occasion & frequency of HED)	**HED:** consumption of five standard drinks (≥ 60g) alcohol per single occasion **Binge drinking:** women (4) & men (5) units in a session at least/month **Hazardous or harmful drinking (HHD):** ≥ 5 on AUDIT-C	**Unconditional multivariable log. Reg.:** **HHD:** 19.1% (24.3%, male; 12.9%, women) **Men:** high sexually permissive attitudes, not poor, multiple sexual partners, tobacco & illicit drug use were associated with HHD. **Women:** high (HIV risk perception, sexually permissive attitudes & peer pressure (lifestyle), spending more nights away in a week, tobacco & illicit drug use were associated with HHD.
**Tomkins et al., 2007** **Russia** (Izhevsk)	**Cross-sectional** (Men controls in a case-control study of premature male mortality, Izhevsk) **Population-based** (Urban)	1750 men (100% men) ? (25-54 years)	**QFQs**	**Hazardous drinking-HD:** (any of these in past year) Having drunk surrogates; having been on zapoi; having frequent hangovers (once/month or more); having drunk spirits daily.	**Logistic regression:** Drinking spirits (79%) & surrogates (8%) at least sometimes in the past year. Drinking spirits (25%) & surrogates (4%) at least weekly & 10% had had episode of zapoi in past year. Education, lowest level in men (associated with indicators of HD. Indicators HD were also associated with being unemployed & levels of household wealth/amenities.
**Weiser et al., 2006** **Botswana**	**Cross-sectional** (5 districts of Botswana with highest number of HIV-infected individuals) **Population-based** (Urban/Rural)	1,268 adults (48% men) 28.8 (18-49 years)	**QFQs**	**Heavy alcohol consumption (HD):** > 14 drinks/wk for women, & > 21 drinks/wk for men) **Problem drinking** (8–14, women, 15–21 for men) &	**Heavy drinking:** 31%, men & 17%, women **Problem drinking:** 39% of men, (79% met HD) & 25 % of women, (69% met HD). Correlates of HD: intergenerational relationships (age gap 10 year), male gender, higher education, & living with a sexual partner. A dose-response relationship was seen between alcohol use & risky sexual behaviors, with moderate drinkers at lower risk than both problem & heavy drinkers.
**Zavos et al., 2015** **Sri Lanka** (Colombo district)	**Cross-sectional** (Data from the Colombo Twin And Singleton Study, CoTASS) **Population-based** (Urban/semi-urban areas)	6014 Sample (twins/48% & Singleton/46% of male) Mean age: 34 (twins) & 43 (singleton) (> 16 years)	**CIDI** Alcohol use: ever had of 12 drinks at any time in life	**Alcohol abuse & dependence:** Definition of CIDI (DSM-IV criteria)	**Robust cluster command:** **12-month prevalence of alcohol use:** 22.7% **Lifetime AA & AD in men:** 6.2% & 4.0% Lifetime AA & AD was associated with greater prevalence of nicotine dependence, depression, anxiety & PTSD (only for AD). Lower standard of living was associated with alcohol use & AD but not with AA

For peer review only - http://bmjopen.bmj.com/site/about/guidelines.xhtml

**Lo et al., 2013**<br><br>**Kenya**<br>(Nyanza Province)	**Prospective study**<br>(Longitudinal database of demographic & health census data in western Kenya)<br>**Population-based**<br>(Rural area)<br>Secondary data	72,292 individuals<br>(43.1% men)<br>? (≥ 18 years)	Questions on (ever use & current use)	**1) % of time drunk when drinking in past 30 days:** (Did not get drunk, Drunk < 50%, Drunk 50%+)<br>**2) Days drinking/month:** (1-7, 8-17 & 18+)<br>**3) Problem drinking:** drinking ≥ 8 days/past 30 days & were drunk at least 50% of times they drank	**Crude and adjusted logistic regression:**<br>Overall, ever drinking was 20.7%<br>Drinking/past 30 days was 7.3% & 34.6%.<br>(60.3%, being drunk on ≥ 50%+) of all drinking occasions)<br>Alcohol use increased with decreasing socio-economic status & oldest women.<br>Current smoking, men, all age groups ≥ 40 & highest wealth index quintile (significantly associated with problem drinking).
**Pillai et al., 2013**<br><br>**India**<br>(Northern Goa)	**Cross-sectional**<br><br>**Population-based survey**<br>(rural & urban communities)	2641 men<br>(100% men)<br>? (18-49 years)	**QFQs** &<br>Drunkenness	**Current drinkers:**<br>**low risk** (< 40 g/d),<br>**medium risk** (40–60 g/d),<br>& high risk (> 60 g/d)<br>**HED**: ≥ 60g in a single occasion in past 12 months<br>**Drunkenness**: times drank to feel drunk in last 1 year (< monthly, ≥ monthly but < weekly), & ≥ weekly)	**Logistic regression + Moderating effect:**<br>Of current drinkers:<br>**HED:** 28.6 % (rural 31 %; urban 27.2 %) & Drunkenness: 33.7% (rural 30.5 %; urban 35.5 %) ➔ monthly or more frequent<br>**HED**: associated with older age, being separated, lower education, & LSI<br>Weekly or more frequent drunkenness was associated only with rural residence.<br>All three risky drinking patterns were associated with CMDs, sexual risk, intimate partner violence, acute alcohol-related consequences, & AD.
**Sau, 2017**<br><br>**India**<br>(West Bengal)	**Cross-sectional**<br>(Adult population of the state of West Bengal, Gram Panchayat, GP)<br>**Community-based**	99 adults<br>(54.5% men)<br>38.62 (≥ 18) years	**AUDIT**	**AUDIT (WHO scoring):**<br>≥ 8 (hazardous/harmful use & possible AD)<br>0-7 (Zone-I): Low risk drinking/abstinence risk<br>8-15 (Zone-II): Alcohol use in excess of low-risk,<br>16-19 (Zone-III): Harmful & hazardous drinking &<br>20-40 (Zone-IV): Alcohol dependence risk level.	**Intraclass correlation, chi-square test, logistic regression & Bootstrapping:**<br>**Mean AUDIT** score was 7.11 (5.55 to 8.74)<br>**Low risk drinking/abstinence:** 65.5% & **Alcohol use in excess of low risk:** 17.6%, & **Harmful & hazardous drinking:** 8.5% & **Alcohol dependence** was 8.4%<br>**Hazardous, harmful use & AD** was 34.5%<br>Male gender and being employed were more prone to become high risk level drinker.
**Takahashi et al., 2017**<br><br>**Kenya**<br>(Western)	**Cross-sectional**<br>(Adults residing in Ikolomani Sub-county, Kakamega)<br>**Community-based**	478 participants<br>(41.4% men)<br>41 (18–65) years	**AUDIT**<br><br>**Type & QFQs**	**Current drinkers:** use of any alcohol in the last month,<br>Hazardous/high-risk drinkers:	**Univariate & multivariate analyses:**<br>Current & hazardous/high-risk alcohol use: 31.7% (men 54.6%; 8.9%, women) vs 28.7%<br>More than one drinker in the family, ≥ 5 drinker friends & positive attitude towards alcohol intake were positively associated with

For peer review only - http://bmjopen.bmj.com/site/about/guidelines.xhtml

	(Rural)			AUDIT score of ≥ 8	current alcohol drinking status, and with hazardous/high-risk alcohol consumption.<br>Women were less likely to be current drinkers & hazardous/high-risk drinkers.
**Yeung et al., 2015**<br><br>**Cambodia**<br>(Puok district)	**Mixed methods**<br>(Adults living in 2 selected rural communities<br>**Community-based**<br>Rural communities	120 households<br>(49.0% men)<br>? (≥ 18 years)	**AUDIT-C-Q**<br>**QFQs**<br>8 FGDs<br>**NIAAA**<br>Guidelines	**AUD:** cut off score of ≥ 5 in men & ≥ 4 in women<br>HED: ≥ 6 drinks in a single sitting at least monthly (NIAAA)	**χ2, Welch 2-sample t-test, Log. Regression**<br>**AUD & HED:** 4% and 31%, respectively.<br>**AUD** (47% men, 5% women (P < 0.0001); **HED** (47% men, 15% women (P = 0 .0001).<br>Male sex, younger age (decreasing age), and increasing income (higher monthly) were significant risk factors for AUD and HED
**Alem et al., 1999**<br><br>**Ethiopia**<br>(Butajira)	**Cross-sectional**<br>(Demographic surveillance site)<br>**Community-based**<br>(mostly rural)	12531 residents<br>(50% male)<br>? (≥ 15 years)	5-item questionnaire (questions for alcohol user vs non-users &<br>**GAGE-4** items)	**Problem drinking (PD):** consumption beyond safe limits (≥ 2 positive responses on CAGE).<br>Cigarettes smoked daily: 1-3=mild,<br>4-9=moderate,<br>>9= heavy	**Chi-square statistics:**<br>Current drinkers: 23.4 % (15% women & 36% for men).<br>PD, 15.7% in alcohol users; overall PD, 3.7% (7.5% men & 0.90% women).<br>(2.4% in urban dwellers & 4.0% in rural)<br>Christian religion, male sex, ethnically non-Gurage, & smoking (associated with PD in both sexes). Marital status (divorced men), mental distress & income were associated with PD only in men & being widowed & divorced in women
**Kebede and Alem, 1999**<br><br>**Ethiopia**<br>(Addis Ababa)	**Cross-sectional**<br>Adults in Addis Ababa<br>**Population-based**<br>(Urban residents)	10203 adults<br>(45.1% men)<br>? (≥ 15 years)	**CAGE**<br>(1st stage) &<br><br>**CIDI**<br>(2nd stage)	**Problem drinking (PD):** ≥ 2 of on CAGE items, &<br>**Alcohol dependence (AD):** CIDI (ICD-10 diagnoses)	**Bivariate and multivariate analysis:**<br>**PD** was 2.7%, lifetime AD, 1.0% (1.9% in male & 0.1% for women) & one-month AD, 0.8% (1.5% for men and 0.06% for women).<br>PD increased with increasing age<br>PD decreased with increasing educational attainment. 39% increased risk of PD with employment & female sex had a 96% decreased risk of PD. Only sex (women had an 84% less risk to be AD compared to men).
**Nalwadda et al., 2018**<br><br>**Uganda**<br>(Kamuli District)	**Cross-sectional**<br>(Men attending PHC & men in population; part of the PRIME project)<br>**Community-based** & facility-based<br>(Rural district)	351 men<br>(Community study)<br>778 men<br>(Facility Survey)<br>(100% men)<br>? (≥ 18 years)	**AUDIT**<br>(10 item)	**AUD definition (AUDIT):**<br>**Hazardous** (score 8–15),<br>**Harmful** (score 16–19) or<br>**Dependent** (score ≥ 20) drinking behaviors<br>(cut-offs defined by WHO)	**Kruskal–Wallis test & Fisher's exact test:**<br>**Community study**: 4.1% of all men were AUDIT+ (AUD); (2.9% hazardous, 0.7% harmful & 0.5% with dependent drinking)<br>**Facility study:** 5.7% of all men were AUDIT+; (4.5% hazardous; 0.6%, harmful)<br>47.5% AUDIT+ men: AUD ruined their lives<br>55.0% AUDIT+ men did not seek treatment<br>AUDIT scores were higher among older men, men with paid/self-employment status and higher PHQ-9 score (P < 0.05).

For peer review only - http://bmjopen.bmj.com/site/about/guidelines.xhtml

**Rathod et al., 2018**<br><br>**Nepal**<br>(Central district)	**Cross-sectional**<br>(Adults in Chitwan District; part of PRIME consortium)<br>**Population-based**<br>Secondary analysis	3482 sample<br>(36% men)<br>? (18-88) years	**AUDIT**<br>(10-item)	**Abstinent:** Score of 0,<br>**Recent (12 months) consumer:** Score of ≥ 1<br>Score of ≥ 8: **positive screen for AUD,**<br>8–15: **hazardous** drinking,<br>16-19: **harmful** drinking &<br>≥ 20: **dependent drinking**	**X^2 test & Negative binomial regression:**<br>23.8% of male screened AUD+ (AUD)<br>5.3% of female drinkers screened AUD+<br>Men with AUD, 38% spoke to another person about their problems & 80% had internalized stigma.<br>Being a drinker was associated with age, religion, caste, education, occupation & tobacco use. AUDIT scores were associated with age, caste, marital status, occupation, tobacco use, depression, functional status & suicidal ideation.
**Teferra et al., 2016**<br><br>**Ethiopia**<br>(Sodo district, southern Ethiopia)	**Cross-sectional**<br>(Adults from rural Sodo district (PRIME survey)<br>**Community-based**<br>(Rural residents)	1500 adults<br>(50.5% men)<br>? (≥ 18 years)	**FAST**<br>**Kessler-10** (psychological distress)<br>**LTE** (adverse life events)	**Hazardous alcohol use (HD):**<br>FAST score ≥ 3 out of 16	**Exploratory multivariable log. regression:**<br>Prevalence of **hazardous alcohol use**: 21%; (31% in males & 10.4 % in females)<br>Factors associated with HD were being male, increasing age, having experienced ≥ 1 stressful/adverse life events, & severe psychological distress (AOR = 2.96).<br>High social support was protective from hazardous alcohol use (AOR = 0.41)
**Zewdu et al., 2019**<br><br>**Ethiopia**<br>(South, Sodo district)	**Cross-sectional**<br>(Adults who lived for at least 6 months in Sodo dist)<br>**Community-based**<br>(Rural district)	1485 individuals<br>(45.7% men)<br>39 (≥ 18) years	**AUDIT-10**	**Probable AUD:** score ≥ 8<br>8–15 (medium level of alcohol problem)<br>≥ 16 (high level of alcohol problems)<br>≥ 20 (possible alcohol dependence-AD)<br>**Binge drinking (BD):** drinking ≥ 6 alcoholic drinks on a single occasion	**Poisson regression with robust variance:**<br>Weighted prevalence of AUD was 13.9%; 25.8% in men & 2.4% women, P < 0.001<br>(Hazardous/harmful/AD: 9.9%/2.2%/1.8%)<br>23.3% had BD<br>87.0% of cases scored ≥ 16 had never sought help & 70.0% had high internalized stigma<br>AUD were associated & more prevalent in men (aPR = 7.7), farmers, traders, & daily laborers. People with AUD had increased total depressive symptom score & higher total disability score, more stressful life events & suicidal ideation (aPR 1.5)
**Getachew et al., 2017**<br><br>**Ethiopia**	**Cross-sectional**<br>(2015 national noncommunicable diseases STEPS survey)<br>**Community-based**<br>(Urban,27.4% & rural, 72.58%)	9,800 participants<br>(40.6% men)<br>34.5 (15-69) years	**QFQs**<br>(WHO STEPS questionnaire)	**Current drinkers**: alcohol use a month before survey<br>Lifetime alcohol use: ever<br>**Past 12-month users:**<br>HED/Excessive Alcohol Consumption: drinking ≥ 6 drinks in men & ≥ 4 in women on one occasion.	**Logistic regression:**<br>Prevalence of lifetime alcohol consumption & current drinkers was 49.3% & 40.7%.<br>Among ever drinkers, 89.6% drank alcohol in the past 12-months.<br>**HED:** 12.4% (20.5% males & 2.7% females)<br>Factors independently associated with HED, were male sex, rural residence), married, and current tobacco smoking (AOR=2.87).
**Abd Rashid et al.,**	**Cross-sectional**	**363 participants**	**AUDIT**	**Hazardous alcohol use:**	**Multiple logistic regression analysis**

For peer review only - http://bmjopen.bmj.com/site/about/guidelines.xhtml

**2021**<br><br>**Malaysia** (Sabah Borneo Island)	(People in Bingkor who consumed alcohol in the past 12 months)<br>(urban setting)	(51.5% men)<br>? (≥ 26 years old, 90.6%)	(hazardous alcohol use)<br>**MINI V5.0** based on DSM-IV (psychiatric morbidity)	AUDIT scores of ≥8	80.2% admitted having consumed alcohol.<br>Preferred type of drink: beer (67.8%), tuak tapai (61.7%), wine (31.7%), tuak beras and whisky (16.8%), imported alcohol drinks such as vodka (9.1%) and 'samsu' (3.9%).<br>41% of participants (high risk for hazardous alcohol use) vs 39.1% (with low risk of hazardous alcohol use).<br>Being male & being a non- Muslim had a higher risk to develop hazardous alcohol use (OR = 3.313 & 3.834 respectively).<br>Having a current obsessive- compulsive disorder was associated with a higher risk of hazardous alcohol use (OR = 0.265).
**Assanangkornchai et al., 2020**<br><br>**Thailand**	**Cross-sectional**<br><br>(Thailand's 5th National Health Examination Survey, NHES-5, 2014)<br>(urban/53.6%, rural/46.4%)	**13177 participants**<br>(49.2% men)<br>46.7 (> 20 years)	**AUDIT**<br>(for AUD)<br>**MINI, Thai version 5.0.0**<br>(for MDE)	**AUD**: non-problem drinkers (0–7), hazardous drinkers (8–15), and harmful-dependent drinkers (16– 40) on AUDIT<br>**MDE**: defined according to DSM-IV criteria	**Multinomial logistic regression:**<br>10.3% and 1.9% hazardous drinkers and harmful-dependent drinkers, respectively<br>2.5% met the criteria for MDE in the past 12 months before the survey.<br>Approximately 20% were current smokers.<br>Associations between MDE and either hazardous (HD) or harmful dependent drinking (HDD) were strongest among those in third tercile (highest/wealthiest) of wealth index, first tercile (lowest/poorst), secondary school level of education or above, living in urban areas, & those who are employed.
**Ding et al., 2020**<br><br>**China**	**Cross-sectional**<br>(China Health and Retirement Longitudinal Study, 2011– 2012)<br>**Community based**<br>(Urban/40.5%, Rural/59.5%)	**17,302 subjects**<br>(49.30% men)<br>59.67 (aged ≥ 45 years)	**QFQ**<br>(for alcohol use)	**Heavy drinking:** >14 drinks per week (males) & >7 drinks per week for females	**Binary & multinomial logistic regressions**<br>Overall prevalence of heavy drinking, obesity, current smoking, and physical inactivity were 7.23%, 11.53%, 27.46%, and 44.06%, respectively.<br>Compared with healthy subjects (no hypertension, high cholesterol, or diabetes), newly detected hypertensive patients were more likely to smoke (OR, 1.34), be heavy drinkers (1.45), and be obese (1.94).
**Hernandez-Vasquez et al., 2022**<br><br>**Peru**	**Cross-sectional**<br>[(2018 Peruvian Demographic & Family Health Survey (ENDES)]<br>**A Population-Based Analysis**	**32,020 people (analysis)**<br>(42.8% men)<br>? (≥ 18 years old)	**SAMHSA definition (RSOD):** Bing Drinking (BD)	**BD:** consumption of 5 & 4 or more alcoholic beverages on the same occasion for men & and women, respectively, in the last 30 days before the survey	**Poisson's family GLMs with link function (log) were used for (cPR and aPR).**<br>BD was found in 22.4%. Men (32.6%) presented a higher consumption pattern than women (12.8%).<br>Men aged 25–44 had a higher probability of BD (aPR: 1.28). The age group of ≥ 60 was associated with a lower probability (aPR: 0.70) of BD compared to younger group of men (18-24 years).

For peer review only

For peer review only - http://bmjopen.bmj.com/site/about/guidelines.xhtml

	(Urban/65.7%, Rural/34.5%)				Women aged ≥ 60 years was associated with a lower probability of BD (aPR: 0.24). Secondary (aPR: 2.01) or higher level of education (aPR: 2.04) was a factor associated with a higher prevalence of BD in men
**Jadnanansing et al., 2021**<br><br>**Suriname**	**Cross-sectional** [(populations in both region (rural/Nickerie & urban/Paramaribo)]	**2863 participants** (43% men)<br>39.97 years (?)	**AUDIT & ASSIST:** (for AUD)	**Risky alcohol use:** A score of > 7 on AUDIT	**Simple & Multivariable logistic regression**<br>AUD is 6.4% in urban area & 5.8% in rural area. Men had highest addiction risk at about 16% compared with 2% for females.<br>A treatment gap of 50% was found for AUDs in the rural area (64% urban area).<br>Married persons are significantly less likely to become alcoholic than singles and other groups in urban area.<br>In both areas, higher education was associated with a lower probability of alcohol abuse and dependence, while handymen showed a higher odd.
**Jirapramukpitak et al., 2008**<br><br>**Thailand** (Bangkok)	**Cross-sectional** (Suburban community of Bangkok in 2003 and 2004)	**1052 residents** (46.3%)<br>? (16–25 years	**AUDIT** (hazardou or harmful drinking) & **DIS** (illicit drug use-Diagnostic Interview Schedule)	**Illicit drug use:** assessed with self-report adapted from (DIS) and **Hazardous/harmful drinking:** with AUDIT **Migration**: the occasion when a young person born in amore rural area moves for the first time into Greater Bangkok.	**Multivariate analysis (logistic regression)**<br>10.9% (82 males and 17 females) had illicit drug use and 24.3% (179 males and 62 females) hazardous and harmful drinking.<br>Hazardous/harmful drinking was associated independently with being late migrants, who moved at the age of 15 or older.
**Moreira et al., 1998**<br><br>**Brazil**	**Cross-sectional** (Adults in Porto Alegre, a city in southern Brazil)	**1099 individuals** (45% men)<br>? (18-88 years old)	**QFQs** (type, quantity, & frequency) **& CAGE questionnaire**	**Heavy drinking**: average consumption of 30g/day or more, a level of exposure associated with health risks **Dependence:** Two positive answers to the CAGE questionnaire	**Simple/multiple linear & logstic regression**<br>24.1% had never drunk alcohol (9.0%/men & 36.5%/women). 29.3% of men & 4.2% of women were heavy drinkers. 16% & 4.0% were CAGE+, respectively.<br>Consumption of 30 g/day ethanol was associated with increases of 1.5 & 2.3 mmHg in DBP & SBP for men and 2.1 and 3.2 mmHg for women respectively.<br>Prevalence of HTN was higher among those ingesting ≥ 30 g/day (odds ratio = 2.9).
**Oancea et al., 2021**<br><br>**Brazil**	**Cross-sectional** (2013 Brazilian National Health Survey)	**59,399 individuals** (47.6% men) weighted median age, 40.53 (18-60+	**NIAAA definitions** (Binge drinking/BD &	**BD (NIAAA)**: a pattern of drinking that brings BAC levels to at least 0.08 g/dl. (4 drinks for women & 5	**Weighted & adjusted logisitic reggression**<br>14.8% were current smokers, 13.8% were binge drinkers & 3.2% were heavy drinkers.<br>Self-reported current depression/SRCD,7.6%

For peer review only - http://bmjopen.bmj.com/site/about/guidelines.xhtml

		years)	Heavy drinking/HD)	for men in about 2hrs)<br>**HD**: ≥ 5 days of BD episodes in a month is defined as the HD index.	There was significant weighted & adjusted increase in the odds of SRCD among young adults (18–39 years) who were binge drinkers compared to those who were not binge drinkers (AOR = 1.32).
**Pengpid et al., 2021**<br><br>**South Africa**	**Cross-sectional**<br>(National survey of all household members, who resided in that household the previous night)<br>(Rural informal/ 26%, Rural farms/ 5%, Urban/69%)	**39,210 persons**<br>(48.3% men)<br>Median age,34 (IQR,25-48)<br>(15 years & older)	**AUDIT** (Hazardous, harmful, or dependent alcohol use (HHDA):<br>**ASSIST** (Drug use in the past 3 months)<br>**K10** (Kessler Psychological Distress Scale)	**HHDA:**<br>Adults (≥ 20 yrs): cut-off score is ≥ 8 on AUDIT & Adolescents (15–19 years): 5 or more on AUDIT<br>**Drug use in past 3 months**: Any drug used in past 3 months was coded as 1 and never as 0'.<br>**Psychological distress:** scores ≥ 20 on (K10)	**Unadjusted & adjusted logisric regression**<br>10.3% engaged in HHDA, 16.5% (males) & 4.6% (females). Past 3-month drug use was 8.6%, 13.3% (males) & 4.1% (females). Men of middle age (25-34) with higher education, urban residence, drug use and psychological distress were positively associated with HHDA. Women of middle age (25-34) and mixed race, residing on rural farms and urban areas, drug use and psychological distress were positively associated & older age (≥55) & Indians or Asians were negatively associated with HHDA.
**Prais et al., 2008**<br><br>**Brazil**<br>(Metropolitan area of Belo Horizonte, & Bambuí)	**Cross-sectional**<br>(elderly Brazilian men, ≥ 60 years were the study population)<br>**Population based**<br>(urbann setting)	**685 residents in RMBH & 642 in Bambuí**<br>(100% men)<br>**Mean age:**<br>68.8 yrs (RMBH)<br>69.0 yrs (Bambuí)<br>(≥ 60 years)	**RSOD criteria**<br>(for BD)	**Binge Drinking:**<br>Consumption of five or more alcoholic drinks on a single occasion in the last 30 days.	**Multivariate analyses (PR estimated by Robust Poisson Regression)**<br>Prevalence of BD was two times higher among residents in metropolitan area of Belo Horizonte (27.1%) than in Bambuí (13.7%).<br>**RMBH:** higher schooling level [8+ yrs] (PR = 1.55), worse self-rated health [reasonable, bad, or very bad] (PR = 0.62) and inability to perform activities of daily living (PR = 0.12) remained significantly associated with BD.<br>**Bambuí**: worse self-rated health (PR = 0.57) and being divorced or separated (PR = 2.49) remained significantly associated with BD.
**Trangenstein et al., 2018**<br><br>**South Africa**<br>(Tshwane Metropole)	**Cross-sectional**<br>(Adults who used alcohol in the past six months).<br>(Data from South African arm of the multi-country International Alcohol Control, IAC study)	**713 adults**<br>(65.8% men)<br>36.3 (18-65 years)	**International Alcohol Control (IAC) questionnair:**<br>(Asks QFQs over past six months)	**Heavy Drinking (HD):**<br>consuming ≥ 96g of absolute alcohol (AA) (roughly 8 standard drinks, or 120 ml) for men or ≥ 72g (6 standard drinks, or 90 ml) for women at least monthly.<br>**Low risk**: occasions that did not include HD	**Multivariate logistic regression**<br>HD was 53%. HD did not vary by gender (F1, 19 = 3.96, p = 0.06), age, race/ethnicity, or total annual personal income. Bivariate analyses revealed that HD differed by marital status (F2.48, 47.11 =3.09, p = 0.04).<br>Adjusting for marital status & primary container size, single persons were found to have substantially higher odds of HD.

For peer review only - http://bmjopen.bmj.com/site/about/guidelines.xhtml

	(urban setting)				
**Vellios and Van Walbeek, 2018**<br><br>**South Africa**	**Cross-sectional** (data from wave 4 of the 2014-2015 National Income Dynamics Study, NIDS) (rural/35.4%, urban/64.6%)	**22,752 (wave 4)** (46.8% men) ? (≥ 15 years)	**QFQs:**1) How often do you drink alcohol? 2) On a day you have an alcoholic drink, how many standard drinks do you usually have?	**Binge drinker:** use of ≥5 standard drinks on an average drinking day. **Current drinker**: any option from (iii) I drink alcohol very rarely, (iv) Less than once a week, (v) On 1 or 2 days a week, (vi) On 3 or 4 days a week, (vii) On 5 or 6 days a week, & (viii) Every day.	**Multiple logit regressions**<br>Current alcohol use (any amount) in 2014 - 2015 was reported by 33.1% of the population (47.7% males, 20.2% females). Of current drinkers, 43.0% reported BD (48.2% males, 32.4% females).<br>Self-reported BD as a proportion of the total population was 14.1% (22.8% M, 6.4% F).<br>Self-reported BD was highest among males & females aged 25-34 years (49.4%).<br>Smoking cigarettes for both genders substantially increased the likelihood of drinking any amount (aOR: 5.08 males, 4.80 females) and of BD (aOR: 1.53 for males, 3.36 for females).<br>As a percentage of total population, people aged 25-34 years were more likely to binge than aged 15-24 years, for both males (OR 1.44) and females (OR 1.49). Compared with married males, males living with a partner (OR 1.58) or who were single (OR 1.74) were more likely to BD.<br>Compared with married females, females living with a partner (OR 1.68) or single (OR 1.41) were more likely to BD.<br>Having children in the house slightly increased the probability of BD for males (OR 1.21), but not for females.
**Aremu et al., 2021**<br><br>**Nigeria** (urban poor people in Ibadan)	**Cross-sectional** (two selected urban poor communities in Ibadan, Nigeria)	**500 Participants** (29.4% men) 35.36 (18-65 years)	**Modified version of WHO STEPS instrument**	**Alcohol consumers:** Ever consumed, Current consumers (12mo.) Current & **frequent consumers within 30 days (low, medium, and high)** **Low consumers:** consuming < 4 (men) & < 2 (women) SDs/occasion **Medium:** 4-6 (men) & 2-4 (women) SDs per occasion **High:** > 6 (men) & > 4 (women) SDs per occasion	**Descriptive & inferential statistics (X^2)**<br>29.0% had consumed alcohol either in past or present, 17.8% consumed alcohol within last one year, 15.8% were current consumer of alcohol & 13.6% were frequent consumers who had taken alcohol within 30 days (11.6% low consumers, 1.2% medium consumers and 0.8% high consumers).<br>More male (53.1%) reported to have ever consumed alcohol compared to female (46.9%). 62.3% of non-current alcohol users was female & 37.7% were male. 59.3% of respondents not currently consuming alcohol were currently married (30.3% were not).<br>74.1% of the low consumers were male, 66.7% medium consumers were females, & 75.0% of high alcohol consumers were male
**Bonnechère et al., 2022**	**Cross- sectional** (Data from the	**4692 individuals** (45.7% men)	Quantity/Frequency	**4 levels of consumption:** No consumption (None)	**Multinomial logistic regression:**<br>3559 (75.8%) were not consuming any alcohol, 12.9% had low,

For peer review only - http://bmjopen.bmj.com/site/about/guidelines.xhtml

**Burkina Faso**	2013 Burkina Faso WHO STEPwise)<br>Rural (75.1%), Urban (24.9%)<br>**Population- based**	? (25–64 years)	Questions **(QFQs)**	**Low**: intake of pure alcohol of <40g/day (men) & <20g for women<br>**Mid**: 40-59.9g/day (men) & 20-39.9g for women<br>**Abusive consumption:** ≥60g/day (M) & ≥40g (W)<br>**Dependent variable:** mean alcohol consumption in the last 30 days.	8.5% had mid and 2.7% had abusive alcohol consumption.<br>Age was associated with any level of alcohol consumption with a gradient effect and older people having a higher level of consumption in comparison with no consumption.<br>Tobacco consumption was significantly associated with alcohol intake with gradient effect, those with higher tobacco use being at higher risk of abusive alcohol intake.<br>Sex is an important risk factor for abusive consumption with increased risk for men compared with women. Jobless people & housemaker was associated with a decreased risk of having abusive consumption.
**Dahal et al., 2021**<br><br>**Nepal** (Kathmandu district)	**Cross-sectional** (adults residing in municipalities of Kathmandu district for at least six months)<br>**Community-based** (unplanned urbanization)	**245 participants** (47.3% men)<br>**Mean age:** 41.19/male, & 40.91/female (18–69 years)	**WHO STEPS questionnaire (QFQs)**	**Current episodic heavy drinking (HED):** six or more drinks on any day in the past 30 days.	**Bivariate & multivariate analysis**<br>67.3% were lifetime abstainers.<br>Prevalence of alcohol consumption in last 12 months was 31.0% & HED was 12.7%.<br>Prevalence of current smoking, low intake of fruits & vegetables and low physical activity was found to be 22%, 93.9% and 10.2% respectively. 52.2% of participants were overweight/obese & prevalence of raised BP was 27.8%.<br>Odds of alcohol consumption were higher among male (AOR: 2.78), employed (AOR: 2.30), & those who belonged to Chhetri (AOR: 2.83), Janajati (AOR: 6.18), Dalit and Madhesi, (AOR: 7.51) ethnic groups.
**Jonas et al., 2014**<br><br>**India** (rural Central India)	**Cross-sectional** (data from Central India Eye and Medical Study, CIEMS, in rural region of Central Maharashtra)<br>**Population-based**	**4711(participated)** (46.5% men)<br>49.5 (30+ years)	AUDIT<br>CESD 20-item<br>FTND (smoking behavior)	**Harmful or hazardous drinking:** sum score of 8 or more on AUDIT<br>**Clinical episode of major depression:** score of > 21 in the CES-D.	**Test of for association not performed**<br>Alcohol consumption was 23.0%; 6.0% subjects had an AUDIT score ≥8 (hazardous drinking), & 4.63% subjects a score ≥ 13 (women) or ≥ 15 (men) (alcohol dependence)
**Olickal et al., 2021**<br><br>**India** (Puducherry, South	**Cross-sectional** (adult men aged above 18 years in Puducherry, South	**316 adult men** (100% men)<br>45.2 (≥18 years)	**WHO AUDIT WHO QoL-BREF questionnaire**	**Hazardous alcohol**: AUDIT score of 8–15<br>**Harmful alcohol use**: AUDIT score of 16–19	**Independent t-test, One-way ANOVA & Kruskal Wallis test, Multiple linear regression**<br>Mean (SD) AUDIT score was 13.2 (6.7).<br>Probable dependence was 8.2%, & hazardous or harmful use was

For peer review only - http://bmjopen.bmj.com/site/about/guidelines.xhtml

India)	India)<br>**Community-based**<br>(rural/50%, urban/50%)			**Probable alcohol dependence**: score of 20 or more on AUDIT<br>**High risk:** A score eight and above on AUDIT<br>**QoL:** A higher score is indicative of a better QoL in each of the domains.	27.8%. Overall mean score of QoL was lower among alcohol users compared to non-alcohol users (50.7 vs 63.5)<br>QoL score was significantly lower among alcohol users (also in all domains).<br>High-risk alcohol users and urban residence had 11.2 & 4.1 less QoL scores respectively and educated had 7 more QoL scores compared to the reference category.
**Olickal et al., 2022**<br><br>**India**<br>(Puducherry, South India)	**Cross-sectional & Qualitative design**<br>(Mixed design)<br><br>(All men ≥ 18 years from urban & rural field practice areas of a tertiary care centre in Puducherry, South India)	**316 subjects**<br>(100% men)<br>45.2 (19-60+ years)	**WHO AUDIT**<br>Discussion guide for FGD	**Probable alcohol dependence:** A total score of ≥20 on AUDIT	**A log binomial regression (prevalence ratio) & Manual content analysis**<br>Alcohol use was 38%, 40% were daily users)<br>(34% in rural to 42% in urban areas)<br>Among alcohol users, 21.7% were probable dependents on alcohol.<br>Older individuals had a 2.9 times higher risk of alcohol use than young individuals (<30).<br>No formal education was a high-risk factor for alcohol use, compared to educated.<br>Individuals residing in rural areas (APR = 1.05), self-reported comorbidities (APR = 1.21), family history of alcohol use (APR = 2.42) and tobacco use (APR = 2.42) were significantly associated with alcohol use.
**Sarma et al., 2019**<br><br>**India**<br>(Kerala, South India)	**Cross-sectional**<br>[(all individuals between 18-69 years old were eligible, in both rural & urban (49.3%) areas)]<br>**Community-based**	**12,012 adults**<br>(37% men)<br>42.5 (18–69 years)	**WHO STEPS instrument**<br>**GPAQ**<br>(Global Physical Activity Questionnaire)<br>**Anthropometric measurements**	**Current alcohol use:** intake of at least one standard drink of alcohol in the past 30 days.<br>**Current tobacco use**: use of any form of tobacco within the past 30 days.<br>**Raised Blood Pressure (BP):**<br>BP of ≥140/≥90 mm Hg, or if the person is currently using antihypertensive medication.	**Weighted means, Percentages with 95% CI, & variance inflation applied**<br>Current use of tobacco & alcohol in men was 20.3% & 28.9% respectively.<br>The overall prevalence of raised BP was 30.4%.
**Endashaw Hareru et al., 2022**	**Cross-sectional**<br>(Residents of Dilla town, Gedeo zone,	**666 participants**<br>(70% men)<br>Mean: 33.3 years	**AIDIT:** AUD<br>**Kessler Psychological**	**AUD:** AUDIT score of ≥ 8	**Bivariate & multivariate binary logistic regression analysis**<br>AUD during the past year was 30.6%.<br>Being male (AOR = 8.33), age of less than 33 years old (AOR =

For peer review only - http://bmjopen.bmj.com/site/about/guidelines.xhtml

**Ethiopia** (Dilla town)	Southern Ethiopia with age of ≥ 18 years) **Community-based**	(≥ 18 years)	**Distress Scale (K10): ASSIST 2.0:** current and lifetime substance use		1.78), current cigarette smoking (AOR = 2.49), current khat chewing (AOR = 6.23), high level of psychological distress (AOR = 7.69) and poor social support (AOR = 2.30) were significantly associated with AUD.
**Gutema et al., 2020**<br><br>**Ethiopia** (Arba Minch HDSS)	**Cross-sectional** (Adult residents of Arba Minch HDSS (nine Kebeles of Arba Minch Zuria District, Southern Ethiopia) **Community-based** (rural residents, 83.7%)	**3346 participants** (50% men) 44.6 years (25– 64 years)	**WHO STEPS instruments** (alcohol use) **SRQ-20** (mental stress status)	**HED or Excessive Alcohol Consumption:** use of ≥ 6 drinks for men and ≥ 4 drinks for women on a single occasion at least once per month. **Mental stress** (mild, moderate, and severe)	**Binary logistic regression**<br>Prevalence (HED) was 13.7%.<br>HED was associated with occupation (daily laborer: AOR 0.49; & housewives: AOR0.63 compared with farmers), wealth index (2nd quintiles: AOR 0.55 & 3rd quintiles: AOR 0.66) compared with 1st quintiles; & climatic zone (midland: AOR 1.80; highland: AOR 1.95 compared with lowland).<br>Tobacco use (AOR 4.28), & khat use (AOR 4.75) were also associated with HED.
**Legas et al., 2021**<br><br>**Ethiopia** (South Gondar)	**Cross-sectional** (adult residents whose age was 18 years and above in the South Gondar zone, 61.3% from urban areas) **Community-based**	**848 (interviewed)** (62.3% men) ? (≥ 18 years)	**AUDIT**-AUD **PHQ-9** **PSS**-Perceived stress scale questionnaire) **Oslo social support scale** **SPIN**-Social phobia inventory scale	**AUD**: score of 8 or above on AUDIT **Depression:** A score of five or more on the PHQ-9	**Bivariate & multivariable logistic analysis**<br>AUD over the last 12-months was 23.7%.<br>16.50% had hazardous alcohol use, 5.2% had harmful alcohol use, and 2% had probable alcohol dependence.<br>Being male (AOR = 4.34), poor social support (AOR = 1.95), social phobia (AOR = 1.69), perceived high level of stress (AOR = 2.85), current cigarette smoking (AOR = 3.06) and comorbid depression (AOR = 1.81) were significantly associated with AUD.
**Wainberg et al., 2018**<br><br>**Mozambique** (Zambézia Province)	**Cross-sectional** (2014 survey) (16 year or older female heads-of-household in Mozambique, Zambézia Province) **Population-based** /rural Mozambique	**2,752 participants** (no men, 100% female) Median: 27 years (16-62 years)	**AUDIT** (Alcohol use) **PHQ** (Depression)	**Hazardous, harmful & high-risk drinkers:** AUDIT scores > 4 (recommended cutoff for women) **Depression:** A score of ≥ 10 on PHQ-8 (associated with clinical depression)	**Binomial logistic regression model:**<br>Overall prevalence of current alcohol consumption among female heads of hh was 15%. "hazardous drinkers" was 8%.<br>A positive depression screening (aOR: 2.20), death of a child (aOR: 2.44), & currently being pregnant (1.83) were associated with increased odds of hazardous drinking.<br>Being single (aOR: 0.48) & experiencing food insecurity (aOR:0.96) were associated with reduced odds of risky drinking.
**Bete et al., 2022**	**Cross-sectional**	**955 adults**	**ASSIST**	**Current and ever**	**Bi-variable & multivariate binary logistic regressions:**

For peer review only - http://bmjopen.bmj.com/site/about/guidelines.xhtml

**Ethiopia** (Harari regional state)	(residents aged >18 years) **Community-based** (80.55% urban dwellers)	(44.18% men) 42.28 years (> 18 years)		**substance users:** use of a specified substance (for non-medical purposes) in last 3 months and once in lifetime respectively	The overall prevalence of **current alcohol use was (**8.24%) , tobacco use (14.5%), and khat use (63.30%). The availability of alcohol, being unemployed, and being a current khat user were significantly associated with current alcohol use.
**Castelo Branco and de Vargas, 2023** **Brazil** (Northern Brazilian Amazon, state of Amapa)	**Cross-sectional** (Karipunan respondents aged ≥ 15) **Population-based**	**230 participants** (51.3%, men) ? (≥ 15)	**AUDIT**	**hazardous/harmful alcohol use (Zones II-IV of AUDIT Score, problematic use):** AUDIT score > 8.	**Fisher's exact test, & logistic regression: simple & multiple** (Hosmer-Lemeshow test/C statistic, & Spearman correlation tests) Prevalence of alcohol use: 70%; 59.6% (low-risk use), **38.3 (hazardous/harmful alcohol use), & 2.2% (probable alcohol dependence). Overall, 40.5% had hazardous or harmful alcohol use**; **66.6%** were men, and **33.4%** were women. Being male sex (AOR: 3.30), being Catholic (5.53) compared to Evangelical were associated with hazardous or harmful alcohol use.
**Rezaei et al., 2022** **Iran** **(national survey)**	**Cross-sectional** (The STEPs survey in Iran, 2016) **Population-based** (urban residents, 71.09%)	**29,068** participants (47.92%, men) 44.4 years (18 to 100 years)	**WHO's guidelines** (WHO STEPS instrument)	**Current alcohol consumption:** drink alcohol in past 12 months **Lifetime consumption:** ever drink alcohol in life.	**Univariate and multiple logistic regression analysis** National level prevalence rates of lifetime and current alcohol consumption were 8.00% and 4.04% respectively. The highest prevalence was reported among 25-34 years old. Individuals of higher socioeconomic status consumed significantly greater levels of alcohol. Current alcohol drinkers were 2 times more prone to trafic injury as compared to nondrinkers (ORadj: 2.0).
**Tegegne et al., 2023** **Ethiopia** **(national survey)**	**Cross-sectional** (2016 Ethiopian Demographic and Health Surveys (EDHS) **Population-based** (80.29%, rural)	**12,688** participants (100%, male) 30.92 years (15-59 years)	**QFQs**	**Ever alcohol drinking:** drinks alcohol during the lifetime.	**Multilevel multinomial logistic regression** Only Khat users (22.0%), only **Alcohol users (35.6%)**, and dual Alcohol and Khat users were (9.0%). At the individual level: age group of 30-44 years (AOR: 1.75) and 45-59 years (AOR:1.62) are more likely to drink alcohol compared to 15-29 years. Higher educational level (AOR: 1.4) compared to no education and having occupation (AOR:1.88) compared to people without occupation, increased the odds of drinking alcohol. Divorced males (AOR: 0.5) compared to single males; Protestant (AOR: 0.01), Muslim (AOR: 0.04), and other religion follower males (AOR: 0.35) compared to Orthodox religion have lower likelihood of alcohol drinking.
**Wolde, 2023** **Ethiopia**	**Cross-sectional** (elderly people living in towns in	**382 elderly people** (34.5%, male) 67 years	**AUDIT** **ASSIST**	**Alcohol Use Disorder (AUD):** AUDIT score > 8.	**Bivariate and multivariable logistic regression model** Magnitude of **AUD**, current alcohol use, and life-time alcohol use was **27.5%**, 52.4%, and 89.3%, respectively.

For peer review only - http://bmjopen.bmj.com/site/about/guidelines.xhtml

(South West Ethiopia)	Ethiopia) **Community-based**	(≥ 60 years)			AUD was associated with cognitive impairment (AOR: 2.53), poor sleep quality (AOR: 2.67), chronic medical illness (AOR: 3.27), and suicidal ideation or attempt (AOR: 2.07).
**Abrevations:** **AA**: Alcohol Abuse; **AD**: Alcohol Dependence; **aPR**: adjusted Prevalence Ratio; **ASSIST**: Alcohol, Smoking, and Substance Involvement Screening Test; **AUD**: Alcohol Use Disorder; **AUDIT**: Alcohol Use Disorder Identification Test; **BD:** Binge drinking**; CAGE**: Cut down, Annoyed, Guilty feeling & Eye opener; **CESD:** Center for Epidemiologic Studies Depression Scale; **FAST**: Fast Alcohol Screening Test; **FTND:** Fagerstrom Test for Nicotine Dependence; **HD**: Heavy drinking; **HED**: Heavy Episodic Drinking; **wk**: week; **M:** men; **MDE:** Major Depressive Episode; **NIAAA**: National Institute on Alcohol Abuse and Alcoholism; **PHQ-9:** Patient Health Questionnaire-9 item; **PR:** Prevalence Ratio; **PRIME**: Programme for Improving Mental Healthcare; **QFQs**: quantity/frequency questionnaires; **QoL:** Quality of Life; **RMBH:** metropolitan region of Belo Horizonte; **RR:** response rate**; SD:** Standard drink; **W:** women; **yr.**: year; **?:** mean age or age range for subjects is not determined.					

For peer review only

For peer review only - http://bmjopen.bmj.com/site/about/guidelines.xhtml

Supplementary File 5

Newcastle-Ottawa Scale (NOS) quality assessment summary for the study "A scoping review of assessment tools for, magnitudes of, and factors associated with problem drinking in population-based studies," 2023.

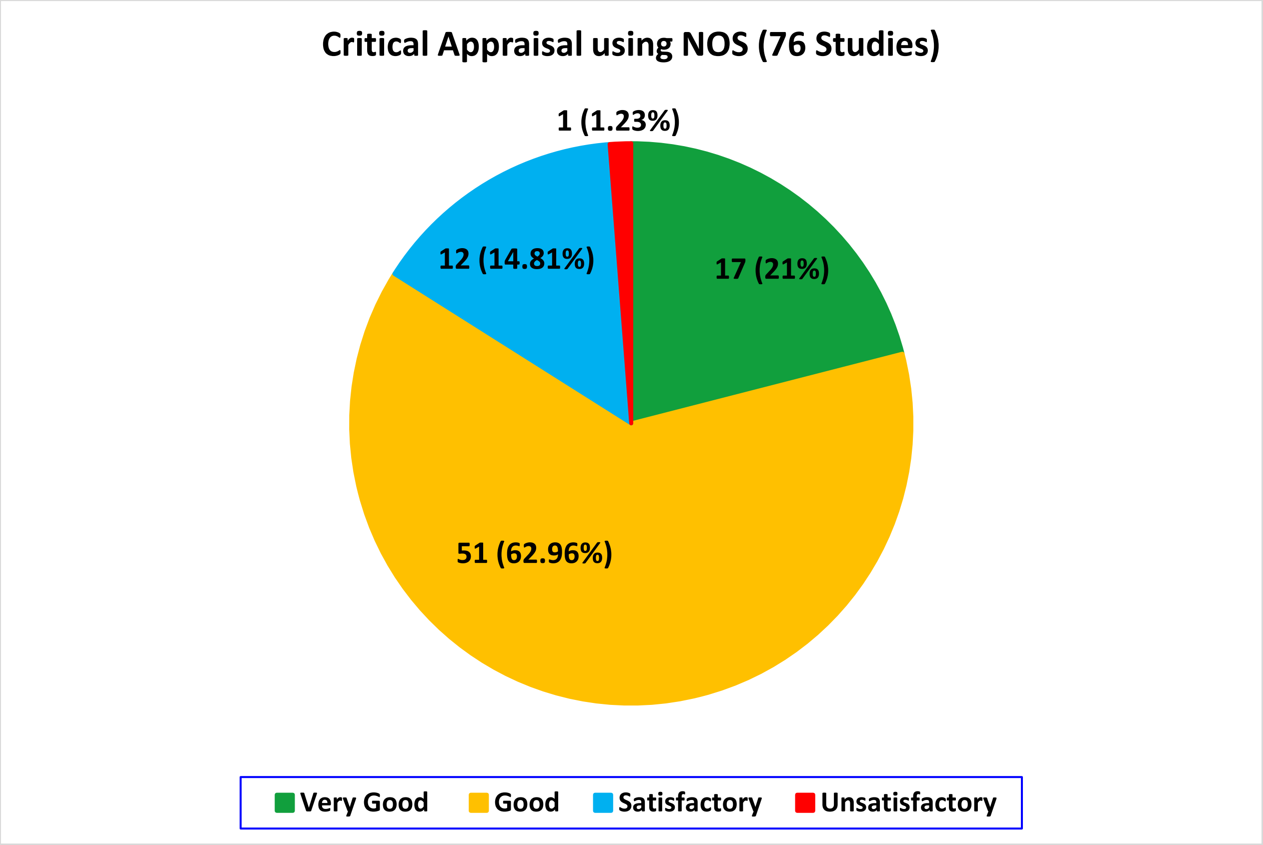

Figure: Newcastle-Ottawa Scale (NOS) quality assessment reports of studies for the study "A scoping review of assessment tools for, magnitudes of, and factors associated with problem drinking in population-based studies," 2023.

Table: Newcastle-Ottawa Scale (NOS) quality assessment- item level summary for "A scoping review of assessment tools for, magnitudes of, and factors associated with problem drinking in population-based studies," 2023.	Studies (81)
**Selection: (Maximum 5 points/scores/stars)**	
**1. Representativeness of the sample:**	
a. Truly representative of the average in the target population. * (all subjects/consecutive or random sampling)	**69**
b. Somewhat representative of the average in the target group. * (non-random sampling)	**12**
c. Selected group of users/convenience sample.	**0**
d. No description of the derivation of the included subjects (sampling strategy).	**0**
**2. Sample size:**	
a. Justified and satisfactory (including sample size calculation). * (1 score)	**44**
b. Not justified	**23**
c. No information provided	**14**
**3. Non-respondents:**	
a. Proportion of target sample recruited attains pre-specified target or basic summary of non-respondent characteristics in sampling frame recorded. *	**72**
b. Unsatisfactory recruitment rate, no summary data on non-respondents.	**02**
c. No information provided	**07**
**4. Ascertainment of the exposure (risk factor/disease) or screening/surveillance (measuremnt) tool:**	
a. Secure record (medical charts) or validated measurement (screening/surveillance) tool. **	**28**
b. Non-validated measurement tool, but the tool is available or described or Self report. *	**52**
c. No description of the measurement tool.	**01**
**Comparability: (Maximum 2 stars)**	

For peer review only

**1. Comparability of subjects in different outcome groups on the basis of design or analysis. Confounding factors controlled.**	
a. Data/results adjusted for relevant predictors/risk factors/confounders e.g., age, sex, marital status, job etc. **	**68**
b. Data/results not adjusted for all relevant confounders/risk factors/information not provided.	**13**
**Outcome: (Maximum 3 stars)**	
**1. Assessment of outcome:**	
a. Independent blind (structured) assessment. **	**14**
b. Record linkage. **	**0**
c. Self report. *	**67**
d. No description.	**0**
**2. Statistical test:**	
a.Statistical test used to analyse the data clearly described, appropriate and measures of association presented including confidence intervals and probability level (p value). *	**74**
b.Statistical test not appropriate, not described, or incomplete.	**07**

For peer review only

Preferred Reporting Items for Systematic reviews and Meta-Analyses extension for Scoping Reviews (PRISMA-ScR) Checklist

SECTION	ITEM	PRISMA-ScR CHECKLIST ITEM	REPORTED ON PAGE #
**TITLE**			
Title	1	Identify the report as a scoping review.	
**ABSTRACT**			
Structured summary	2	Provide a structured summary that includes (as applicable): background, objectives, eligibility criteria, sources of evidence, charting methods, results, and conclusions that relate to the review questions and objectives.	
**INTRODUCTION**			
Rationale	3	Describe the rationale for the review in the context of what is already known. Explain why the review questions/objectives lend themselves to a scoping review approach.	
Objectives	4	Provide an explicit statement of the questions and objectives being addressed with reference to their key elements (e.g., population or participants, concepts, and context) or other relevant key elements used to conceptualize the review questions and/or objectives.	
**METHODS**			
Protocol and registration	5	Indicate whether a review protocol exists; state if and where it can be accessed (e.g., a Web address); and if available, provide registration information, including the registration number.	
Eligibility criteria	6	Specify characteristics of the sources of evidence used as eligibility criteria (e.g., years considered, language, and publication status), and provide a rationale.	
Information sources*	7	Describe all information sources in the search (e.g., databases with dates of coverage and contact with authors to identify additional sources), as well as the date the most recent search was executed.	
Search	8	Present the full electronic search strategy for at least 1 database, including any limits used, such that it could be repeated.	
Selection of sources of evidence†	9	State the process for selecting sources of evidence (i.e., screening and eligibility) included in the scoping review.	
Data charting process‡	10	Describe the methods of charting data from the included sources of evidence (e.g., calibrated forms or forms that have been tested by the team before their use, and whether data charting was done independently or in duplicate) and any processes for obtaining and confirming data from investigators.	
Data items	11	List and define all variables for which data were sought and any assumptions and simplifications made.	
Critical appraisal of individual sources of evidence§	12	If done, provide a rationale for conducting a critical appraisal of included sources of evidence; describe the methods used and how this information was used in any data synthesis (if appropriate).	
Synthesis of results	13	Describe the methods of handling and summarizing the data that were charted.	

St. Michael's
Inspired Care.
Inspiring Science.

For peer review only - http://bmjopen.bmj.com/site/about/guidelines.xhtml

SECTION	ITEM	PRISMA-ScR CHECKLIST ITEM	REPORTED ON PAGE #
**RESULTS**			
Selection of sources of evidence	14	Give numbers of sources of evidence screened, assessed for eligibility, and included in the review, with reasons for exclusions at each stage, ideally using a flow diagram.	
Characteristics of sources of evidence	15	For each source of evidence, present characteristics for which data were charted and provide the citations.	
Critical appraisal within sources of evidence	16	If done, present data on critical appraisal of included sources of evidence (see item 12).	
Results of individual sources of evidence	17	For each included source of evidence, present the relevant data that were charted that relate to the review questions and objectives.	
Synthesis of results	18	Summarize and/or present the charting results as they relate to the review questions and objectives.	
**DISCUSSION**			
Summary of evidence	19	Summarize the main results (including an overview of concepts, themes, and types of evidence available), link to the review questions and objectives, and consider the relevance to key groups.	
Limitations	20	Discuss the limitations of the scoping review process.	
Conclusions	21	Provide a general interpretation of the results with respect to the review questions and objectives, as well as potential implications and/or next steps.	
**FUNDING**			
Funding	22	Describe sources of funding for the included sources of evidence, as well as sources of funding for the scoping review. Describe the role of the funders of the scoping review.	

JBI = Joanna Briggs Institute; PRISMA-ScR = Preferred Reporting Items for Systematic reviews and Meta-Analyses extension for Scoping Reviews.

* Where *sources of evidence* (see second footnote) are compiled from, such as bibliographic databases, social media platforms, and Web sites.

† A more inclusive/heterogeneous term used to account for the different types of evidence or data sources (e.g., quantitative and/or qualitative research, expert opinion, and policy documents) that may be eligible in a scoping review as opposed to only studies. This is not to be confused with *information sources* (see first footnote).

‡ The frameworks by Arksey and O'Malley (6) and Levac and colleagues (7) and the JBI guidance (4, 5) refer to the process of data extraction in a scoping review as data charting.

§ The process of systematically examining research evidence to assess its validity, results, and relevance before using it to inform a decision. This term is used for items 12 and 19 instead of "risk of bias" (which is more applicable to systematic reviews of interventions) to include and acknowledge the various sources of evidence that may be used in a scoping review (e.g., quantitative and/or qualitative research, expert opinion, and policy document).

From: Tricco AC, Lillie E, Zarin W, O'Brien KK, Colquhoun H, Levac D, et al. PRISMA Extension for Scoping Reviews (PRISMAScR): Checklist and Explanation. Ann Intern Med. 2018;169:467–473. doi: 10.7326/M18-0850.

For peer review only - http://bmjopen.bmj.com/site/about/guidelines.xhtml

A scoping review of assessment tools for, magnitudes of, and factors associated with problem drinking in population-based studies

Journal:	*BMJ Open*
Manuscript ID	bmjopen-2023-080657.R2
Article Type:	Original research
Date Submitted by the Author:	08-Feb-2024
Complete List of Authors:	Gizachew, Kefyalew Dagne; Addis Ababa University, Department of Psychiatry, School of Medicine, College of Health Sciences; Debre Berhan University, Department of Psychiatry, College of Health Sciences and Medicine<br>Myers, Bronwyn; Curtin University, Curtin enAble Institute, Faculty of Health Sciences; South African Medical Research Council, Alcohol, Tobacco and Other Drug research Institute<br>Awoke, Mihretu; Addis Ababa University, Department of Psychiatry, School of Medicine, College of Health Sciences<br>Teferra, Solomon; Addis Ababa University, Department of Psychiatry, School of Medicine, College of Health Sciences
**Primary Subject Heading**:	Mental health
Secondary Subject Heading:	Mental health, Public health, Addiction, Epidemiology
Keywords:	Systematic Review, Ethanol, AUDIT, EPIDEMIOLOGIC STUDIES, MENTAL HEALTH, Substance misuse < PSYCHIATRY

I, the Submitting Author has the right to grant and does grant on behalf of all authors of the Work (as defined in the below author licence), an exclusive licence and/or a non-exclusive licence for contributions from authors who are: i) UK Crown employees; ii) where BMJ has agreed a CC-BY licence shall apply, and/or iii) in accordance with the terms applicable for US Federal Government officers or employees acting as part of their official duties; on a worldwide, perpetual, irrevocable, royalty-free basis to BMJ Publishing Group Ltd ("BMJ") its licensees and where the relevant Journal is co-owned by BMJ to the co-owners of the Journal, to publish the Work in this journal and any other BMJ products and to exploit all rights, as set out in our licence.

The Submitting Author accepts and understands that any supply made under these terms is made by BMJ to the Submitting Author unless you are acting as an employee on behalf of your employer or a postgraduate student of an affiliated institution which is paying any applicable article publishing charge ("APC") for Open Access articles. Where the Submitting Author wishes to make the Work available on an Open Access basis (and intends to pay the relevant APC), the terms of reuse of such Open Access shall be governed by a Creative Commons licence – details of these licences and which Creative Commons licence will apply to this Work are set out in our licence referred to above.

Other than as permitted in any relevant BMJ Author's Self Archiving Policies, I confirm this Work has not been accepted for publication elsewhere, is not being considered for publication elsewhere and does not duplicate material already published. I confirm all authors consent to publication of this Work and authorise the granting of this licence.

For peer review only

A scoping review of assessment tools for, magnitudes of, and factors associated with problem drinking in population-based studies

Kefyalew Dagne Gizachew [1, 2], Bronwyn Myers [3,4,5], Awoke Mihretu [1] & Solomon Teferra [1]

[1] Department of Psychiatry, School of Medicine, College of Health Sciences, Addis Ababa University, Addis Ababa, Ethiopia

[2] Department of Psychiatry, College of Health Sciences and Medicine, Debre Berhan University, Debre Berhan, Ethiopia

[3] Curtin enAble Institute, Faculty of Health Sciences, Curtin University, Kent Street, Bentley, WA 6102, Australia

[4] Alcohol, Tobacco and Other Drug Research Unit, South African Medical Research Council, Cape Town, South Africa.

[5] Division of Addiction Psychiatry, Department of Psychiatry and Mental Health, University of Cape Town, Cape Town, South Africa

***Corresponding Author:** Kefyalew Dagne Gizachew

Tel: +251910487276; P.O. Box: 9086; E-mail: kdgc08@yahoo.com

ABSTRACT

Background: The term "problem drinking" includes a spectrum of alcohol problems ranging from excessive or heavy drinking to alcohol use disorder. Problem drinking is a leading risk factor for death and disability globally. It has been measured and conceptualized in different ways, which has made it difficult to identify common risk factors for problem alcohol use. This scoping review aims to synthesise what is known about the assessment of problem drinking, its magnitude, and associated factors.

Methods: Four databases (PubMed, EMBASE, PsycINFO, Global Index Medicus/GIM) and Google Scholar were searched from inception to November 25, 2023. Studies were eligible if they focused on people aged 15 and above, were population-based studies reporting problem alcohol use, and published in the English language. This review was reported based on guidelines from the "Preferred Reporting Items for Systematic Reviews

For peer review only

and Meta-Analyses extension for Scoping Reviews (PRISMA-ScR) Checklist." Critical appraisal was done using the Newcastle-Ottawa Scale (NOS).

Results: From the 14,296 records identified, 10,749 underwent title/abstract screening, of which 352 full-text articles were assessed, and 81 articles were included for data extraction. Included studies assessed alcohol use with self-report quantity/frequency questionnaires, criteria to determine risky single occasion drinking, validated screening tools, or structured clinical and diagnostic interviews. The most widely used screening tool was the Alcohol Use Disorder Identification Test. Studies defined problem drinking in various ways, including excessive/heavy drinking, binge drinking, alcohol use disorder, alcohol abuse, and alcohol dependence. Across studies, the prevalence of heavy drinking ranged from <1.0% to 53.0%, binge drinking from 2.7% to 48.2%, alcohol abuse from 4.0% to 19.0%, alcohol dependence from 0.1% to 39.0%, and alcohol use disorder from 2.0% to 66.6%. Factors associated with problem drinking varied across studies. These included socio-demographic and economic factors (age, sex, relationship status, education, employment, income level, religion, race, location, and alcohol outlet density) and clinical factors (like medical problems, mental disorders, other substance use, and quality of life).

Conclusions: Due to differences in measurement, study designs, and assessed risk factors, the prevalence of and factors associated with problem drinking varied widely across studies and settings. The alcohol field would benefit from harmonized measurements of alcohol use and problem drinking as this would allow for comparisons to be made across countries and for meta-analyses to be conducted.

Scoping review registration: Open Science Framework (OSF) ID: https://osf.io/2anj3.

Keywords: Scoping review, Alcohol, Alcohol use disorder, Problem drinking, Heavy drinking, Binge drinking, Heavy episodic drinking, Alcohol use assessment

For peer review only

ARTICLE SUMMARY

Strengths and limitations of this study

- ► To the authors' knowledge, this is the first scoping review to synthesise the evidence on the prevalence of and factors associated with problem drinking across global settings.
- ► Strengths include an extensive search of four databases, with 81 original articles included for evidence synthesis.
- ► The review was limited to the community-based studies; studies conducted at institutions like hospitals, primary health care centers (PHC), addiction centers, and colleges or universities were not included.

For peer review only

INTRODUCTION

The nature of alcohol use, related issues, and how they manifest throughout life have long been the subject of scientific research (1). In 2016, the "Global Burden of Disease Study" identified alcohol use as a leading risk factor for death and disability, ranking it seventh among the top risk factors for disability-adjusted life years (DALYs) and deaths globally (2, 3). Alcohol use has been identified as a risk factor for more than 200 injuries and diseases, including alcohol use disorder, liver cirrhosis, malignancies, injuries, tuberculosis, HIV/AIDS (4, 5), noncommunicable diseases (NCDs) (6), mental disorders (7), violence-related harms and injuries (8). These problems can arise from acute episodes of alcohol intoxication or chronic, heavy alcohol use (9). The phrase "alcohol use disorder" (AUD) describes the complete range of problematic patterns of alcohol use, ranging from less severe difficulties such as heavy episodic/binge drinking (HED/BD) and risky drinking to harmful drinking and more serious disorders like alcohol abuse (AA) and alcohol dependence (AD) (10). These different definitions of problem alcohol use and inconsistent ways of measuring these problems have contributed to challenges in understanding the nature and extent of alcohol-related problems across the AUD continuum. In this review, we use the term "problem drinking" to refer to any problem with alcohol use, including AUD. Different definitions and terms for problem alcohol use (11-26) are summarised in Table 1.

Table 1: Different definitions and terms for problem alcohol use.

Terms	Definitions
**Low-risk drinking**	Generally defined as a daily intake of no more than 20g of alcohol with at least two non-drinking days weekly. Low-risk drinking limits are defined differently for cis-gender males and females, i.e., not more than three and two drinks a day on average, respectively (20).
**Problem Drinking (PD)**	Problem drinking, commonly referred to as "alcohol abuse," "alcohol misuse," or "AUD," is a pattern of alcohol intake that harms one's health or relationships with others. It is a general term that covers a range of alcohol-related problems, from mild to severe (11-16).

For peer review only - http://bmjopen.bmj.com/site/about/guidelines.xhtml

**Hazardous drinking**	A quantity or pattern of alcohol intake that puts individuals at risk for adverse health events, which carry the possibility of physical or psychological harm (17, 18).
**Harmful drinking**	A quantity and pattern of alcohol intake that causes physical or psychological harm and the presence of physical or psychological complications (17, 19).
**Heavy episodic/binge drinking (HED/BD)**	Defined as the intake of five or more drinks for men and four or more drinks for women per occasion in most studies (roughly 60 grams of pure alcohol), which brings blood alcohol concentration (BAC) levels to 0.08 gram/dL in about two hours (21).
**Excessive/heavy drinking (HD)**	Heavy drinking is the quantity of alcohol consumed that exceeds a set threshold. It is often defined as the weekly use of more than 14 drinks on average for males and more than seven drinks for females. Some countries define it as the average number of binge episodes per person during 30 days or weekly drinking of more than 21 drinks for males and more than 14 drinks for females (21-24).
**Alcohol dependence (AD)**	Based on the Diagnostic and Statistical Manual of Mental Disorders- 4th edition (DSM-IV), alcohol dependence is characterized by a problematic pattern of alcohol use that results in clinically significant impairment or distress. It is also a symptom of continuing to use alcohol despite knowing that continued use will cause serious social or interpersonal problems (for example, violent arguments with their spouse while intoxicated or abusing children) (25).
**Alcohol abuse (AA)**	Alcohol abuse is a pattern of alcohol intake that has adverse outcomes and harms a person's physical health, mental health, interpersonal connections, and general functioning. Alcohol abuse involves excessive and frequent alcohol consumption despite its harmful effects. It can be less severe than alcohol dependence because it requires fewer symptoms and can only

For peer review only

	be diagnosed once the DSM-IV criteria have determined that alcohol dependence is not present (25).
**Alcohol use disorder (AUD)**	AUD is a chronic medical disorder defined by an individual's compulsive and problematic pattern of alcohol consumption, diagnosed when an individual's alcohol consumption leads to significant distress or impairment in their daily functioning. It is characterized by a cluster of behavioral and physical symptoms, including withdrawal, tolerance, and craving, based on the Diagnostic and Statistical Manual of Mental Disorders- 5th edition (DSM-5) (11, 26).

Alcohol consumption is responsible for a wide range of adverse health outcomes (3), and alcohol-related harms are well established (27). Problem drinking, including any form of AUD, is a critical public health issue that has an impact on people and communities all around the world (28).

Risk factors for the emergence and advancement of problem drinking are not well understood (2). Despite the severe burden of alcohol use globally, there is fragmented evidence on the contribution of specific risk factors to problem drinking (2).

Although alcohol consumption occurs on a continuum, our understanding of when to intervene and risk factors to target in interventions is hampered by differences in how problem drinking is conceptualized and measured and the lack of synthesised evidence on factors associated with problem drinking.

A comprehensive global review of evidence on the nature and extent of problem drinking serves several essential purposes. First, it offers crucial epidemiological data, such as burden or prevalence rates, trends, and problem drinking patterns over time. With this information, public policymakers, researchers, and healthcare workers may more accurately understand the scope of the problem, pinpoint individuals at high risk, and more effectively allocate resources to problem drinking prevention and treatment. Second, the information from the review may be utilized to create awareness of problem drinking and develop policy initiatives on screening and treatment strategies to reduce its prevalence. Third, studying problem drinking data enables a clearer understanding of

For peer review only - http://bmjopen.bmj.com/site/about/guidelines.xhtml

factors related to the development or progression of problem drinking. This information is needed to guide prevention initiatives and treatments focusing on specific risk factors, such as the environment, clinical variables, and comorbid mental health problems.

Previous reviews recommended a need for further research on the magnitude of problem drinking, focusing on LMICs (2). These reviews targeted specific regions, contexts, and populations and focused on a particular type of problem drinking pattern or set of risk factors to the exclusion of others. A review covering a broader range of measures, definitions, and associated risk factors will provide a more integrated understanding of the phenomenon, and this will provide an opportunity to identify commonalities and variations of problem drinking across diverse settings and populations (2).

In summary, this review aims to synthesise the global literature on the nature and extent of problem drinking, how problem drinking was assessed, and factors associated with problem drinking among the general population.

METHODS

This scoping review was reported based on guidelines from the "Preferred Reporting Items for Systematic Reviews and Meta-Analyses extension for Scoping Reviews (PRISMA-ScR) Checklist," a tool that is used to guide the scoping review process (29). A copy of the PRISMA-ScR checklist for scoping reviews is supplemented as an additional file (Research Checklist 1).

Eligibility criteria

For this review, only articles written in the English language were considered. The PICO framework for prevalence studies (Population, Measurement of presence of disease, Design, and Setting) guided the choice of eligibility criteria. Accordingly, for studies to be included, they had to (a) study people aged 15 years or older (Population); (b) report problem drinking or AUD using any screening scales, measures, instruments, clinical diagnostic interviews or laboratory tests to detect alcohol use (Measurement of the presence of disease); (c) have any epidemiological, population-based design (Design); and (d) be located in any country or type of setting, as long as the study had a community-based sample (Setting). Due to the inclusion of all prevalence studies on problem drinking with a global focus and the broad coverage of settings, only population-based studies are

For peer review only

For peer review only - http://bmjopen.bmj.com/site/about/guidelines.xhtml

included in this scoping review, and studies conducted at PHC, hospital settings, universities, or schools are excluded.

Information sources

The literature search included four databases: PubMed, EMBASE, PsycINFO, and Global Index Medicus (GIM) and searched from database inception (spanning from 1996, 1974, 1906, and 1948 respectively) to August 26, 2019. Database searching was updated twice: first on July 22, 2022, and second on November 25, 2023. Additional records were identified through other sources, such as Google Scholar.

To ensure methodological rigor, a scoping review protocol for the review was registered with Open Science Framework (OSF), which can be accessed using associated project ID of https://osf.io/2anj3 or registration DOI of https://doi.org/10.17605/OSF.IO/9SYV7.

Search Criteria

The PI (KD) developed the search strategy with close consultations with supervisors (ST and BM). The search strategy consisted of key terms, free texts, and controlled vocabulary search terms such as (Medical Subject Heading/MeSH terms for MEDLINE and Emtree terms for Embase) for the main big terms of "prevalence," "alcohol," and "community/population-based health surveys." Terms within each set were grouped using Boolean "OR" operators, and terms across sets were combined using "AND" operators.

Although our scoping review has a global focus, 'Ethiopia' is included as a search term in our search strategy for all databases. Since this scoping review is a formative stage of connected consecutive studies on problem drinking and related alcohol use conditions in Ethiopia and intended to inform further studies, we did not want to miss out on any alcohol-related studies in Ethiopia. Since the Boolean Operator used here is (OR) with the study focus (community/population-based studies), including the term 'Ethiopia' as a search term did not limit the search to studies conducted in Ethiopia or detract from the review's global focus. Terms related to alcohol use and the search strategy for searched databases are included in (Supplementary File 1).

Selection of sources of evidence

For peer review only

After the databases were searched, the titles and abstracts of identified records were imported into EndNote software for deduplication and to facilitate the review process. Two reviewers (KD and AM) independently completed screening article titles and abstracts in the first stage and screening full-text articles in the second stage using a priori inclusion and exclusion criteria to determine eligibility. These two reviewers met to resolve screening and selection differences with discussion and to reach a consensus on whether to include an article. These two independent reviewers assessed the eligibility of 352 full-text articles for the final inclusion of 81 articles in the scoping review. These reviewers achieved a 96.6% level of agreement on which articles to include in the review.

Data charting process

We developed a data extraction form that included items relating to study characteristics (author, year of publication and citation, study country/location), study design, study setting and population, sample size, study tools or measures, and results. Two reviewers (KD and AM) independently extracted data from included studies using this form. These reviewers met to resolve data extraction differences with discussion and to reach a consensus on what to extract from the included articles.

Collating, summarising, and reporting the results

As a scoping review, the aim was to map and aggregate findings to offer and present an overview of the topic and all the material studied. Data were analyzed using descriptive statistics, and the results were reported using narrative synthesis and presented in tables. Although critical appraisal of the quality of included studies is not mandatory in scoping reviews, we decided to assess study quality so that findings from the current scoping review could inform the selection of alcohol screening tools and measures in future studies. We used the "Newcastle-Ottawa Quality Assessment Scale (NOS)" for cross-sectional studies (30-32). We slightly modified the semantics of some items to better align with this review (Supplementary File 2). The tool has three domains, each with maximum stars (points/scores): i) selection (maximum five stars/*****), ii) comparability (maximum two stars/**), and iii) outcome (maximum three stars/***) giving a total score of 10. Studies that scored 9-10 points were considered very good, those that scored 7-8 points were rated as good, those that scored 5-6 points were rated as satisfactory, and those that scored 4 points or less were rated as unsatisfactory (31).

For peer review only

Patient and Public Involvement

There was no patient or public involvement in this scoping review.

RESULTS

The search yielded 14,296 articles from all databases and three additional records from Google Scholar. After deduplication, there were 10,749 records, and all these articles underwent title and abstract screening. After titles/abstracts screening, 352 articles were assessed for full-text eligibility, of which 81 articles were included for data extraction. The PRISMA flow diagram summarizes this article selection process (Figure 1).

Characteristics of included studies

The publication year for included articles ranged from 1996 to 2023. Only five studies were published before 2000, 19 from 2000-2010, and 57 from 2011-2023. The extracted results of articles from high-income countries (HICs) and low- and middle-income countries (LMICs) are presented separately in two tables, not for specific purposes but for better visualization. Of the 81 full-text articles included in this scoping review, 29 were from HICs (Table: Supplementary File 3), and the remaining 52 studies were from LMICs (Table: Supplementary File 4). Of these 52 studies, 38 were from middle-income countries, 25 were from upper-middle-Income countries, 13 were from lower-middle-income countries, and 14 were from low-income countries.

Most of the studies employed a cross-sectional study design (73/81), and the rest of the studies were longitudinal/cohort designs (6/81) or mixed quantitative and qualitative designs (2/81). For the majority of included studies (n=30, 37.0%), the study population resided in an urban location, followed by a mixed urban/rural setting (n=27, 33.3% of studies) and rural (n=9, 11.1%). Fifteen (18.5%) studies did not specify the location of the population.

Among the included studies, the total sample size ranged from 99 to 358,355 participants. Only 11 studies had a sample size of less than 500 individuals. Almost 74.1% (n=60) of the studies included had more than 1000 participants in their sample. Nine studies were conducted only among men, two only among women, and four studies did not specify gender. Four studies were conducted among young adults (16-25 years old) and seven among older people (adults ≥50 years old). Across studies, participants ranged from 15 to 100 years old, and the mean or median age ranged from 20 to 81.

For peer review only

For peer review only - http://bmjopen.bmj.com/site/about/guidelines.xhtml

Critical appraisal of included studies

When assessing the overall methodological quality of included studies, 17 (21.0%) were rated as very good, 51 (63.0%) as good, 12 (14.8%) as satisfactory, and one (1.2%) as unsatisfactory (see Supplementary File 5 for quality assessment).

Measurement of problem drinking

The included studies used a variety of methods to assess problem drinking, including self-report quantity/frequency (QF) questionnaires that included risky single occasion drinking (RSOD) criteria, validated screening tools, and structured clinical interviews or assessments (gold-standard).

Quantity/frequency (QF) questionnaires and risky single occasion drinking criteria

Of the 81 included studies, 19 of the 29 conducted in HICs (Table: Supplementary File 3) and 21 of the 52 conducted in LMICs (Table: Supplementary File 4) have used QF questionnaires. The time interval in which the pattern of alcohol consumption (frequency and quantity) was defined and reported was expressed in days, weeks, months, past 12 months (current use), and ever (lifetime) use. Some studies assessed adherence to country-specific guidelines of recommended limits as part of the QF questionnaires. These guidelines included the French alcohol consumption habits (33), Australian National Health and MRC 2009 guidelines for mean daily alcohol intake (34), the Health Council of Netherlands recommended limit for alcohol (35), and the UK National Statistics definition for BD or heavy drinking (36). Nine studies from HICs (e.g., Ireland (37) and Switzerland (38)) and four studies from LMICs applied risky single occasion drinking criteria. Among HICs, a survey in the US used National Institute on Alcohol Abuse and Alcoholism (NIAAA) guidelines and Substance Abuse and Mental Health Services Administration (SAMHSA) definitions for BD (39, 40).

Screening and diagnostic interviews for problem drinking

Studies used a variety of screening tools to assess problem drinking. The most commonly used screening tools included the CAGE questionnaire (41-43), the Alcohol Use Disorders Identification Test (AUDIT) (17), the Michigan Alcohol Screening Test (MAST)

For peer review only

(44, 45), and the Alcohol, Smoking, and Substance Involvement Screening Test (ASSIST) (46).

Specifically, three studies from HICs (35, 47, 48) and four from LMICs (49-52) used the CAGE. Five studies from HICs, including New Zealand (53), the Netherlands (35), the UK (54), Norway (55), and Sweden (56), used either the full or abbreviated versions of the AUDIT. Similarly, 24 studies from LMICs used the AUDIT. The three-item AUDIT-C was used in South Africa, Cambodia, the UK, and Sweden (54, 56-58), and a four-item version of the AUDIT- the Fast Alcohol Screening Test (FAST) was used in Ethiopia (59). Only four studies in LMICs, conducted in Suriname (60), South Africa (61) and Ethiopia (62, 63) used the ASSIST.

The included studies have used five different AUD diagnostic interviews. First, several studies used the Composite International Diagnostic Interview (CIDI) (64-68). Eleven studies from HICs including Hong Kong (69), Germany (70, 71), Israel (72), Australia (73), the Netherlands (74), Sweden (75), Ireland (37), USA (76), Finland (77), and Switzerland (38) used country-specific versions of CIDI-structured diagnostic tools based on DSM-III, DSM-III-R, DSM-IV, DSM-5, or ICD-10 and ICD-11 (78, 79) to detect and diagnose AUD, alcohol abuse, or alcohol dependence. It was also used in three studies from LMICs, including Sri Lanka (80), Ethiopia (51), and South Africa (81).

Second, Alcohol Abuse and Alcoholism's Alcohol Use Disorder and Associated Disabilities Interview Schedule-DSM-IV version (AUDADIS-IV) (82) was used in one study in the USA, a HICs (76).

Third, the Structured Clinical Interview for DSM-IV (SCID-I) (83-85) was used in a Finnish study (77) to detect lifetime DSM-IV substance use disorder (SUD).

Fourth, the Diagnostic and Statistical Manual of Mental Disorders- 4th and 5th editions (DSM-IV and DSM-5) (25, 26) was used by two HIC studies (from Switzerland (38) and Sweden (75)) to diagnose alcohol abuse, alcohol dependence, or AUD.

Fifth, studies used the Mini International Neuropsychiatric Interview (M.I.N.I.) versions 5, 6, & 7.0.2 (86-88) to detect AUD. This is a DSM-IV-based diagnostic tool for detecting alcohol abuse and dependence during the past 12 months. Only one HIC study (from the USA) used the M.I.N.I. (89). It was employed for the detection of alcohol abuse or

For peer review only

For peer review only - http://bmjopen.bmj.com/site/about/guidelines.xhtml

dependence in three studies from LMICs, namely South Africa (81), Malaysia (90), and Thailand (91).

Definitions of problem drinking

Studies defined problem drinking in a variety of ways, including HED/BD, excessive (heavy) drinking, or AUD. Definitions of heavy drinking and HED/BD differed according to the recommended drinking limits of countries and how individual studies operationalized the construct. For instance, a study in Finland (47) defined heavy drinking for males as ≥280g of absolute ethanol or 24 drinks per week and/or a CAGE score ≥3 and for women as ≥190g of absolute ethanol or 16 drinks per week and/or a CAGE score ≥2. Another study in the USA (39) defined heavy drinking for males as >14 drinks per week and >4 drinks per day and for females as >7 drinks per week and >3 drinks per day. This weekly drinking definition of heavy drinking is also applied in China (92). A study in France (33) defined heavy drinking as ≥60g ethanol per day or six glasses per day of any alcoholic drink for males and ≥30g per day or about three glasses per day for females. Heavy drinking in two studies in the Netherlands (35, 74) and one study in Botswana (24) for women was >14 standard glasses per week, and for men, it was >21 drinks per week. Two studies in Brazil (49, 93) operationalized heavy drinking or hazardous drinking as an average of ≥30g per day, irrespective of gender. Studies from South Africa classified heavy drinking as >7 drinks per week (94).

HED was sometimes used interchangeably with BD. Studies in Hong Kong (69, 95) and the US (96) defined HED/BD as drinking ≥5 drinks in a row on a single occasion in the past month, irrespective of sex. Most studies described it differently for males and females. The NIAAA guidelines for risky drinking criteria, SAMHSA definition, or risky single occasion drinking criteria were mainly applied to define HED/BD (94, 97-99). In the US (76, 100), Singapore (101), Peru (97), South Africa (57), and Brazil (98, 99, 102), HED/BD was defined as ≥5 drinks per occasion for men and ≥4 drinks per occasion for women, a pattern of drinking that brings blood alcohol level (BAC) to at least 0.08 g/dl and reflects ≥60g pure alcohol. It was also defined like this by studies conducted in India and Ireland (37, 103). In South Africa, one study (94) used a cut-off of >3 drinks per occasion weekly, and another study (104) used ≥5 drinks on an average drinking day to define HED. Other studies defined HED/BD using different criteria. In Cambodia (58) and

For peer review only - http://bmjopen.bmj.com/site/about/guidelines.xhtml

Nepal (105), this was defined as the use of ≥6 drinks in a single sitting at least monthly using NIAAA definitions, and in Ethiopia (106, 107), as an intake of ≥6 drinks in males and ≥4 drinks in females on a single occasion. The definition of BD differed in a study conducted in the United Kingdom (36), with BD defined as >8 standard drinks per session for males and >6 standard drinks per session for females. Some studies examined risky single occasion drinking, defined as ≥6 drinks per single occasion, and at-risk volume drinking, defined as ≥21 drinks per week, and risky single occasion drinking at least monthly for men in Switzerland (38).

Hazardous/harmful alcohol use, also known as Harmful/hazardous drinking, probable AUD, risky alcohol use, high-risk drinking, or hazardous, harmful, or dependent alcohol use, was defined as a score of ≥8 on the AUDIT in most studies including studies conducted in New Zealand (53), Norway (55), Brazil (108, 109), South Africa (61, 102), India (110-113), Kenya (114), Uganda (115), Nepal (116), Ethiopia (63, 117-119), Malaysia (90), Thailand (91, 120), and Suriname (60). This definition is in keeping with the WHO recommended cut-offs for problem drinking on the AUDIT (17). In contrast, one study used an AUDIT score >4 to define hazardous, harmful, and high-risk drinking for females in Mozambique (121).

We noted more variability in the cut-offs used across studies when using short AUDIT forms to define hazardous or harmful drinking. A cut-off score of ≥5 on AUDIT-C (a three-item version of the full AUDIT) was used in South Africa (57) and the UK (54). Risky drinking was defined as 8-12 for males and 6-12 for females on AUDIT-C in Sweden (56), while hazardous alcohol use in Ethiopia (59) was defined as a score of ≥3 on the FAST. But a different definition was applied for hazardous drinking in Russia (122), which was stated as having any of the following in the past year: having drunk surrogate alcohols (non-beverage alcohols and illegally produced alcohols), having been on zapoi (several days of continuous drunkenness during which one withdraws from the society), having frequent hangovers once or more per month and having consumed spirits daily. One study in China (123) used the MAST to define cases of alcohol dependence, and it was classified using a MAST score of ≥5 with 1-4 (low), 5-6 (light), and 40-53 (severe).

Prevalence of problem drinking, its pattern, and associated factors

Prevalence and patterns of problem drinking

For peer review only - http://bmjopen.bmj.com/site/about/guidelines.xhtml

For peer review only

Six HIC studies assessed heavy drinking (Table: Supplementary File 3). Across these studies, the reported prevalence of heavy drinking ranged from 5.0% to 39.9% for males and from <1.0% to 12.9% for females (33, 34, 39, 47, 72). Heavy drinking was reported by eight out of 47 LMIC studies comprising Brazil (49, 93, 98), South Africa (94, 124), Botswana (24), China (92), and Brazil (52) (Table: Supplementary File 4). The prevalence of heavy drinking in these studies ranged from 3.2% to 53.0% in the overall population, 29.2% to 31.0% in males, and 3.7% to 17.0% in females.

HED/BD was reported in nine studies conducted in HICs, including Hong Kong (69), USA (40, 76, 96, 100), UK (36), Singapore (101), Chile (125), and Ireland (37) (Table: Supplementary File 3). Across these studies, the prevalence of HED/BD ranged from 14.5% to 24.7% in males, 3.5% to 18.0% in females, and 13.7% to 86.0% in the overall sample. HED/BD was also reported by fourteen out of 52 studies from LMICs consisting of South Africa (94, 102, 104), India (103), Cambodia (58), Peru (97), Brazil (98, 99), Nigeria (126), Burkina Faso (127), Nepal (105), and Ethiopia (106, 107, 117) (Table: Supplementary File 4). The overall prevalence of HED/BD ranged from 3.7% to 43.0%. The prevalence of HED/BD ranged from 13.7% to 48.2% in males and 2.7% to 15.0% in females.

The prevalence of AUD, including older diagnostic categories like alcohol abuse and alcohol dependence, was reported by 10 out of 29 HIC studies, including Hong Kong (69), Finland (77), Germany (70), Switzerland (38), Israel (72), Australia (73), UK (54), Sweden (75), Chicago, USA (89), and Ireland (37) (Table: Supplementary File 3). In these studies, the prevalence of any lifetime or current AUD ranged from 4.3% to 36.8% in the overall population, 19.8% to 38.3% in males, and 6.3% to 20.6% in females. The prevalence of alcohol abuse ranged from 4.0% to 4.5%, and alcohol dependence ranged from 0.4% to 12.3% in the overall sample, 6.1% in males, and 6.1% in females.

Likewise, AUD comprising alcohol abuse, alcohol dependence, hazardous, harmful, or dependent alcohol use was reported by 31 of 52 LMIC studies, including South Africa (57, 61, 81, 102), Sri Lanka (80), Ethiopia (50, 51, 59, 63, 117-119), China (123), Brazil (49, 52, 108, 109), India (110-113), Kenya (114), Uganda (115), Nepal (116), Cambodia (58), Malaysia (90), Thailand (91, 120), Suriname (60), and Mozambique (121) (Table: Supplementary File 4). Either current or lifetime prevalence of any AUD ranged from 4.1%

For peer review only - http://bmjopen.bmj.com/site/about/guidelines.xhtml

to 41.0% in the overall sample, from 14.5% to 66.6% in males, and from 2.0% to 33.4% in females. The prevalence of lifetime or current alcohol abuse ranged from 6.2% to 9.0% in the overall sample, estimated at 19.0% in males and 6.0% in females. The prevalence of lifetime or current alcohol dependence ranged from 0.8% to 26.5% in the overall population, from 1.5% to 39.0% in males, and from 0.1% to 19.1% in females.

Factors associated with problem drinking

Most studies from HICs and LMICs identified factors associated with different types of problem drinking. These factors can be grouped into socio-demographic and socio-economic; clinical (medical problems or clinical parameters and mental disorders); substance use and risky behaviours; and psychosocial support, functioning, disability, and quality of life factors (Table: Supplementary File 3 and Table: Supplementary File 4). Studies from both HICs and LMICs examined a range of socio-demographic factors associated with problem drinking, but the nature and direction of the relationship between these factors and problem drinking were inconsistent. Seven out of 29 studies in HICs found that age was associated with problem drinking. Some studies found that older age was associated with heavy drinking (35, 76), while others found that this association existed for men but not women (69). In contrast, other studies reported associations between problem drinking and young adulthood (72, 73), with some studies noting that alcohol use declined with age (56), and age was associated with abstention among women (39) and inversely associated with heavy drinking among men (33, 34). Furthermore, nineteen out of 52 studies in LMICs found that age was associated with problem drinking. Some studies reported that older age was associated with alcohol use and different types of problem drinking (49, 51, 59, 93, 102-104, 113-116, 128, 129), while others found that younger age was associated with problem drinking (58, 61, 93, 97, 118, 127).

Several studies found associations between male sex and problem drinking. Seven studies from HICs (35, 56, 70, 72, 73, 76, 89) found that male sex was associated with alcohol use and various types of problem drinking. Another 19 studies from LMICs found that male sex was associated with different forms of problem drinking (24, 50, 51, 57-59, 90, 93, 94, 105, 106, 109, 110, 114, 117-119, 127, 128).

For peer review only - http://bmjopen.bmj.com/site/about/guidelines.xhtml

Some studies from HICs found associations between not being in a relationship and problem drinking, including studies conducted in Australia (73), Israel (72), and China (69). Included studies from LMICs also reported associations between not being in a relationship and various types of AUD (50, 60, 81, 99, 103, 104, 116, 124). In contrast, only a handful of studies found that these associations existed for being in a relationship (24, 106, 121) and age-gap relationships (24).

In terms of socio-economic and environmental indicators, only a couple of studies from HICs examined associations between problem drinking and factors like educational attainment (33, 34, 74), employment (69), being immigrants (72), lower (39) or higher (34) income, location (33, 34), or higher neighborhood alcohol outlet density (40). Thirteen included studies from LMICs found that education was associated with problem drinking, with some studies finding that a lower educational level was associated with alcohol abuse and heavy drinking (49, 51, 60, 102, 103, 113, 122, 130). In contrast, others found that this association existed for higher educational levels (24, 61, 97, 99, 129). Thirty-three studies conducted in LMICs examined associations between problem drinking and economic factors, finding equivocal results. While several studies found associations between lower income (49, 50, 80, 81, 93, 102, 103, 128, 130) or unemployment (62, 122) and problem drinking, others found associations between problem drinking and higher income (57, 58, 94, 102, 107, 108, 110, 121, 122, 128, 131) or being employed (51, 58, 60, 105, 107, 110, 115-117, 127, 129). Only a few studies from LMICs examined associations between factors like religious affiliation (50, 90, 109, 129, 130), living in urban or rural setting and location (61, 102, 106, 107, 113); ethnicity and race (49, 50, 57, 61, 93, 94, 102, 105, 116); household living circumstances (49, 104) and problem drinking.

Three studies conducted in HICs (73) and fifteen in LMICs (50, 59, 61, 63, 80, 90, 93, 98, 108, 115-119, 121) found associations between mental disorders and different forms of problem drinking. Only one HIC study found associations between medical problems like higher BMI and being non-diabetic than diabetic (39) and problem drinking. In contrast, eight studies from LMICs found associations between medical problems like chronic disease (63, 93), high blood pressure (92, 123), obesity (94), self-reported physical comorbidities (113), traffic injury (131), and problem drinking. Only a few studies from

For peer review only

LMICs found associations between problem drinking and less psychosocial support (59, 118, 119), more impaired functioning, disability, poorer quality of life, cognitive impairment, and poor sleep quality (63, 99, 112, 116, 117). In terms of other substance use factors, seven studies were conducted in HICs (33-35, 69, 73, 76, 77), and seventeen studies from LMICs (50, 57, 61, 62, 80, 93, 94, 104, 106-108, 113, 116, 118, 119, 127, 128) reported associations between cigarette smoking, current khat use, other substance use and various types of problem drinking.

DISCUSSION

In this scoping review, we identified 81 population-based studies (29 from HICs and 52 from LMICs) that described the prevalence of alcohol consumption and problem drinking and factors associated with problem drinking. Based on the publication year of included articles, there were more than triple the number of published articles in the last decade compared to the previous decade. This increase in publications over time implies that researchers are more interested and involved in alcohol use studies than before.

Despite this growing body of evidence, this review highlights significant heterogeneity of study designs, measures, and outcomes that hamper the synthesis of evidence on alcohol prevalence and associated harms across studies. The development of the AUDIT (17) attempted to solve this heterogeneity in the measurement of problem drinking, but the uptake has not been significant.

More specifically, this review identified significant heterogeneity and inconsistency in how various forms of problem drinking were defined and measured (24, 33, 35-39, 47, 49, 57, 58, 69, 74, 76, 92-107), which aligns with previous reviews (2). Although problem drinking exists on a continuum from mild to more severe, various studies tended to focus on one point in the problem severity continuum (e.g. heavy drinking, HED/BD or alcohol abuse, alcohol dependence, and AUD) and measures these forms of problem drinking with diverse measurement tools like quantity/frequency questions, risky single occasion drinking criteria, screening tools, or structured diagnostic interviews (33-40, 46, 49-63, 69-77, 80, 81, 89-91, 94, 97-99, 102, 108-123, 129, 131). These tools also were variable in the timeframe used to assess problem drinking, with the assessment period ranging from days, weeks, months, or years among the studies included in this review (33-40, 62, 63, 94, 97-99, 109, 129, 131).

For peer review only

For peer review only - http://bmjopen.bmj.com/site/about/guidelines.xhtml

This variability in how alcohol use and various forms of problem drinking are defined and measured is a significant weakness in the literature, with previous studies noting a lack of attention to the validity of alcohol screening tools and questionnaires (132). Many challenges in understanding the true prevalence of problem drinking arise from different definitions and inconsistent approaches to measuring it (2). This was evident in the current review, where we noted considerable differences in the prevalence estimates for problem drinking, partly due to variability in how problem drinking was conceptualised and measured. It is crucial to have a uniform and precise definition of problem drinking that can be applied across studies. This approach will allow for a more accurate estimation of prevalence and more effective identification of people with problem drinking, and it will enhance the robustness of the evidence base on which to advocate for alcohol harm reduction.

Harmonized measures and consensus on the best ways of measuring alcohol use and problem drinking would aid with comparative studies of problem drinking prevalence. Despite the difficulties and challenges associated with building consensus on the best measures for assessing problem drinking and various indicators of problem drinking development, there is an increasing interest in developing agreement on this topic (133). Notably, even if consensus is reached on which measures of problem drinking to use, these self-report measures would be subject to reporting bias, specifically under-reporting or over-reporting of alcohol consumption. These self-report measures can be supplemented with objective measures of alcohol use (alcohol biomarkers) such as Phosphatidylethanol (PEth) (134-139). There is emerging evidence of the benefits of incorporating self-report alcohol use measures with alcohol biomarkers like PEth for valid assessment of problem drinking (137-150).

Problem drinking is affected by numerous factors at population and individual levels, and identifying these factors is important for informing the design of harm minimization interventions (28). The factors associated with problem drinking from our review, summarized as socio-demographic and economic characteristics (age, sex, relationship status, education, employment, income level, religion, race, location, and alcohol outlet density), clinical factors (medical problems, mental disorders, substance use), and quality of life fit into the biopsychosocial model used in medicine, psychiatry, and psychology to

For peer review only

understand health and illness (151, 152). This review identified heterogeneity in the kinds of factors that were investigated by included studies as well as the measures used to assess these exposures. This likely contributed to the inconsistent associations found between these factors and the risk of problem drinking.

In addition, it is important to note that this review has weaknesses concerning the examination of factors associated with problem drinking, including the use of less powerful statistical tests (nonparametric tests) or no use of statistical tests (36, 37, 47, 48, 50, 53, 89, 100, 111, 115, 126, 153), only a few variables were modeled to control confounding (71, 77, 91, 97, 112, 113, 125, 127), use of non-validated tools that could result in measurement errors (33, 35, 36, 49, 81, 95, 105, 119, 129), sampling only (predominantly) males or females that could cause selection bias (55, 63, 75, 113, 121, 129), high attrition rates (40, 75, 130), and small sample sizes (58, 63, 90, 109, 110).

This review highlights the need for additional research on factors associated with problem drinking. Prospective cohort studies that address these methodological limitations and examine the correlates and consequences of problem drinking are needed to guide the design of alcohol harm minimization interventions. The inconsistency reported in the current scoping review requires a united effort among researchers to refine alcohol use assessment methods to make them clearer and systematize definitions. Hence, future studies could focus on contextual adaptation of WHO-recommended tools like the AUDIT or its shortened versions. Addressing the challenges associated with measuring and defining problem drinking would improve the validity and reliability of future studies, enhance our understanding of the nature and extent of problematic alcohol use, and provide evidence to inform interventions to minimize alcohol-related harms.

Strengths and limitations

Our scoping review has several strengths. The review protocol was registered at Open Science Framework (OSF), and we followed PRISMA-ScR guidelines in our scoping review. A comprehensive search strategy was employed to locate global studies. We decided to critically appraise the quality of the included studies, though it is not mandatory in the scoping reviews. This scoping review has several limitations. First, to make our review more feasible, we included only community-based studies, and studies conducted at institutions like hospitals, primary health care services, addiction centers, and

For peer review only

For peer review only - http://bmjopen.bmj.com/site/about/guidelines.xhtml

colleges/universities were not included, so findings may not be generalizable to these settings. Second, this review was limited to articles published in English. Accordingly, publication bias is possible as studies conducted in other languages and unpublished reports on alcohol use would not have been included.

CONCLUSIONS

This review highlights heterogeneity in ways in which problem drinking and associated factors have been conceptualized and measured. It also identified methodological weaknesses across the included studies. Together, these findings limit our confidence in the prevalence estimates for problem drinking, our ability to compare findings across studies, and pool data for pooled prevalence estimates. Due to the community-based and cross-sectional nature of the included studies, this review does not provide data on alcohol-related harms. Future alcohol-related research could improve the quality and reliability of findings by strictly following a priori proposed methods and protocols, using validated tools for assessing problem drinking, applying appropriate statistical tests, controlling for possible confounders, minimizing selection bias, and using a sufficiently large and justifiable sample size.

Abbreviations

AA: Alcohol abuse; AD: Alcohol dependence; ASSIST: Alcohol, Smoking, and Substance Involvement Screening Test; AUD: Alcohol use disorder; AUDIT: Alcohol Use Disorder Identification Test; BD: Binge drinking; CIDI: Composite International Diagnostic Interview; DSM-III/DSM-III-R: The Diagnostic and Statistical Manual of Mental Disorders, Third Edition; DSM-IV/DSM-5: Diagnostic and Statistical Manual of Mental Disorders 4th and 5th edition; HD: Heavyy drinking; HED: Heavy episodic drinking; HED/BD: heavy episodic or binge drinking; HICs: High-Income countries; ICD: The International Classification of Diseases; LMICs: Low-and Middle-Income countries; MINI: Mini International Neuropsychiatric Interview; MRC: Medical Research Council; PD: Problem drinking; PHC: Primary Health Care; PRISMA-ScR: Preferred Reporting Items for Systematic reviews and Meta-Analyses extension for Scoping Reviews; QF: quantity/frequency questionnaires; RSOD: risky single occasion drinking criteria; SCID: Structured Clinical Interview for DSM Disorders; WHO: World Health Organization

Acknowledgments

For peer review only

For peer review only - http://bmjopen.bmj.com/site/about/guidelines.xhtml

Our appreciation is dedicated to AMARI (African Mental heAlth Research Initiative) and Addis Ababa University (AAU) for providing training to Kefyalew Dagne in "Systematic Review & Meta-Analysis." The authors would like to acknowledge the Ethiopian Public Health Association (EPHA) Annual Scientific Conference for providing the opportunity to present this research at their 34th (2023) conference.

Author Contributions

KD was involved in the project's conceptualization, writing the protocol, developing a search strategy, searching, screening, and extracting included articles, synthesising the results, writing the discussion section of the manuscript, and harmonizing the entire document. ST approved the conceptualized research project, the protocol, and the draft manuscript. BM reviewed the search strategy and provided in-depth reviews of the manuscript. AM was involved in screening and extracting included articles. All authors involved read and approved the final manuscript.

Funding

Kefylew Dagne was supported through AMARI, funded through the DELTAS Africa Initiative (DEL-15-01). The DELTAS Africa Initiative is an independent funding scheme of the African Academy of Sciences (AAS)'s Alliance for Accelerating Excellence in Science in Africa (AESA) and supported by the New Partnership for Africa's Development Planning and Coordinating Agency (NEPAD Agency) with funding from the Wellcome Trust (DEL-15-01) and the UK government. The views expressed in this publication are those of the author (s) and not necessarily those of AAS, NEPAD Agency, Wellcome Trust, or the UK government.

Availability of data and materials

All relevant materials and data supporting the results of this study are contained within the manuscript, and relevant documents will be available upon request.

Ethics approval and consent to participate

Not applicable.

Consent for publication

Not applicable.

Competing interests

The authors declare that they have no competing interests.

For peer review only

Author Details

1 Department of Psychiatry, School of Medicine, College of Health Sciences, Addis Ababa University, Addis Ababa, Ethiopia

2 Department of Psychiatry, College of Health Sciences and Medicine, Debre Berhan University, Debre Berhan, Ethiopia

3 Curtin enAble Institute, Faculty of Health Sciences, Curtin University, Kent Street, Bentley, WA 6102, Australia

4 Alcohol, Tobacco and Other Drug Research Unit, South African Medical Research Council, Cape Town, South Africa

5 Division of Addiction Psychiatry, Department of Psychiatry and Mental Health, University of Cape Town, Cape Town, South Africa

Figure Legends

Figure 1: PRISMA flow diagram of included studies in the scoping review, 2023.

References

1. Toner P, Böhnke JR, Andersen P, McCambridge J. Alcohol screening and assessment measures for young people: A systematic review and meta-analysis of validation studies. Drug and alcohol dependence. 2019;202:39-49.
2. Griswold MG, Fullman N, Hawley C, Arian N, Zimsen SR, Tymeson HD, et al. Alcohol use and burden for 195 countries and territories, 1990–2016: a systematic analysis for the Global Burden of Disease Study 2016. The Lancet. 2018;392(10152):1015-35.
3. World Health Organization. Global status report on alcohol and health 2018: World Health Organization; 2019.
4. World Health Organization. Management of Substance Abuse Unit. Global status report on alcohol and health, 2014: World Health Organization; 2014.
5. Corrao G, Bagnardi V, Zambon A, La Vecchia C. A meta-analysis of alcohol consumption and the risk of 15 diseases. Preventive medicine. 2004;38(5):613-9.
6. Low WY, Lee YK, Samy AL. Non-communicable diseases in the Asia-Pacific region: Prevalence, risk factors and community-based prevention. Int J Occup Med Environ Health. 2015;28(1):20-6.

For peer review only

7. HELZER JE, PRZYBECK TR. The co–occurrence of alcoholism with other psychiatric disorders in the general population and its impact on treatment Journal of Studies on Alcohol 1988;49:219–24.
8. Fone D, Dunstan F, White J, Webster C, Rodgers S, Lee S, et al. Change in alcohol outlet density and alcohol-related harm to population health (CHALICE). BMC public health. 2012;12:428.
9. Lester L, Baker R, Coupland C, Orton E. Alcohol misuse and injury outcomes in young people aged 10–24. Journal of Adolescent Health. 2018;62(4):450-6.
10. Newbury-Birch D, Ferguson J, Connor N, Divers A, Waller G. A rapid systematic review of worldwide alcohol use disorders and brief alcohol interventions in the criminal justice system. Frontiers in psychiatry. 2022;13.
11. Hagman BT, Falk D, Litten R, Koob GF. Defining recovery from alcohol use disorder: development of an NIAAA research definition. American Journal of Psychiatry. 2022;179(11):807-13.
12. Enoch M-A, Goldman D. Problem drinking and alcoholism: diagnosis and treatment. American family physician. 2002;65(3):441-9.
13. Van Oers J, Bongers I, Van de Goor L, Garretsen H. Alcohol consumption, alcohol-related problems, problem drinking, and socioeconomic status. Alcohol and alcoholism (Oxford, Oxfordshire). 1999;34(1):78-88.
14. Mosel S. Problem Drinking vs. Alcoholism [updated 25 October 2022. Available from: https://alcohol.org/alcoholism/or-is-it-just-a-problem/.
15. American Addiction Centers. Problem Drinker Defined [updated October 9, 2020. Available from: https://alcoholrehab.com/alcoholism/signs-of-alcoholism/problem-drinker-defined/.
16. HRB National Drugs Library. Research Glossary [Available from: https://www.drugsandalcohol.ie/glossary/ Accessed 6 July 2023.
17. Saunders JB, Aasland OG, Babor TF, De La Fuente JR, Grant M. Development of the alcohol use disorders identification test (AUDIT): WHO collaborative project on early detection of persons with harmful alcohol consumption-II. Addiction (Abingdon, England). 1993;88(6):791-804.
18. Edwards G, Arif A, Hadgson R. Nomenclature and classification of drug-and alcohol-related problems: a WHO Memorandum. Bulletin of the World Health Organization. 1981;59(2):225-42.
19. Organization WH. The ICD-10 classification of mental and behavioural disorders: clinical descriptions and diagnostic guidelines: World Health Organization; 1992.
20. Babor TF, de la Fuente JR, Saunders J, Grant M. The Alcohol Use Disorders Identification Test: Guidelines for use in. Primary care. 2001.
21. Fairbairn N, Wood E, Dobrer S, Dong H, Kerr T, Debeck K. The relationship between hazardous alcohol use and violence among street-involved youth. The American journal on addictions. 2017;26(8):852-8.
22. Reid MC, Fiellin DA, O'Connor PG. Hazardous and harmful alcohol consumption in primary care. Archives of internal medicine. 1999;159(15):1681-9.
23. Tsai J, Ford ES, Li C, Pearson WS, Zhao G. Binge drinking and suboptimal self-rated health among adult drinkers. Alcoholism, clinical and experimental research. 2010;34(8):1465-71.
24. Weiser SD, Leiter K, Heisler M, McFarland W, Percy-de Korte F, DeMonner SM, et al. A population-based study on alcohol and high-risk sexual behaviors in Botswana. PLoS medicine. 2006;3(10):e392.
25. American Psychiatric Association. Diagnostic and statistical manual of mental disorders: DSM-IV: American psychiatric association Washington, DC; 1994.
26. American Psychiatric Association. Diagnostic and statistical manual of mental disorders: DSM-5. Fifth ed: Arlington, VA, American Psychiatric Association; 2013.
27. Iranpour A, Nakhaee N. A review of alcohol-related harms: a recent update. Addiction & health. 2019;11(2):129.

For peer review only

For peer review only - http://bmjopen.bmj.com/site/about/guidelines.xhtml

28. Park SH, Kim DJ. Global and regional impacts of alcohol use on public health: Emphasis on alcohol policies. Clinical and molecular hepatology. 2020;26(4):652.
29. Tricco AC, Lillie E, Zarin W, O'Brien KK, Colquhoun H, Levac D, et al. PRISMA extension for scoping reviews (PRISMA-ScR): checklist and explanation. Annals of internal medicine. 2018;169(7):467-73.
30. Ribeiro CM, Beserra BTS, Silva NG, Lima CL, Rocha PRS, Coelho MS, et al. Exposure to endocrine-disrupting chemicals and anthropometric measures of obesity: A systematic review and meta-analysis. BMJ open. 2020;10(6):e033509.
31. Herzog R, Álvarez-Pasquin M, Díaz C, Del Barrio JL, Estrada JM, Gil Á. Are healthcare workers' intentions to vaccinate related to their knowledge, beliefs and attitudes? A systematic review. BMC public health. 2013;13(1):1-17.
32. Dubey VP, Kievišienė J, Rauckiene-Michealsson A, Norkiene S, Razbadauskas A, Agostinis-Sobrinho C. Bullying and Health Related Quality of Life among Adolescents—A Systematic Review. Children. 2022;9(6):766.
33. Bataille V, Ruidavets JB, Arveiler D, Amouyel P, Ducimetiere P, Perret B, et al. Joint use of clinical parameters, biological markers and cage questionnaire for the identification of heavy drinkers in a large population-based sample. Alcohol and Alcoholism. 2003;38(2):121-7.
34. Coulson CE, Williams LJ, Henry MJ, Berk M, Lubman DI, Brennan SL, et al. Patterns of alcohol use and associated physical and lifestyle characteristics according to new Australian guidelines. The Australian and New Zealand journal of psychiatry. 2010;44(10):946-51.
35. Geels LM, Vink JM, van Beek JH, Bartels M, Willemsen G, Boomsma DI. Increases in alcohol consumption in women and elderly groups: evidence from an epidemiological study. BMC public health. 2013;13:207.
36. Williamson RJ, Sham P, Ball D. Binge drinking trends in a UK community-based sample. Journal of Substance Use. 2003;8(4):234-7.
37. O'Dwyer C, Mongan D, Millar SR, Rackard M, Galvin B, Long J, et al. Drinking patterns and the distribution of alcohol-related harms in Ireland: evidence for the prevention paradox. BMC public health. 2019;19(1):1323.
38. Mohler-Kuo M, Foster S, Gmel G, Dey M, Dermota P. DSM-IV and DSM-5 alcohol use disorder among young Swiss men. Addiction (Abingdon, England). 2015;110(3):429-40.
39. Andrews-Chavez JY, Lee CS, Houser RF, Falcon LM, Tucker KL. Factors associated with alcohol consumption patterns in a Puerto Rican urban cohort. Public health nutrition. 2015;18(3):464-73.
40. Auchincloss AH, Niamatullah S, Adams M, Melly SJ, Li J, Lazo M. Alcohol outlets and alcohol consumption in changing environments: prevalence and changes over time. Substance abuse treatment, prevention, and policy. 2022;17(1):7.
41. Ewing J, Rose B. Identifying the Hidden Alcoholic [w:] Paper read at the 29th International Congress on Alcoholism and Drug Dependence. Australia Butterworth, Sydney. 1970.
42. Ewing JA. Detecting alcoholism: the CAGE questionnaire. Jama. 1984;252(14):1905-7.
43. Mayfield D, McLeod G, Hall P. The CAGE questionnaire: validation of a new alcoholism screening instrument. American journal of psychiatry. 1974;131(10):1121-3.
44. Selzer ML. The Michigan Alcoholism Screening Test: The quest for a new diagnostic instrument. American journal of psychiatry. 1971;127(12):1653-8.
45. Pokorny AD, Miller BA, Kaplan HB. The brief MAST: A shortened version of the Michigan Alcoholism Screening Test. American journal of psychiatry. 1972;129(3):342-5.
46. Group WAW. The alcohol, smoking and substance involvement screening test (ASSIST): development, reliability and feasibility. Addiction (Abingdon, England). 2002;97(9):1183-94.

For peer review only

47. Aalto M, Seppa K, Kiianmaa K, Sillanaukee P. Drinking habits and prevalence of heavy drinking among primary health care outpatients and general population. Addiction (Abingdon, England). 1999;94(9):1371-9.
48. Aira M, Hartikainen S, Sulkava R. Community prevalence of alcohol use and concomitant use of medication - A source of possible risk in the elderly aged 75 and older? International journal of geriatric psychiatry. 2005;20(7):680-5.
49. Moreira LB, Fuchs FD, Moraes RS, Bredemeier M, Cardozo S, Fuchs SC, et al. Alcoholic beverage consumption and associated factors in Porto Alegre, a southern Brazilian city: a population-based survey. Journal of studies on alcohol. 1996;57(3):253-9.
50. Alem A, Kebede D, Kullgren G. The epidemiology of problem drinking in Butajira, Ethiopia. Acta psychiatrica Scandinavica Supplementum. 1999;397:77-83.
51. Kebede D, Alem A. The epidemiology of alcohol dependence and problem drinking in Addis Ababa, Ethiopia. Acta psychiatrica Scandinavica Supplementum. 1999;397:30-4.
52. Moreira LB, Fuchs FD, Moraes RS, Bredemeier M, Duncan BB. Alcohol intake and blood pressure: The importance of time elapsed since last drink. Journal of hypertension. 1998;16(2):175-80.
53. Foulds J, Wells JE, Lacey C, Adamson S, Mulder R. Harmful drinking and talking about alcohol in primary care: New Zealand population survey findings. Acta psychiatrica Scandinavica. 2012;126(6):434-9.
54. Britton A, Fat LN, Neligan A. The association between alcohol consumption and sleep disorders among older people in the general population. Scientific reports. 2020;10(1):5275.
55. Husberg VH, Hopstock LA, Friborg O, Rosenvinge JH, Bergvik S, Rognmo K. Epidemiology of comorbid hazardous alcohol use and insomnia in 19 185 women and men attending the population-based Tromso Study 2015-2016. BMC public health. 2022;22(1):844.
56. Lindstrom J, Hellstrom C, Simonsson B, Molarius A. Alcohol consumption and self-rated health among older people: population-based study in Sweden. Journal of public health (Oxford, England). 2020;42(4):756-65.
57. Peltzer K, Ramlagan S, Satekge M. Alcohol use, problem drinking and health risk factors among South African youths. Journal of Psychology in Africa. 2012;22(4):671-6.
58. Yeung W, Leong W-Y, Khoun K, Ong W, Sambi S, Lim S-M, et al. Alcohol use disorder and heavy episodic drinking in rural communities in cambodia: Risk factors and community-perceived strategies. Asia-Pacific Journal of Public Health. 2015;27(8):835-47.
59. Teferra S, Medhin G, Selamu M, Bhana A, Hanlon C, Fekadu A. Hazardous alcohol use and associated factors in a rural Ethiopian district: a cross-sectional community survey. BMC public health. 2016;16:218.
60. Jadnanansing R, Blankers M, Dwarkasing R, Etwaroo K, Lumsden V, Dekker J, et al. Prevalence of substance use disorders in an urban and a rural area in Suriname. Trop Med Health. 2021;49(1):12.
61. Pengpid S, Peltzer K, Ramlagan S. Prevalence and correlates of hazardous, harmful or dependent alcohol use and drug use amongst persons 15 years and older in South Africa: Results of a national survey in 2017. African journal of primary health care & family medicine. 2021;13(1):e1-e8.
62. Bete T, Lami M, Negash A, Eyeberu A, Birhanu A, Berhanu B, et al. Current alcohol, tobacco, and khat use and associated factors among adults living in Harari regional state, eastern Ethiopia: A community-based cross-sectional study. Frontiers in psychiatry. 2022;13:955371.
63. Wolde A. Alcohol Use Disorder and Associated Factors Among Elderly in Ethiopia. Substance Abuse: Research and Treatment. 2023;17.
64. Organization WH, Organization WH. CIDI-interview (version 1.0),(b) CIDI-user manual,(c) CIDI-training manual,(d) CIDI-computer programs. Geneva: World Health Organization.

For peer review only

For peer review only - http://bmjopen.bmj.com/site/about/guidelines.xhtml

65. Robins L, Wittchen H, Wing J, Sartorius N, Pull C, Towle L, et al. The composite international diagnostic interview (CIDI) reliability and applicability in different countries. Psychiatry: A world perspective. 1990;1:118-24.
66. Robins LN, Wing J, Wittchen HU, Helzer JE, Babor TF, Burke J, et al. The Composite International Diagnostic Interview: an epidemiologic instrument suitable for use in conjunction with different diagnostic systems and in different cultures. Archives of general psychiatry. 1988;45(12):1069-77.
67. Wittchen H-U, Robins LN, Cottler LB, Sartorius N, Burke JD, Regier D. Cross-cultural feasibility, reliability and sources of variance of the Composite International Diagnostic Interview (CIDI). The British Journal of Psychiatry. 1991;159(5):645-53.
68. Kessler RC, Üstün TB. The world mental health (WMH) survey initiative version of the world health organization (WHO) composite international diagnostic interview (CIDI). International journal of methods in psychiatric research. 2004;13(2):93-121.
69. Kim JH, Lee S, Chow J, Lau J, Tsang A, Choi J, et al. Prevalence and the factors associated with binge drinking, alcohol abuse, and alcohol dependence: a population-based study of Chinese adults in Hong Kong. Alcohol and alcoholism (Oxford, Oxfordshire). 2008;43(3):360-70.
70. Meyer C, Rumpf HJ, Hapke U, Dilling H, John U. Prevalence of alcohol consumption, abuse and dependence in a country with high per capita consumption: findings from the German TACOS study. Transitions in Alcohol Consumption and Smoking. Social psychiatry and psychiatric epidemiology. 2000;35(12):539-47.
71. Bott K, Meyer C, Rumpf H-J, Hapke U, John U. Psychiatric Disorders among At-Risk Consumers of Alcohol in the General Population. Journal of studies on alcohol. 2005;66(2):246-53.
72. Neumark YD, Lopez-Quintero C, Grinshpoon A, Levinson D. Alcohol drinking patterns and prevalence of alcohol-abuse and dependence in the Israel National Health Survey. The Israel journal of psychiatry and related sciences. 2007;44(2):126-35.
73. Proodfoot H, Teeson M. Who seeks treatment for alcohol dependence? Findings form the Australian National Survey of Mental Health and Wellbeing. Social psychiatry and psychiatric epidemiology. 2002;37(10):451-6.
74. Veerbeek MA, Ten Have M, van Dorsselaer SA, Oude Voshaar RC, Rhebergen D, Willemse BM. Differences in alcohol use between younger and older people: Results from a general population study. Drug and alcohol dependence. 2019;202:18-23.
75. Lundin A, Waern M, Love J, Lovestad S, Hensing G, Danielsson AK. Towards ICD-11 for alcohol dependence: Diagnostic agreement with ICD-10, DSM-5, DSM-IV, DSM-III-R and DSM-III diagnoses in a Swedish general population of women. Drug and alcohol dependence. 2021;227:108925.
76. Chou KL, Liang K, Mackenzie CS. Binge drinking and axis I psychiatric disorders in community-dwelling middle-aged and older adults: Results from the National Epidemiologic Survey on Alcohol and Related Conditions (NESARC). Journal of Clinical Psychiatry. 2011;72(5):640-7.
77. Latvala A, Tuulio-Henriksson A, Perala J, Saarni SI, Aalto-Setala T, Aro H, et al. Prevalence and correlates of alcohol and other substance use disorders in young adulthood: A population-based study. BMC psychiatry. 2009;9:73.
78. WHO. The ICD-10 classification of mental and behavioural disorders: clinical descriptions and diagnostic guidelines: World Health Organization; 1992.
79. Reed GM, First MB, Kogan CS, Hyman SE, Gureje O, Gaebel W, et al. Innovations and changes in the ICD-11 classification of mental, behavioural and neurodevelopmental disorders. World psychiatry. 2019;18(1):3-19.
80. Zavos HM, Siribaddana S, Ball HA, Lynskey MT, Sumathipala A, Rijsdijk FV, et al. The prevalence and correlates of alcohol use and alcohol use disorders: a population based study in Colombo, Sri Lanka. BMC psychiatry. 2015;15:158.

For peer review only

81. Andersson LMC, Twum-Antwi A, Staland-Nyman C, van Rooyen D. Prevalence and socioeconomic characteristics of alcohol disorders among men and women in the Eastern Cape Province, South Africa. Health & social care in the community. 2018;26(1):e143-e53.
82. Grant BF, Dawson DA, Hasin DS. The alcohol use disorder and associated disabilities interview schedule-DSM-IV version. Bethesda, MD: National Institute on Alcohol Abuse and Alcoholism. 2001.
83. Robins LN, Helzer JE, Croughan J, Ratcliff KS. National Institute of Mental Health diagnostic interview schedule: Its history, characteristics, and validity. Archives of general psychiatry. 1981;38(4):381-9.
84. Spitzer RL, Williams JB, Gibbon M, First MB. The structured clinical interview for DSM-III-R (SCID): I: history, rationale, and description. Archives of general psychiatry. 1992;49(8):624-9.
85. First MB, Spitzer RL, Gibbon M, Williams JB. Computer-assisted SCID-Clinician version. 1997.
86. Sheehan D, Janavs J, Baker R, Harnett-Sheehan K, Knapp E, Sheehan M. Mini international neuropsychiatric interview. Tampa: University of South Florida. 1994.
87. Sheehan D, Lecrubier Y, Sheehan KH, Janavs J, Weiller E, Keskiner A, et al. The validity of the Mini International Neuropsychiatric Interview (MINI) according to the SCID-P and its reliability. European psychiatry. 1997;12(5):232-41.
88. Sheehan DV, Lecrubier Y, Sheehan KH, Amorim P, Janavs J, Weiller E, et al. The Mini-International Neuropsychiatric Interview (MINI): the development and validation of a structured diagnostic psychiatric interview for DSM-IV and ICD-10. Journal of clinical psychiatry. 1998;59(20):22-33.
89. Mondi CF, Giovanelli A, Ou SR, Reynolds AJ. Psychiatric and substance use disorders in a predominately low-income, black sample in early midlife. Journal of psychiatric research. 2022;148:332-9.
90. Abd Rashid RB, Mohd Daud MNB, Guad RM, Gan SH, Wan Husin W, Giloi N, et al. Prevalence and risk factors associated with alcohol consumption among indigenous people in Sabah Borneo Island. The Australian journal of rural health. 2021;29(3):464-72.
91. Assanangkornchai S, Nontarak J, Aekplakorn W, Chariyalertsak S, Kessomboon P, Taneepanichskul S. Socio-economic inequalities in the association between alcohol use disorder and depressive disorder among Thai adults: a population-based study. BMC psychiatry. 2020;20(1):553.
92. Ding L, Liang Y, Tan ECK, Hu Y, Zhang C, Liu Y, et al. Smoking, heavy drinking, physical inactivity, and obesity among middle-aged and older adults in China: cross-sectional findings from the baseline survey of CHARLS 2011-2012. BMC public health. 2020;20(1):1062.
93. Dias da Costa JS, Silveira MF, Gazalle FK, Oliveira SS, Hallal PC, Menezes AMB, et al. Heavy alcohol consumptions and associated factors: A population-based study. Revista de saude publica. 2004;38(2):284-91.
94. Peltzer K, Phaswana-Mafuya N. Problem drinking and associated factors in older adults in South Africa. African journal of psychiatry. 2013;16(2):104-9.
95. Janghorbani M, Ho SY, Lam TH, Janus ED. Prevalence and correlates of alcohol use: A populationbased study in Hong Kong. Addiction (Abingdon, England). 2003;98(2):215-24.
96. Miller JW, Gfroerer JC, Brewer RD, Naimi TS, Mokdad A, Giles WH. Prevalence of adult binge drinking: a comparison of two national surveys. American journal of preventive medicine. 2004;27(3):197-204.
97. Hernandez-Vasquez A, Chacon-Torrico H, Vargas-Fernandez R, Grendas LN, Bendezu-Quispe G. Gender Differences in the Factors Associated with Alcohol Binge Drinking: A Population-Based Analysis in a Latin American Country. International journal of environmental research and public health. 2022;19(9):4931.
98. Oancea SC, de Oliveira GD, Sukumaran P, Vogeltanz-Holm N, Nucci LB. The association between alcohol consumption and self-reported current depression among adults residing in Brazil. Journal of public health (Oxford, England). 2021;43(2):e204-e12.

For peer review only

99. Prais HAC, De Loyola Filho AI, Firmo JOA, Lima-Costa MF, Uchoa E. A population-based study on binge drinking among elderly Brazilian men: Evidence from the Belo Horizonte and Bambui health surveys. Revista Brasileira de Psiquiatria. 2008;30(2):118-23.
100. Shockey TM, Esser MB. Binge Drinking by Occupation Groups among Currently Employed U.S. Adults in 32 States, 2013-2016. Substance use & misuse. 2020;55(12):1968-79.
101. Lee YY, Wang P, Abdin E, Chang S, Shafie S, Sambasivam R, et al. Prevalence of binge drinking and its association with mental health conditions and quality of life in Singapore. Addictive behaviors. 2020;100:106114.
102. Peltzer K, Davids A, Njuho P. Alcohol use and problem drinking in South Africa: findings from a national population-based survey. African journal of psychiatry. 2011;14(1):30-7.
103. Pillai A, Nayak MB, Greenfield TK, Bond JC, Nadkarni A, Patel V. Patterns of alcohol use, their correlates, and impact in male drinkers: a population-based survey from Goa, India. Social psychiatry and psychiatric epidemiology. 2013;48(2):275-82.
104. Vellios N, Van Walbeek C. Self-reported alcohol use and binge drinking in South Africa: Evidence from the National Income Dynamics Study, 2014-2015. South African Medical Journal. 2018;108(1):33-9.
105. Dahal S, Sah RB, Niraula SR, Karkee R, Chakravartty A. Prevalence and determinants of noncommunicable disease risk factors among adult population of Kathmandu. PloS one. 2021;16(9 September):e0257037.
106. Getachew T, Defar A, Teklie H, Gonfa G, Bekele A, Bekele A, et al. Magnitude and predictors of excessive alcohol use in Ethiopia: findings from the 2015 national non-communicable diseases STEPS survey. Ethiopian Journal of Health Development. 2017;31(1):312-9.
107. Gutema BT, Chuka A, Ayele G, Tariku EZ, Aschalew Z, Baharu A, et al. Prevalence of heavy episodic drinking and associated factors among adults residing in Arba Minch health and demographic surveillance site: a cross sectional study. BMC public health. 2020;20(1):1895.
108. Mendoza-Sassi RA, Beria JU. Prevalence of alcohol use disorders and associated factors: a population-based study using AUDIT in southern Brazil. Addiction (Abingdon, England). 2003;98(6):799-804.
109. Castelo Branco FMF, de Vargas D. Alcohol use patterns and associated variables among the Karipuna indigenous people in the extreme Northern Brazilian Amazon. Journal of ethnicity in substance abuse. 2023;22(1):29-44.
110. Sau A. AUDIT (Alcohol use disorders identification test) to estimate the pattern and correlates of alcohol consumption among the adult population of west bengal, india: A community based cross sectional study. Journal of Clinical and Diagnostic Research. 2017;11(4):LC01-LC4.
111. Jonas JB, Nangia V, Rietschel M, Paul T, Behere P, Panda-Jonas S. Prevalence of depression, suicidal ideation, alcohol intake and nicotine consumption in rural central India. The Central India Eye and Medical Study. PloS one. 2014;9(11).
112. Olickal JJ, Saya GK, Selvaraj R, Chinnakali P. Association of alcohol use with quality of life (QoL): A community based study from Puducherry, India. Clinical Epidemiology and Global Health. 2021;10:100697.
113. Olickal JJ, Selvaraj R, Saya GK, Chinnakali P. Alcohol use among adult men and its impact on personal and family life: findings of a community-based cross-sectional survey and focus groups discussions from a low alcohol taxed region, Puducherry, India. Family practice. 2022;39(2):316-22.
114. Takahashi R, Wilunda C, Magutah K, Mwaura-Tenambergen W, Wilunda B, Perngparn U. Correlates of alcohol consumption in rural western Kenya: A cross-sectional study. BMC psychiatry. 2017;17(1):175.
115. Nalwadda O, Rathod SD, Nakku J, Lund C, Prince M, Kigozi F. Alcohol use in a rural district in Uganda: findings from community-based and facility-based cross-sectional studies. International journal of mental health systems. 2018;12:12.

For peer review only

116. Rathod SD, Luitel NP, Jordans MJD. Prevalence and correlates of alcohol use in a central Nepal district: secondary analysis of a population-based cross-sectional study. Global mental health (Cambridge, England). 2018;5:e37.
117. Zewdu S, Hanlon C, Fekadu A, Medhin G, Teferra S. Treatment gap, help-seeking, stigma and magnitude of alcohol use disorder in rural Ethiopia. Substance abuse treatment, prevention, and policy. 2019;14(1):4.
118. Endashaw Hareru H, Kaso AW, Debela BG, Abebe L, Sisay WTD, Kassa Abebe R, et al. Alcohol use disorder and its associated factors among residents in Southern Ethiopia during the era of COVID-19. SAGE open medicine. 2022;10:20503121221105031.
119. Legas G, Asnakew S, Belete A, Beyene GM, Wubet GM, Bayih WA, et al. Magnitude and correlates of alcohol use disorder in south Gondar zone, northwest Ethiopia: A community based cross-sectional study. PloS one. 2021;16(9):e0257804.
120. Jirapramukpitak T, Prince M, Harpham T. Rural-urban migration, illicit drug use and hazardous/harmful drinking in the young Thai population. Addiction (Abingdon, England). 2008;103(1):91-100.
121. Wainberg M, Oquendo MA, Peratikos MB, Gonzalez-Calvo L, Pinsky I, Duarte CS, et al. Hazardous alcohol use among female heads-of-household in rural Mozambique. Alcohol (Fayetteville, NY). 2018;73:37-44.
122. Tomkins S, Saburova L, Kiryanov N, Andreev E, McKee M, Shkolnikov V, et al. Prevalence and socio-economic distribution of hazardous patterns of alcohol drinking: study of alcohol consumption in men aged 25-54 years in Izhevsk, Russia. Addiction (Abingdon, England). 2007;102(4):544-53.
123. Ji A, Lou P, Dong Z, Xu C, Zhang P, Chang G, et al. The prevalence of alcohol dependence and its association with hypertension: a population-based cross-sectional study4 in Xuzhou city, China. BMC public health. 2018;18(1):364.
124. Trangenstein PJ, Morojele NK, Lombard C, Jernigan DH, Parry CD. Heavy drinking and contextual risk factors among adults in South Africa: findings from the International Alcohol Control study. Substance abuse treatment, prevention, and policy. 2018;13(1):43.
125. Mason-Jones AJ, Cabieses B. Alcohol, binge drinking and associated mental health problems in young urban Chileans. PloS one. 2015;10(4).
126. Aremu T, Anibijuwon IB, John-Akinola YO, Oluwasanu M, Oladepo O. Prevalence and Factors Associated With Alcohol Use in Selected Urban Communities in Ibadan, Nigeria. International quarterly of community health education. 2021:272684x211006515.
127. Bonnechère B, Samadoulougou S, Cisse K, Tassembedo S, Kouanda S, Kirakoya-Samadoulougou F. Alcohol consumption and associated risk factors in Burkina Faso: results of a population-based cross-sectional survey. BMJ open. 2022;12(2):e058005.
128. Lo TQ, Oeltmann JE, Odhiambo FO, Beynon C, Pevzner E, Cain KP, et al. Alcohol use, drunkenness and tobacco smoking in rural western Kenya. Tropical medicine & international health : TM & IH. 2013;18(4):506-15.
129. Tegegne KD, Boke MM, Lakew AZ, Gebeyehu NA, Kassaw MW. Alcohol and khat dual use among male adults in Ethiopia: A multilevel multinomial analysis. PloS one. 2023;18(9):e0290415.
130. Burazeri G, Kark JD. Prevalence and determinants of binge drinking in middle age in a transitional post-communist country: a population-based study in Tirana, Albania. Alcohol and alcoholism (Oxford, Oxfordshire). 2010;45(2):180-7.
131. Rezaei N, Ahmadi N, Shams Beyranvand M, Hasan M, Gohari K, Yoosefi M, et al. Alcohol consumption and related disorders in Iran: Results from the National Surveillance of Non-Communicable Diseases' Survey (STEPs) 2016. PLOS global public health. 2022;2(11):e0000107.
132. Midanik L. The validity of self-reported alcohol consumption and alcohol problems: a literature review. British journal of addiction. 1982;77(4):357-82.

For peer review only

For peer review only - http://bmjopen.bmj.com/site/about/guidelines.xhtml

133. Hussong AM, Gottfredson NC, Bauer DJ, Curran PJ, Haroon M, Chandler R, et al. Approaches for creating comparable measures of alcohol use symptoms: Harmonization with eight studies of criminal justice populations. Drug and alcohol dependence. 2019;194:59-68.
134. Litten RZ, Bradley AM, Moss HB. Alcohol biomarkers in applied settings: recent advances and future research opportunities. Alcoholism: Clinical and Experimental Research. 2010;34(6):955-67.
135. Nanau RM, Neuman MG. Biomolecules and biomarkers used in diagnosis of alcohol drinking and in monitoring therapeutic interventions. Biomolecules. 2015;5(3):1339-85.
136. Elrasheed A, Al Ghaferi H, Ali AY. Assessment of alcohol exposure: testing for ethylglucuronide (Etg), ethylsulfate (Ets). Int J Emerg Ment Health. 2017;19.
137. Viel G, Boscolo-Berto R, Cecchetto G, Fais P, Nalesso A, Ferrara SD. Phosphatidylethanol in blood as a marker of chronic alcohol use: a systematic review and meta-analysis. International journal of molecular sciences. 2012;13(11):14788-812.
138. Aradottir S, Asanovska G, Gjerss S, Hansson P, Alling C. Phosphatidylethanol (PEth) concentrations in blood are correlated to reported alcohol intake in alcohol-dependent patients. Alcohol and alcoholism. 2006;41(4):431-7.
139. Kechagias S, Dernroth DN, Blomgren A, Hansson T, Isaksson A, Walther L, et al. Phosphatidylethanol compared with other blood tests as a biomarker of moderate alcohol consumption in healthy volunteers: a prospective randomized study. Alcohol and Alcoholism. 2015;50(4):399-406.
140. Kader R, Seedat S, Koch J, Parry C. A preliminary investigation of the AUDIT and DUDIT in comparison to biomarkers for alcohol and drug use among HIV-infected clinic attendees in Cape Town, South Africa. African journal of psychiatry. 2012;15(5):346-51.
141. Williams PP, Mathews C, Jordaan E, Washio Y, Terplan M, Parry C. Validity Of Self-reported Alcohol And Other Drug Use Among Pregnant Women Attending Midwife Obstetric Units In The Cape Metropole, South Africa. 2019.
142. Dolman JM, Hawkes ND. Combining the audit questionnaire and biochemical markers to assess alcohol use and risk of alcohol withdrawal in medical inpatients. Alcohol and Alcoholism. 2005;40(6):515-9.
143. Francis JM, Weiss HA, Helander A, Kapiga SH, Changalucha J, Grosskurth H. Comparison of self-reported alcohol use with the alcohol biomarker phosphatidylethanol among young people in northern Tanzania. Drug and alcohol dependence. 2015;156:289-96.
144. Littlefield AK, Brown JL, DiClemente RJ, Safonova P, Sales JM, Rose ES, et al. Phosphatidylethanol (PEth) as a biomarker of alcohol consumption in HIV-infected young Russian women: comparison to self-report assessments of alcohol use. AIDS and behavior. 2017;21:1938-49.
145. Röhricht M, Paschke K, Sack PM, Weinmann W, Thomasius R, Wurst FM. Phosphatidylethanol reliably and objectively quantifies alcohol consumption in adolescents and young adults. Alcoholism: Clinical and Experimental Research. 2020;44(11):2177-86.
146. Jørgenrud B, Kabashi S, Nadezhdin A, Bryun E, Koshkina E, Tetenova E, et al. The association between the alcohol biomarker phosphatidylethanol (PEth) and self-reported alcohol consumption among Russian and Norwegian medical patients. Alcohol and Alcoholism. 2021;56(6):726-36.
147. Piano MR, Tiwari S, Nevoral L, Phillips SA. Phosphatidylethanol levels are elevated and correlate strongly with AUDIT scores in young adult binge drinkers. Alcohol and Alcoholism. 2015;50(5):519-25.
148. Finanger T, Vaaler AE, Spigset O, Aamo TO, Andreassen TN, Gråwe RW, et al. Identification of unhealthy alcohol use by self-report and phosphatidylethanol (PEth) blood concentrations in an acute psychiatric department. BMC psychiatry. 2022;22(1):1-10.
149. McGinnis KA, Tate JP, Bryant KJ, Justice AC, O'Connor PG, Rodriguez-Barradas MC, et al. Change in alcohol use based on self-report and a quantitative biomarker, phosphatidylethanol, in people with HIV. AIDS and behavior. 2022:1-9.

For peer review only - http://bmjopen.bmj.com/site/about/guidelines.xhtml

150. Hasken JM, Marais A-S, de Vries MM, Kalberg WO, Buckley D, Parry CD, et al. Assessing the sensitivity and specificity of phosphatidylethanol (PEth) cutoffs to identify alcohol exposed pregnancies. Current Research in Toxicology. 2023;4:100105.
151. Engel GL. The need for a new medical model: a challenge for biomedicine. Science. 1977;196(4286):129-36.
152. Kissin B, Hanson M. The bio-psycho-social perspective in alcoholism. Alcoholism and clinical psychiatry: Springer; 1982. p. 1-19.
153. Sarma PS, Sadanandan R, Thulaseedharan JV, Soman B, Srinivasan K, Varma RP, et al. Prevalence of risk factors of non-communicable diseases in Kerala, India: results of a cross-sectional study. BMJ open. 2019;9(11):e027880.

For peer review only

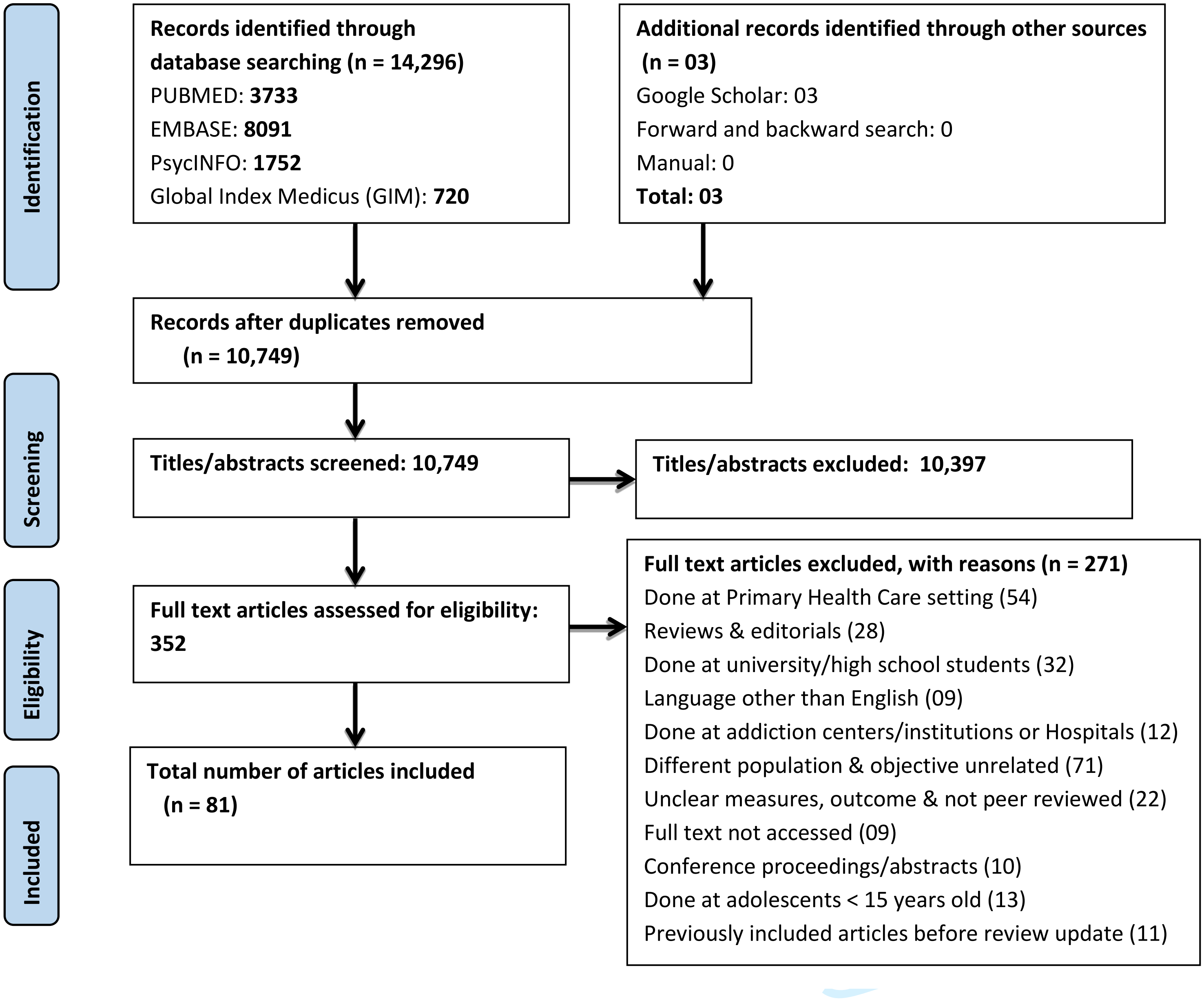

Figure 1: PRISMA flow diagram of included studies in the problem drinking scoping review, 2023.

Supplementary File 1

Search Strategy used for a study "A scoping review of assessment tools for, magnitudes of, and factors associated with problem drinking in population-based studies," 2023.

A) PubMed/MEDLINE:

((((Prevalence [Title/Abstract]) OR "Prevalence" [Mesh])) AND (((alcohol* [Title/Abstract] OR "alcohol abuse" [Title/Abstract] OR "alcohol use" [Title/Abstract] OR "alcohol use disorder" [Title/Abstract] OR "alcohol dependence" [Title/Abstract] OR "alcohol consumption" [Title/Abstract] OR "heavy drinking" [Title/Abstract] OR "risk drinking" [Title/Abstract] OR "harmful drinking" [Title/Abstract] OR "hazardous drinking" [Title/Abstract] OR "binge drinking" [Title/Abstract])) OR ("Alcohol Drinking" [Mesh] OR "Alcoholism" [Mesh] OR "Binge Drinking" [Mesh]))) AND (((Ethiopia [Title/Abstract] OR community-based [Title/Abstract] OR "community based" [Title/Abstract] OR population-based [Title/Abstract] OR "population based" [Title/Abstract])) OR ("Ethiopia"[Mesh] OR "Health Surveys/epidemiology" [Mesh] OR "Population Health/epidemiology" [Mesh]))

B) EMBASE:

1. exp prevalence/
2. prevalence.ti. or prevalence.ab.

3. 1 or 2

4. exp alcohol consumption/ or exp alcohol/ or exp alcohol abuse/
5. exp alcoholism/ or exp drinking behavior/ or exp binge drinking/
6. (alcohol$ or "alcohol abuse" or "alcohol use" or "alcohol consumption" or "binge drinking").ti. or (alcohol$ or "alcohol abuse" or "alcohol use" or "alcohol consumption" or "binge drinking").ab.

7. 4 or 5 or 6

8. exp Ethiopia/
9. "community based".mp.
10. "population based".mp.
11. exp primary health care/

For peer review only

12. (Ethiopia or 'community based' or 'population based' or 'primary health care').ti. or (Ethiopia or 'community based' or 'population based' or 'primary health care').ab.

13. 8 or 9 or 10 or 11 or 12

14. 3 and 7 and 13

15. limit 14 to dd=20190826-20220722

16. limit 14 to rd=20190826-20220722

17. 15 or 16

18. limit 14 to dd=20220722-20231125

19. limit 14 to rd=20220722-20231125

20. 18 or 19

C) PsycINFO:

1. prevalence.mp.
2. prevalence.ti. or prevalence.ab.
3. exp "Alcohol Use Disorder"/ or exp Alcohol Abuse/ or exp Alcohol Drinking Patterns/
4. exp Binge Drinking/ or exp Drinking Behavior/ or exp Alcoholism/
5. (alcohol$ or "alcohol abuse" or "alcohol use" or "alcohol consumption" or "binge drinking").ti. or (alcohol$ or "alcohol abuse" or "alcohol use" or "alcohol consumption" or "binge drinking").ab.

6. 1 or 2

7. 3 or 4 or 5

8. ethiopia.mp.
9. "community based".mp.
10. "population based".mp.
11. exp Primary Health Care/
12. (Ethiopia or 'community based' or 'population based' or 'primary health care').ti. or (Ethiopia or 'community based' or 'population based' or 'primary health care').ab.

13. 8 or 9 or 10 or 11 or 12

14. 6 and 7 and 13

15. limit 14 to up=20190826-20220722

For peer review only

16. limit 14 to ch=20190826-20220722

17. 15 or 16

18. limit 14 to up=20220722-20231125

19. limit 14 to ch=20220722-20231125

20. 18 or 19

D) Global Index Medicus (GIM):

(tw:(prevalence)) AND (tw:(alcohol$ OR "alcohol abuse" OR "alcohol use" OR "alcohol consumption" OR "binge drinking")) AND (tw:(Ethiopia OR "community based" OR "population based" OR "primary health care"))

Abbreviations

Date Delivered (dd): the date a citation XML file was produced for distribution to Ovid with the state = "new." The Date Delivered is removed when a record is revised.

Revised Date (rd): the date the citation XML file was produced for distribution to Ovid with the state="update".This date can change if an updated record is delivered to Ovid.

Update Date/Code (up): The date a record was added to the database since the yearly reload completion.

Correction Date (ch): CH field appears in corrected records and contains the date the record was revised.

For peer review only

Supplementary File 2

Newcastle-Ottawa Scale (NOS) adapted for quality assessment of cross-sectional studies for the study "A scoping review of assessment tools for, magnitudes of, and factors associated with problem drinking in population-based studies," 2023.

**Selection: (Maximum 5 points/scores/stars)**
**1. Representativeness of the sample:**
a. Truly representative of the average in the target population. * (all subjects/consecutive or random sampling)
b. Somewhat representative of the average in the target group. * (non-random sampling)
c. Selected group of users/convenience sample.
d. No description of the derivation of the included subjects (sampling strategy).
**2. Sample size:**
a. Justified and satisfactory (including sample size calculation). * (1 score)
b. Not justified
c. No information provided
**3. Non-respondents:**
a. Proportion of target sample recruited attains pre-specified target or basic summary of non-respondent characteristics in sampling frame recorded. *
b. Unsatisfactory recruitment rate, no summary data on non-respondents.
c. No information provided
**4. Ascertainment of the exposure (risk factor/disease) or screening/surveillance (measurement) tool:**
a. Secure record (medical charts) or validated measurement (screening/surveillance) tool. **
b. Non-validated measurement tool, but the tool is available or described or Self-report. *
c. No description of the measurement tool.
**Comparability: (Maximum 2 stars)**
**1. Comparability of subjects in different outcome groups on the basis of design or analysis. Confounding factors controlled.**
a. Data/results adjusted for relevant predictors/risk factors/confounders e.g., age, sex, marital status, job etc. **

For peer review only

b. Data/results not adjusted for all relevant confounders/risk factors/information not provided.
**Outcome: (Maximum 3 stars)**
**1. Assessment of outcome:**
a. Independent blind (structured) assessment. **
b. Record linkage. **
c. Self report. *
d. No description.
**2. Statistical test:**
a.Statistical test used to analyse the data clearly described, appropriate, and measures of the association presented including confidence intervals and probability level (p-value). *
b.Statistical test not appropriate, not described, or incomplete.

Scoring for cross-sectional Studies:

Very Good Studies: 9-10 points

Good Studies: 7-8 points

Satisfactory Studies: 5-6 points

Unsatisfactory Studies: 0 to 4 points

For peer review only

References

1. Ribeiro CM, Beserra BTS, Silva NG, Lima CL, Rocha PRS, Coelho MS, et al. Exposure to endocrine-disrupting chemicals and anthropometric measures of obesity: A systematic review and meta-analysis. BMJ open. 2020;10(6):e033509.

2. Zhao C, Xing F, Yeo YH, Jin M, Le R, Le M, et al. Only one-third of hepatocellular carcinoma cases are diagnosed via screening or surveillance: a systematic review and meta-analysis. European journal of gastroenterology & hepatology. 2020;32(3):406-19.

3. Patra J, Bhatia M, Suraweera W, Morris SK, Patra C, Gupta PC, et al. Exposure to second-hand smoke and the risk of tuberculosis in children and adults: a systematic review and meta-analysis of 18 observational studies. PLoS medicine. 2015;12(6):e1001835.

4. Modesti P, Reboldi G, Cappuccio F. Newcastle-Ottawa Quality Assessment Scale (adapted for cross sectional studies). PloS one. 2016;11(1):e0147601.

5. Dubey VP, Kievišienė J, Rauckiene-Michealsson A, Norkiene S, Razbadauskas A, Agostinis-Sobrinho C. Bullying and Health Related Quality of Life among Adolescents—A Systematic Review. Children. 2022;9(6):766.

6. Naafs JC, Vendrig LM, Limpens J, Van Der Lee H, Duijnhoven RG, Marchal J, et al. Cognitive outcome in congenital central hypothyroidism: a systematic review with meta-analysis of individual patient data. European journal of endocrinology. 2020;182(3):351-61.

For peer review only

Supplementary File 3

Table: Prevalence, associated factors, and pattern of problem drinking in high-income countries (HICs), 2023.

**Author, Year**<br><br>**Country/Location**	**Study Design & Study Setting (population)**	**Participants: Sample size** (Male subjects, %) Mean age (range) in years	**Tools (measures) or questions used**	**Outcomes: (Definition/nature of use)**	**Results & statistical methods used.**
**Aalto et al., 1999**<br><br>**Finland (town of Lahti)**	**Cross-sectional** PHC outpatients & General population (Urban residents)	PHC,2370 (40.3%)<br>OHC,3268 (29.3%)<br>GNP,544 (51.7%)<br>38-41(20-60) years	Quantity or frequency questionnaires **(QFQs)** (last 2 month) **CAGE**	**Heavy drinking:**<br>**Male**: ≥ 280g of absolute ethanol /24 drinks/week/ &/or ≥ 3 in CAGE.<br>**Women**: ≥ 190g/16 drinks per wk &/or ≥ 2 in CAGE.<br>**Abstinence:** no self-reported drinking at all & no answers to CAGE	**t-test & Chi-square analysis:**<br>**Men:** heavy drinking in PHC, OHC & GNP were 19.5%, 17.3% & 16.4%, respectively.<br>**Women:** corresponding figures were 8.6%, 6.2% & 12.9%.
**Aira et al., 2005**<br><br>**Finland (City of Kuopio)**	**Cross-sectional** home-dwelling elderly persons, **Community-based** (Urban residents)	700 persons (27.4% men)<br>81 (75-95.7) years	**QFQs** (1 year) & **CAGE**	**Four categories:**<br>Abstainers,<br>< 1 unit/week,<br>1–7 units/week, &<br>> 7 units/week.	**Chi-square & t-test** (frequencies vs means):<br>44% had used alcohol during past year (65% of men & 36% of women).<br>≥ 3 units/occasion used by 2.9% of women & by 11.7% of men.
**Andrews-Chavez et al., 2015**<br><br>**United States (Greater Boston area, MA)**	**Cross-sectional** (Puerto Rican adults, Hispanics). (Urban residents)	1472 adults (29.6% men)<br>? (45–75) years	**QFQs NIAAA** definitions **(NIAAA guidelines)**	**Lifetime abstainer (LA):** (**< 12 drinks** in lifetime)<br>**Former drinker (FD):** (**> 12 drinks** in lifetime, but not currently drinking)<br>**Moderate drinker (MD):** (**Man/women**: ≤14/7drinks per week & ≤ 4/3 drinks/d)<br>**Heavy drinker (HD)**: (**Man/women**:>14/7drinks per week & > 4/3drinks/d)	**A multinomial logistic regression model:** 8% men & 39% women were **LAs;** 40% of men & 25% women (**FDs**); **&** 21 % men & 8 % of women **(HDs).**<br>**Young men**: likely than older to be MDs.<br>**Women**: higher BMI, age, lower income & psychological acculturation (associated with abstention); age, lower perceived emotional support associated with increased FD; & women without v. with diabetes were more likely to be heavy drinkers.
**Bataille et al., 2003**<br><br>**France (Lille, Strasbourg & Toulouse)**	**Cross-sectional** (3rd MONICA) **Population survey** (Urban/Semi-urban & rural)	3508 subjects (51.0% men)<br>50.3 (35–64) years	Self-reported **QFQs** French alcohol consumption habits	**Heavy drinkers:**<br>**Men:** ≥ 60g ethanol/day, (6 glasses/d-any drink) &<br>**Women:** ≥ 30g/day (3 glasses/day)	**Multivariate analyses:**<br>14% men & 40.8% women (non-drinkers) 9.0% women & 14.4% of men were HDs.<br>Low educational level, smoking, apoprotein B, HDL, MCV), GGT & CAGE score for men, & living area, age, MCV, GGT & the CAGE score for women were significantly associated with **heavy drinking (HD).**

For peer review only - http://bmjopen.bmj.com/site/about/guidelines.xhtml

				**Reference class (RC):** non-drinkers & moderate drinkers together.	
**Coulson et al., 2010**<br><br>**Australia (south Eastern)**	**Cohort study** (Geelong Osteoporosis Study, GOS)<br>**Community-based cohort** (secondary data)	1420 men (100%)<br>56 (20 – 93) years	Validated self-report **FFQ**<br>**Mean daily alcohol intake** (Australian National Health & MRC 2009 guidelines)	**Consumption/12 months**: (never, < 1/month, 1–3 days/month, 1–6 days/week & every day<br>**Mean daily alcohol intake** non-drinkers/nil, > 0 but ≤ 2 drinks/ day, > 2 drinks/day<br>**(with in past 12 months)**	**ANOVA & Multivariate analyses:**<br>Age-standardized proportion of non-drinkers was 8.7%, 51.5% consumed ≤ 2 drinks/day (≤ 20g/day), & 39.9% > 2 standard drinks per day (> 20g ethanol/day).<br>**Alcohol use (> 20g/day)** was positively associated with cigarette smoking, weight, higher SES & inversely with age & physical activity.
**Foulds et al., 2012**<br><br>**New Zealand**	**Cross-sectional** (Permanent private dwellers)<br>**Population survey**	12,488 adults (42.2% male)<br>? (≥ 15 years)	**AUDIT**	**Harmful/hazardous drinking (HHD):**<br>Score of ≥ 8 on AUDIT	**Crosstabs & logistic regression models:**<br>**HHD:** 17.7% (men, 25.6%; women, 10.4%);<br>**Overall, 9.4%** of attendees with HHD reported talking about alcohol.
**Geels et al., 2013**<br>**Netherlands**	**Cross-sectional** (All Netherlands Twin Register, NTR registered at a valid address)<br>**Population survey** (Urban)	16,587 subjects (36.5% men)<br>41.6 (18–97) years	**QFQs** (12 mo.)<br><br>Health Council of Netherlands recommended limit<br>CAGE & AUDIT	**Excessive alcohol use:**<br>**Women:** > 14 standard glasses per week<br>**Men:** > 21 drinks/week	**Linear/logistic/multinomial regressions:**<br>>30.0% of men & >20% of women drinking 6–7 times per week)<br>**Women:** 25–45 years had 5.7-5.9% of excessive drinking, & 55–65 years (15.5%)) Older age, sex (male), and initiation of cigarette & cannabis use were predictors of alcohol use
**Janghorbani et al., 2003**<br><br>**Hong Kong (China)**	**Cross-sectional** (Cantonese-speaking adult population)<br>**Population-based** (Urban)	2900 subjects (48.7% men)<br>45.8 (25–74) years	**QFQs** (weekly)	**Heavy drinkers:** men, > 400g & women, > 280g/wk<br>**Light drinkers:** men, < 168g & women, <112g/wk<br>**Moderate drinkers:**<br>Men: ≤ 400g/ ≥ 168g &<br>Women: ≤ 280g/ ≥112g/wk<br>**Binge drinking**: ≥ 5 drinks in a row in the past month.	**GLMs/multiple/logistic regression models:**<br>**Mean weekly alcohol consumption**: 64.3g, men & 13.7g, women (P < 0.001).<br>Current drinking vs non-drinking, male sex, smoking (women), HDL, ≤ primary education, diastolic BP & separated/widowed were associated positively with **weekly ethanol consumption.**
**Kim et al., 2008**<br><br>**Hong Kong (China)**	**Cross-sectional** (All Hong Kong Chinese adults)<br>**Population based** (Urban)	9860 adults (50.0% men)<br>28 (18–70) years	Pattern **(QFQs)**<br><br>**CIDI** (Chinese version based on DSM-IV)	**Mean drinking/past year:** < once/wk, 1–3 times/wk, 4+ times/week)<br>**Binge drinking/past mo.** (5 servings of alcohol per one occasion in 30 days)<br>**Alcohol abuse or dependence**	**Stepwise multivariate logistic regression:**<br>**10.9% of entire sample** reported at least one of AUDs (AA, AD & binge drinking).<br>**Binge drinking :**14.5% in males (18.7% AA & 12.3% AD) & 3.5% in females (16.0% AA & 9.9% AD)<br>**Male binge drinkers** were less likely to be older & students but more likely to be employed in service industry.

For peer review only - http://bmjopen.bmj.com/site/about/guidelines.xhtml

				(Chinese CIDI)	**Female binge drinker**: less likely to be > 60 years or married & more likely to be smokers<br>**In both genders,** smoking was significantly associated with binge drinking
**(Chou et al., 2011**<br><br>**United States**	**Prospective study** (subsample of 3-year prospective study**; waves 1 & 2 of NESARC**)<br>**Population-based** (Urban)<br>**secondary data analyses**	13,442 analyzed (40.6% men)<br>? (≥ 50 years)	**QFQs**<br><br>**AUDADIS-IV** (DSM-IV)	**Binge drinking (BD):** ≥ 5 drinks/occasion (men) ≥ 4 per occasion (women)<br>**Current drinkers:** without BD<br>**Occasional BD:** < monthly in past year) &<br>**Frequent BD:** ≥ 1/month in past year<br>**DSM-IVAUDs** (Alcohol use, AA & AD)	**Multinomial & logistic regression:**<br>BD was 24.7% in men & 12.4% in females.<br>Overall, male respondents were significantly more likely to have BD.<br>Both men & women with occasional BD & frequent BD were significantly more likely than current male/female drinkers without BD to have **alcohol abuse disorder and alcohol dependence disorder (AUDs)**
**Latvala et al., 2009**<br><br>**Finland**	**Cross-sectional** (Finnish young adults)<br>**Population-based** (Urban)	605-diagnostic assessment done (sex unspecified)<br>28.6 (21-35) years	**SCID-I** complemented by medical record data	**Lifetime Substance Use Disorders (SUDs):**<br><br>DSM-IV diagnosis	**t-tests, X^2 tests & logistic regression:**<br>Lifetime AA or AD were 13.1% (19.8% for males & 6.3% for females). And total prevalence of AA & AD alone was 7.6% & 5.6%.<br>Behavioral, affective & parental factors, early initiation of substance use, learning difficulties & lower education were found to be associated with **alcohol & other SUDs.**
**Meyer et al., 2000**<br><br>**Germany (Northern, city of Lubeck)**	**Cross-sectional** of longitudinal project (Adult general population)<br>(Urban)	4075 analyzed (50.2% of men)<br>? (18 to 64 years)	**M-CIDI** (DSM-IV, adapted CIDI)<br>**Ever/current** QFQs	**Hazardous consumption:** 20-40g/d (women) & 30-60g/day (men) and<br>**Harmful consumption:** > 40g/day (women) & > 60g/d (men)<br>**AA or AD**:<br>DSM-IV Diagnosis (M-CIDI diagnostic software)	**Logistic regression analyses:**<br>Lifetime AUDs (4.5% AA, 3.8% AD) & men vs women for AA (8.1% vs 1.0%) & AD (6.0% vs 1.5%) respectively<br>**Hazardous & harmful consumption:** (13.2% lifetime; 6.0% in last 12-months)<br>**Male:** more affected by lifetime AUDs.<br>Association between AUDs & alcohol consumption pattern revealed a weaker relation for AA compared to AD.
**Miller et al., 2004**<br><br>**United States**	**Cross-sectional** (US Adults; BRFSS, telephone survey & NSDUH, an in-person survey)	**355,371** (BRFSS)<br>**87,145** (NSDU) were analyzed (sex unspecified)<br>? (≥ 18 years)	**Pattern (QFQs)**	**Binge drinking**: ≥ 5 drinks on an occasion	**two-tailed t-test:**<br>National binge drinking prevalence was:<br>14.7% for BRFSS and 21.6% for NSDUH<br>Most binge drinkers were male (74% BRFSS, 68% NSDUH) & white, non-Hispanic (73% BRFSS, 76% NSDUH)
**Mohler-Kuo et al., 2015**<br><br>**Switzerland**	**Cohort study** (Young Swiss men from C-SURF)<br>**Population-based**	5943 total sample (100% men)<br>20.0 (18–25) years	DSM-IV & DSM-5 criteria<br><br>QFQs	**AA & AD** (DSM-IV) &<br>**AUD** (≥ 2 criteria-DSM-5) (12-month prevalence)<br>**RSOD** (≥ 6 drinks/single occasion)	**Multinomial logistic regression:**<br>31.7% met DSM-5 AUD (21.2% mild; 10.5% moderate/severe], less than overall DSM-IV criteria for AA & AD (36.8%)

For peer review only

	(Rural, 60.3%; Urban, 39.7%)		RSOD & at-risk volume drinking	**At-risk volume drinking** (≥ 21 drinks/wk & RSOD at least monthly)	Relative to those meeting both DSM-IV & DSM-5 criteria, all other subgroups reported less alcohol and illicit drug use.
**Neumark et al., 2007**<br><br>**Israel**	**Cross-sectional** (Israeli adults) **National population-based survey**	4,859 adults (49.0% men) ? (≥ 21 years)	**WMH-CIDI** (lifetime & past 12-month DSM-IV Dx)	**DSM-IV** (AA & AD) **Frequent drinking:** (3 ormore times in one week at least once) in the past year. **Frequent heavy drinking:** consumption of ≥ 3 drinks, ≥ 3 times a week at least once during past year	**Logistic regression models:** **Lifetime AD** was 41%, **Frequent drinking was 5%, & frequent HD was** (6.8% of men & < 1% of women) Lifetime AA/AD was 4.3% (4.0%, AA & 0.4% AD criteria) Significantly higher rates among males (AOR=7.3), younger adults (AOR=5), immigrants (AOR=2.0) & never married (AOR=1.6)
**Proodfoot and Teeson, 2002**<br><br>**Australia**	**Cross-sectional** (Australian National Survey of Mental Health & Wellbeing, NSMHWB)	10,641 respondents (sex unspecified) ? (≥ 18 years)	**CIDI 2.1** **(**modified WHO version) **QFQs**	**DSM-IV Diagnosis for AA & AD** **High level of dependence:** ≥ 4 criteria for dependence.	**Multiple logistic regressions:** AD was 4.1% (males 6.1% & females 2.3%) Variables correlated with AD were male sex, young age (18-34); not being in a married or de facto relationship & having any affective, anxiety or other substance use disorder.
**Veerbeek et al., 2019**<br><br>**Netherlands**	**Cohort study** (Data from, NEMESIS-2 **Population-based** (6 categories of urbanicity: very high to very low)	4618 persons (sex unspecified) ? (23–70 years)	**CIDI V 3.0** DSM-IV International guidelines for alcohol use definitions	**Alcohol disorder:** AA &/or AD (past 12 months) **Heavy alcohol use:** > 14 drinks/wk (women) & > 21 drinks/wk for men	**Multinomial logistic regression analyses:** Prevalence of heavy alcohol use was higher in older (55–70 years) than younger people (6.7% vs 3.8%), but alcohol disorder was less prevalent (1.3% vs 3.9%). Heavy alcohol use was associated with higher level of education in older adults compared to younger adults.
**Williamson et al., 2003**<br><br>**United Kingdom**	**Cross-sectional** (Subjects from 26 general practices registered with MRC-GPRF) Community-based project in the UK<br><br>**Secondary data**	**20,062** unrelated index subjects (40.0% men) ? (20–60 years)	**UK definition** for binge or heavy drinking behaviour &<br><br>**QFQs** for (single session drinking criteria)	**Binge/heavy session drinkers**: males > 8 & females > 6 units/session<br><br>**Non (binge/heavy session) drinking:** not fulfilling session drinking criteria, including abstainers	**No statistical analysis performed** Average number of units of alcohol per week consumed was 16 for men and 8 for women. 17% of subjects had binge drinking fashion. (15% for male vs 18% for females) Binge drinking was found to be most prevalent amongst males & females in their 20s (33% of male vs 38% of females).
**Auchincloss et al., 2022**<br><br>**USA (**Philadelphia, Pennsylvania metropolitan area)	**Cross-sectional analyses** (population-based cohort) (Urban setting)	**772 (cross-sectional analyses)** (48% men) ? (21–64 years)	Quantity/ Frequency Questions **(QFQs)** RSOD criteria	**BD** (SAMHSA definition): at least one day in past 30 days the person consumed a high volume of alcohol on a single occasion (≥5 alcoholic drinks for males and ≥ 4 for females).	**Logistic regression and Poisson regression** Among alcohol users in either time period, 22% consumed 8 or more drinks per week and 37% reported at least 1 binge occasion in the past 30 days. higher outlet density was associated with more alcohol consumption and residing farther from an outlet was associated with less alcohol consumption.

For peer review only

**Bott et al., 2005**<br><br>**Germany**<br>(Lübeck city and its catchment area)	**Cross-sectional**<br>(part of a longitudinal study)<br>(urban setting)	4,074 (analysis)<br>(44.9% men)<br>42.7 (18-64 years)	DSM-IV based Munich CIDI (M-CIDI).<br>**Quantity/ frequency index,QFI**<br>(at-risk drinking = Based on the British Medical Association's, 1995, recommendations)	**Four alcohol-use groups:**<br>(1) moderate drinkers/ abstainers (MOD/A): < 12 times in their lives or <20g/women & <30g/men pure alcohol/day<br>**(2)** at-risk drinkers (ARD): >20/30g pure alcohol/day<br>(3) DSM-IV criteria for alcohol abuse (AA)<br>(4) DSM-IV criteria for alcohol dependence (AD)	**Multinomial regression analysis (multivariate associations):**<br>9% of participants were at-risk drinkers.<br>Prevalence rates for at-risk drinkers were 16.9% for affective, 18.1% for anxiety and 17.8% for somatoform disorders.<br>Compared with MOD/A, atrisk drinkers showed a 2-fold increased risk of having a psychiatric disorder. Subjects with AA showed a comparable level of risk & with AD showed an even greater risk. Female at-risk drinkers were twice as likely to have a psychiatric disorder compareed to male.
**Britton et al., 2020**<br><br>**United Kingdom**	**Cross-sectional**<br>(part of Whitehall II study, civil servants at phase 11 (2012–13)<br>(urban setting)	6117 (alcohol & sleep data)<br>(70.9% men)<br>Mean age: 69.4 men, 69.6 women<br>(61–81 years)	**Volume of consumption**<br>(drinks used in last 7 days)<br>**Retrospective alcohol life-course grid**<br>(AUDIT-C)	**Hazardous drinking/HD**:<br>≥ 5 points on AUDIT-C<br>**Non-drinkers**: didn't drink alcohol in past year.	**Logistic regression:**<br>15.7% of men consumed 21 or more units per week compared to only 2.4% of women.<br>30.5% men & 12.8% women reported HD.<br>men drinking > 21 units/wk or drinking hazardously were more likely to have disturbed sleep than those not drinking in past week or not drinking hazardously.
**Husberg et al., 2022**<br><br>**Norway (Tromsø)**	**Cross-sectional data**<br>(population-based)<br>(Tromsø 1-7, T7 = 2015-2016<br>(urban setting)	**19,185 (analysis)**<br>(47.5% men)<br>Mean age: 57.2 women, 57.4 men<br>(40-96 years)	**AUDIT:**<br>Hazardous alcohol use (HAU)	Hazardous alcohol use:<br>AUDIT ≥ 8 as a cut-off	**Logistic binomial regression model:**<br>Insomnia was more prevalent among participants with a HAU (24.1%) than without (18.9%).<br>Participants who had HAU had higher odds of insomnia (OR= 1.49).
**Lee et al., 2020**<br><br>**Singapore**	**Cross-sectional**<br>(Singapore Mental Health Study, SMHS 2016)<br>(urban setting)	**6126 (interviewed)**<br>(50% men)<br>? (18 yrs & above)	**QFQs** (alcohol use)<br>**CIDI 3.0**<br>(mental disorders)<br>**DSM-IV**<br>(diagnosis of mental disorders)	**Bing Drinking (BD):**<br>consumption of 5 or more drinks (male) or 4 or more drinks (female) on a single occasion in the past 12 months.	**Multiple logistic regressions**<br>13.7% reported past-year BD (17.6% of males and 9.8% of females).<br>Moderate associations between BD and mood and anxiety disorders (ORadj =1.8–4.4), were noted, while associations with AUDs were much stronger (ORadj=5.3–9.7).<br>Associations betweenn BD & anxiety disorders were observed exclusively in females (ORadj=2.3–3.3). Binge drinkers reported a lower quality of life compared to their non-binging counterparts.

For peer review only

**Lindstrom et al., 2020**<br><br>**Sweden**	**Cross-sectional**	11,716<br>(50.4% men)<br>? (65-99 years)	AUDIT-C (Alcohol consumption)	non-drinker = 0; moderate drinker = 1–7 (male), 1–5 (female); risk-drinker = 8–12 (male), 6–12 (female). Non-drinker was not consumed alcohol during the last 12 months.	**Logistic regression analysis**<br>Men (83%) were more prone to drink alcohol compared to women (71%). The prevalence of risk drinking was about 2% for both genders.<br>Alcohol consumption declined with age. Moderate consumption of alcohol was associated with lower probability of poor SRH compared to non-drinking (AOR=0.64 for men) and (AOR= 0.68 for women).
**Lundin et al., 2021**<br><br>**Sweden** (Gothenburg, second largest city in Sweden)	**Longitudinal** (Women and Alcohol in Gothenburg (WAG) Study, cohort in 1986, 1994/2000 & 2013) (urban setting)	**1,614 (baseline)**<br>(100% women)<br>? (across different age-group?)	CIDI-SAM, ICD-10 & ICD-1, DSM-IV & DSM-5	AUD, alcohol abuse (AA), alcohol dependence (AD) based on CIDI-SAM or (DSM-III, DSM-III-R, DSM-IV, DSM-5, & ICD-10 & ICD-11)	**contingency tables & Cohen's Kappa coefficient (κ)**<br>Baseline: prevalence of lifetime AD was 10.6 % (ICD-11); 4.0 % (ICD-10); 4.3 % (DSM-IV); 7.5 % (DSM-III-R); and 12.3 % (DSM-III). DSM-5 AUD was 14.3 %.
**Mason-Jones and Cabieses, 2015**<br><br>**Chile**	**Cross-sectional**<br><br>(Chilean National Health Survey 2010, ENS 2010) (88% lived in urban settings)	Adolescents (absolute n=435, weighted n = 1860812)<br>Young adults (absolute n = 412, weighted n = 1386 547)<br>(50.3% men)<br>? (adolescents 15-20 years & young adults 21-25 years).	**QFQs** (Alcohol prevalence in last year, & BD prevalence in last month)	**Alcohol prevalence in last year:** 'yes' labeled as "1" and 'no' labeled as "0".<br>**BD prevalence last month:** had drunk four or more units of alcohol in a single episode in the last 4 weeks.	**Conditional logistic regression models:**<br>65% of adolescents and 85% of young adults reported drinking alcohol in the last year &<br>of those (who used alcohol in the last year) 83% of adolescents and 86% of young adults reported BD in the previous month. Adolescents who reported binging alcohol were also more likely, compared to young adults, to report being depressed (OR 12.97) or to feel very anxious in the last month.<br>Adolescent females were more likely to report poor life satisfaction in the previous year (OR 8.50), feel depressed (OR 3.41).<br>Being female was also associated with a self-reported diagnosis of depression for both age groups.
**Mondi et al., 2022**<br><br>**USA (Chicago)**	**Cross-sectioal**<br><br>(CLHS data collection, predominately Black sample) (grew up in urban poverty)	**301** CLHS participants<br>(40% men)<br>? (32-37 years invited to CLHS)	**M.I.N.I. 7.0.2.** (based on DSM-IV & ICD-10 criteria)	DSM-IV & ICD-10 criteria for major depressive disorder, generalized anxiety disorder, post-traumatic stress disorder, substance use disorder, and AUD.	**Independent samples t-tests**<br>Males endorsed significantly higher rates of any AUD within the past 12 months (38.3%) than females (20.6%).<br>Probable prevalence rate for any AUD was 27.7%.

For peer review only - http://bmjopen.bmj.com/site/about/guidelines.xhtml

**O'Dwyer et al., 2019**<br><br>**Ireland**	**Cross-sectional**<br>(Data generated from 2013 National Alcohol Diary Survey, NADS)	**4338 drinkers**<br>(49.9% men)<br>? (18–75 years old)	**RSOD** criteria (HED)<br>**DSM-IV (CIDI)**<br>**Alcohol-related harms/ARH (8 questions)**	**HED:** consuming 60 g or more of pure alcohol in a single drinking occasion.<br>**Alcohol dependence** (AD) (DSM-IV criteria)<br>Current drinkers, non-drinkers, monthly HED, occasional HED, low-risk drinkers, ARH	**Crosstabs (Pearson χ2, bivariate assoc.)**<br>There was a relatively even breakdown of low-risk (31.0%), occasional HED (30.6%), and monthly HED (31.5%) drinkers.<br>AD constituted 6.9% of all drinkers.<br>Overall, 29% of drinkers experienced at least one harm from their own drinking in last year.<br>Respondents who were AD had a greater individual risk of experiencing each harm.
**Shockey and Esser, 2020**<br>**USA** (District of Columbia and territories)	**Cross-sectional**<br><br>(U.S. employed adults who resided in 32 states, BRFSS data)	**358,355 employed adults**<br>(48% men)<br>? (18-55 years)	Industry & occupation (I&O) optional module<br>BRFSS &<br>**QFQs**	**BD**: men consuming ≥ 5 drinks or women consuming ≥ 4 or more drinks, on an occasion.	**No statistical analysis performed.**<br>20.8% reported BD, with an average of nearly 49 times per year and an average intensity of 7.4 drinks per binge episode, resulting in 478 total binge drinks per binge drinker. The adjusted BD prevalence ranged from 15.9% among community and social services workers to 26.3% among construction and extraction workers.

Abrevations: **AA:** alcohol abuse; **AD**: alcohol dependence; **ARH**: Alcohol Related Harm; **AUD:** Alcohol Use Disorder; **AUDADIS-IV**: Alcohol Abuse and lcoholism's Alcohol Use Disorder and Associated Disabilities Interview Schedule– DSM-IV Version; **AUDIT**: Alcohol Use Disorder Identification Test; **BD**: Binge Drinking; **BRFSS**: Behavioral Risk Factor Surveillance System; **CLHS**: Chicago Longitudinal Health Study; **C-SURF**: Cohort Study on Substance Use Risk Factors; **FFQ**: Food Frequency Questionnaire; **GLM**: General linear models; **GNP**: General Population; **GPRF**: General Practice Research Framework; **HAU:** Heavy alcohol use; **ICD-10/11:** International Classification of Diseases 10th/11th Revision; **MONICA**: MONItoring of trends and determinants of CArdiovascular disease; **NIAAA**: National Institute on Alcohol Abuse and Alcoholism; **NRR**: Non response rate; **wk**: week; **NSDUH**: National Survey on Drug Use and Health; **OHC**: Occupational Health Care clinic; **PHC:** Primary Health Care clinic outpatients; **QFQs**: Quantity Frequency Questionnaires of alcohol use; **RSOD**: Risky Single-Occasion Drinking; **SAMHSA**: Substance Abuse and Mental Health Services Administration; **USA:** United States of America; **yr.:** year; ?: mean age is not mentioned.

For peer review only

Supplementary File 4

Table: Prevalence, associated factors, and pattern of problem drinking in low-and middle-income countries (LMICs), 2023.

Author, Year<br><br>Country/Location	Study Design & Study Setting<br><br>(population)	Participants: Sample size (Male, %) Mean age (range) in years	Tools (Measures) or questions used	Outcomes: (Definition/nature of use)	Results & statistical methods used:
**Andersson et al., 2018**<br><br>**South Africa**<br>(Eastern Cape Province)	**Cross-sectional**<br>(Nelson Mandela Metropolitan & Sundays River Valley City)<br>**Population-based**<br>(Urban/semi-urban/ rural setting)	1000 participants (52% of men)<br>27 (18-40) years	**M.I.N.I. 6.0 (DSM-IV)**	**Alcohol dependence & Alcohol abuse (AD/AA):** (DSM-IV diagnosis during the past 12 months)	**χ2 statistics & logistic regression models:**<br>AD: 26.5% (39.0% men & 19.1% women)<br>AA: 9% (19.0% for men & 6.0% for women).<br>**AD:** higher in rural/semi-rural in men (43.1%) and women (26.8%) than in urban/semi-urban.<br>Widowed and separated women compared to married or cohabiting and women with low income (don't want to disclose) compared to weekly household income of ≥ 1,001 RAND remained statistically significant.
**Burazeri and Kark, 2010**<br><br>**Albania**<br>(Tirana)	**Cross-sectional**<br>(transitional post-communist Albania (Muslim, 68.5%)<br>**Population-based**	685 individuals (65.7% of men)<br>52.6 (35–74) years	Quantity/ frequency questionnaires **(QFQ**s) (patterns questions) (12 months)	**Drunkenness/hangovers:** never, very exceptionally, 2-3 times/year, 1/month, 1/fortnight & once/week). Composite Binging score: drunkenness or hangovers during w/c ≥3 units (≈60g of ethanol) consumed	**Binary/multivariable logistic regression:**<br>10.3% of men had ≥ 2-3 annual episodes of drunkenness & and hangovers each.<br>**Women**: both markers of binging, 1.4%<br>**Men:** 8.9% drinking ≥ 60g alcohol/session.<br>Binge drinking was related to low educational level, financial loss in pyramid collapse, & religiosity (inversely) in both Muslims and Christians (all in men).
**Dias da Costa et al., 2004**<br><br>**Brazil**<br>(Rio Grande do Sul State)	**Cross-sectional**<br>(Adults of municipality of Pelotas)<br>**Population-based**<br>(Urban area)	2,177 adults (43%)<br>41.6 (20-69) years	**QFQs**<br>(weekly use)	**Moderate consumption:** up to 30g/day of ethanol)<br>Heavy consumption or hazardous drinking, HD: ≥ 30g/day of ethanol/week	**Non-conditional logistic regression:**<br>Moderate consumption was 65.1%<br>**HD:** 14.3% (29.2%, men & 3.7% in women).<br>Men, elders, blacks, low SES, heavy smokers, & chronic disease presented higher prevalence of HD. Men with minor psychiatric disorders had higher prevalence of HD & in women (association between age & HD was inversely related).

For peer review only - http://bmjopen.bmj.com/site/about/guidelines.xhtml

**Ji et al., 2018**<br><br>**China** (Xuzhou city, Jiangsu)	**Cross-sectional** (11 regions in Xuzhou city)<br>**Population-based** (urban/rural areas)	36,157 participants (48.40% of men)<br>45.5 (18-75) years	**MAST**	**Alcohol dependent (AD):** MAST score of ≥ 5<br>0 (no alcohol dependence)<br>1–4 (low AD), 5–6 (light AD), 7–25 (mild AD), 26–39 (moderate AD & 40–53 (severe AD)	**χ2 &, t-tests; multivariate log. Regression:**<br>**AD:** 11.56% (22%, males & 1.74%, females)<br>Newly detected hypertension rate was 9.46%<br>Significant associations were found between AD & blood pressure. AD was positively correlated with systolic blood pressure & diastolic blood pressure (r = 0.077, P< 0.01).
**Mendoza-Sassi and Beria, 2003**<br><br>**Brazil**	**Cross-sectional** (Residents in municipality of Rio Grande, Southern Brazil)<br>**Population-based** (Urban population)	1260 people (46.1% of men)<br>40.3 (15-94) years	**AUDIT**<br><br>**SRQ-20**	**Alcohol Use Disorder (AUD):** AUDIT score ≥ 8	**Log. regression in multivariate analysis:**<br>**AUDs:**7.9% (2.5%, women & 14.5%, men).<br>Risk of alcohol misuse increased across increasing social class (P linear trend = 0.03)<br>Males had OR=6.89 compared with women.<br>Smokers (OR 3.27) & ex-smokers (OR 1.30) were at higher risk than non-smokers.<br>Those with minor psychiatric disorders had a 2.48 OR of presenting a positive test (AUD).
**Moreira et al., 1996**<br><br>**Brazil** (Porto Alegre)	**Cross-sectional** (Adult population of Porto Alegre, Southern Brazil)<br>**Population-based** (Urban)	1,091 individuals (45.0% of men)<br>Mean age: 41/men; 44/women<br>(≥ 18) years	**CAGE** & Type & **QFQs** of alcoholic drink	**Heavy drinking (HD):** Average of ≥ 30g/day<br>**Alcohol dependence/AD:** Two positive answers to the CAGE questionnaire	**X^2-test & logistic regression models:**<br>**AD** was 9.3%; **heavy drinking** was 15.5%.<br>Increasing age, lower education & income, non-white race (associated with HD & AD).<br>Households with 3-4 persons were associated with lowest risk HD, but AD was higher in crowded households (5-11). Presence of one with HD/AD in household was associated with HD but not with AD.
**Peltzer et al., 2011**<br><br>**South Africa**	**Cross-sectional** (Part of SABSSM 2008 survey) (62.5% located in urban areas)	13,828 persons (43.7% of men)<br>? (≥ 15 years)	**AUDIT**	**Binge drinking (BD):** Females (4) & males (5) standard drinks/occasion<br>**Hazardous or harmful drinking:** AUDIT cut-off score ≥ 8	**Adjusted logistic regression:**<br>**Risky (hazardous/harmful drinking):** 9% (17% among men & 2.9% for women)<br>Overall prevalence of BD: 9.6%<br>**Men:** risky drinking was associated with 20-54 years than 15-19; Colored population group; lower (economic status & education.)<br>**Women:** risky drinking was associated with urban residence, Colored population group; lower education; and higher income
**Peltzer and Phaswana-Mafuya, 2013**<br><br>**South Africa**	**Cross-sectional** (older South Africans, Study of Global Ageing &	2144 participants (41.1% of men)<br>? (> 60 years old)	**QFQs** & **NIAAA** risky drinking criteria	**Risky drinking (2 ways):**<br>**Heavy drinkers:** (>7 drinks per week) &<br>**Binge drinkers:**	**Multivariate logistic regression:**<br>**Heavy & binge drinking**: 4% vs 3.7%<br>Male gender, white population group; tobacco use & being obese were associated with risky drinking.

For peer review only

	Adults Health, SAGE in 2008) **Population-based** (Urban, 63.2%)			(>3 drinks/one occasion at least weekly)	Hypertension, diabetes, and depression were not associated
**Peltzer et al., 2012**<br><br>**South Africa**	**Cross-sectional** (South African Youths, Black, 97.5%; 4 of 9 provinces in SA) **Population-based**	3123 participants (54.6% of men) 20.5 (18-24) years	**AUDIT-C** (Frequency of drinking, quantity consumed per occasion & frequency of HED)	**HED:** consumption of five standard drinks (≥ 60g) alcohol per single occasion **Binge drinking:** women (4) & men (5) units in a session at least/month **Hazardous or harmful drinking (HHD):** ≥ 5 on AUDIT-C	**Unconditional multivariable log. Reg.:** **HHD:** 19.1% (24.3%, male; 12.9%, women) **Men:** high sexually permissive attitudes, not poor, multiple sexual partners, tobacco & illicit drug use were associated with HHD. **Women:** high (HIV risk perception, sexually permissive attitudes & peer pressure (lifestyle), spending more nights away in a week, tobacco & illicit drug use were associated with HHD.
**Tomkins et al., 2007**<br><br>**Russia** (Izhevsk)	**Cross-sectional** (Men controls in a case-control study of premature male mortality, Izhevsk) **Population-based** (Urban)	1750 men (100% men) ? (25-54 years)	**QFQs**	**Hazardous drinking-HD:** (any of these in past year) Having drunk surrogates; having been on zapoi; having frequent hangovers (once/month or more); having drunk spirits daily.	**Logistic regression:** Drinking spirits (79%) & surrogates (8%) at least sometimes in the past year. Drinking spirits (25%) & surrogates (4%) at least weekly & 10% had had episode of zapoi in past year. Education, lowest level in men (associated with indicators of HD. Indicators HD were also associated with being unemployed & levels of household wealth/amenities.
**Weiser et al., 2006**<br><br>**Botswana**	**Cross-sectional** (5 districts of Botswana with highest number of HIV-infected individuals) **Population-based** (Urban/Rural)	1,268 adults (48% men) 28.8 (18-49 years)	**QFQs**	**Heavy alcohol consumption (HD):** > 14 drinks/wk for women, & > 21 drinks/wk for men) **Problem drinking** (8–14, women, 15–21 for men) &	**Heavy drinking:** 31%, men & 17%, women **Problem drinking:** 39% of men, (79% met HD) & 25 % of women, (69% met HD). Correlates of HD: intergenerational relationships (age gap 10 year), male gender, higher education, & living with a sexual partner. A dose-response relationship was seen between alcohol use & risky sexual behaviors, with moderate drinkers at lower risk than both problem & heavy drinkers.
**Zavos et al., 2015**<br><br>**Sri Lanka** (Colombo district)	**Cross-sectional** (Data from the Colombo Twin And Singleton Study, CoTASS) **Population-based** (Urban/semi-urban areas)	6014 Sample (twins/48% & Singleton/46% of male) Mean age: 34 (twins) & 43 (singleton) (> 16 years)	**CIDI** Alcohol use: ever had of 12 drinks at any time in life	**Alcohol abuse & dependence:** Definition of CIDI (DSM-IV criteria)	**Robust cluster command:** **12-month prevalence of alcohol use:** 22.7% **Lifetime AA & AD in men:** 6.2% & 4.0% Lifetime AA & AD was associated with greater prevalence of nicotine dependence, depression, anxiety & PTSD (only for AD). Lower standard of living was associated with alcohol use & AD but not with AA

For peer review only

**Lo et al., 2013**<br><br>**Kenya**<br>(Nyanza Province)	**Prospective study** (Longitudinal database of demographic & health census data in western Kenya)<br>**Population-based** (Rural area)<br>Secondary data	72,292 individuals (43.1% men)<br>? (≥ 18 years)	Questions on (ever use & current use)	**1) % of time drunk when drinking in past 30 days:** (Did not get drunk, Drunk < 50%, Drunk 50%+)<br>**2) Days drinking/month:** (1-7, 8-17 & 18+)<br>**3) Problem drinking:** drinking ≥ 8 days/past 30 days & were drunk at least 50% of times they drank	**Crude and adjusted logistic regression:**<br>Overall, ever drinking was 20.7%<br>Drinking/past 30 days was 7.3% & 34.6%.<br>(60.3%, being drunk on ≥ 50%+) of all drinking occasions)<br>Alcohol use increased with decreasing socio-economic status & oldest women.<br>Current smoking, men, all age groups ≥ 40 & highest wealth index quintile (significantly associated with problem drinking).
**Pillai et al., 2013**<br><br>**India**<br>(Northern Goa)	**Cross-sectional**<br><br>**Population-based survey**<br>(rural & urban communities)	2641 men<br>(100% men)<br>? (18-49 years)	**QFQs** & Drunkenness	**Current drinkers:**<br>**low risk** (< 40 g/d),<br>**medium risk** (40–60 g/d),<br>& high risk (> 60 g/d)<br>**HED**: ≥ 60g in a single occasion in past 12 months<br>**Drunkenness**: times drank to feel drunk in last 1 year (< monthly, ≥ monthly but < weekly), & ≥ weekly)	**Logistic regression + Moderating effect:**<br>Of current drinkers:<br>**HED:** 28.6 % (rural 31 %; urban 27.2 %) & Drunkenness: 33.7% (rural 30.5 %; urban 35.5 %) ➔ monthly or more frequent<br>**HED**: associated with older age, being separated, lower education, & LSI<br>Weekly or more frequent drunkenness was associated only with rural residence.<br>All three risky drinking patterns were associated with CMDs, sexual risk, intimate partner violence, acute alcohol-related consequences, & AD.
**Sau, 2017**<br><br>**India**<br>(West Bengal)	**Cross-sectional**<br>(Adult population of the state of West Bengal, Gram Panchayat, GP)<br>**Community-based**	99 adults<br>(54.5% men)<br>38.62 (≥ 18) years	**AUDIT**	**AUDIT (WHO scoring):**<br>≥ 8 (hazardous/harmful use & possible AD)<br>0-7 (Zone-I): Low risk drinking/abstinence risk<br>8-15 (Zone-II): Alcohol use in excess of low-risk,<br>16-19 (Zone-III): Harmful & hazardous drinking &<br>20-40 (Zone-IV): Alcohol dependence risk level.	**Intraclass correlation, chi-square test, logistic regression & Bootstrapping:**<br>**Mean AUDIT** score was 7.11 (5.55 to 8.74)<br>**Low risk drinking/abstinence:** 65.5% & **Alcohol use in excess of low risk:** 17.6%, & **Harmful & hazardous drinking:** 8.5% & **Alcohol dependence** was 8.4%<br>**Hazardous, harmful use & AD** was 34.5%<br>Male gender and being employed were more prone to become high risk level drinker.
**Takahashi et al., 2017**<br><br>**Kenya**<br>(Western)	**Cross-sectional**<br>(Adults residing in Ikolomani Sub-county, Kakamega)<br>**Community-based**	478 participants<br>(41.4% men)<br>41 (18–65) years	**AUDIT**<br><br>**Type** & **QFQs**	**Current drinkers:** use of any alcohol in the last month,<br>Hazardous/high-risk drinkers:	**Univariate & multivariate analyses:**<br>Current & hazardous/high-risk alcohol use: 31.7% (men 54.6%; 8.9%, women) vs 28.7%<br>More than one drinker in the family, ≥ 5 drinker friends & positive attitude towards alcohol intake were positively associated with

For peer review only

	(Rural)			AUDIT score of ≥ 8	current alcohol drinking status, and with hazardous/high-risk alcohol consumption.<br>Women were less likely to be current drinkers & hazardous/high-risk drinkers.
**Yeung et al., 2015**<br><br>**Cambodia**<br>(Puok district)	**Mixed methods**<br>(Adults living in 2 selected rural communities<br>**Community-based**<br>Rural communities	120 households<br>(49.0% men)<br>? (≥ 18 years)	**AUDIT-C-Q**<br>**QFQs**<br>8 FGDs<br>**NIAAA**<br>Guidelines	**AUD:** cut off score of ≥ 5 in men & ≥ 4 in women<br>HED: ≥ 6 drinks in a single sitting at least monthly (NIAAA)	**χ2, Welch 2-sample t-test, Log. Regression**<br>**AUD & HED:** 4% and 31%, respectively.<br>**AUD** (47% men, 5% women (P < 0.0001); **HED** (47% men, 15% women (P = 0 .0001).<br>Male sex, younger age (decreasing age), and increasing income (higher monthly) were significant risk factors for AUD and HED
**Alem et al., 1999**<br><br>**Ethiopia**<br>(Butajira)	**Cross-sectional**<br>(Demographic surveillance site)<br>**Community-based**<br>(mostly rural)	12531 residents<br>(50% male)<br>? (≥ 15 years)	5-item questionnaire (questions for alcohol user vs non-users &<br>**GAGE-4**<br>items)	**Problem drinking (PD):** consumption beyond safe limits (≥ 2 positive responses on CAGE).<br>Cigarettes smoked daily: 1-3=mild,<br>4-9=moderate,<br>>9= heavy	**Chi-square statistics:**<br>Current drinkers: 23.4 % (15% women & 36% for men).<br>PD, 15.7% in alcohol users; overall PD, 3.7% (7.5% men & 0.90% women).<br>(2.4% in urban dwellers & 4.0% in rural)<br>Christian religion, male sex, ethnically non-Gurage, & smoking (associated with PD in both sexes). Marital status (divorced men), mental distress & income were associated with PD only in men & being widowed & divorced in women
**Kebede and Alem, 1999**<br><br>**Ethiopia**<br>(Addis Ababa)	**Cross-sectional**<br>Adults in Addis Ababa<br>**Population-based**<br>(Urban residents)	10203 adults<br>(45.1% men)<br>? (≥ 15 years)	**CAGE**<br>(1st stage) &<br><br>**CIDI**<br>(2nd stage)	**Problem drinking (PD):**<br>≥ 2 of on CAGE items, &<br>**Alcohol dependence (AD):** CIDI (ICD-10 diagnoses)	**Bivariate and multivariate analysis:**<br>**PD** was 2.7%, lifetime AD, 1.0% (1.9% in male & 0.1% for women) & one-month AD, 0.8% (1.5% for men and 0.06% for women).<br>PD increased with increasing age<br>PD decreased with increasing educational attainment. 39% increased risk of PD with employment & female sex had a 96% decreased risk of PD. Only sex (women had an 84% less risk to be AD compared to men).
**Nalwadda et al., 2018**<br><br>**Uganda**<br>(Kamuli District)	**Cross-sectional**<br>(Men attending PHC & men in population; part of the PRIME project)<br>**Community-based** & facility-based<br>(Rural district)	351 men<br>(Community study)<br>778 men<br>(Facility Survey)<br>(100% men)<br>? (≥ 18 years)	**AUDIT**<br>(10 item)	**AUD definition (AUDIT):**<br>**Hazardous** (score 8–15),<br>**Harmful** (score 16–19) or<br>**Dependent** (score ≥ 20) drinking behaviors (cut-offs defined by WHO)	**Kruskal–Wallis test & Fisher's exact test:**<br>**Community study**: 4.1% of all men were AUDIT+ (AUD); (2.9% hazardous, 0.7% harmful & 0.5% with dependent drinking)<br>**Facility study:** 5.7% of all men were AUDIT+; (4.5% hazardous; 0.6%, harmful)<br>47.5% AUDIT+ men: AUD ruined their lives<br>55.0% AUDIT+ men did not seek treatment<br>AUDIT scores were higher among older men, men with paid/self-employment status and higher PHQ-9 score (P < 0.05).

For peer review only - http://bmjopen.bmj.com/site/about/guidelines.xhtml

**Rathod et al., 2018**<br><br>**Nepal**<br>(Central district)	**Cross-sectional**<br>(Adults in Chitwan District; part of PRIME consortium)<br>**Population-based**<br>Secondary analysis	3482 sample<br>(36% men)<br>? (18-88) years	**AUDIT**<br>(10-item)	**Abstinent:** Score of 0,<br>**Recent (12 months) consumer:** Score of ≥ 1<br>Score of ≥ 8: **positive screen for AUD,**<br>8–15: **hazardous** drinking,<br>16-19: **harmful** drinking &<br>≥ 20: **dependent drinking**	**X^2 test & Negative binomial regression:**<br>23.8% of male screened AUD+ (AUD)<br>5.3% of female drinkers screened AUD+<br>Men with AUD, 38% spoke to another person about their problems & 80% had internalized stigma.<br>Being a drinker was associated with age, religion, caste, education, occupation & tobacco use. AUDIT scores were associated with age, caste, marital status, occupation, tobacco use, depression, functional status & suicidal ideation.
**Teferra et al., 2016**<br><br>**Ethiopia**<br>(Sodo district, southern Ethiopia)	**Cross-sectional**<br>(Adults from rural Sodo district (PRIME survey)<br>**Community-based**<br>(Rural residents)	1500 adults<br>(50.5% men)<br>? (≥ 18 years)	**FAST**<br>**Kessler-10**<br>(psychological distress)<br>**LTE** (adverse life events)	**Hazardous alcohol use (HD):**<br>FAST score ≥ 3 out of 16	**Exploratory multivariable log. regression:**<br>Prevalence of **hazardous alcohol use**: 21%; (31% in males & 10.4 % in females)<br>Factors associated with HD were being male, increasing age, having experienced ≥ 1 stressful/adverse life events, & severe psychological distress (AOR = 2.96).<br>High social support was protective from hazardous alcohol use (AOR = 0.41)
**Zewdu et al., 2019**<br><br>**Ethiopia**<br>(South, Sodo district)	**Cross-sectional**<br>(Adults who lived for at least 6 months in Sodo dist)<br>**Community-based**<br>(Rural district)	1485 individuals<br>(45.7% men)<br>39 (≥ 18) years	**AUDIT-10**	**Probable AUD:** score ≥ 8<br>8–15 (medium level of alcohol problem)<br>≥ 16 (high level of alcohol problems)<br>≥ 20 (possible alcohol dependence-AD)<br>**Binge drinking (BD):** drinking ≥ 6 alcoholic drinks on a single occasion	**Poisson regression with robust variance:**<br>Weighted prevalence of AUD was 13.9%; 25.8% in men & 2.4% women, P < 0.001<br>(Hazardous/harmful/AD: 9.9%/2.2%/1.8%)<br>23.3% had BD<br>87.0% of cases scored ≥ 16 had never sought help & 70.0% had high internalized stigma<br>AUD were associated & more prevalent in men (aPR = 7.7), farmers, traders, & daily laborers. People with AUD had increased total depressive symptom score & higher total disability score, more stressful life events & suicidal ideation (aPR 1.5)
**Getachew et al., 2017**<br><br>**Ethiopia**	**Cross-sectional**<br>(2015 national noncommunicable diseases STEPS survey)<br>**Community-based**<br>(Urban,27.4% & rural, 72.58%)	9,800 participants<br>(40.6% men)<br>34.5 (15-69) years	**QFQs**<br>(WHO STEPS questionnaire)	**Current drinkers**: alcohol use a month before survey<br>Lifetime alcohol use: ever<br>**Past 12-month users:**<br>HED/Excessive Alcohol Consumption: drinking ≥ 6 drinks in men & ≥ 4 in women on one occasion.	**Logistic regression:**<br>Prevalence of lifetime alcohol consumption & current drinkers was 49.3% & 40.7%.<br>Among ever drinkers, 89.6% drank alcohol in the past 12-months.<br>**HED:** 12.4% (20.5% males & 2.7% females)<br>Factors independently associated with HED, were male sex, rural residence), married, and current tobacco smoking (AOR=2.87).

For peer review only

For peer review only - http://bmjopen.bmj.com/site/about/guidelines.xhtml

**Abd Rashid et al., 2021**<br><br>**Malaysia**<br>(Sabah Borneo Island)	**Cross-sectional**<br>(People in Bingkor who consumed alcohol in the past 12 months)<br>(urban setting)	**363 participants**<br>(51.5% men)<br>? (≥ 26 years old, 90.6%)	**AUDIT**<br>(hazardous alcohol use)<br>**MINI V5.0**<br>based on DSM-IV<br>(psychiatric morbidity)	**Hazardous alcohol use:**<br>AUDIT scores of ≥8	**Multiple logistic regression analysis**<br>80.2% admitted having consumed alcohol.<br>Preferred type of drink: beer (67.8%), tuak tapai (61.7%), wine (31.7%), tuak beras and whisky (16.8%), imported alcohol drinks such as vodka (9.1%) and 'samsu' (3.9%).<br>41% of participants (high risk for hazardous alcohol use) vs 39.1% (with low risk of hazardous alcohol use).<br>Being male & being a non- Muslim had a higher risk to develop hazardous alcohol use (OR = 3.313 & 3.834 respectively).<br>Having a current obsessive- compulsive disorder was associated with a higher risk of hazardous alcohol use (OR = 0.265).
**Assanangkornchai et al., 2020**<br><br>**Thailand**	**Cross-sectional**<br><br>(Thailand's 5th National Health Examination Survey, NHES-5, 2014)<br>(urban/53.6%, rural/46.4%)	**13177 participants**<br>(49.2% men)<br>46.7 (> 20 years)	**AUDIT**<br>(for AUD)<br>**MINI, Thai version 5.0.0**<br>(for MDE)	**AUD**: non-problem drinkers (0–7), hazardous drinkers (8–15), and harmful-dependent drinkers (16– 40) on AUDIT<br>**MDE**: defined according to DSM-IV criteria	**Multinomial logistic regression:**<br>10.3% and 1.9% hazardous drinkers and harmful-dependent drinkers, respectively<br>2.5% met the criteria for MDE in the past 12 months before the survey.<br>Approximately 20% were current smokers.<br>Associations between MDE and either hazardous (HD) or harmful dependent drinking (HDD) were strongest among those in third tercile (highest/wealthiest) of wealth index, first tercile (lowest/poorst), secondary school level of education or above, living in urban areas, & those who are employed.
**Ding et al., 2020**<br><br>**China**	**Cross-sectional**<br>(China Health and Retirement Longitudinal Study, 2011– 2012)<br>**Community based**<br>(Urban/40.5%, Rural/59.5%)	**17,302 subjects**<br>(49.30% men)<br>59.67 (aged ≥ 45 years)	**QFQ**<br>(for alcohol use)	**Heavy drinking:** >14 drinks per week (males) & >7 drinks per week for females	**Binary & multinomial logistic regressions**<br>Overall prevalence of heavy drinking, obesity, current smoking, and physical inactivity were 7.23%, 11.53%, 27.46%, and 44.06%, respectively.<br>Compared with healthy subjects (no hypertension, high cholesterol, or diabetes), newly detected hypertensive patients were more likely to smoke (OR, 1.34), be heavy drinkers (1.45), and be obese (1.94).
**Hernandez-Vasquez et al., 2022**<br><br>**Peru**	**Cross-sectional**<br>[(2018 Peruvian Demographic & Family Health Survey (ENDES)]	**32,020 people (analysis)**<br>(42.8% men)<br>? (≥ 18 years old)	**SAMHSA definition (RSOD):** Bing Drinking (BD)	**BD:** consumption of 5 & 4 or more alcoholic beverages on the same occasion for men & and women, respectively, in the	**Poisson's family GLMs with link function (log) were used for (cPR and aPR).**<br>BD was found in 22.4%. Men (32.6%) presented a higher consumption pattern than women (12.8%).<br>Men aged 25–44 had a higher probability of BD (aPR: 1.28). The age group of ≥ 60 was associated with a lower probability (aPR:

For peer review only

For peer review only - http://bmjopen.bmj.com/site/about/guidelines.xhtml

	**A Population-Based Analysis** (Urban/65.7%, Rural/34.5%)			last 30 days before the survey	0.70) of BD compared to younger group of men (18-24 years). Women aged ≥ 60 years was associated with a lower probability of BD (aPR: 0.24). Secondary (aPR: 2.01) or higher level of education (aPR: 2.04) was a factor associated with a higher prevalence of BD in men
**Jadnanansing et al., 2021**<br><br>**Suriname**	**Cross-sectional** [(populations in both region (rural/Nickerie & urban/Paramaribo)]	**2863 participants** (43% men)<br>39.97 years (?)	**AUDIT & ASSIST:** (for AUD)	**Risky alcohol use:** A score of > 7 on AUDIT	**Simple & Multivariable logistic regression**<br>AUD is 6.4% in urban area & 5.8% in rural area. Men had highest addiction risk at about 16% compared with 2% for females.<br>A treatment gap of 50% was found for AUDs in the rural area (64% urban area).<br>Married persons are significantly less likely to become alcoholic than singles and other groups in urban area.<br>In both areas, higher education was associated with a lower probability of alcohol abuse and dependence, while handymen showed a higher odd.
**Jirapramukpitak et al., 2008**<br><br>**Thailand** (Bangkok)	**Cross-sectional** (Suburban community of Bangkok in 2003 and 2004)	**1052 residents** (46.3%)<br>? (16–25 years	**AUDIT** (hazardou or harmful drinking) & **DIS** (illicit drug use-Diagnostic Interview Schedule)	**Illicit drug use:** assessed with self-report adapted from (DIS) and **Hazardous/harmful drinking:** with AUDIT **Migration**: the occasion when a young person born in amore rural area moves for the first time into Greater Bangkok.	**Multivariate analysis (logistic regression)**<br>10.9% (82 males and 17 females) had illicit drug use and 24.3% (179 males and 62 females) hazardous and harmful drinking.<br>Hazardous/harmful drinking was associated independently with being late migrants, who moved at the age of 15 or older.
**Moreira et al., 1998**<br><br>**Brazil**	**Cross-sectional** (Adults in Porto Alegre, a city in southern Brazil)	**1099 individuals** (45% men)<br>? (18-88 years old)	**QFQs** (type, quantity, & frequency**)** **& CAGE questionnaire**	**Heavy drinking**: average consumption of 30g/day or more, a level of exposure associated with health risks **Dependence:** Two positive answers to the CAGE questionnaire	**Simple/multiple linear & logstic regression**<br>24.1% had never drunk alcohol (9.0%/men & 36.5%/women).<br>29.3% of men & 4.2% of women were heavy drinkers. 16% & 4.0% were CAGE+, respectively.<br>Consumption of 30 g/day ethanol was associated with increases of 1.5 & 2.3 mmHg in DBP & SBP for men and 2.1 and 3.2 mmHg for women respectively.<br>Prevalence of HTN was higher among those ingesting ≥ 30 g/day (odds ratio = 2.9).
**Oancea et al., 2021**	**Cross-sectional**	**59,399 individuals** (47.6% men)	**NIAAA definitions**	**BD (NIAAA):** a pattern of drinking that brings BAC	**Weighted & adjusted logisitic reggression**

For peer review only - http://bmjopen.bmj.com/site/about/guidelines.xhtml

**Brazil**	(2013 Brazilian National Health Survey)	weighted median age, 40.53 (18-60+ years)	(Binge drinking/BD & Heavy drinking/HD**)**	levels to at least 0.08 g/dl. (4 drinks for women & 5 for men in about 2hrs)<br>**HD**: ≥ 5 days of BD episodes in a month is defined as the HD index.	14.8% were current smokers, 13.8% were binge drinkers & 3.2% were heavy drinkers.<br>Self-reported current depression/SRCD,7.6%<br>There was significant weighted & adjusted increase in the odds of SRCD among young adults (18–39 years) who were binge drinkers compared to those who were not binge drinkers (AOR = 1.32).
**Pengpid et al., 2021**<br><br>**South Africa**	**Cross-sectional**<br>(National survey of all household members, who resided in that household the previous night)<br>(Rural informal/ 26%, Rural farms/ 5%, Urban/69%)	**39,210 persons**<br>(48.3% men)<br>Median age,34 (IQR,25-48)<br>(15 years & older)	**AUDIT**<br>(Hazardous, harmful, or dependent alcohol use (HHDA):<br>**ASSIST** (Drug use in the past 3 months)<br>**K10** (Kessler Psychological Distress Scale)	**HHDA:**<br>Adults (≥ 20 yrs): cut-off score is ≥ 8 on AUDIT & Adolescents (15–19 years): 5 or more on AUDIT<br>**Drug use in past 3 months**: Any drug used in past 3 months was coded as 1 and never as 0'.<br>**Psychological distress:** scores ≥ 20 on (K10)	**Unadjusted & adjusted logisric regression**<br>10.3% engaged in HHDA, 16.5% (males) & 4.6% (females). Past 3-month drug use was 8.6%, 13.3% (males) & 4.1% (females).<br>Men of middle age (25-34) with higher education, urban residence, drug use and psychological distress were positively associated with HHDA. Women of middle age (25-34) and mixed race, residing on rural farms and urban areas, drug use and psychological distress were positively associated & older age (≥55) & Indians or Asians were negatively associated with HHDA.
**Prais et al., 2008**<br><br>**Brazil**<br>(Metropolitan area of Belo Horizonte, & Bambuí)	**Cross-sectional**<br>(elderly Brazilian men, ≥ 60 years were the study population)<br>**Population based**<br>(urbann setting)	**685 residents in RMBH & 642 in Bambuí**<br>(100% men)<br>**Mean age:**<br>68.8 yrs (RMBH)<br>69.0 yrs (Bambuí)<br>(≥ 60 years)	**RSOD criteria**<br>(for BD)	**Binge Drinking:**<br>Consumption of five or more alcoholic drinks on a single occasion in the last 30 days.	**Multivariate analyses (PR estimated by Robust Poisson Regression)**<br>Prevalence of BD was two times higher among residents in metropolitan area of Belo Horizonte (27.1%) than in Bambuí (13.7%).<br>**RMBH:** higher schooling level [8+ yrs] (PR = 1.55), worse self-rated health [reasonable, bad, or very bad] (PR = 0.62) and inability to perform activities of daily living (PR = 0.12) remained significantly associated with BD.<br>**Bambuí**: worse self-rated health (PR = 0.57) and being divorced or separated (PR = 2.49) remained significantly associated with BD.
**Trangenstein et al., 2018**<br><br>**South Africa**<br>(Tshwane Metropole)	**Cross-sectional**<br>(Adults who used alcohol in the past six months).<br>(Data from South African arm of the multi-country International	**713 adults**<br>(65.8% men)<br>36.3 (18-65 years)	**International Alcohol Control (IAC) questionnair:**<br>(Asks QFQs over past six months)	**Heavy Drinking (HD):**<br>consuming ≥ 96g of absolute alcohol (AA) (roughly 8 standard drinks, or 120 ml) for men or ≥ 72g (6 standard drinks, or 90 ml) for women at least monthly.	**Multivariate logistic regression**<br>HD was 53%. HD did not vary by gender (F1, 19 = 3.96, p = 0.06), age, race/ethnicity, or total annual personal income. Bivariate analyses revealed that HD differed by marital status (F2.48, 47.11 =3.09, p = 0.04).<br>Adjusting for marital status & primary container size, single persons were found to have substantially higher odds of HD.

For peer review only - http://bmjopen.bmj.com/site/about/guidelines.xhtml

	Alcohol Control, IAC study) (urban setting)			**Low risk**: occasions that did not include HD	
**Vellios and Van Walbeek, 2018**<br><br>**South Africa**	**Cross-sectional** (data from wave 4 of the 2014-2015 National Income Dynamics Study, NIDS) (rural/35.4%, urban/64.6%)	**22,752 (wave 4)** (46.8% men) ? (≥ 15 years)	**QFQs:**1) How often do you drink alcohol? 2) On a day you have an alcoholic drink, how many standard drinks do you usually have?	**Binge drinker:** use of ≥5 standard drinks on an average drinking day.<br>**Current drinker**: any option from (iii) I drink alcohol very rarely, (iv) Less than once a week, (v) On 1 or 2 days a week, (vi) On 3 or 4 days a week, (vii) On 5 or 6 days a week, & (viii) Every day.	**Multiple logit regressions**<br>Current alcohol use (any amount) in 2014 - 2015 was reported by 33.1% of the population (47.7% males, 20.2% females). Of current drinkers, 43.0% reported BD (48.2% males, 32.4% females).<br>Self-reported BD as a proportion of the total population was 14.1% (22.8% M, 6.4% F).<br>Self-reported BD was highest among males & females aged 25-34 years (49.4%).<br>Smoking cigarettes for both genders substantially increased the likelihood of drinking any amount (aOR: 5.08 males, 4.80 females) and of BD (aOR: 1.53 for males, 3.36 for females).<br>As a percentage of total population, people aged 25-34 years were more likely to binge than aged 15-24 years, for both males (OR 1.44) and females (OR 1.49). Compared with married males, males living with a partner (OR 1.58) or who were single (OR 1.74) were more likely to BD.<br>Compared with married females, females living with a partner (OR 1.68) or single (OR 1.41) were more likely to BD.<br>Having children in the house slightly increased the probability of BD for males (OR 1.21), but not for females.
**Aremu et al., 2021**<br><br>**Nigeria** (urban poor people in Ibadan)	**Cross-sectional** (two selected urban poor communities in Ibadan, Nigeria)	**500 Participants** (29.4% men) 35.36 (18-65 years)	**Modified version of WHO STEPS instrument**	**Alcohol consumers:** Ever consumed, Current consumers (12mo.) Current & **frequent consumers within 30 days (low, medium, and high)**<br>**Low consumers:** consuming < 4 (men) & < 2 (women) SDs/occasion<br>**Medium:** 4-6 (men) & 2-4 (women) SDs per occasion<br>**High:** > 6 (men) & > 4 (women) SDs per occasion	**Descriptive & inferential statistics (X^2)**<br>29.0% had consumed alcohol either in past or present, 17.8% consumed alcohol within last one year, 15.8% were current consumer of alcohol & 13.6% were frequent consumers who had taken alcohol within 30 days (11.6% low consumers, 1.2% medium consumers and 0.8% high consumers).<br>More male (53.1%) reported to have ever consumed alcohol compared to female (46.9%). 62.3% of non-current alcohol users was female & 37.7% were male. 59.3% of respondents not currently consuming alcohol were currently married (30.3% were not).<br>74.1% of the low consumers were male, 66.7% medium consumers were females, & 75.0% of high alcohol consumers were male

For peer review only

For peer review only - http://bmjopen.bmj.com/site/about/guidelines.xhtml

**Bonnechère et al., 2022**<br><br>**Burkina Faso**	**Cross- sectional** (Data from the 2013 Burkina Faso WHO STEPwise) Rural (75.1%), Urban (24.9%) **Population- based**	**4692 individuals** (45.7% men) ? (25–64 years)	Quantity/Frequency Questions **(QFQs)**	**4 levels of consumption:** No consumption (None) **Low**: intake of pure alcohol of <40g/day (men) & <20g for women **Mid**: 40-59.9g/day (men) & 20-39.9g for women **Abusive consumption:** ≥60g/day (M) & ≥40g (W) **Dependent variable:** mean alcohol consumption in the last 30 days.	**Multinomial logistic regression:** 3559 (75.8%) were not consuming any alcohol, 12.9% had low, 8.5% had mid and 2.7% had abusive alcohol consumption. Age was associated with any level of alcohol consumption with a gradient effect and older people having a higher level of consumption in comparison with no consumption. Tobacco consumption was significantly associated with alcohol intake with gradient effect, those with higher tobacco use being at higher risk of abusive alcohol intake. Sex is an important risk factor for abusive consumption with increased risk for men compared with women. Jobless people & housemaker was associated with a decreased risk of having abusive consumption.
**Dahal et al., 2021**<br><br>**Nepal** (Kathmandu district)	**Cross-sectional** (adults residing in municipalities of Kathmandu district for at least six months) **Community-based** (unplanned urbanization)	**245 participants** (47.3% men) **Mean age:** 41.19/male, & 40.91/female (18–69 years)	**WHO STEPS questionnaire (QFQs)**	**Current episodic heavy drinking (HED):** six or more drinks on any day in the past 30 days.	**Bivariate & multivariate analysis** 67.3% were lifetime abstainers. Prevalence of alcohol consumption in last 12 months was 31.0% & HED was 12.7%. Prevalence of current smoking, low intake of fruits & vegetables and low physical activity was found to be 22%, 93.9% and 10.2% respectively. 52.2% of participants were overweight/obese & prevalence of raised BP was 27.8%. Odds of alcohol consumption were higher among male (AOR: 2.78), employed (AOR: 2.30), & those who belonged to Chhetri (AOR: 2.83), Janajati (AOR: 6.18), Dalit and Madhesi, (AOR: 7.51) ethnic groups.
**Jonas et al., 2014**<br><br>**India** (rural Central India)	**Cross-sectional** (data from Central India Eye and Medical Study, CIEMS, in rural region of Central Maharashtra) **Population-based**	**4711(participated)** (46.5% men) 49.5 (30+ years)	AUDIT CESD 20-item FTND (smoking behavior)	**Harmful or hazardous drinking:** sum score of 8 or more on AUDIT **Clinical episode of major depression:** score of > 21 in the CES-D.	**Test of for association not performed** Alcohol consumption was 23.0%; 6.0% subjects had an AUDIT score ≥8 (hazardous drinking), & 4.63% subjects a score ≥ 13 (women) or ≥ 15 (men) (alcohol dependence)
**Olickal et al., 2021**	**Cross-sectional**	**316 adult men** (100% men)	**WHO AUDIT**	**Hazardous alcohol**: AUDIT score of 8–15	**Independent t-test, One-way ANOVA & Kruskal Wallis test, Multiple linear regression**

For peer review only - http://bmjopen.bmj.com/site/about/guidelines.xhtml
For peer review only

**India** (Puducherry, South India)	(adult men aged above 18 years in Puducherry, South India) **Community-based** (rural/50%, urban/50%)	45.2 (≥18 years)	**WHO QoL-BREF questionnaire**	**Harmful alcohol use**: AUDIT score of 16–19 **Probable alcohol dependence**: score of 20 or more on AUDIT **High risk:** A score eight and above on AUDIT **QoL:** A higher score is indicative of a better QoL in each of the domains.	Mean (SD) AUDIT score was 13.2 (6.7). Probable dependence was 8.2%, & hazardous or harmful use was 27.8%. Overall mean score of QoL was lower among alcohol users compared to non-alcohol users (50.7 vs 63.5) QoL score was significantly lower among alcohol users (also in all domains). High-risk alcohol users and urban residence had 11.2 & 4.1 less QoL scores respectively and educated had 7 more QoL scores compared to the reference category.
**Olickal et al., 2022** **India** (Puducherry, South India)	**Cross-sectional & Qualitative design** (Mixed design) (All men ≥ 18 years from urban & rural field practice areas of a tertiary care centre in Puducherry, South India)	**316 subjects** (100% men) 45.2 (19-60+ years)	**WHO AUDIT** Discussion guide for FGD	**Probable alcohol dependence:** A total score of ≥20 on AUDIT	**A log binomial regression (prevalence ratio) & Manual content analysis** Alcohol use was 38%, 40% were daily users) (34% in rural to 42% in urban areas) Among alcohol users, 21.7% were probable dependents on alcohol. Older individuals had a 2.9 times higher risk of alcohol use than young individuals (<30). No formal education was a high-risk factor for alcohol use, compared to educated. Individuals residing in rural areas (APR = 1.05), self-reported comorbidities (APR = 1.21), family history of alcohol use (APR = 2.42) and tobacco use (APR = 2.42) were significantly associated with alcohol use.
**Sarma et al., 2019** **India** (Kerala, South India)	**Cross-sectional** [(all individuals between 18-69 years old were eligible, in both rural & urban (49.3%) areas)] **Community-based**	**12,012 adults** (37% men) 42.5 (18–69 years)	**WHO STEPS instrument GPAQ** (Global Physical Activity Questionnaire) **Anthropometric measurements**	**Current alcohol use:** intake of at least one standard drink of alcohol in the past 30 days. **Current tobacco use**: use of any form of tobacco within the past 30 days. **Raised Blood Pressure (BP):** BP of ≥140/≥90 mm Hg, or if the person is currently using antihypertensive medication.	**Weighted means, Percentages with 95% CI, & variance inflation applied** Current use of tobacco & alcohol in men was 20.3% & 28.9% respectively. The overall prevalence of raised BP was 30.4%.

For peer review only

For peer review only - http://bmjopen.bmj.com/site/about/guidelines.xhtml

**Endashaw Hareru et al., 2022**<br><br>**Ethiopia**<br>(Dilla town)	**Cross-sectional**<br>(Residents of Dilla town, Gedeo zone, Southern Ethiopia with age of ≥ 18 years)<br>**Community-based**	**666 participants**<br>(70% men)<br>Mean: 33.3 years<br>(≥ 18 years)	**AIDIT:** AUD<br>**Kessler Psychological Distress Scale (K10):**<br>**ASSIST 2.0:** current and lifetime substance use	**AUD:** AUDIT score of ≥ 8	**Bivariate & multivariate binary logistic regression analysis**<br>AUD during the past year was 30.6%.<br>Being male (AOR = 8.33), age of less than 33 years old (AOR = 1.78), current cigarette smoking (AOR = 2.49), current khat chewing (AOR = 6.23), high level of psychological distress (AOR = 7.69) and poor social support (AOR = 2.30) were significantly associated with AUD.
**Gutema et al., 2020**<br><br>**Ethiopia**<br>(Arba Minch HDSS)	**Cross-sectional**<br>(Adult residents of Arba Minch HDSS (nine Kebeles of Arba Minch Zuria District, Southern Ethiopia)<br>**Community-based**<br>(rural residents, 83.7%)	**3346 participants**<br>(50% men)<br>44.6 years<br>(25– 64 years)	**WHO STEPS instruments**<br>(alcohol use)<br>**SRQ-20**<br>(mental stress status)	**HED or Excessive Alcohol Consumption:** use of ≥ 6 drinks for men and ≥ 4 drinks for women on a single occasion at least once per month.<br>**Mental stress** (mild, moderate, and severe)	**Binary logistic regression**<br>Prevalence (HED) was 13.7%.<br>HED was associated with occupation (daily laborer: AOR 0.49; & housewives: AOR0.63 compared with farmers), wealth index (2nd quintiles: AOR 0.55 & 3rd quintiles: AOR 0.66) compared with 1st quintiles; & climatic zone (midland: AOR 1.80; highland: AOR 1.95 compared with lowland).<br>Tobacco use (AOR 4.28), & khat use (AOR 4.75) were also associated with HED.
**Legas et al., 2021**<br><br>**Ethiopia**<br>(South Gondar)	**Cross-sectional**<br>(adult residents whose age was 18 years and above in the South Gondar zone, 61.3% from urban areas)<br>**Community-based**	**848 (interviewed)**<br>(62.3% men)<br>? (≥ 18 years)	**AUDIT**-AUD<br>**PHQ-9**<br>**PSS**-Perceived stress scale questionnaire)<br>**Oslo social support scale**<br>**SPIN**-Social phobia inventory scale	**AUD**: score of 8 or above on AUDIT<br>**Depression:** A score of five or more on the PHQ-9	**Bivariate & multivariable logistic analysis**<br>AUD over the last 12-months was 23.7%.<br>16.50% had hazardous alcohol use, 5.2% had harmful alcohol use, and 2% had probable alcohol dependence.<br>Being male (AOR = 4.34), poor social support (AOR = 1.95), social phobia (AOR = 1.69), perceived high level of stress (AOR = 2.85), current cigarette smoking (AOR = 3.06) and comorbid depression (AOR = 1.81) were significantly associated with AUD.
**Wainberg et al., 2018**<br><br>**Mozambique**<br>(Zambézia Province)	**Cross-sectional**<br>(2014 survey)<br>(16 year or older female heads-of-household in Mozambique, Zambézia	**2,752 participants**<br>(no men, 100% female)<br>Median: 27 years<br>(16-62 years)	**AUDIT**<br>(Alcohol use)<br>**PHQ**<br>(Depression)	**Hazardous, harmful & high-risk drinkers:**<br>AUDIT scores > 4 (recommended cutoff for women)<br>**Depression:** A score of ≥ 10 on PHQ-8 (associated with clinical depression)	**Binomial logistic regression model:**<br>Overall prevalence of current alcohol consumption among female heads of hh was 15%. “hazardous drinkers” was 8%.<br>A positive depression screening (aOR: 2.20), death of a child (aOR: 2.44), & currently being pregnant (1.83) were associated with increased odds of hazardous drinking.<br>Being single (aOR: 0.48) & experiencing food insecurity (aOR:0.96) were associated with reduced odds of risky drinking.

For peer review only - http://bmjopen.bmj.com/site/about/guidelines.xhtml

	Province)<br>**Population-based**<br>/rural Mozambique				
**Bete et al., 2022**<br><br>**Ethiopia**<br>(Harari regional state)	**Cross-sectional**<br>(residents aged >18 years)<br>**Community-based**<br>(80.55% urban dwellers)	**955 adults**<br>(44.18% men)<br>42.28 years<br>(> 18 years)	**ASSIST**	**Current and ever substance users:** use of a specified substance (for non-medical purposes) in last 3 months and once in lifetime respectively	**Bi-variable & multivariate binary logistic regressions:**<br>The overall prevalence of **current alcohol use was (**8.24%) , tobacco use (14.5%), and khat use (63.30%).<br>The availability of alcohol, being unemployed, and being a current khat user were significantly associated with current alcohol use.
**Castelo Branco and de Vargas, 2023**<br><br>**Brazil**<br>(Northern Brazilian Amazon, state of Amapa)	**Cross-sectional**<br>(Karipunan respondents aged ≥ 15)<br>**Population-based**	**230 participants**<br>(51.3%, men)<br>? (≥ 15)	**AUDIT**	**hazardous/harmful alcohol use (Zones II-IV of AUDIT Score, problematic use):**<br>AUDIT score > 8.	**Fisher's exact test, & logistic regression: simple & multiple**<br>(Hosmer-Lemeshow test/C statistic, & Spearman correlation tests)<br>Prevalence of alcohol use: 70%; 59.6% (low-risk use), **38.3 (hazardous/harmful alcohol use)**, **& 2.2% (probable alcohol dependence). Overall, 40.5% had hazardous or harmful alcohol use**; **66.6%** were men, and **33.4%** were women.<br>Being male sex (AOR: 3.30), being Catholic (5.53) compared to Evangelical were associated with hazardous or harmful alcohol use.
**Rezaei et al., 2022**<br><br>**Iran**<br><br>**(national survey)**	**Cross-sectional**<br>(The STEPs survey in Iran, 2016)<br>**Population-based**<br>(urban residents, 71.09%)	**29,068** participants<br>(47.92%, men)<br>44.4 years (18 to 100 years)	**WHO's guidelines**<br>(WHO STEPS instrument)	**Current alcohol consumption:** drink alcohol in past 12 months<br>**Lifetime consumption:** ever drink alcohol in life.	**Univariate and multiple logistic regression analysis**<br>National level prevalence rates of lifetime and current alcohol consumption were 8.00% and 4.04% respectively.<br>The highest prevalence was reported among 25-34 years old.<br>Individuals of higher socioeconomic status consumed significantly greater levels of alcohol.<br>Current alcohol drinkers were 2 times more prone to trafic injury as compared to nondrinkers (ORadj: 2.0).
**Tegegne et al., 2023**<br><br>**Ethiopia**<br>**(national survey)**	**Cross-sectional**<br>(2016 Ethiopian Demographic and Health Surveys (EDHS)<br>**Population-based**<br>(80.29%, rural)	**12,688** participants<br>(100%, male)<br>30.92 years<br>(15-59 years)	**QFQs**	**Ever alcohol drinking:** drinks alcohol during the lifetime.	**Multilevel multinomial logistic regression**<br>Only Khat users (22.0%), only **Alcohol users (35.6%)**, and dual Alcohol and Khat users were (9.0%).<br>At the individual level: age group of 30-44 years (AOR: 1.75) and 45-59 years (AOR:1.62) are more likely to drink alcohol compared to 15-29 years.<br>Higher educational level (AOR: 1.4) compared to no education and having occupation (AOR:1.88) compared to people without occupation, increased the odds of drinking alcohol.<br>Divorced males (AOR: 0.5) compared to single males; Protestant (AOR: 0.01), Muslim (AOR: 0.04), and other religion follower

For peer review only - http://bmjopen.bmj.com/site/about/guidelines.xhtml

					males (AOR: 0.35) compared to Orthodox religion have lower likelihood of alcohol drinking.
**Wolde, 2023**<br><br>**Ethiopia** (South West Ethiopia)	**Cross-sectional** (elderly people living in towns in Ethiopia)<br>**Community-based**	**382 elderly people** (34.5%, male)<br>67 years<br>(≥ 60 years)	**AUDIT**<br>**ASSIST**	**Alcohol Use Disorder (AUD):** AUDIT score > 8.	**Bivariate and multivariable logistic regression model**<br>Magnitude of **AUD**, current alcohol use, and life-time alcohol use was **27.5%**, 52.4%, and 89.3%, respectively.<br>AUD was associated with cognitive impairment (AOR: 2.53), poor sleep quality (AOR: 2.67), chronic medical illness (AOR: 3.27), and suicidal ideation or attempt (AOR: 2.07).

Abrevations: **AA**: Alcohol Abuse; **AD**: Alcohol Dependence; **aPR**: adjusted Prevalence Ratio; **ASSIST**: Alcohol, Smoking, and Substance Involvement Screening Test; **AUD**: Alcohol Use Disorder; **AUDIT**: Alcohol Use Disorder Identification Test; **BD:** Binge drinking; **CAGE**: Cut down, Annoyed, Guilty feeling & Eye opener; **CESD:** Center for Epidemiologic Studies Depression Scale; **FAST**: Fast Alcohol Screening Test; **FTND:** Fagerstrom Test for Nicotine Dependence; **HD**: Heavy drinking; **HED**: Heavy Episodic Drinking; **wk**: week; **M:** men; **MDE:** Major Depressive Episode; **NIAAA**: National Institute on Alcohol Abuse and Alcoholism; **PHQ-9:** Patient Health Questionnaire-9 item; **PR:** Prevalence Ratio; **PRIME**: Programme for Improving Mental Healthcare; **QFQs**: quantity/frequency questionnaires; **QoL:** Quality of Life; **RMBH:** metropolitan region of Belo Horizonte; **RR:** response rate; **SD:** Standard drink; **W:** women; **yr.**: year; **?:** mean age or age range for subjects is not determined.

For peer review only

Supplementary File 5

Newcastle-Ottawa Scale (NOS) quality assessment summary for the study "A scoping review of assessment tools for, magnitudes of, and factors associated with problem drinking in population-based studies," 2023.

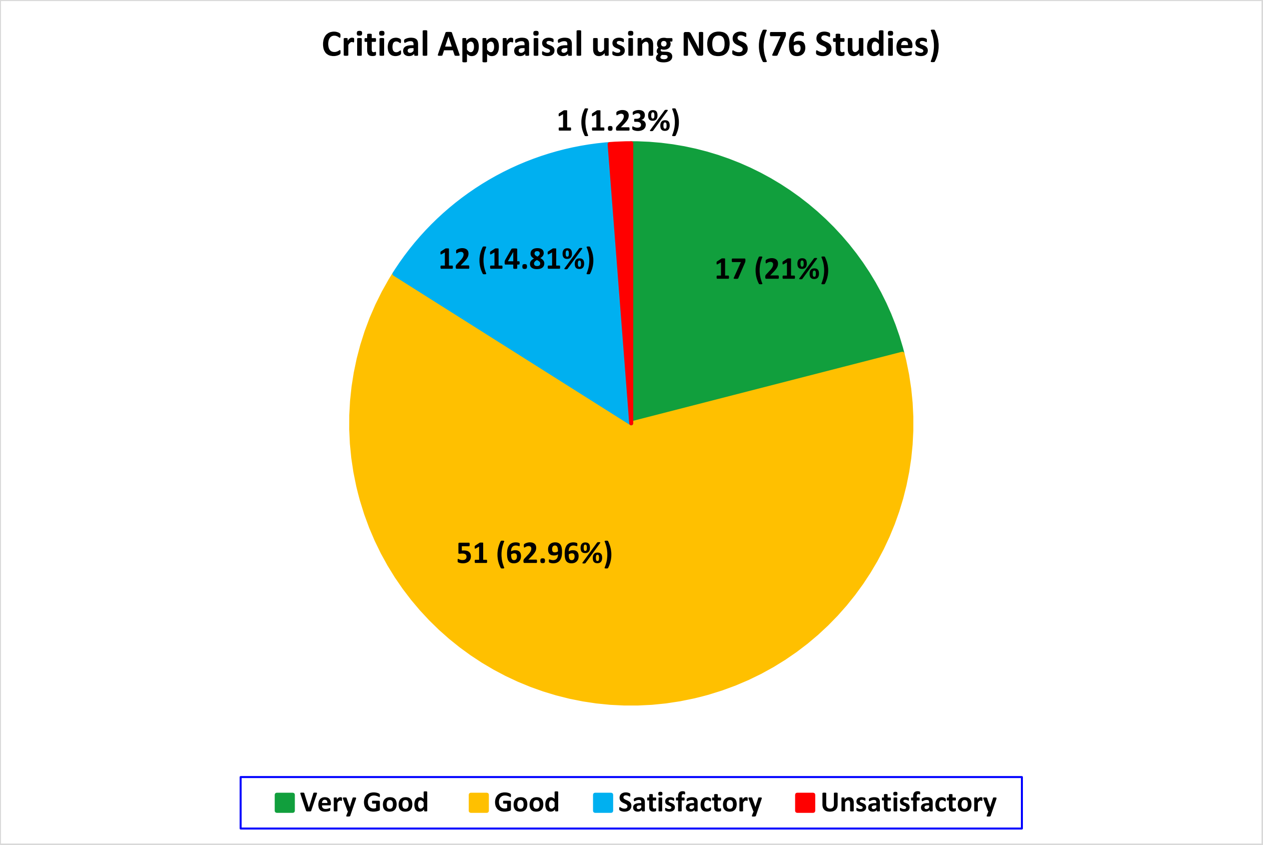

Figure: Newcastle-Ottawa Scale (NOS) quality assessment reports of studies for the study "A scoping review of assessment tools for, magnitudes of, and factors associated with problem drinking in population-based studies," 2023.

Table: Newcastle-Ottawa Scale (NOS) quality assessment- item level summary for "A scoping review of assessment tools for, magnitudes of, and factors associated with problem drinking in population-based studies," 2023.	Studies (81)
**Selection: (Maximum 5 points/scores/stars)**	
**1. Representativeness of the sample:**	
a. Truly representative of the average in the target population. * (all subjects/consecutive or random sampling)	**69**
b. Somewhat representative of the average in the target group. * (non-random sampling)	**12**
c. Selected group of users/convenience sample.	**0**
d. No description of the derivation of the included subjects (sampling strategy).	**0**
**2. Sample size:**	
a. Justified and satisfactory (including sample size calculation). * (1 score)	**44**
b. Not justified	**23**
c. No information provided	**14**
**3. Non-respondents:**	
a. Proportion of target sample recruited attains pre-specified target or basic summary of non-respondent characteristics in sampling frame recorded. *	**72**
b. Unsatisfactory recruitment rate, no summary data on non-respondents.	**02**
c. No information provided	**07**
**4. Ascertainment of the exposure (risk factor/disease) or screening/surveillance (measuremnt) tool:**	
a. Secure record (medical charts) or validated measurement (screening/surveillance) tool. **	**28**
b. Non-validated measurement tool, but the tool is available or described or Self report. *	**52**
c. No description of the measurement tool.	**01**
**Comparability: (Maximum 2 stars)**	

For peer review only

**1. Comparability of subjects in different outcome groups on the basis of design or analysis. Confounding factors controlled.**	
a. Data/results adjusted for relevant predictors/risk factors/confounders e.g., age, sex, marital status, job etc. **	**68**
b. Data/results not adjusted for all relevant confounders/risk factors/information not provided.	**13**
**Outcome: (Maximum 3 stars)**	
**1. Assessment of outcome:**	
a. Independent blind (structured) assessment. **	**14**
b. Record linkage. **	**0**
c. Self report. *	**67**
d. No description.	**0**
**2. Statistical test:**	
a.Statistical test used to analyse the data clearly described, appropriate and measures of association presented including confidence intervals and probability level (p value). *	**74**
b.Statistical test not appropriate, not described, or incomplete.	**07**

For peer review only

Research Checklist 1 (PRISMA-ScR Checklist)

Preferred Reporting Items for Systematic reviews and Meta-Analyses extension for Scoping Reviews (PRISMA-ScR) Checklist

SECTION	ITEM	PRISMA-ScR CHECKLIST ITEM	REPORTED ON PAGE #
**TITLE**			
Title	1	Identify the report as a scoping review.	1
**ABSTRACT**			
Structured summary	2	Provide a structured summary that includes (as applicable): background, objectives, eligibility criteria, sources of evidence, charting methods, results, and conclusions that relate to the review questions and objectives.	1
**INTRODUCTION**			
Rationale	3	Describe the rationale for the review in the context of what is already known. Explain why the review questions/objectives lend themselves to a scoping review approach.	6-7
Objectives	4	Provide an explicit statement of the questions and objectives being addressed with reference to their key elements (e.g., population or participants, concepts, and context) or other relevant key elements used to conceptualize the review questions and/or objectives.	7
**METHODS**			
Protocol and registration	5	Indicate whether a review protocol exists; state if and where it can be accessed (e.g., a Web address); and if available, provide registration information, including the registration number.	8
Eligibility criteria	6	Specify characteristics of the sources of evidence used as eligibility criteria (e.g., years considered, language, and publication status), and provide a rationale.	7-8
Information sources*	7	Describe all information sources in the search (e.g., databases with dates of coverage and contact with authors to identify additional sources), as well as the date the most recent search was executed.	8
Search	8	Present the full electronic search strategy for at least 1 database, including any limits used, such that it could be repeated.	8
Selection of sources of evidence†	9	State the process for selecting sources of evidence (i.e., screening and eligibility) included in the scoping review.	9
Data charting process‡	10	Describe the methods of charting data from the included sources of evidence (e.g., calibrated forms or forms that have been tested by the team before their use, and whether data charting was done independently or in duplicate) and any processes for obtaining and confirming data from investigators.	9
Data items	11	List and define all variables for which data were sought and any assumptions and simplifications made.	9

St. Michael's
Inspired Care. Inspiring Science.

For peer review only - http://bmjopen.bmj.com/site/about/guidelines.xhtml

SECTION	ITEM	PRISMA-ScR CHECKLIST ITEM	REPORTED ON PAGE #
Critical appraisal of individual sources of evidence§	12	If done, provide a rationale for conducting a critical appraisal of included sources of evidence; describe the methods used and how this information was used in any data synthesis (if appropriate).	9-10
Synthesis of results	13	Describe the methods of handling and summarizing the data that were charted.	9
**RESULTS**			
Selection of sources of evidence	14	Give numbers of sources of evidence screened, assessed for eligibility, and included in the review, with reasons for exclusions at each stage, ideally using a flow diagram.	10 (Figure 1)
Characteristics of sources of evidence	15	For each source of evidence, present characteristics for which data were charted and provide the citations.	10
Critical appraisal within sources of evidence	16	If done, present data on critical appraisal of included sources of evidence (see item 12).	11
Results of individual sources of evidence	17	For each included source of evidence, present the relevant data that were charted that relate to the review questions and objectives.	11 (Table 2 & 3
Synthesis of results	18	Summarize and/or present the charting results as they relate to the review questions and objectives.	11-18
**DISCUSSION**			
Summary of evidence	19	Summarize the main results (including an overview of concepts, themes, and types of evidence available), link to the review questions and objectives, and consider the relevance to key groups.	18-21
Limitations	20	Discuss the limitations of the scoping review process.	21
Conclusions	21	Provide a general interpretation of the results with respect to the review questions and objectives, as well as potential implications and/or next steps.	21
**FUNDING**			
Funding	22	Describe sources of funding for the included sources of evidence, as well as sources of funding for the scoping review. Describe the role of the funders of the scoping review.	22-23

JBI = Joanna Briggs Institute; PRISMA-ScR = Preferred Reporting Items for Systematic reviews and Meta-Analyses extension for Scoping Reviews.

* Where *sources of evidence* (see second footnote) are compiled from, such as bibliographic databases, social media platforms, and Web sites.

† A more inclusive/heterogeneous term used to account for the different types of evidence or data sources (e.g., quantitative and/or qualitative research, expert opinion, and policy documents) that may be eligible in a scoping review as opposed to only studies. This is not to be confused with *information sources* (see first footnote).

‡ The frameworks by Arksey and O'Malley (6) and Levac and colleagues (7) and the JBI guidance (4, 5) refer to the process of data extraction in a scoping review as data charting.

§ The process of systematically examining research evidence to assess its validity, results, and relevance before using it to inform a decision. This term is used for items 12 and 19 instead of "risk of bias" (which is more applicable to systematic reviews of interventions) to include and acknowledge the various sources of evidence that may be used in a scoping review (e.g., quantitative and/or qualitative research, expert opinion, and policy document).

From: Tricco AC, Lillie E, Zarin W, O'Brien KK, Colquhoun H, Levac D, et al. PRISMA Extension for Scoping Reviews (PRISMAScR): Checklist and Explanation. Ann Intern Med. 2018;169:467–473. doi: 10.7326/M18-0850.

For peer review only - http://bmjopen.bmj.com/site/about/guidelines.xhtml
